# The first annotated checklist of the bees of Colombia reveals an overlooked diversity and areas with major data deficiencies

**DOI:** 10.7717/peerj.21684

**Published:** 2026-10-01

**Authors:** Ruben D. Martín-Rojas, Diego A. Guevara, Juan Diego Maldonado Cepeda, Nicolás David Gutiérrez Rubiano, Danny Vélez, Victor H. Gonzalez, Rodulfo Ospina-Torres, Neil S. Cobb, M Lucia Aguilar-Benavides, Eduardo A.B. Almeida, Julián Álvarez Romero, María José Arenas-Herrera, Jose Luis Benavides-Lopez, M. Argenis Bonilla-Gómez, Nelson A. Canal, Nancy S. Carrejo, Cindy J. Celis, Juan Camilo Dorado-Suarez, Giovanny Fagua, Delly Rocío García-Cárdenas, Victor Alfonso García-Moya, Dario Guevara-Plata, Fernando Fernández, Luz Miryam Gómez-P, Roberto J. Guerrero, Maria R. Manzano, Juan Diego Herrera-Manrique, Mario Alejandro Marín, Gabriel R. Navas-S, Jhon César Neita Moreno, Santiago R. Ramírez, Maria Cristina Gallego Ropero, Mateo Restrepo-Rúa, Luis F. Sánchez-Alvarán, Antonella Sardi-Saavedra, Valentina Sarria-Rodríguez, Paula Andrea Sepúlveda Cano, Ángela Soto-Correa, Edwin Daniel Torres Pineda, Juliana Torres-Toro, Elder A. Vásquez-Lenis, María C. Vélez Naranjo, Erika Valentina Vergara-Navarro, Marta Wolff, Laura Melissa Guzman, Margarita M. López-Uribe

**Affiliations:** 1 Department of Entomology, Pennsylvania State University, University Park, PA, United States of America; 2 Departamento de Biologia, Faculdade de Filosofia, Ciências e Letras de Ribeirão Preto, Universidade de São Paulo, Ribeirão Preto, São Paulo, Brazil; 3 Laboratorio de Abejas Silvestres LABUN, Departamento de Biología, Universidad Nacional de Colombia, Bogotá, Cundinamarca, Colombia; 4 Programa de Ecosistemas y Recursos Naturales, Instituto Amazónico de Investigaciones Científicas SINCHI, Bogotá D.C, Colombia; 5 Undergraduate Biology Program, University of Kansas, Lawrence, KS, United States of America; 6 Biodiversity Outreach Network, Flagstaff, AZ, United States of America; 7 Grupo Biodiversidad y Ecologia de Abejas Silvestres (BEAS), Facultad de Ciencias Básicas y Aplicadas, Universidad Militar Nueva Granada, Cajicá, Cundinamarca, Colombia; 8 Semillero SEIRPA, Grupo de Sistemática Molecular, Laboratorio de Entomología, Departamento de Biología, Pontifica Universidad Javeriana, Bogotá, Cundinamarca, Colombia; 9 Museo Entomológico Francisco Luis Gallego, Facultad de Ciencias, Universidad Nacional de Colombia, Medellín, Antioquia, Colombia; 10 Departamento de Ciencias Biológicas, Universidad de Los Andes, Bogotá, Cundinamarca, Colombia; 11 Grupo de investigación Biología de Organismos Tropicales-BIOTUN, Departamento de Biología, Facultad de Ciencias, Universidad Nacional de Colombia, Bogotá, Cundinamarca, Colombia; 12 Facultad de Ingeniería Agronómica, Universidad del Tolima, Ibagué, Tolima, Colombia; 13 Museo de Entomología de la Universidad del Valle, Departamento de Biología, Universidad del Valle, Cali, Valle del Cauca, Colombia; 14 Departamento de Zoologia, Universidade Federal do Paraná, Curitiba, Parana, Brazil; 15 Área de Entomologia, Museu de História Natural Capão da Imbuia, Curitiba, Parana, Brazil; 16 Programa de Biología, Universidad del Quindío, Armenia, Quindío, Colombia; 17 Mestrando em Ecologia e Recursos Naturais, Universidade Estadual do Norte Fluminense Darcy Ribeiro, Campos dos Goytacazes, Rio de Janeiro, Brazil; 18 Departamento de Biología, Museo de Historia Natural, Universidad del Cauca, Popayán, Cauca, Colombia; 19 Instituto de Ciencias Naturales, Universidad Nacional de Colombia, Bogotá, Cundinamarca, Colombia; 20 Grupo Bioforense, Facultad de derecho y ciencias forenses, Coleccion entomologica, Tecnológico de Antioquia institucion Universitaria, Medellín, Antioquia, Colombia; 21 Universidad del Magdalena, Santa Marta, Magdalena, Colombia; 22 Departamento de Ciencias Agrícola, Universidad Nacional de Colombia, Palimara, Valle del Cauca, Colombia; 23 Programa de Biología, Universidad de Cartagena, Cartagena, Bolívar, Colombia; 24 Colecciones de Entomología e Invertebrados - Centro de Colecciones y Gestión de Especies, Dirección de Conocimiento, Instituto de Investigación de Recursos Biológicos Alexander von Humboldt, Villa de Leyva, Boyaca, Colombia; 25 Department of Evolution and Ecology, University of California, Davis, CA, United States of America; 26 Colección de Insectos, Universidad del Quindio, Armenia, Quindío, Colombia; 27 Grupo de Entomología, Universidad de Antioquia, Medellín, Antioquia, Colombia; 28 Red de Diversidad Biológica del Occidente Mexicano, Centro Regional del Bajío, Instituto de Ecología A.C, Pátzcuaro, Michoacán, Mexico; 29 Colecciones Biológicas, Universidad CES, Medellín, Antioquia, Colombia; 30 Colección Taxonómica Nacional de Insectos “Luis María Murillo” (CTNI), Corporacion Colombiana de Investigacíon Agropecuaria (Agrosavia), Tibaitatá, Cundinamarca, Colombia; 31 Department of Entomology, Cornell University, Ithaca, NY, United States of America

**Keywords:** Neotropic, Biodiversity, Distribution, Pollinators, New records, Andrenidae, Apidae, Colletidae, Halictidae, Megachillidae

## Abstract

**Background:**

Most existing knowledge about bee biodiversity comes from temperate regions, where diversity is presumed highest—yet substantial data gaps across the tropics confound this pattern. Checklists are critical for documenting species presence, identifying knowledge gaps, and guiding conservation. We present the first comprehensive bee checklist for Colombia, a country in tropical northern South America that serves as a key biogeographic bridge between Central and South America. We hypothesize that Colombia’s complex topography, its position at the intersection of multiple biogeographic regions, and its partial Gondwanan origin contribute to exceptionally high bee diversity and endemism.

**Methods:**

We built a network of local entomological collections to compile specimen data (*n* = 62,158), combined with occurrence records from public datasets (*n* = 25,520) and species records reported in the taxonomic literature (*n* = 297). Our objectives were to: (1) synthesize the first bee species checklist for Colombia and their genus-level biological traits (ecoregional distribution, nesting habitat, sociality), (2) characterize diversity patterns at the department level using species richness, Shannon index, and weighted endemism, (3) estimate undescribed species via rarefaction curves, and (4) compare species richness and phylogenetic diversity (Faith’s phylogenetic diversity, mean pairwise distance (MPD), mean nearest taxon distance (MNTD) and standardized effect sizes of MPD and MNTD) with countries of Gondwanan origin (Brazil, Chile) *versus* those in the Holarctic region (USA).

**Results:**

We report 738 species across 123 genera and five families, including 246 new country records and 140 endemic species (19% of total fauna). Sampling effort was highly uneven: 41% of the records were concentrated in the Andean region, while xeric areas—where highest diversity is expected—remain critically undersampled. Species in the family Apidae dominated assemblages (69%), and the Andes ecoregion held the highest richness and endemism. Biological traits across the 738 species showed that solitary (57%) and above-ground nesting (57%) strategies predominate. Accumulation curves suggested that ∼26% of the bee fauna remains undescribed or unreported. Phylogenetic analyses did not support the hypothesis of higher diversity in Colombia relative to other countries of Gondwanan or Holarctic origin. We also found weak support for the relationship between species richness and geographic area across countries.

**Conclusions:**

This study provides essential baseline knowledge for ecological, taxonomic, and conservation research of Neotropical bees. Geographic sampling biases highlight urgent needs for surveys in undersampled regions, especially xeric ecosystems. The multi-institutional network developed here demonstrates the value of collaborative frameworks for biodiversity documentation in megadiverse tropical regions and lays the foundation for long-term pollinator monitoring and conservation.

## Introduction

Bees comprise a diverse group of pollinating insects with close to 21,000 described species distributed in all the continents, except Antarctica (Michener, 2007; Almeida et al., 2023; Ascher & Pickering, 2025). These insects exhibit remarkable variation in biological traits, displaying a spectrum of social behaviors, ranging from solitary to eusocial, as well as parasitic, nocturnal, and crepuscular lifestyles (Michener, 1974; Danforth, Minckley & Neff, 2019). Bees also vary widely in the specificity of their pollen foraging behavior, ranging from strict specialists that collect pollen from flowers belonging to a single plant genus to generalists (Müller et al., 2006). These pollinating insects also exhibit varied nesting habits, including soil excavators, wood excavators, renters, and above-ground builders (Danforth, Minckley & Neff, 2019). The close ecological and evolutionary associations between bees and flowering plants make these insects the most dominant pollinators in both natural and managed ecosystems (Willmer, 2011; Ollerton, Winfree & Tarrant, 2011). Indeed, nearly 90% of the flowering plants in natural ecosystems benefit from bee pollination (Ollerton, Winfree & Tarrant, 2011; Ollerton, 2017; Tong et al., 2023), and about one-third of crops require bees to set fruits (Khalifa et al., 2021). It is estimated that the value of pollination service to the world economy is at least ∼$235 billion annually (Potts et al., 2016).

Phylogenetically, bees form a monophyletic group comprising seven major lineages, commonly recognized as families (Michener, 2007; Almeida et al., 2023). In contrast to the general biodiversity pattern observed in most animal and plant taxa where species richness peaks in tropical regions, bees exhibit an ‘antitropical’ pattern where species richness peaks in warm xeric areas of subtropical regions (Michener, 1979; Orr et al., 2021; Freitas et al., 2022). It is hypothesized that this pattern reflects their evolutionary origin in the arid regions of western Gondwana and would represent a case of long-term niche conservatism (Almeida et al., 2023). However, the antitropical species diversity pattern may be partially driven by data deficiency, as most tropical regions worldwide remain understudied (Orr et al., 2021; Hughes et al., 2021; Vélez & Vivallo, 2024).

Another consideration when characterizing regional biodiversity and species assemblages is that species richness is only one of the many metrics used to describe these patterns. Phylogenetic diversity metrics facilitate a more comprehensive characterization of a region’s biological diversity by integrating the evolutionary distance among lineages into measures of biodiversity (Faith, 1992). Patterns of species richness and phylogenetic diversity do not always spatially coincide (Davies & Buckley, 2011; Fritz, Rahbek & Linder, 2012; Tietje et al., 2023; Pinkert et al., 2025). In this context, the Neotropical region harbors approximately 5,000 described bee species, although the total diversity is estimated to exceed 15,000 species (Freitas et al., 2009; Moure, Urban & Melo, 2022). Some early diverging lineages of the families Apidae (Manueliini, various Eucerinae, and Apinae) (Almeida et al., 2023), Andrenidae (Bossert et al., 2022; Ramos, Martins & Melo, 2022), Colletidae (Almeida et al., 2012), Halictidae (Danforth et al., 2008), and Megachilidae (Neofideliinae, Lithurginae) (Litman et al., 2011; Packer, Litman & Praz, 2017) are all found in South America, which likely increases the bee phylogenetic diversity of the Neotropical bee fauna. Indeed, 18 bee tribes are endemic to the Neotropics, and an additional 14 are restricted to the Western Hemisphere (Almeida et al., 2025). This combination of high endemism and the presence of early divergent lineages poses the hypothesis that bee faunas in South America (Gondwanan origin) may show higher phylogenetic diversity than expected by chance.

The geographic location of Colombia offers a unique biogeographic spot to study biodiversity within the Neotropical region. This unique location makes Colombia the most species-rich country worldwide in butterflies (Garwood et al., 2022), orchids (Betancur et al., 2015) and birds (Avendaño et al., 2021). It is estimated that Colombia harbors 10% of global biodiversity despite occupying only 0.8% of the terrestrial land cover (Arbeláez-Cortés, 2013). Located in northern South America, Colombia serves as a crucial land bridge between Central and South America, facilitating the interchange of faunas of both Gondwanan and Holarctic origins (Bacon et al., 2015). Additionally, the Andes cordillera in Colombia is divided into three mountain ranges, generating a topographic complexity with multiple valleys isolated by high mountain ridges that vary in altitude, reaching its highest elevation at 5,775 m (Hazzi et al., 2018). These altitudinal gradients cause rapid climatic variation over short geographic distances, particularly in temperature, leading to distinct vegetation structures and a high diversity of habitats (Van Wyngaarden & Fandiño Lozano, 2005; Rahbek et al., 2019). All these unique geographic and climatic features collectively support numerous microhabitats, contributing to over 300 distinct ecosystems across the country (Van Wyngaarden & Fandiño Lozano, 2005).

Lastly, Colombia also harbors a confluence of several distinct eco regions with unique faunas. In addition to the Andean region, Colombia’s biodiversity is also shaped by fauna from the Caribbean coast, the Pacific coast, the Amazon basin, the Orinoco basin (González-Orozco, 2021) and the Sierra Nevada de Santa Marta, a “mountain island” surrounded by a large expanse of tropical dry forest, independent of the Andean system (Hoorn et al., 1995). Each of these regions harbors distinct faunistic compositions influenced by climatic conditions, elevation gradients and geographic isolation, consequently fostering high levels of endemism and species richness (Myers et al., 2000; Kattan et al., 2004).

Previous species lists have reported between 466 and 604 bee species in Colombia (Smith-Pardo, 2003; Vélez-Ruiz, 2009). However, these figures are likely underestimates of the actual number of described species in the country, which is expected to be closer to 1,400 species (Dorey et al., 2026). Inventories of bee diversity in Colombia are scarce and are greatly hindered by the lack of available taxonomic tools for specimen identification in the region. Additionally, there are some persistent biases in the existing data. Previous studies have predominantly focused on specific ecosystems, such as the high-Andean ecosystems (Gonzalez & Engel, 2004; Gonzalez, Velez-Ruiz & Engel, 2014; Turley et al., 2026) and tropical dry forests (Poveda-Coronel, Riaño Jiménez & Cure, 2018; Flórez-Gómez, Maldonado-Cepeda & Ospina-Torres, 2020). In contrast, lower-elevation Andean rain forests, xeric regions, the Orinoco and the Amazon basins remain largely unstudied. Significant knowledge gaps exist for non-corbiculate bees, which constitute approximately 40% of the country’s known bee fauna (Cortés-Gómez, Rico-Aristizábal & Amarillo-Suarez, 2023; Morales-Rozo et al., 2025). The lack of comprehensive knowledge on bee biodiversity across several groups and regions poses major challenges for their conservation and management (Vogel Ely et al., 2017). Given that species are the fundamental units of conservation (Hortal et al., 2015), developing comprehensive species checklists for specific regions is the first step to generate baseline information to characterize the species present in an area, track changes in their distributions, and identify species of conservation concern. Furthermore, checklists can highlight taxonomic groups in need of revision and improved identification tools (Bartomeus et al., 2019). Such species lists can be compiled by integrating records from public databases (*e.g.*, GBIF), collaborative networks of museums and entomological collections, and taxonomic literature revisions (Gibbs et al., 2017; Kilpatrick et al., 2020; Ghisbain et al., 2023; Ghisbain et al., 2025; Bartholomew et al., 2024; Wilson et al., 2025).

In this study, we document bee diversity and their biological traits (ecoregional distribution, nesting habitat, sociality) in Colombia by synthesizing information from entomological collections, taxonomic literature, and public datasets. To achieve this, we established a collaborative network encompassing 18 entomological collections across the country. Here, we (1) report the first annotated checklist of the bees of Colombia, (2) characterize the patterns of bee diversity at the department level (administrative units) by estimating species richness, exponential of the Shannon index, and weighted endemism for each department, (3) estimate the number of undescribed species using species rarefaction curves, and (4) compare the levels of species richness and phylogenetic diversity (Faith’s phylogenetic diversity, mean pairwise distance (MPD), mean nearest taxon distance (MNTD) and standardized effect sizes of MPD and MNTD) in Colombia with other countries of Gondwanan (Brazil, Chile) and Holarctic origin (USA). We tested the hypothesis that countries of Gondwanan origin exhibit higher bee diversity than countries of Holarctic origin against the null hypothesis that differences in bee diversity are explained by the geographic area of each country.

## Materials and Methods

### Network of entomological collections in Colombia

We built a collaborative network of museums and entomological collections throughout the country under the project “Proyecto Abejas de Colombia” (Fig. 1). This network was established to foster national cooperation among institutions, with the aim of advancing knowledge of Colombian bee diversity, promoting the digitization of collection data, and facilitating international collaboration. Collections were visited between March 2019 and April 2025. We exclusively included specimen information from individuals that were properly curated, mounted, and labeled with comprehensive collection data (department, municipality, collector, and date) and an associated catalog number. For collections with existing digital databases, we identified specimens to species, updated taxonomic records, and, in some cases, verified existing identifications. For undigitized collections, we first identified previously unidentified specimens using taxonomic literature (Table S1). Subsequently, the collection curator digitized the material in accordance with Darwin Core standards before sharing the dataset for inclusion in this study (Wieczorek et al., 2012). The association between specimen records and catalog numbers enhances information traceability and facilitates future updates to the data presented here. Among all the material reviewed, we found countless specimens that are likely new species to science. However, we did not describe any new species for the country since this is beyond the scope of this study. We consider that species descriptions should be carried out within the framework of taxonomic revisions for different groups. From all the entomological collections, we collected 62,158 records from 520 species (Fig. 2).

**Figure 1:**
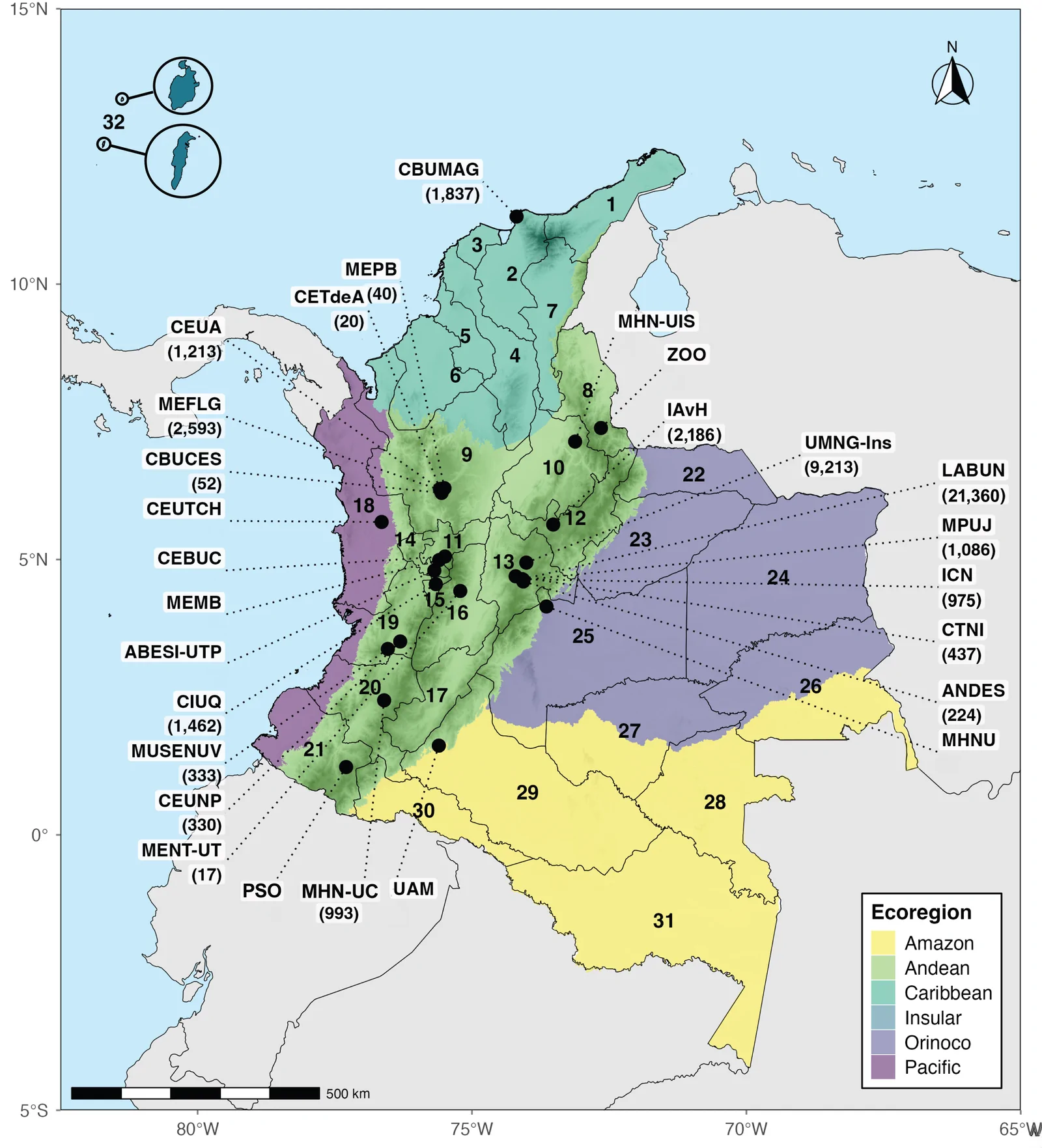
Map of Colombia depicting departments, eco regions, and entomological collections. Colors indicate the six main eco regions: Amazon, Andean, Caribbean, Insular, Orinoco, and Pacific. Departments are numbered as follows: 1. La Guajira, 2. Magdalena, 3. Atlántico, 4. Bolívar, 5. Sucre, 6. Córdoba, 7. Cesar, 8. Norte de Santander, 9. Antioquia, 10. Santander, 11. Caldas, 12. Boyacá, 13. Cundinamarca, 14. Risaralda, 15. Quindío, 16. Tolima, 17. Huila, 18. Chocó, 19. Valle del Cauca, 20. Cauca, 21. Nariño, 22. Arauca, 23. Casanare, 24. Vichada, 25. Meta, 26. Guainía, 27. Guaviare, 28. Vaupés, 29. Caquetá, 30. Putumayo, 31. Amazonas, 32. San Andrés, Providencia y Santa Catalina. All entomological collections shown on the map are part of the “Proyecto Abejas de Colombia”. Collections marked with specimen numbers contributed data to this article, while those without numbers are network members that did not contribute specimens to this study. Collections are indicated by round black symbols on the map and correspond to the following institutions: LABUN, Laboratorio de Abejas, Universidad Nacional de Colombia; Sede Bogotá (Cundinamarca); UMNG-Ins, Colección Entomológica de la Facultad de Ciencias, Universidad Militar Nueva Granada (Cundinamarca); MEFLG, Museo Entomológico Francisco Luis Gallego, Universidad Nacional de Colombia, Sede Medellín (Antioquia); IAvH, Instituto Alexander von Humboldt, Instituto de Investigación de Recursos Biológicos Alexander von Humboldt (Boyacá); CBUMAG, Centro de Colecciones Biológicas, Universidad del Magdalena (Magdalena); CIUQ, Colección de Insectos de la Universidad del Quindío (Quindío); CEUA, Colección Entomológica, Universidad de Antioquia (Antioquia); MPUJ, Museo Javeriano de Historia Natural, Pontificia Universidad Javeriana (Cundinamarca); MHN-UC, Museo de Historia Natural, Universidad del Cauca (Cauca); ICN, Instituto de Ciencias Naturales, Universidad Nacional de Colombia, Sede Bogotá (Cundinamarca); CTNI, Colección Taxonómica Nacional de Insectos “Luis María Murillo”, Corporación colombiana de investigación agropecuaria AGROSAVIA (Cundinamarca); MUSENUV, Museo de Entomología de la Universidad del Valle (Valle del Cauca); CEUNP, Colección Entomológica, Universidad Nacional de Colombia, Sede Palmira (Valle del Cauca); ANDES, Museo de Historia Natural C.J. Marinkelle, Universidad de los Andes (Cundinamarca); CBUCES, Colección de Artrópodos Terrestres, Universidad CES (Antioquia); MEPB, Museo Entomológico Piedras Blancas, Comfenalco, Antioquia (Antioquia); CETdeA, Colección Entomológica, Tecnológico de Antioquia (Antioquia); MENT-UT, Museo Laboratorio de Entomología, Universidad del Tolima (Tolima); CEBUC, Colección Entomológica del Programa de Biología, Universidad de Caldas (Caldas); MEMB, Museo Entomológico “Marcial Benavides”, Centro Nacional de Investigaciones de Café (Caldas); ABESI-UTP, Colección Abejas Silvestres, Universidad Tecnológica de Pereira (Risaralda); MHN-UIS, Museo de Historia Natural, Universidad Industrial de Santander (Santander); CEUTCH, Colección Entomológica, Universidad Tecnológica del Chocó (Chocó); UAM, Museo de Historia Natural, Universidad de la Amazonia (Caquetá); PSO, Colección Zoológica, Universidad de Nariño (Nariño); ZOO, Colección Zoología General, Universidad de Pamplona (Norte de Santander); MHNU, Museo de Historia Natural, Universidad de los Llanos (Meta).

### Literature review

We conducted a systematic review of the taxonomic literature that included original bee species descriptions from Colombia, taxonomic revisions that included groups found in Colombia and notes on new species records (Table S1). Additionally, we reviewed all species reported in Moure’s Catalogue of Bees in the Neotropical Region (Moure, Urban & Melo, 2022) to retrieve information from the literature about undigitized records of species and specimens reported for Colombia. From these records, we extracted locality data, including department, municipality, and geographic coordinates (when available), as well as the collector’s name, date of collection, the institution where the material was deposited, and catalog number (when available). From the literature review, we collected 297 records from 155 species (Fig. 2).

**Figure 2:**
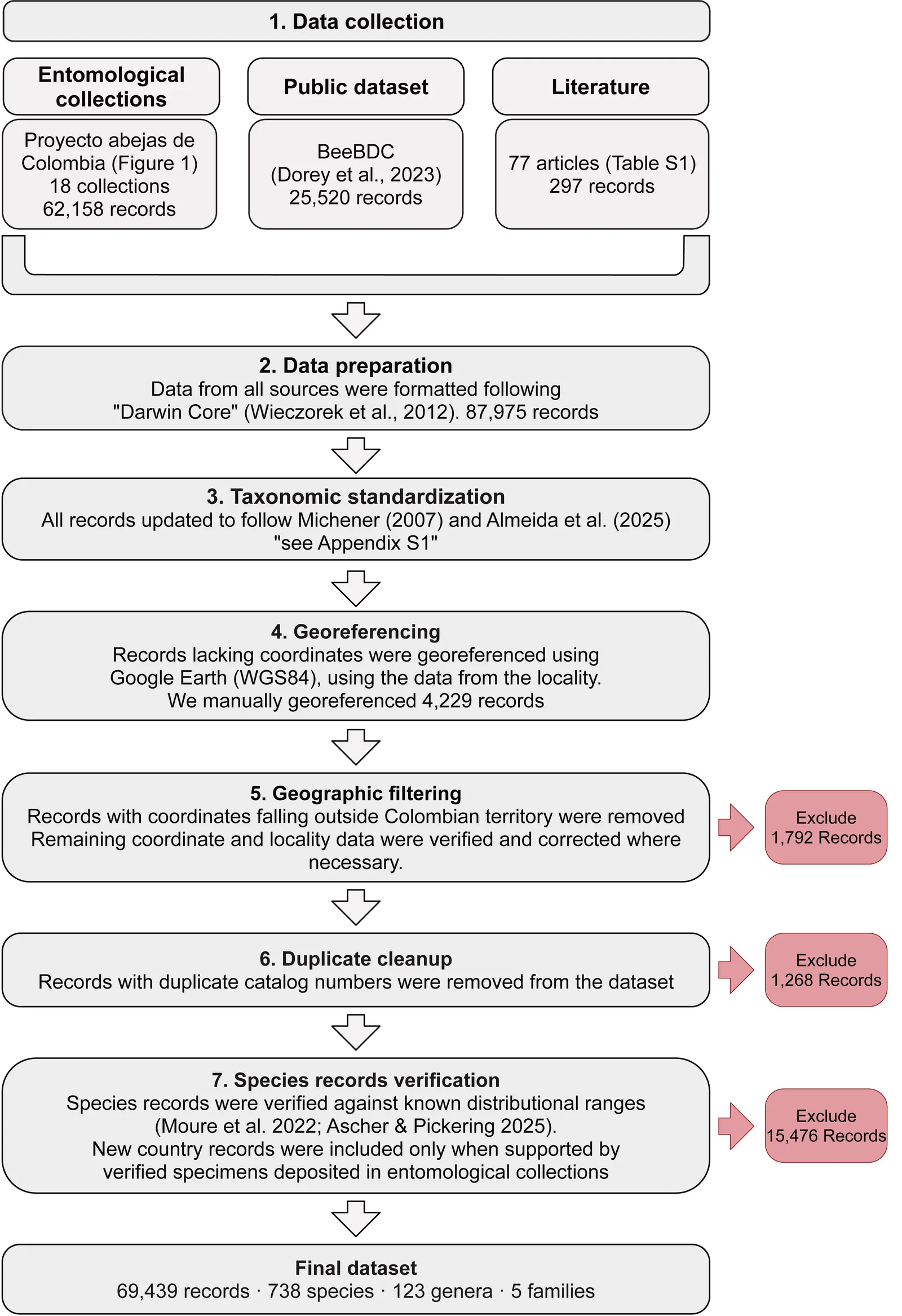
Workflow followed to assemble and clean the databases from the three data sources: entomological collections, public repositories, and taxonomic literature. Records were then processed through seven sequential cleaning steps: (1) data preparation, (2) taxonomic standardization, (3) georeferencing, (4) geographic filtering, (5) duplicate cleanup, and (6) species-level verification. The final dataset comprises 69,439 records representing 738 species, 123 genera, and five families.

### Public datasets

We incorporated occurrence records from the R package BeeBDCv1.3.3 (Dorey et al., 2023). This package compiles approximately 20 million global bee records from public repositories such as the Global Biodiversity Information Facility (GBIF), Symbiota Collections of Arthropods Network (SCAN), Integrated Digitized Biocollections (iDigBio), iNaturalist, the United States Geological Survey (USGS), and the Atlas of Living Australia (ALA). From the BeeBDC database, we filtered the occurrences to include only records from Colombia. Additional records from nine insect collections included in the BeeBDC package were manually added because those records were excluded from the repository due to the lack of specific geographic coordinate information (Dorey et al., 2023). For these records, we manually added georeferenced information based on locality data using Google Earth, and coordinates were recorded in the WGS84 geographic coordinate system. Note that records from public repositories and databases were exclusively used as occurrence data for species already reported in the country from specimens in entomological collections or documented in primary literature. From this public dataset, we used 25,520 records from 400 species (Fig. 2).

### Taxonomic classification

We primarily followed the traditional taxonomic classification proposed by Michener (2007) with modifications supported by recent phylogenetic studies as summarized by Almeida et al. (2025) for the families, subfamilies, and tribes included in this checklist (see Appendix S1). For the stingless bees, we followed Moure’s Catalogue of Bees in the Neotropical Region Moure, Urban & Melo (2022), except for *Nanoplebeia* (Engel, 2021), *Exochotrigona* Engel 2019, and *Parapartamona* Schwarz 1948. Based on recent phylogenies of Meliponini (Rasmussen & Cameron, 2009; Lepeco et al., 2024), we treated the subgenera *Nanoplebeia* (Engel et al., 2021) and *Exochotrigona* (Engel et al., 2019) as genera. The genus *Parapartamona* was recognized following the proposal of Gonzalez & Smith-Pardo (2003). For *Euglossa* Latreille, 1802, we followed the subgeneric classification proposed by Engel (2021). Finally, for the clade comprising *Andinaugochlora* Eickwort, 1969, *Neocorynura* Schrottky, 1910, and *Neocorynurella* Engel, 1997 (Gonçalves, 2016), we followed Smith-Pardo & Gonzalez (2009) in treating them as a single genus under *Neocorynura*.

### Criteria for data inclusion in the checklist

We merged all data from entomological collections, public repositories and databases, and records from the literature into a single database (Table S2) following Darwin Core standards (Wieczorek et al., 2012) with minimal fields including: scientificName, family, subfamily, tribe, genus, subgenus, specificEpithet, taxonRank, scientificNameAuthorship, namePublishedInYear, identificationQualifier, typeStatus, identifiedBy, dateIdentified, decimalLatitude, decimalLongitude, verbatimElevation, continent, country, stateProvince, county, locality, eventDate, day, month, year, recordedBy, samplingProtocol, catalogNumber, institutionCode, collectionCode, datasetName,verbatimScientificName, bibliographicCitation, references, and dataSource.

After merging all datasets, we updated the taxonomy of all species records to follow the classification described above (see Appendix S1; Fig. 2). When we found duplicated records between the BeeBDC dataset and records in the entomological collections, we retained the records from the collections as these had undergone direct curatorial verification. We further curated these records using their geographic information compared to reports in the literature (Moure, Urban & Melo, 2022; Ascher & Pickering, 2025). For species previously reported for Colombia, we confirmed that the occurrence data aligned with their known ranges within the country. Species not previously reported for Colombia were included only when their presence was supported by verified specimens from insect collections (Fig. 2). Conversely, we removed species from previously published species lists (Smith-Pardo, 2003; Vélez-Ruiz, 2009) when their presence in Colombia could not be verified using data from entomological collections, public repositories and databases, and primary literature (Table S3).

We also removed species from the checklist because their generic classification remains unclear under our current understanding of bee taxonomy. This applies to *Tetralonia duplofasciata* Kriechbaumer, 1900 and *Tetralonia rufotibialis* Friese, 1925, that were originally described from Colombia but are listed as *incertae sedis* in Moure’s Catalog (Moure, Urban & Melo, 2022), either because the type specimen is lost or has not been studied to confirm their placement within a recognized genus of South American Eucerini. Finally, we included genera that are reported for the country but have no species identifications for those specimens.

### Biological trait data collection

We conducted a systematic literature review to compile data on two biological traits: sociality and nesting behavior, for all the genera in our checklist (Table S4). We conducted a Google Scholar search for each species including the species name and “nesting biology” or “sociality”. When no information was available, biological traits were assigned using information from the genus. We followed the classification of sociality of Michener (1974) and nesting behavior of Danforth, Minckley & Neff (2019), which we adapted into broad categories. For sociality, species were classified into four broad categories: solitary, variable (including communal, quasisocial and semisocial behaviour), eusocial, and brood parasite (kleptoparasite). For nesting behavior, species were classified into four broad categories: below-ground nesting (soil excavators), above-ground nesting (including wood excavators, renters, aerial, termite or ant nests), a variable category was established for genera exhibiting below-ground and above-ground strategies, and brood parasite for kleptoparasite species.

To visualize the distribution of biological traits across the Colombian bee fauna, we used the mega-phylogeny of Henríquez-Piskulich, Hugall & Stuart-Fox (2024), which includes 20,183 bee species representing 428 genera worldwide. From this tree, we derived a genus-level phylogeny by retaining only genera present in our database using the function ‘drop.tip’ from the R package ape v5.8 (Paradis & Schliep, 2019). A total of 22 genera in our checklist were absent from the phylogeny of Henríquez-Piskulich, Hugall & Stuart-Fox (2024). For these, we inserted one representative species per genus into the original species-level tree prior to genus-level pruning, using the function “bind.tip” from the R package phytools v2.5-2 (Revell, 2012). Each missing genus was placed as a polytomy in the clade of its closest relative (Qian & Jin, 2016). Phylogenetic placements were based on published molecular phylogenies specific to each group: stingless bees (Meliponini) followed Lepeco et al. (2024); Neopasiphaeinae followed Almeida et al. (2019); Caenohalictini followed Gonçalves & Melo (2010); Augochlorini followed Gonçalves, De Meira & Rosa (2022); Anthidiini followed Parizotto, Urban & Melo (2022); Tapinotaspidini followed Aguiar et al. (2020).

### Estimation of diversity indices and endemism

To characterize patterns of diversity in Colombia, we constructed a presence–absence matrix at the department level (*i.e.,* administrative units of Colombia; Fig. 1). We focused on these units because these are used for policy and conservation actions. While administrative units may not hold intrinsic biological value, they serve as discrete areas to study biodiversity, identify gaps of information and develop conservation efforts. To estimate diversity indices, we calculated species richness—the total number of species per unit—and species diversity—using species number of occurence records as reported in the databases to estimate the exponential Shannon diversity index (*q* = 1) for each department using the ‘iNEXT’ function in the R package iNEXT v3.0.2 (Chao et al., 2014). To estimate endemism, we classified species in a binary fashion as either endemic (only reported from Colombia) or more widely distributed, and calculated the weighted endemism index following Shipley & McGuire (2022) using the ‘weighted_endemism’ function of the R package phyloregion v1.0.9 (Daru, Karunarathne & Schliep, 2020).

### Estimation of undescribed species number based on rarefaction curves

To estimate the approximate number of species that remained to be described in the country, we used a species accumulation curve approach using the ‘iNEXT’ function in the R package iNEXT v3.0.2 (Chao et al., 2014). This approach is typically used to determine whether a community has been sufficiently sampled to capture its diversity (Soberón & Llorente, 1993), but has also been implemented to estimate undescribed diversity at biome and country levels (Ter Steege et al., 2016; Piepenbring et al., 2020; Dorey et al., 2026). We used these species accumulation curves to estimate undescribed species at the country and department level. To visualize results, we constructed maps for these diversity metrics using shape files available from the Colombian geological services “Instituto Geográfico Agustín Codazzi” (https://www.colombiaenmapas.gov.co/) using the R package ggplot2 v4.0.0 (Wickham, 2009) and sf v 1.1-0 (Pebesma & Bivand, 2023).

### Estimates of phylogenetic diversity metrics

To test the hypothesis that Colombia’s bee fauna exhibits higher phylogenetic diversity than other other countries of Gondwanan (Brazil, Chile) *versus* those from the Holarctic (USA), we estimated phylogenetic diversity metrics as Faith’s Phylogenetic Distance (PD) (Webb et al., 2002), mean pairwise distance (MPD), and mean nearest taxon distance (MNTD) (Qian & Jin, 2021), along with the standardized effect sizes of MPD and MNTD (Kembel et al., 2010). These countries were selected because they represent well-studied regional faunas of Gondwanan origin in contrast to those associated with the Holarctic region, enabling a comparative evaluation of deviations from the expected PD–richness relationship. To calculate diversity metrics from different countries, we first downloaded the checklists of the bee faunas from the US, Chile and Brazil from Discover Life (Ascher & Pickering, 2025). Additionally, we downloaded the global checklist, which served as a null model capturing all bee species worldwide. To homogenize taxonomy across datasets, we standardized the US, Brazil, and Chile lists to match the same genus-level classification used in our Colombian checklist.

To estimate phylogenetic distance among taxa, we used the mega-phylogeny of bees by Henríquez-Piskulich, Hugall & Stuart-Fox (2024). To include species in our datasets that were not represented in the phylogeny, we added all missing species at the genus level as polytomies using the ‘congeneric.merge’ function in the R package pez v1.2.5 (Pearse et al., 2015). This was necessary because 8,404 species in our analysis lacked sequences in the mega-phylogeny of bees, and previous studies have shown that phylogenetic diversity metrics derived from such trees with resolution at the genus level are virtually identical to those from fully resolved species-level phylogenies (Qian & Jin, 2021). The final phylogeny included 19,898 species in total (Null model: 19,898 spp.; Brazil: 1,876 spp.; Chile: 458 spp.; Colombia: 697 spp.; USA: 3,583 spp.). The only species that remained unplaced in the final phylogeny were those from genera not represented in the original phylogeny, including 17 from Colombia, 30 from Brazil, 4 from the USA and 55 of our null model (Table S5).

Using this phylogeny, we calculated phylogenetic diversity (Faith’s PD) (Webb et al., 2002), mean pairwise distance (MPD), and mean nearest taxon distance (MNTD) (Qian & Jin, 2021), along with the standardized effect sizes of MPD and MNTD (Kembel et al., 2010) using the ‘pd’, ‘mpd’, ‘mntd’, ‘ses.mpd’ and ‘ses.mntd’ functions in the R package picante v1.8.2 . To evaluate the alternative hypothesis that phylogenetic diversity metrics are a function of country area, we fitted simple ordinary least squares linear regression models (*y* ∼*Area*), where *y* represents each phylogenetic diversity metric (PD, MPD, MNTD, sesMPD, sesMNTD, species richness, and number of genera) and *Area* corresponds to the total land area (km^2^) of each country. We assessed model fit using the coefficient of determination (R^2^) and tested significance with the F-statistic at *α* = 0.05. Linear models were fitted using the ‘lm’ function from the R package stats v4.5.2 (R Core Team, 2021).

## Results

After the cleanup workflow, we compiled a total of 69,439 records from the various sources (entomological collections: 44,557; online repositories and databases: 24,585; and literature reviews: 297). In total, we found records for 738 species currently classified into five families, 13 subfamilies, 38 tribes, and 123 genera (Appendix S2). The family Apidae was the most species-rich with 508 species (69 genera), followed by Halictidae with 108 (29), and Megachilidae with 67 (14). Colletidae (46 species in 9 genera) and Andrenidae (9 species in 4 genera) exhibited the lowest species diversity (Fig. 3). We found 246 new species records for the country, significantly expanding records from previous species list in 2003 and 2009 (Vélez-Ruiz, 2009; Moure, Urban & Melo, 2022; Ascher & Pickering, 2025). These new records include 168 species for Apidae, 31 for Halictidae, 18 for Colletidae, 27 for Megachilidae, and 2 for Andrenidae. *Euglossa* Latreille, 1802 and *Centris* Fabricius, 1804 were the genera with the highest species richness, with 78 and 74 species respectively (Fig. 3).

**Figure 3:**
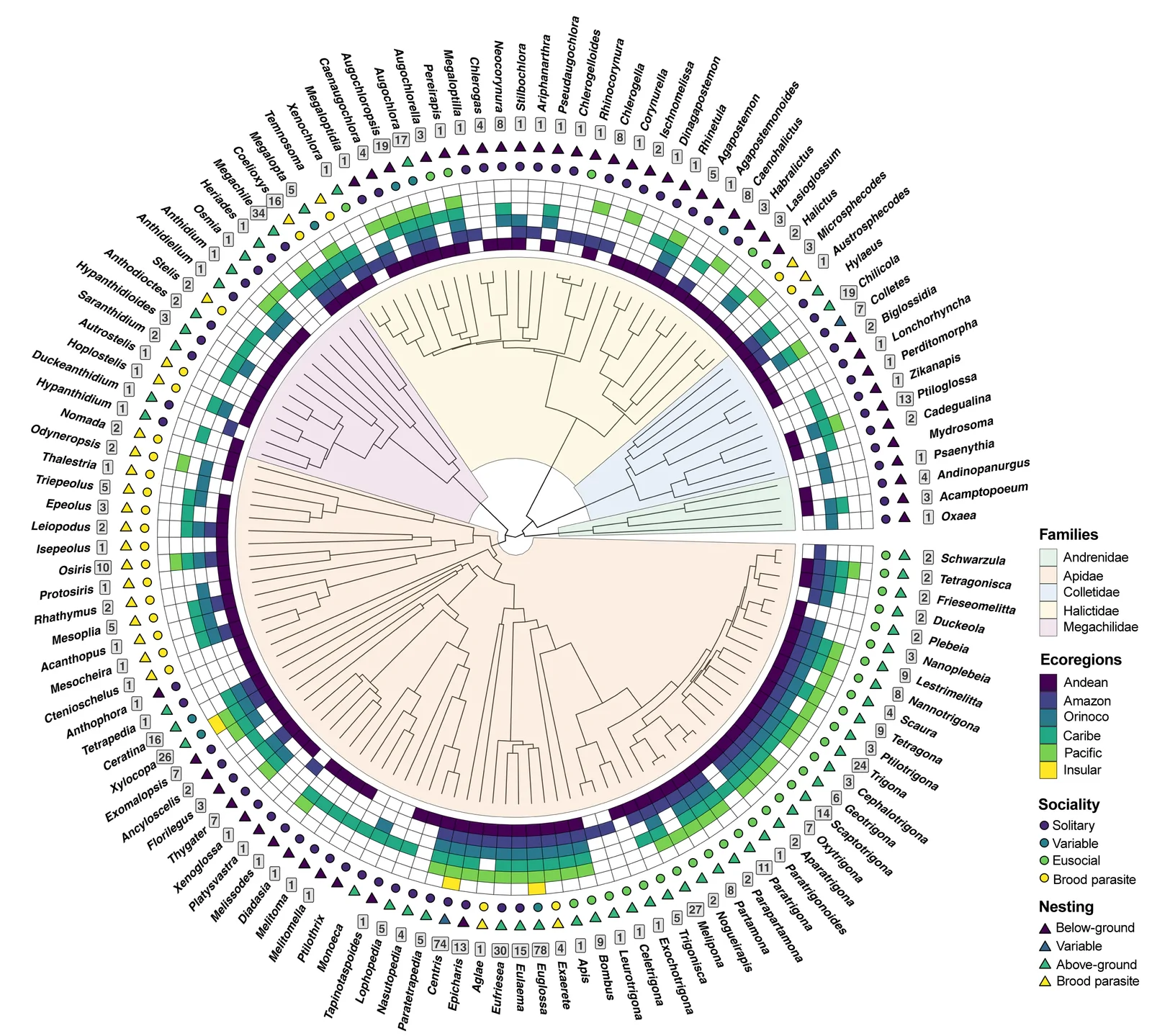
Genus-level phylogeny of Colombian bees derived from the phylogeny of Henríquez-Piskulich, Hugall & Stuart-Fox (2024), and annotated with biological traits and distributional data. The rings are arranged from the innermost to the outermost as follows: (1) Ecoregional distribution: tiles indicate presence (filled) in each of the six ecoregions defined for Colombia; (2) Social behavior: circles indicate solitary, variable, eusocial, or brood parasitoid; (3) Nesting behavior: triangles indicate below-ground, variable, above-ground, or brood parasitism. Rectangles show the number of species recorded per genus in our checklist. Genera without numbers are country records, but there are no known species. For details about trait coding, see Table S4B.

We excluded 109 species from the checklist due to the lack of supporting information from specimen records or taxonomic literature. Specifically, these exclusions comprised eight species from DiscoverLife, and 101 from Vélez-Ruiz (2009) (Table S3). Of the total species reported, 355 were represented by more than 10 occurrence records, while 198 were represented by 2–9 records. Additionally, 185 species had only a single record in our database. Notably, 107 species were reported exclusively from type material, with no subsequent observations or new records in collections. Here, we report the first native occurrence of the genus *Osmia* for South America, with *Osmia azteca* Cresson, 1878, reported for the eco region of Chocó in the department of Antioquia. Another species of this genus was previously introduced to Chile from Europe *Osmia niveata* Fabricius (1804), but no native representative had been documented for the continent until now (Alfaro et al., 2026). Additionally, the presence of the adventive species *Megachile concinna* Smith, 1879 is recorded for the first time in the Caribbean region, representing the third adventive species reported for the country (Gonzalez et al., 2019).

For behavioral traits, solitary behavior was the most common strategy, recorded in 424 species (∼57%; Fig. 3), followed by eusocial with 181 species (25%). Sixty-seven species (9%) exhibited variable sociality (*e.g.*, communal, quasisocial and semisocial). Brood parasites represented 9% of the species in our checklist and occur in three families: Apidae (42), Megachilidae (20), and Halictidae (5) (Fig. 3). For nesting habitat, above-ground nesting was the most common strategy recorded for 421 species (57%), followed by below-ground nesting in 251 (34%). The families Apidae (64%) and Megachilidae (70%) were predominantly above-ground nesting bees, while the family Andrenidae consists of below-ground nesting bees, which also was the most common nesting habit in the families Halictidae (74%) and Colletidae (44%).

We found high variability in the number of records and richness across the different ecoregions and departments. The Andean region showed the highest species richness (483 spp.) followed by the Orinoco (280 spp.), Amazon (265 spp.), Caribbean (222 spp.), Pacific (169 spp.) and Insular regions (3 spp.) (Fig. 3). At the department level, we found the highest species richness in Antioquia, Meta, Cundinamarca and Valle del Cauca with 269, 220, 207 and 200 species, respectively (Fig. 4B). On the other hand, Norte de Santander, Córdoba, Atlántico, La Guajira, Arauca, and San Andrés and Providencia presented the lowest number of species, with fewer than 35 species reported for each department. The departments of Antioquia and Cundinamarca showed the highest number of records with 14,884 and 14,046, respectively, representing 41% of the country’s data (Fig. 4A). Conversely, Córdoba, Norte de Santander, Atlántico, and San Andrés & Providencia exhibited the lowest number of records, with less than 200 per department, representing only a combined 0.6% of our dataset. We found a strong correlation between the total number of records (considered a proxy for sampling effort) and the number of species reported in each department (*R*^2^ = 0.722; *p*-value <0.001; Fig. S1).

**Figure 4:**
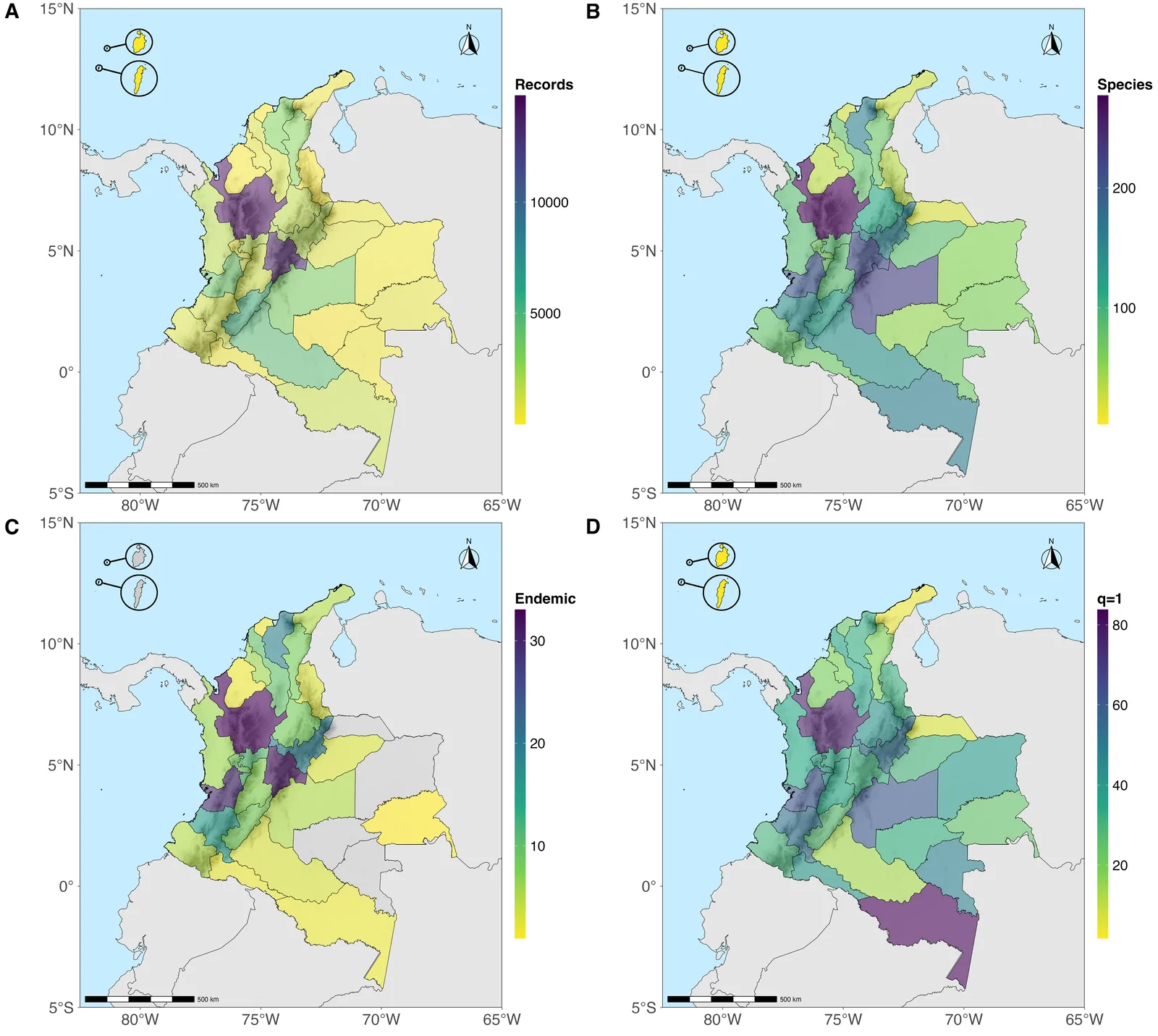
Bee diversity metrics visualized across departments in Colombia. (A) Number of occurrence records per department, (B) number of bee species documented per department, (C) weighted endemism index of bee species by department. Color gradients range from yellow (low values) to dark purple (high values), and (D) exponential Shannon diversity index (*q* = 1) per department.

The diversity estimated from the exponential of the Shannon index (Fig. 4C) showed a partially different pattern compared to species richness (Fig. 4B). Not all departments with high species richness showed high effective diversity. For instance, Antioquia showed the highest species richness in the country (269 spp.) and a high *q* = 1 value (76 spp.). In contrast, Amazonas, with lower species richness (161 spp.), showed the same *q* = 1 value (76 spp.) than Antioquia, suggesting that sampling in Amazonas has been more evenly distributed compared to Antioquia. On the other hand, Cundinamarca, despite having one of the highest species richness (207 spp.), displayed an intermediate *q* = 1 value (31 spp.), indicating a marked dominance on the species that have been sampled in this department. In contrast, Arauca, La Guajira, and San Andrés and Providencia presented the lowest *q* = 1 diversity (<7 spp.), in line with the low number of recorded species in these departments. We found that 19% of the species recorded (140) for Colombia are endemic (*i.e.,* only found in Colombia), in the families Apidae (59), Halictidae (40), Colletidae (18), Megachilidae (17) and Andrenidae (6). The departments that showed the highest number of endemic species for Colombia were Cundinamarca (35), Valle del Cauca (29), Antioquia (26), Boyacá (22), Magdalena (18) and Cauca (14) (Fig. 4D). We note that if an endemic species is present in two departments, it is included as an endemic species of both units.

The rarefaction curve revealed that the number of bee species in Colombia has not stabilized (Fig. 5A). We estimated that there are approximately 1,035 species in the country, with a completeness of 74%, meaning that 26% of the bee fauna is yet to be described or reported. As observed at the national level, species accumulation curves did not stabilize at the departmental level (Figs. 5B–5D). Even in departments with the highest species richness and number of records—such as Cundinamarca and Antioquia—the curves did not reach an asymptote (Fig. 5B). Although departments like Amazonas, Boyacá, Magdalena, Meta, Santander, and Valle del Cauca exhibited high species richness, their accumulation curves remain in a linear phase of growth (Fig. 5B), indicating underestimation of richness. This same pattern is evident across other departments (Figs. 5C and 5D). However, in many departments, the low number of records (Fig. 4A) suggests that the estimated species richness is likely an underrepresentation of the actual diversity due to limited sampling effort or data availability. The accumulation curves by family followed the same general pattern of non-stabilization observed at the national and departmental levels (Figs. 5E and 5F). Among the dominant families, Apidae showed the highest species richness, with an estimated total of 641 species, while Halictidae reached an estimated richness substantially lower, with both curves still in an active growth phase, indicating that a considerable fraction of their diversity remains undetected (Fig. 5E). The remaining families exhibited curves far from reaching an asymptote despite their lower absolute richness (Fig. 5F).

**Figure 5:**
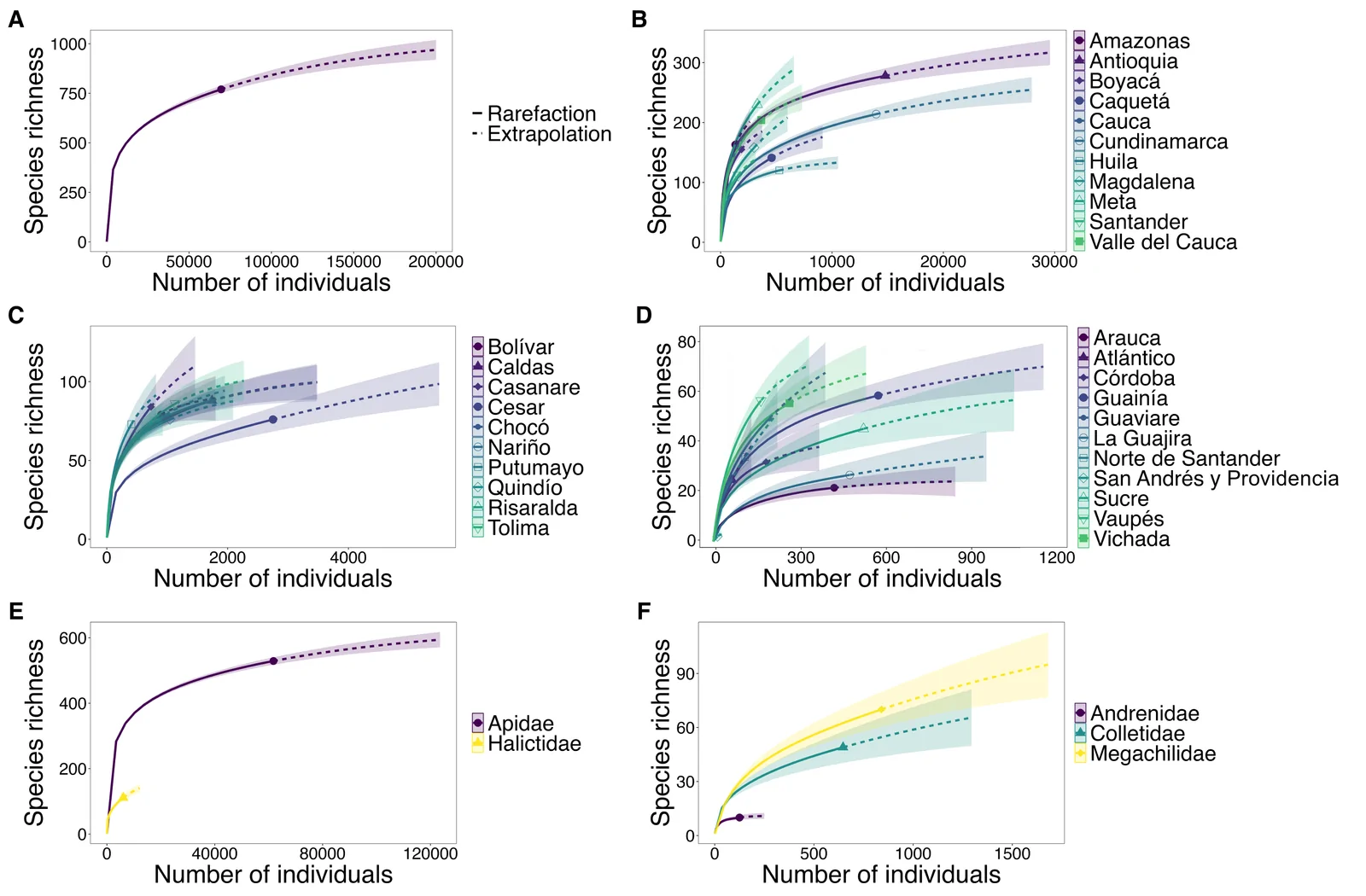
Species-rarefaction curves for Colombia, departments and families. (A) Country accumulation curve, with solid lines representing interpolated (rarefaction) richness and dashed extensions showing extrapolated richness. (B–D) Department-level curves, grouped by current total species richness: (B) departments with high species richness (>100 species), (C) departments with intermediate species richness (50–100 species), and (D) departments with low species richness (<50 species). (E–F) Family-level accumulation curves: (E) the two most speciose families, Apidae and Halictidae, and (F) the remaining families recorded in Colombia (Andrenidae, Colletidae, and Megachilidae). Ribbons indicate 95% bootstrap confidence.

Phylogenetic diversity metrics showed that Colombia exhibits lower total Faith’s PD compared to Brazil, USA, and the global dataset (Table 1) but higher compared to Chile. These patterns are expected based on the strong correlation between species richness and phylogenetic distance. However, the standardized MPD and MNTD values (sesMPD and sseMNTD), which control for differences in species richness, reveal different patterns. Colombia’s bee fauna was the most clustered compared to the faunas of other countries (Table 1). This indicated that the average evolutionary distance between pairs of species in Colombia was shorter than in the other countries. Colombia’s sesMNTD values were intermediate compared to other countries (Table 1) suggesting that the evolutionary distance between pairs of most closely related species is on intermediate distance. This pattern is typical of species assemblages with some highly species-rich genera and many genera with few species. We found a weak support for the hypothesis that species richness is highly correlated with country area (*R*^2^ = 0.8023; *p*-value = 0.1043; Fig. S2).

**Table 1:** Phylogenetic diversity metrics for Colombia, Brazil, the USA, and the world.

Country	Number of species	Number of genera	PD	MPD	MNTD	sesMPD	sesMNTD
Brazil	1,876	169	34,992.24	206.50	33.08	−26.13	−13.88
Chile	458	81	10,550.34	206.71	35.87	−12.26	−7.34
Colombia	697	112	15,255.03	191.21	36.88	−44.01	−5.98
US	3,583	110	72,025.73	210.56	38.19	−20.27	0.29
All	19,898	437	381,237.47	214.98	37.35	−3.11	5.33

**Notes.**

MNDT mean nearest taxon distance MPD mean pairwise distance PD Phylogenetic diversity sesMNTD standardized effect sizes of MNTD sesMNPD standardized effect sizes of MPD

## Discussion

Here, we present the first annotated checklist of the bees of Colombia, reporting 738 species across 123 genera (Fig. 3). This represents an increase of more than 20% in the total number of species reported compared to previous species list for the country, and also confirms and rules out the presence of several species listed in earlier studies (Smith-Pardo, 2003; Vélez-Ruiz, 2009). We also found that 19% of the bee fauna is endemic to Colombia, highlighting the country’s unique biodiversity. This study depicts the story of the Neotropical bee fauna: assemblages characterized by diversity clustered around high species rich genera that have recently diversified in this region (*e.g.*, *Euglossa* and *Centris* accounting 20% of all species) and many species-poor endemic genera that are relicts of old diversification events (*e.g.*, *Oxaea*, *Andinopanurgus*, *Chilicola, Habralictus,* and *Caenohalictus*). On the other hand, our results also show a strong geographic bias and uneven sampling coverage across different administrative units. The highest species richness, endemic species number, and records are concentrated in the Andean region (Fig. 4), home to the country’s largest universities and research centers, and where bee studies have been traditionally conducted (Cortés-Gómez, Rico-Aristizábal & Amarillo-Suarez, 2023). In contrast, departments where arid regions are concentrated (*e.g.*, La Guajira and Arauca) and high bee biodiversity is expected, remain to be thoroughly sampled. Geographically, this means that most of the collections have been conducted in only 8% (87,161 km^2^) of the Colombian territory.

The two previous species lists of Colombian bees (Smith-Pardo, 2003; Vélez-Ruiz, 2009) served as foundational references for understanding the country’s bee diversity. However, they lacked detailed geographic information, catalog numbers and location of the specimens reported, which hindered the traceability of several previously reported records. Similarly, Discover Life provided additional records to our checklist, but eight species listed in those sources could not be corroborated with associated specimens in collections or from literature. Vélez-Ruiz (2009) reported 589 valid species for Colombia, but we found no information to support the records of 101 of those species in collections, the literature reviewed, or the online databases consulted. Despite this exclusion of species from previous species lists and reports, our annotated checklist places Colombia as the fourth most bee-diverse country in South America, after Brazil (1,985 spp.), Argentina (1,183 spp.) and Peru (779) (Ascher & Pickering, 2025). This pattern follows a direct correlation between geographic area and bee biodiversity in the Americas (Fig. S3). Following the number of species reported for South American countries in DiscoverLife, the number of bee species for Colombia exceed those reported for Bolivia (590), Paraguay (501), Ecuador (473), Chile (474), Venezuela (113), and Uruguay (83) (Ascher & Pickering, 2025). Considering estimates from our accumulation curve (Fig. 5), we predict that species richness in Colombia is underestimated by at least 26%.

We did not find support for the hypothesis that Colombia exhibits higher levels of bee species and phylogenetic diversity compared to other countries of Gondwanan origin *versus* those in the Holarctic region (Table 1). However, standardized metrics of phylogenetic diversity captured the high genus richness of the Colombian bee fauna, revealing intermediate sesMNTD values in comparison to other South American countries. The Colombian fauna is characterized by the presence of species that belong to lineages that persisted in South America from their Gondwanan ancestors (*e.g.*, Augochlorini, Caenohalictini, Diphaglossinae, Neopasiphaeinae, Protandrenini, Xeromelissinae; Almeida et al., 2023) and some genera with very fast and recent diversification (*e.g.*, *Euglossa*; Ramírez et al., 2010). The MPD, MNTD and their effect sizes indicate strong phylogenetic clustering for the Colombian bee fauna. This pattern is largely explained by the overrepresentation of species in the subfamily Apinae, which account for 52% of the species recorded in Colombia. Although the Colombian and Brazilian bee faunas have more genera (123 and 208, respectively) than the USA (110), phylogenetic diversity metrics were lower in both South American countries because these metrics are strongly correlated with species richness (Qian & Jin, 2021). In contrast, Chile shows the opposite pattern with high phylogenetic diversity values despite the presence of a small number of species due to the presence of several old lineages that are endemic to this part of the world (Marshall et al., 2023). Overall, these metrics reveal that bee diversity is not evenly distributed across these faunas, and when considering South America, Chile harbors a remarkably high number of phylogenetically unique, early diverging lineages (Marshall et al., 2023; Almeida et al., 2023) (*e.g.*, Euherbstiini, Manueliini, Neffapini, Neofideliinae, and Nolanomelissini) despite having moderate species richness (474 according to Ascher & Pickering, 2025).

Although solitary species make up most of the bee diversity of the Colombian fauna (424 species), eusocial behavior accounted for 25% of the species (181 species) in our checklist, a higher proportion than reported for the USA and Europe, where 4% and 5% of their bee faunas exhibit an eusocial behavior (Cordeiro et al., 2026). Additionally, we observed the dominance of above-ground nesting (421 species) over below-ground nesting (250 species). These patterns in the natural history traits of the tropical bee fauna point to the challenges that tropical environments pose for bees nesting underground regarding the susceptibility of the larval food to spoilage due to the high humidity, and the high pressure from predators (*e.g.*, ants) (Michener, 1979; Jeanne, 1979). Eusocial species can compensate some of these threats through division of labor, which increases foraging in environment with sparse resources, and provides collective defense against nest predators and microbial pathogens that may be more common in humid environments (Cremer, Pull & Fürst, 2018; Heinrich et al., 2025). It is possible that these ecological conditions favored the dominance of Apidae in this Neotropical species assemblage, where stingless bees and bumble bees account for 25% of the species recorded in our checklist. Below-ground nesting is the most dominant trait among bees with ∼70% of all species worldwide showing this trait (Antoine & Forrest, 2021). However, we found the opposite pattern in our species assemblage, with 34% of the species nesting below-ground. These patterns contrasts greatly with the faunas from the United States and Europe, where 93% and 72% of species are below-ground nesters, respectively, and aligns more closely with Brazil, where approximately 50% of species nest above ground (Cordeiro et al., 2026). Above-ground nesting reduces exposure to moisture by avoiding direct contact with damp soil common in Neotropical habitats (Cane, 1991; Heinrich et al., 2025). The below-ground nesting solitary bees that persist in these tropical conditions have often evolved different waterproofing strategies. Oil-collecting bees (Centridini, Tapinotaspidini, Tetrapediini), for instance, line their brood cells with floral oils protecting larval provisions from fungal decay despite nesting in soil (Buchmann, 1987). Other ground-nesting bees like Colletidae waterproof their nest by lining their brood cells with a cellophane-like secretion from the Dufour’s gland (Almeida, 2008).

Recent studies documenting bee diversity in South America facilitated a re-evaluation of the presence of some species in Colombia. For example, the recent taxonomic work of the genus *Oxytrigona* Cockerell, 1917 (Lepeco, 2024) indicated that the distribution of *Oxytrigona mellicolor* (Packard, 1869) is restricted to Ecuador, and *Oxytrigona tataira* (Smith, 1863) to the northeastern region of Brazil. Consequently, both species were excluded from the species list of Colombia. However, the possible presence of *O. mellicolor* in Colombia cannot be entirely ruled out due to the proximity of its type locality (Napo, Ecuador) to the Colombian Amazon. Similarly, Melo (2023) noted that the records of *Lestrimelitta limao* (Smith, 1863) in Colombia (Guevara, Gonzalez & Ospina, 2020) do not correspond to this species, but may correspond to the recently described species *Lestrimelitta camposi* (Melo, 2023). *Nannotrigona testaceicornis* (Lepeletier, 1836) is another species that had previously been reported for Colombia (Nates-Parra & Rosso-Londoño, 2013). However, a taxonomic revision of the genus (Jaramillo, Ospina & Gonzalez, 2019) found no material confirming its presence in the country. Therefore, this species was also removed from the checklist. Additionally, *Centris sponsa* Smith, 1854, originally described from a specimen from Colombia, was not found in the most recent revision of the subgenus *Centris* (*Ptilotopus*) Klug, 1810 (Vélez & Vivallo, 2018). The authors suggested that the original reference to Colombia may be the result of a labeling error, therefore, this species was also excluded from our checklist.

In addition to the existing geographic bias, we also noticed a taxonomic bias in our dataset. This pattern is primarily driven by two factors: (1) better taxonomic resources for some groups (*e.g.*, *Nannotrigona*, *Oxytrigona*), and (2) an emphasis on bee research in agroecology (Nates-Parra, 2016). These biases are translated into many studies focusing on charismatic groups of bees, such as orchid bees (Bonilla-Gomez & Nates-Parra, 1992; Ospina-Torres & Sandino-Franco, 1997; Ramírez, Dressler & Ospina, 2002; Ramírez, 2005; Parra-H, Ospina-Torres & Ramírez, 2006; Parra-H & Nates-Parra, 2007; Farfán, 2019; Londoño Carvajal, 2022), oil-collecting bees (Moure, 2002; Vélez & Vivallo, 2012; Vivallo, Vélez & Fernández, 2013; Vivallo, Vélez & Fernández, 2016; Vivallo & Vélez, 2016; Vivallo, 2021; Vivallo, 2022a; Vivallo, 2022b; Vivallo, 2024), stingless bees and bumble bees (Liévano, Ospina Torres & Nates-Parra, 1994), which also concentrate the highest species richness in the country (Fig. 3). Stingless bees, in particular, have received the most attention, as they are the most abundant bees in the Neotropical region (Ramalho, 2004; Cairns et al., 2005; Brosi et al., 2008; Sardi-Saavedra et al., 2024; Rodríguez-Rojas et al., 2024), are important pollinators in natural and agricultural areas (Carabalí-Banguero, Montoya-Lerma & Carabalí, 2021; Maldonado-Cepeda et al., 2024; Jaramillo, Reyes-Palencia & Jiménez, 2024), and have ancestral relationships with rural and Indigenous communities (Nates-Parra & Rosso-Londoño, 2013). Furthermore, the growing interest in meliponiculture as a sustainable economic activity has increased the need for precise taxonomic identification of these species (Dos Santos et al., 2022). This has motivated several taxonomic studies (Jaramillo, Ospina & Gonzalez, 2019; González & Roubik, 2008; Guevara, Gonzalez & Ospina, 2020; Lepeco, 2024; Gutiérrez-Rubiano, Ospina-Torres & Guevara, 2025) and the steady description of numerous new species (Gonzalez & Sepúlveda, 2007; Gonzalez, Rasmussen & Velasquez, 2010; Gonzalez & Griswold, 2011; Gonzalez & Griswold, 2012; Gonzalez & Engel, 2012; Nogueira et al., 2019; Nogueira, Ribeiro & De Oliveira, 2020; Nogueira, Rasmussen & Oliveira, 2021; Engel, 2022a; Engel, 2022b; Engel, 2022c; Engel, 2022d; De Oliveira et al., 2024), highlighting the high diversity of this ecologically and culturally important group of pollinators.

Bee groups that are more taxonomically challenging require proper revision. For example, genera such as *Augochlora* Smith, 1853, *Lasioglossum* (*Dialictus*) Robertson, 1902, and *Colletes* Latreille, 1802 have been revised in recent years in various countries in the Americas, revealing a large number of new species in the United States, Mexico, Chile, and Brazil (Gibbs, 2010; Balboa, Hinojosa-Díaz & Ayala, 2017; Ferrari, 2017; Ferrari, 2019; Lepeco & Gonçalves, 2020a; Lepeco & Gonçalves, 2020b; Lepeco & Gonçalves, 2023; Gardner & Gibbs, 2022; Landaverde-González et al., 2023). We also identified five genera that currently lack confirmed species for the country: *Ptilothrix* Smith, 1853, *Protandrena* (aff. *Pseudopanurgus*), *Hylaeus* Fabricius, 1793, *Temnosoma* Smith, and *Monoeca* Lepeletier & Serville, 1828. Although our understanding of Colombian bee diversity has grown significantly over the past decade (Cortés-Gómez, Rico-Aristizábal & Amarillo-Suarez, 2023) the absence of verifiable records for these genera reveals critical gaps in the country’s taxonomic knowledge. This study also underscores the need to develop formal training programs for bee taxonomists in Colombia, who can help fill these critical gaps of information.

Globally, there is growing concern over the decline in bee populations (Goulson et al., 2015). In continental regions such as North America and Europe, this concern has led to the recent publication of numerous checklists (Gibbs et al., 2017; Dibble et al., 2017; Owens et al., 2018; Decker et al., 2020; Kilpatrick et al., 2020; Portman et al., 2023; Ghisbain et al., 2023; Ghisbain et al., 2025; Bartholomew et al., 2024; De Pedro et al., 2024; Zarrillo, Stoner & Ascher, 2025; Wilson et al., 2025), which have collectively contributed to building a robust base knowledge of the bee fauna in these areas. Checklists serve as a starting point to document existing biodiversity in a geographic area, which can be used to regularly assess the arrival of new species or the local extinction of others (Zattara & Aizen, 2021). However, to accurately identify bee declines, these checklists must be complemented with monitoring programs that include community surveys and demographic population studies (Turley et al., 2022). As a mega diverse country, this updated checklist of the bee fauna of Colombia represents a critical first step toward establishing a solid foundation for future ecological, taxonomic, and conservation research. Insect collections provide a unique and valuable resource to harbor these specimens and help fill these knowledge gaps (Bartomeus et al., 2019). This study highlights the importance of regional insect collections for the study of bee biodiversity, which contributed to 64% of the records here reported. This network will be critical for future coordinated efforts related to bee biodiversity and monitoring in Colombia and across countries. Currently, there are 12 bee species categorized under threat in Colombia (Amat-García, Andrade & Amat-García, 2007). However, this classification is partially based on species rarity or the scarcity of records rather than documented population declines, as no long-term studies or monitoring programs currently exist, hindering the conservation of Colombian bees. It is also important to note that the absence of locally managed native bee species commercially available for pollination services increases the risk of potential introduction of non-native managed pollinators, such as *Bombus terrestris* Linnaeus, which have caused severe declines of native bees in other regions of South America (Morales et al., 2013).

## Conclusions

This study presents the first annotated checklist of the bees of any country in the western hemisphere. We documented 738 species in 123 genera across five families in Colombia and serves as a starting point to promote the development of updated inventories and systematic monitoring programs to gain a clearer understanding of the bee diversity in the country. This information is also critical to conduct more rigorous assessments of the actual conservation status of its species. Our results highlight the importance of promoting research on the biological and ecological aspects of these species, as most native bees in Colombia lack basic information regarding their life history traits and ecology. It is also essential to promote taxonomic work of species-rich genera (*e.g.*, *Ceratina*, *Colletes*, *Hylaeus*, *Lasioglossum*) that urgently need revision and have the potential to significantly increase the number of species recorded for the region.

##  Supplemental Information

10.7717/peerj.21684/supp-1 Supplemental Information 1 Taxonomic classification used in this checklist Summary of the taxonomic framework followed in this checklist, including family, subfamily, tribe, and genus assignments for all 738 bee species recorded in Colombia.

10.7717/peerj.21684/supp-2 Supplemental Information 2 Annotated checklist of the bees of Colombia We report 738 bee species (123 genera, five families) recorded for the country, compiled from 18 Colombian entomological collections, public dataset, and published literature. Species are organized by family, subfamily, and tribe in alphabetical order, and listed alphabetically within each tribe. For each species, we provide the author and year of description, type locality, distributional records by department and municipality, and the data source.

10.7717/peerj.21684/supp-3 Supplemental Information 3 References used for species identification and record compilation Bibliographic references used for the taxonomic identification of specimens deposited in the 18 entomological collections consulted, and for the compilation of occurrence records from the published literature.

10.7717/peerj.21684/supp-4 Supplemental Information 4 Database of bee records compiled from entomological collections, online databases, and published literature Occurrence database containing all 69,439 bee records included in the annotated checklist, formatted following Darwin Core standards. Definitions of each term are provided in the Metadata sheet.

10.7717/peerj.21684/supp-5 Supplemental Information 5 Species listed for Colombia whose records could not be confirmed in our annotated checklist List of 109 bee species previously reported for Colombia whose presence in the country could not be verified across the three data sources consulted (entomological collections, online databases, and published literature). For each species, we indicate the source reporting it for Colombia and its current known distribution in the Neotropics.

10.7717/peerj.21684/supp-6 Supplemental Information 6 Biological trait data for bees recorded in Colombia Trait assignments for the 738 species recorded in our checklist. For each species, the table includes family, subfamily, tribe, genus, species, broad sociality (solitary, eusocial, variable, or brood parasite), broad nesting strategy (above-ground, below-ground, variable, or brood parasite), the taxonomic level at which the trait was assigned (*e.g.*, species, genus), and the primary literature sources used for trait assignment. Brood-parasitic genera are classified as such for both sociality and nesting, as they do not construct their own nests.

10.7717/peerj.21684/supp-7 Supplemental Information 7 Genera excluded from the phylogenetic analysis List of bee genera recorded in Colombia that could not be included in the genus-level phylogenetic analysis because they are absent from the species-level phylogeny of Henríquez-Piskulich et al. (2024).

10.7717/peerj.21684/supp-8 Supplemental Information 8 Relationship between sampling effort and species richness across departments Linear regression analysis showing the relationship between the number of bee records and the number of bee species report across departments in Colombia. Both variables were square-root transformed.

10.7717/peerj.21684/supp-9 Supplemental Information 9 Relationship between country area and phylogenetic diversity Linear regression analysis evaluating the relationship between the country area (km^2^) and the phylogenetic diversity metrics calculate for Colombia, Chile, Brazil and USA.

10.7717/peerj.21684/supp-10 Supplemental Information 10 Relationship between country area and the number of reported species in the Americas Linear regression analysis evaluating the relationship between the country area (km^2^) and the number of bees species reported in the Americas. Both variables were log-transformed to reduce skewness and better fit a linear model.

## Additional Information and Declarations

### Data Availability

The following information was supplied regarding data availability: The data is available in the Supplemental Files. The code is available at Zenodo: – Martin-Rojas, R. D. (2026). The first annotated checklist of the bees of Colombia reveals an overlooked diversity and areas with major data deficiencies. [Computer software]. Zenodo. https://doi.org/10.5281/zenodo.21689539.
