# Supplementary material for: The first annotated checklist of the bees of Colombia reveals an overlooked diversity and areas with major data deficiencies
Source: PeerJ. 2026 Oct 1;14:e21684. doi: 10.7717/peerj.21684 (PMC13634308; doi:10.7717/peerj.21684)
Supplement: Supplementary file 1 [file peerj-14-21684-s008.pdf]

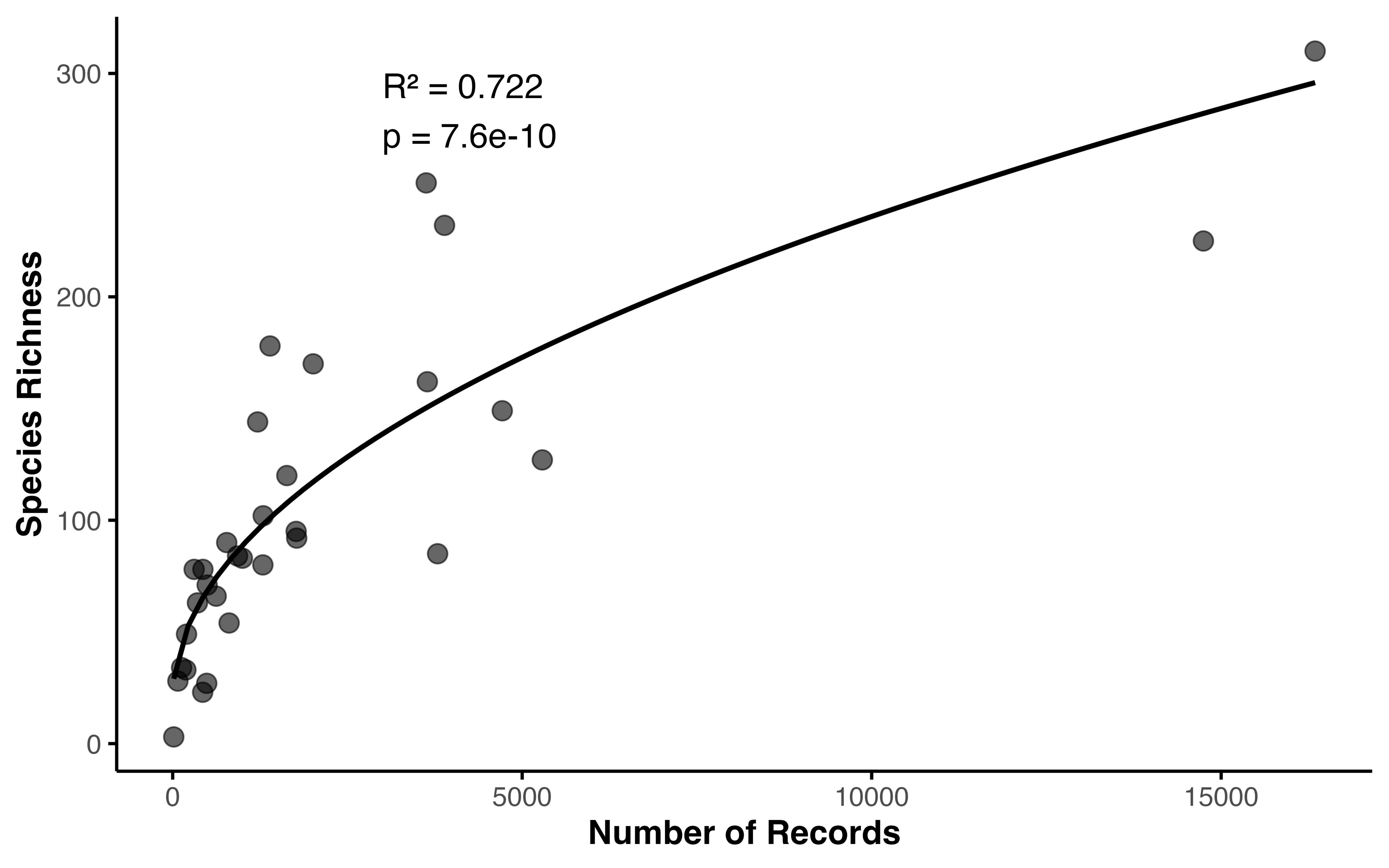

R² = 0.722
p = 7.6e-10
Species Richness
300
200
100
0
0
5000
10000
15000
Number of Records
