# Supplementary material for: The first annotated checklist of the bees of Colombia reveals an overlooked diversity and areas with major data deficiencies
Source: PeerJ. 2026 Oct 1;14:e21684. doi: 10.7717/peerj.21684 (PMC13634308; doi:10.7717/peerj.21684)
Supplement: Supplementary file 2 [file peerj-14-21684-s009.pdf]

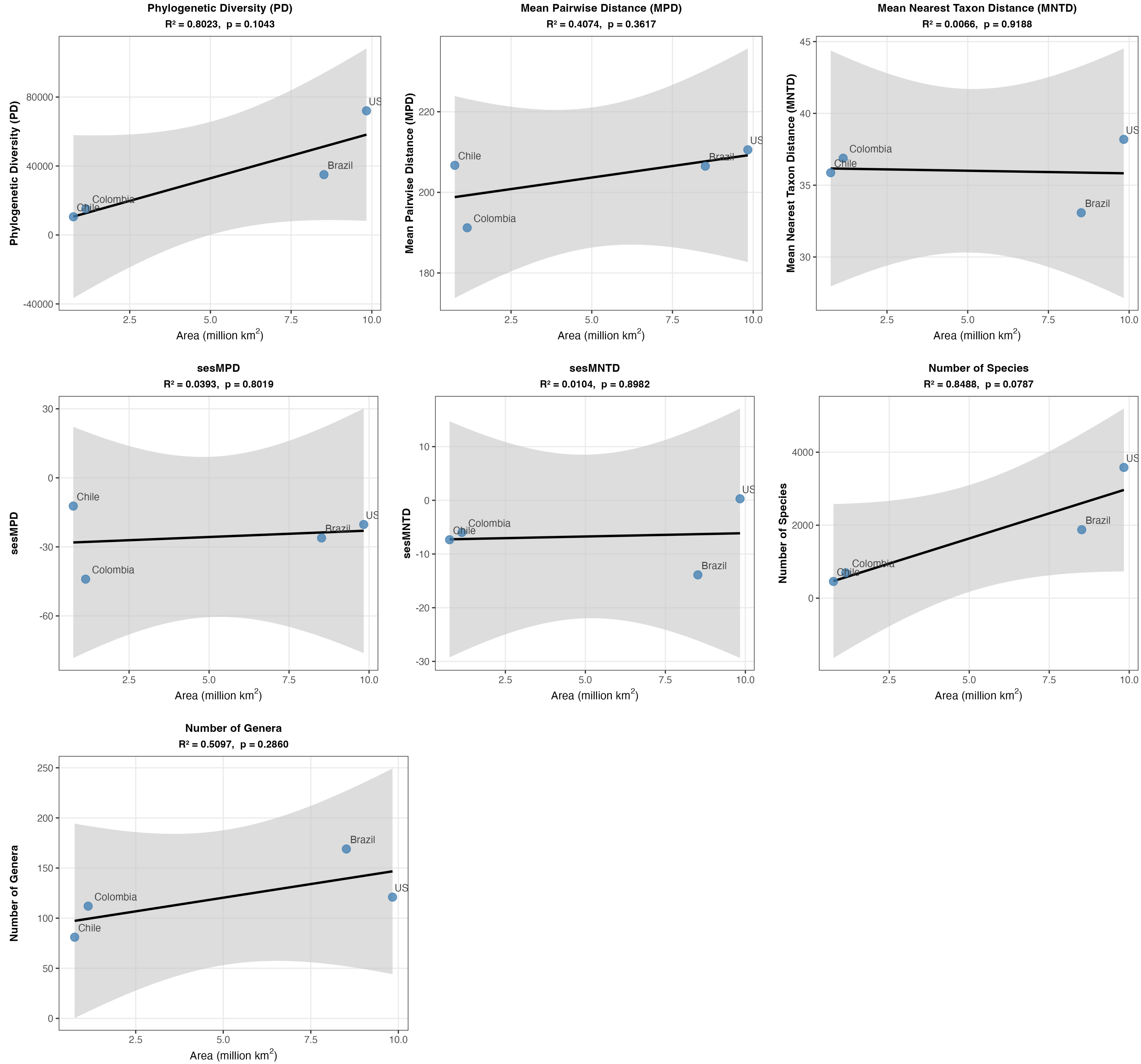

Phylogenetic Diversity (PD)
R² = 0.8023, p = 0.1043
Phylogenetic Diversity (PD)
80000
40000
0
-40000
US
Brazil
Colombia
Chile
2.5
5.0
7.5
10.0
Area (million km²)
Mean Pairwise Distance (MPD)
R² = 0.4074, p = 0.3617
Mean Pairwise Distance (MPD)
220
200
180
Chile
Colombia
Brazil
US
2.5
5.0
7.5
10.0
Area (million km²)
Mean Nearest Taxon Distance (MNTD)
R² = 0.0066, p = 0.9188
Mean Nearest Taxon Distance (MNTD)
45
40
35
30
Colombia
Chile
Brazil
US
2.5
5.0
7.5
10.0
Area (million km²)
sesMPD
R² = 0.0393, p = 0.8019
sesMPD
30
0
-30
-60
Chile
Colombia
Brazil
US
2.5
5.0
7.5
10.0
Area (million km²)
sesMNTD
R² = 0.0104, p = 0.8982
sesMNTD
10
0
-10
-20
-30
Colombia
Chile
Brazil
US
2.5
5.0
7.5
10.0
Area (million km²)
Number of Species
R² = 0.8488, p = 0.0787
Number of Species
4000
2000
0
US
Brazil
Colombia
Chile
2.5
5.0
7.5
10.0
Area (million km²)
Number of Genera
R² = 0.5097, p = 0.2860
Number of Genera
250
200
150
100
50
0
Brazil
US
Colombia
Chile
2.5
5.0
7.5
10.0
Area (million km²)
