# Supplementary material for: The first annotated checklist of the bees of Colombia reveals an overlooked diversity and areas with major data deficiencies
Source: PeerJ. 2026 Oct 1;14:e21684. doi: 10.7717/peerj.21684 (PMC13634308; doi:10.7717/peerj.21684)
Supplement: Supplementary file 3 [file peerj-14-21684-s010.pdf]

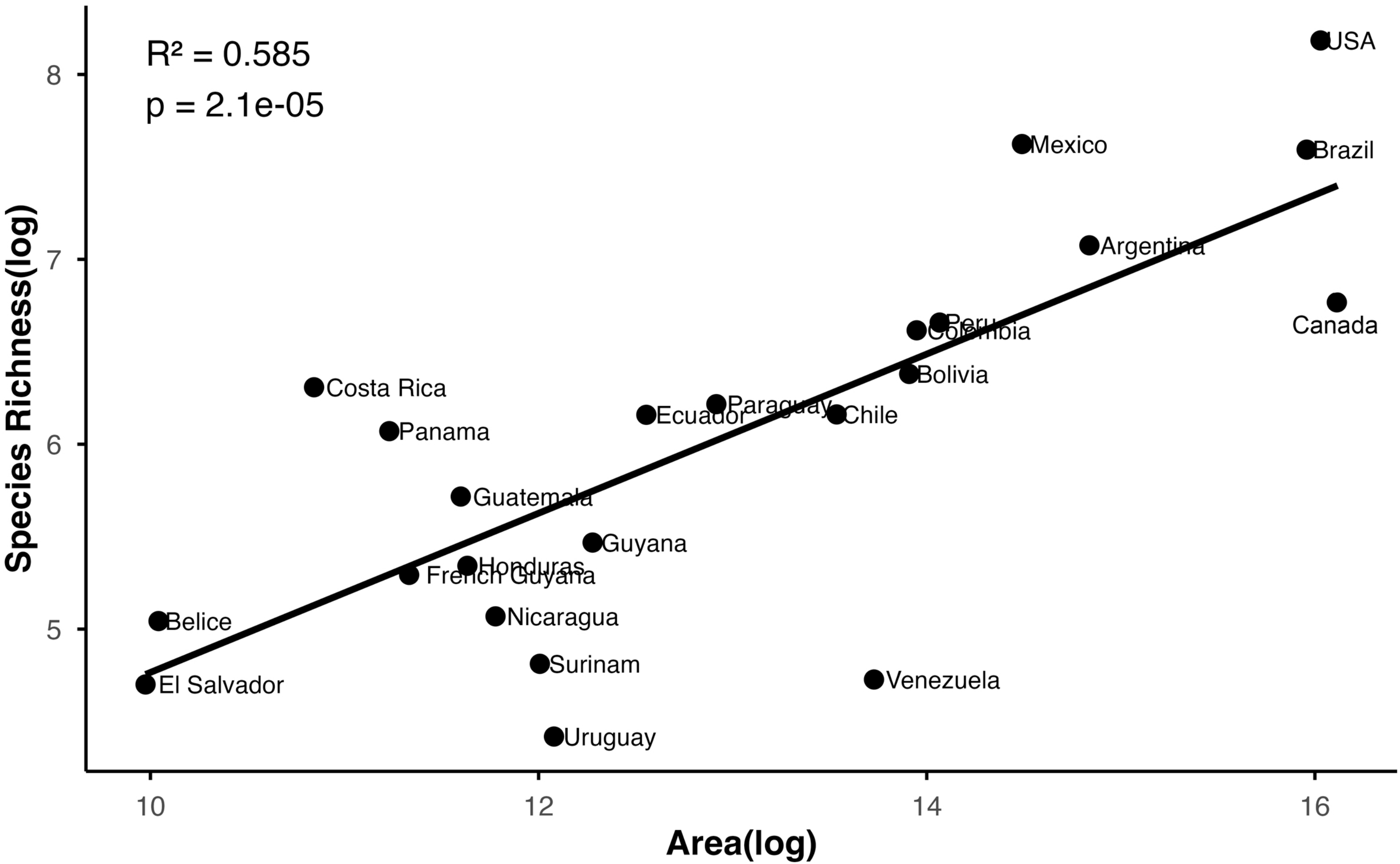
R² = 0.585
p = 2.1e-05
Species Richness(log)
Area(log)
8
7
6
5
10
12
14
16
USA
Mexico
Brazil
Argentina
Canada
Peru
Colombia
Bolivia
Costa Rica
Paraguay
Chile
Ecuador
Panama
Guatemala
Guyana
Honduras
French Guyana
Belice
Nicaragua
Surinam
Venezuela
El Salvador
Uruguay
