# Supplementary material for: The first annotated checklist of the bees of Colombia reveals an overlooked diversity and areas with major data deficiencies
Source: PeerJ. 2026 Oct 1;14:e21684. doi: 10.7717/peerj.21684 (PMC13634308; doi:10.7717/peerj.21684)
Supplement: Supplementary file 4 [file peerj-14-21684-s001.docx]

**S1 Appendix**. **Taxonomic classification used in this checklist**.

For taxonomic classification, we primarily followed Michener (2007), with several updates based on recent molecular phylogenetic studies. For the family Apidae, we followed the subfamily classification proposed by Bossert et al. (2019). Within Apidae, for the subfamily Antophorinae, we follow Orr et al. (2022). For the subfamily Nomadinae, we follow Sless et al. (2022). For the subfamily Eucerinae, we follow Freitas et al. (2021), with the update to the genera of the tribe Eucerini proposed by Freitas et al. (2023). For the tribe Tapidotaspidini, we follow Aguiar et al. (2020). For the family Andrenidae, we followed the revised classification by Bossert et al. (2022). For the Colletidae, we follow Almeida et al. (2012), for the genus Colletes, we follow Ferrari & Packer (2021), and for the subfamily Neopasiphaeinae, we follow Almeida et al. (2019) and Melo (2022). For the family Halictidae, we follow Danforth et al. (2008) and Gibbs et al.(2012) for the subfamily and tribe classification and for the genus in Sphecodini, we follow Gonçalves (2021). For the tribe Augochlorini we follow Gonçalves (2016) phylogeny, except for the *Neocorynura* clade, we follow Smith-Pardo & Gonzalez (2009).

1. **Andrenidae** Latreille

1.1. Oxaeinae Ashmead

1.2. Panurginae Leach

1.2.1. Calliopsini Robertson

1.2.2. Protandrenini Robertson

2. **Apidae** Latreille

2.1. Anthophorinae Dahlbom

2.2.  Apinae Latreille

2.2.1.  Apini Latreille

2.2.2.  Bombini Latreille

2.2.3.  Centridini Cockerell & Cockerell

2.2.4.  Euglossini Latreille

2.2.5.  Meliponini Lepeletier

2.3.  Eucerinae Latreille

2.3.1.  Ancyloscelidini Roig-Alsina & Michener

2.3.2.  Emphorini Robertson

2.3.3.  Eucerini Latreille

2.3.4.  Exomalopsini Vachal

2.3.5.  Tapinotaspidini Roig-Alsina & Michener

2.4.  Nomadinae Latreille

2.4.1.  Coelioxoidini Nates-Parra & Fernandez

2.4.2.  Epeolini Robertson

2.4.3.  Isepeolini Rozen, Eickwort & Eickwort

2.4.4.  Nomadini Latreille

2.4.5.  Osirini Handlirsch

2.4.6.  Protepeolini Linsley & Michene

2.4.7.  Rhathymini Lepeletier

2.5.  Xylocopinae Latreille

2.5.1.  Ceratinini Latreille

2.5.2.  Tetrapediini Michener & Moure

2.5.3.  Xylocopini Latreille

3.    **Colletidae** Lepeletier

3.1.  Colletinae Lepeletier

3.2.  Diphaglossinae Vachal

3.2.1.  Caupolicanini Michener

3.2.2.  Diphaglossini Vachal

3.2.3.  Dissoglottini Moure

3.3.  Hylaeinae Viereck

3.4.  Neopasiphaeinae Cockerell

3.4.1.  Eulonchopriini Moure

3.4.2.  Lonchopriini Moure

3.5.  Xeromelissinae Cockerell

4.    **Halictidae** Thomson

4.1.  Halictinae Thomson

4.1.1.  Augochlorini Beebe

4.1.2.  Caenohalictini Michener

4.1.3.  Halictini Thomson

4.1.4.  Sphecodini Schenck

5.    **Megachilidae** Latreille

5.1.  Megachilinae Latreille

5.1.1.  Anthidiini Ashmead

5.1.2.  Megachilini Latreille

5.1.3.  Osmiini Newman

**References**

Aguiar AJC, Melo GAR, Vasconcelos TNC, Gonçalves RB, Giugliano L, Martins AC. 2020. Biogeography and early diversification of Tapinotaspidini oil-bees support presence of Paleocene savannas in South America. *Molecular Phylogenetics and Evolution* 143:106692. DOI: 10.1016/j.ympev.2019.106692.

Almeida EAB, Packer L, Melo GAR, Danforth BN, Cardinal SC, Quinteiro FB, Pie MR. 2019. The diversification of neopasiphaeine bees during the Cenozoic (Hymenoptera: Colletidae). *Zoologica Scripta* 48:226–242. DOI: 10.1111/zsc.12333.

Almeida EAB, Pie MR, Brady SG, Danforth BN. 2012. Biogeography and diversification of colletid bees (Hymenoptera: Colletidae): Emerging patterns from the southern end of the world. *Journal of Biogeography* 39:526–544. DOI: 10.1111/j.1365-2699.2011.02624.x.

Bossert S, Murray EA, Almeida EAB, Brady SG, Blaimer BB, Danforth BN. 2019. Combining transcriptomes and ultraconserved elements to illuminate the phylogeny of Apidae. *Molecular Phylogenetics and Evolution* 130:121–131. DOI: 10.1016/j.ympev.2018.10.012.

Bossert S, Wood TJ, Patiny S, Michez D, Almeida EAB, Minckley RL, Packer L, Neff JL, Copeland RS, Straka J, Pauly A, Griswold T, Brady SG, Danforth BN, Murray EA. 2022. Phylogeny, biogeography and diversification of the mining bee family Andrenidae. *Systematic Entomology* 47:283–302. DOI: 10.1111/syen.12530.

Danforth BN, Eardley C, Packer L, Walker K, Pauly A, Randrianambinintsoa FJ. 2008. Phylogeny of Halictidae with an emphasis on endemic African Halictinae. *Apidologie* 39:86–101. DOI: 10.1051/apido:2008002.

Ferrari RR, Packer L. 2021. Morphological phylogeny and review of the generic classification of Colletinae (Hymenoptera: Colletidae). *Zoological Journal of the Linnean Society* 193:199–216. DOI: 10.1093/zoolinnean/zlaa128.

Freitas FV, Branstetter MG, Franceschini-Santos VH, Dorchin A, Wright KW, López-Uribe MM, Griswold T, Silveira FA, Almeida EAB. 2023. UCE phylogenomics, biogeography, and classification of long-horned bees (Hymenoptera: Apidae: Eucerini), with insights on using specimens with extremely degraded DNA. *Insect Systematics and Diversity* 7:3. DOI: 10.1093/isd/ixad012.

Freitas FV, Branstetter MG, Griswold T, Almeida EAB. 2021. Partitioned Gene-Tree Analyses and Gene-Based Topology Testing Help Resolve Incongruence in a Phylogenomic Study of Host-Specialist Bees (Apidae: Eucerinae). *Molecular Biology and Evolution* 38:1090–1100. DOI: 10.1093/molbev/msaa277.

Gibbs J, Brady SG, Kanda K, Danforth BN. 2012. Phylogeny of Halictine bees supports a shared origin of eusociality for *Halictus* and *Lasioglossum* (Apoidea: Anthophila: Halictidae). *Molecular Phylogenetics and Evolution* 65:926–939. DOI: 10.1016/j.ympev.2012.08.013.

Gonçalves RB. 2016. A molecular and morphological phylogeny of the extant Augochlorini (Hymenoptera, Apoidea) with comments on implications for biogeography. *Systematic Entomology* 41:430–440. DOI: 10.1111/syen.12166.

Gonçalves RB. 2021. A revised genus-level classification for the Neotropical groups of the cleptoparasitic bee tribe Sphecodini Schenck (Hymenoptera, Apidae, Halictinae). *Revista Brasileira de Entomologia* 65:ee20200094. DOI: 10.1590/1806-9665-rbent-2020-0094.

Melo GAR. 2022. A revised classification for the neopasiphaeine line, with the description of new species of the bee tribe Lonchopriini (Apidae, Colletinae). *Acta Biológica Paranaense* 51.

Michener CD. 2007. *The bees of the world*. The Johns Hopkins University Press.

Orr MC, Branstetter MG, Straka J, Yuan F, Leijs R, Zhang D, Zhou Q, Zhu C-D. 2022. Phylogenomic Interrogation Revives an Overlooked Hypothesis for the Early Evolution of the Bee Family Apidae (Hymenoptera: Apoidea), With a Focus on the Subfamily Anthophorinae. *Insect Systematics and Diversity* 6:7. DOI: 10.1093/isd/ixac022.

Sless TJL, Branstetter MG, Gillung JP, Krichilsky EA, Tobin KB, Straka J, Rozen JG, Freitas FV, Martins AC, Bossert S, Searle JB, Danforth BN. 2022. Phylogenetic relationships and the evolution of host preferences in the largest clade of brood parasitic bees (Apidae: Nomadinae). *Molecular Phylogenetics and Evolution* 166:107326. DOI: 10.1016/j.ympev.2021.107326.

Smith-Pardo A, Gonzalez VH. 2009. A revision of *Neocorynura* bees of the *joannisi* group with new geographical records for other Andean species (Hymenoptera: Halictidae, Augochlorini). *Studies on Neotropical Fauna and Environment* 44:115–129. DOI: 10.1080/01650520903101085.
