# Supplementary material for: The first annotated checklist of the bees of Colombia reveals an overlooked diversity and areas with major data deficiencies
Source: PeerJ. 2026 Oct 1;14:e21684. doi: 10.7717/peerj.21684 (PMC13634308; doi:10.7717/peerj.21684)
Supplement: Supplementary file 5 [file peerj-14-21684-s002.docx]

**Appendix S2. Annotated checklist of the bees of Colombia**

Here we present the first annotated checklist of the bees of Colombia. We report 738 bee species (123 genera, five families) recorded for the country, compiled from 18 Colombian entomological collections, public dataset, and published literature. Species are organized by family, subfamily, and tribe in alphabetical order, and listed alphabetically within each tribe. For each species, we provide the author and year of description, type locality, distributional records by department and municipality, and the data source.

Symbols follow: § =Species known only from the holotype, **†** =Type locality record, ***** =New country record for Colombia compared with Vélez-Ruiz (2009), **◊=** Introduced species. The super index of the in each municipality indicate the sources of the records: ^1^= LABUN, ^2^= MPUJ, ^3^= CTNI, ^4^= MEPB, ^5^= ICN, ^6^= CBUMAG, ^7^= UMNG-ins, ^8^= CETdeA, ^9^= CBUCES-F, ^10^= MENT-UT, ^11^= MUSENUV, ^12^= CEUNP, ^13^= CEUA, ^14^= MEFLG, ^15^= CIUQ, ^16^= MHN-UC, ^17^= IAvH, ^18^= ANDES,^19^= AMNH, ^20^= SEMC, ^21^= EMEC, ^22^= NHMUK, ^23^= DZUP, ^24^= FSCA, ^25^= MCZ, ^26^= MNHN, ^27^= USNM, ^28^= BeeBDC.

**Andrenidae Latreille, 1802**

**Oxaeinae Ashmead, 1899**

**Genus Oxaea Klug, 1807**

***Oxaea flavescens* Klug, 1807**

**Country records**: Casanare: Villanueva^1^; Meta: Mesetas^18^, Vistahermosa^18^.

**Panurginae**

**Calliopsini Robertson, 1922**

**Genus *Acamptopoeum* Cockerell, 1905**

***Acamptopoeum colombiense* Shinn, 1965**

**Country records**: Cesar: Valledupar^28^; Magdalena: Santa Marta†^6^.

***Acamptopoeum fernandezi* González, 2004**

**Country records**: Boyacá: Villa de Leyva†^1^

***Acamptopoeum prinii* (Holmberg, 1884)**

**Country records**: Casanare: Chámeza^1^, Villanueva^1^; Cundinamarca: Medina^1^.

**Protandrenini**

**Genus *Andinopanurgus* Gonzalez & Engel, 2011**

***Andinopanurgus bachue* (Gonzalez & Ruz, 2007)**

**Country records**: Boyacá: Tunja^5^, Ventaquemada^5^, Villa de Leyva†^5,28^; Cundinamarca: La Calera^20^.

***Andinopanurgus guarnensis* (Gonzalez & Ruz, 2007)**

**Country records**: Antioquia: Cocorná^14^, El Carmen de Viboral^14,28^, Envigado^14,28^, Guarne†^14,5,28^, La Unión^14^, Medellín^20^.

***Andinopanurgus rangeli* (Gonzalez & Ruz, 2007)**

**Country records**: Boyacá: Chiquiza^1^, Tunja^1,4,5^, Ventaquemada^28^, Villa de Leyva†^15^; Cundinamarca: Bogotá, D.C.^1,7,20^, Bojacá^7^, Cajicá^7^, Cota^1^, La Calera^1^, Nilo^5^, San Antonio del Tequendama^7^, Silvania^1^, Soacha^7^.

***Andinopanurgus wayruronga* (Gonzalez & Ruz, 2007)***

**Country records:** Cauca: Puracé ^20^

**Genus *Protandrena* Cockerell, 1896**

***Protandrena (cf. Pseudopanurgus*) Cockerell, 1897**

**Country records**: Santander: Onzaga^7^.

**Genus *Psaenythia* Gerstaecker, 1868**

***Psaenythia diceratops* Engel, Guevara, Ospina-Torres & Gonzalez, 2021***

**Country records**: Casanare: Chámeza† ^(Engel et al., 2021)^; Meta: Cumaral^1,(Engel et al., 2021)^.

**Apidae Latreille, 1802**

**Anthophorinae Dahlbom, 1835**

**Anthophorini Dahlbom, 1835**

**Genus Anthophora Latreille, 1803**

**Subgenus *Mystacanthophora* Brooks, 1988**

***Anthophora* *(Mystacanthophora)* *walteri* González & Chávez, 2004**

**Country records**: Boyacá: Chiquiza^1^, Sutamarchan^1^, Tunja^5^, Ventaquemada^18^, Villa de Leyva^1^; Cundinamarca: Bogotá, D.C.^1,3^, Bojacá^1^, Mosquera†^1,5^, San Antonio del Tequendama^7^, Soacha^1^.

**Apinae Latreille, 1802**

**Apinini Latreille, 1802**

**Genus *Apis* Linnaeus, 1758**

***Apis mellifera* Linnaeus 1758 ◊**

**Country records**: Amazonas: Puerto Ñarino^7^; Antioquia: Alejandría^13^, Amalfi^13^, Andes^13^, Angostura^1^, Anorí^13^, Bello^13^, Belmira^13^, Betania^13^, Caldas^13^, Carepa^13^, Caucasia^13^, Copacabana^13^, El Carmen de Viboral^1^, Entrerrios^13^, Envigado^1^, Fredonia^13^, Itagüí^13^, Ituango^13^, Jardín^1,13^, La Ceja^13^, La Unión^1^, Maceo^13^, Marinilla^13^, Medellín^13,6^, Rionegro^13^, San Francisco^18^, San Jerónimo^13^, San Pedro de los Milagros^13^, San Vicente^13^, Santa Fe de Antioquia^7^, Santa Rosa de Osos^13^, Sonsón^18^, Yarumal^13^, Yolombó^13^; Atlántico: Juan de Acosta^6^, Piojó^13^; Bolívar: , San Jacinto^18^, San Juán Nepomuceno^1^, Santa Cruz de Mompox^13^; Boyacá: Arcabuco^18^,Buenavista^1^, El Cocuy^18^, Maripí^1^, Miraflores^1^, Nuevo Colón^1^, Pajarito^18^, Ráquira^1^, San Miguel de Sema^1^, Ventaquemada^18^, Villa de Leyva^18^; Caldas: Aguadas^15^, Aranzazu^15^, Manizales^13^, Pácora^15^, Salamina^15^; Caquetá: Solano^18^; Casanare: Aguazul^7^, Chámeza^1^; Cauca: Miranda^11^; Cesar: Agustín Codazzi^18^ Valledupar^1,6,13,18,^; Chocó: Acandí^13,15^, Sipí^18^; Cundinamarca: Agua de Dios^7^, Anolaima^1^, Arbeláez^1^, Bogotá, D.C.^1,7^, Cajicá^7^, Choachí^1,7^, Cogua^7^, Cota^1^, Fusagasugá^1^, Guacheta^1^, Guasca^7^, La Calera^13,7^, Melgar^7^, Mosquera^1^, Pasca^7^, Puli^1^, San Antonio del Tequendama^7^, Silvania^15^, Soacha^7^, Sopó^7^, Subachoque^7^, Tabio^7^, Tenjo^7^, Tocancipá^7^, Villapinzón^7^, Zipaquirá^5^; Guaviare: San José del Guaviare^18^; Huila: Acevedo^1^, Colombia^1^, Rivera^1^, Teruel^1^, Villavieja^7^; La Guajira: Maicao^18,20^, Manaure^20^, Riohacha^13,20^, San Juan del Cesar^6, 18^; Magdalena: Aracataca^6,18^, Chivolo^6^, Ciénaga^6^, El Retén^6^, Santa Marta^1,6,18^, Zona Bananera^6^; Meta: Acacias^1^, La Macarena^18^, Puerto Gaitán^1;^ Puerto López^1^, Puerto Gaitán^1^,Villavicencio^1,11^; Nariño: Pasto^18^; Putumayo: Mocoa^1^; Quindío: Armenia^1,15^, Buenavista^15^, Cajamarca^15^, Calarcá^15^, Circasia^15^, Córdoba^15^, Filandia^13^, La Tebaida^15^, Pijao^15^, Quimbaya^13,15^, Salento^15^ Risaralda: Pereira^1,13,5^, Pueblo Rico^5^; Santander: Carcasi^13^, Cimitarra^1,13^, Encino^18^, Floridablanca^1^, Socorro^7^; Sucre: Coloso^1^, San Onofre^13^; Valle del Cauca: Caicedonia^15^, Cali^11, 20^, Jamundí^11^, Palmira^12^, Tuluá^12^, Yotoco^1^; Vaupés: Papunahua^1^, Taraira^18^, Yavaraté^18^; Vichada: Cumaribo^18^, Puerto Carreño^18^.

**Bombini Latreille, 1802**

**Genus *Bombus* Latrielle, 1802**

**Subgenus *Cullumanobombus* Vogt, 1911**

***Bombus* *(Cullumanobombus)* *funebris* Smith, 1854**

**Country records**: Antioquia: Bello^28^, Urrao^13,14^; Boyacá: Aquitania^1,18^, Arcabuco^18^, Chiquiza^1,7^, Duitama^5^, El Cocuy^1^, Garagoa^2^, Guicán^1^, Mongui^18^, Sogamoso^28^, Sotaquira^19^, Tunja^1^, Turmequé^28^, Tutaza^18^, Villa de Leyva^1,7^; Caldas: Manizales^17^, Salamina^15,28^, Villamaría^3,17,28^; Cauca: El Tambo^17^, Guapi^2^, Popayán^17^, Puracé^1,14,16,17,20^; Cundinamarca: Bogotá, D.C.^1,3,5,28^, Bojacá^1^, Choachí^1,7,19,20,28^, Chía^7^, Cogua^1^, Fómeque^1,18^, Guasca^1,2,3,28^, La Calera^1,18^, Lenguazaque^28^, Pacho^19^, Pasca^1^, San Cayetano^1^, Sesquilé^7^, Subachoque^1,3^, Tausa^1,5,7^, Ubaque^1,28^, Úbaté^7^, Villapinzón^1,3^, Zipaquirá^1^; Huila: San Agustín^1^; Magdalena: Aracataca^18^; Meta: San Juanito^28^, Nariño: Buesaco^1^, Chachagüí^1,3^, Consacá^28^, Ipiales^3^, Pasto^3,28^, Yacuanquer^3^; Risaralda: Pereira^28^; Santander: Cerrito^1^, Tona^28^; Tolima: Casabianca^28^, Herveo^28^,Murillo^1^, Santa Isabel^1^; Valle del Cauca: Santiago de Cali^11,17^.

***Bombus* *(Cullumanobombus)* *hortulanus* Friese, 1904**

**Country records**: Antioquia: Abejorral^28^, Andes^13^, Bello^13,28^, Belmira^13^, Ciudad Bolívar^13^, El Carmen de Viboral^13,28^, Envigado^1,3,13,14^, Guarne^4,13,28^, Jardín^28^, La Ceja^13,14^, La Estrella^3^, La Unión^6,13,14,28^, Medellín^1,4,5,6,13,28^, Retiro^13,28^, San José de la Montaña^13^, San Luís^2^, Santa Rosa de Osos^28^, Sonsón^13^, Yarumal^13^; Boyacá: Arcabuco^18,28^, Buenavista^1^, Chiquiza^1^, Chitaraque^1^, Duitama^19,28^, El Cocuy^18^, Gachantivá^1^, Garagoa^1,2^, Monguí^19^, Morro^19^, Ráquira^1^, San Miguel de Sema^1^, Santa María^19^, Sotaquirá^19^, Tinjacá^1^, Tunja^1^, Ventaquemada^1,18^, Villa de Leyva^18^; Caldas: Aguadas^15,28^, Aranzazu^15,28^, Manizales^13,4,28^, Manzanares^3^, Salamina^15,28^; Casanare: Orocué^19^, Yopal^18^; Cauca: Inzá^5^, Mercaderes^17^, Puracé^16^, Santa Rosa^16^, Sotará^16^, Tambo^16^; Cundinamarca: Anapoima^19^, Bogotá, D.C.^1,2,3,5,7,19,28^, Bojacá^1,5,14,28^, Cabrera^1^, Cajicá^7,28^, Choachí^1,19,3,7,28^, Chía^2,7^, Cogua^1,2,7^, Cota^1,28^, Fuquene^1^, Fusagasugá^1,2,3^, Fómeque^1,18,2^, Girardot^2^, Granada^1^, Guacheta^28^, Guasca^1,3,7,28^, La Calera^1,2,3,5,7,28^, La Mesa^19^, La Pradera^7^, La Vega^1^, Madrid^1^, Mosquera^1,28^, Nilo^5^, Pacho^1^, Paicol^⁷;^ Puerto Salgar^19^, San Antonio del Tequendama^1,2,7,19,28^, San Cayetano^20^, San Francisco^1,5,28^, San Francisco de Sales^19^, Sasaima^5^, Sesquile^1,7,28^, Sibaté^5^, Silvania^1^, Soacha^7^, Sopó^7^, Subachoque^1,14,5,7,28^, Suesca^1^, Sutatausa^1^, Tabio^7,28^, Tausa^1,28^, Tena^1^, Tenjo^1,14,2,7^, Tocancipa^7^, Ubaque^1^, Ubate^7^, Une^14^, Villa Pinzón^19^, Zipaquirá^1,14,7^; Huila: Acevedo^19^, Algeciras^1^, Hobo^1^, La Plata^1^, Neiva^1^, San Agustín^1,5^; Magdalena: Aracataca^18^, Santa Ana^5^, Santa Marta^1,17,5,6,28^; Meta: Restrepo^5^, San Juanito^28^, San Martín^18^, Villavicencio^5^; Nariño: Buesaco^1^, La Cruz^11^, Mallama^8^, Pasto^3,28^; Norte de Santander: Cucutilla^13,4,28^; Quindío: Calarcá^15^, Salento^15,18,28^; Risaralda: Apia^1^, Murillo^1^, Pereira^1^, Santa Rosa de Cabal^1^, Santuario^1^; Santander: Barrancabermeja^18^, Carcasi^13^, Floridablanca^1^, Piedecuesta^13,28^, Santa Barbara^17^; Tolima: Rovira^15^, San Sebastián de Mariquita^2^; Valle del Cauca: Buenaventura^16^, Dagua^16^, Rio Frío^1^, Santiago de Cali^12,16,28^, Trujillo^28^

***Bombus* *(Cullumanobombus)* *melaleucus* Handlirsch, 1888**

**Country records**: Antioquia: Amalfi^14^, Anorí^13^, Bello^14^, Envigado^14^, Guarne^1^, Jardín^14^, Medellín^14^, Rionegro^14^; Boyacá: Tinjacá^1^; Caldas: Aguadas^15,28^, Pácora^15,28^; Cauca: Balboa^11^; Cundinamarca: Anapoima^1^, Arbeláez^1^, Bogotá, D.C.^28^, San Antonio del Tequendama^7^, Tena^1^, Villeta^1^; Huila: Algeciras^1^, Campoalegre^1^, Hobo^1^; Magdalena: Ciénaga^28^; Meta: Castilla La Nueva^1^; Quindío: Armenia^15^, Filandia^2^; Risaralda: Pereira^1^; Santander: El Carmen de Chucurí^13^, Encino^1^, Floridablanca^1,28^, Guaca^28^, Piedecuesta^13^, Socorro^7^; Sucre: Coveñas^14^; Valle del Cauca: La Cumbre^28^.

***Bombus* *(Cullumanobombus)* *robustus* Smith, 1854**

**Country records**: Antioquia: Belmira^13^, Ciudad Bolívar^28^, El Carmen de Viboral^13^, Entrerríos^13^, Envigado^1,14^, Medellín^20^, San Francisco^13^, San José de la Montaña^13^; Boyacá: Aquitania^1^, Arcabuco^1^, Chiquiza^1^, Duitama^5,28^, Mongua^28^, Monguí^17^, Morro^18^, Sotaquirá^1^, Tibaná^1^, Ventaquemada^17^, Villa de Leyva^1,28^; Caldas: Aguadas^15,28^, Aranzazu^15,28^, Salamina^15,28^; Cauca: Belalcázar^5^, Caloto^12^, Inzá^1,5^, Páez^1^, Patía^1^, Piendamó^1^,Poliandra^¹¹^,  Puracé^1^, Santander de Quilichao^16^, Silvia^12^; Cundinamarca: Beltrán^1^, Bogotá, D.C.^1,2,3,5,28,20^, Carmen de Carupa^20^, Chipaque^5^, Choachí^1,18,3,5,28^, Chía^1,7^, Cogua^1^, Cota^1^, El Colegio^5^, Facatativá^28^, Fómeque^1,17,2^, Gachalá^1^, Guachetá^1^, Guasca^1,3,7,28^, Guatavita^1,5^, Junín^1^, La Calera^1,2,3,7^, La Vega^1^, Machetá^3^, Mosquera^1^, Pacho^1^, San Antonio del Tequendama^1^, Sesquilé^1^, Silvania^5^, Subachoque^1^, Suesca^1^, Tausa^1,7^, Tena^1^, Tenjo^18^, Tocaima^5^, Ubaque^1,28^, Une^14^, Villeta^3^, Zipaquirá^1,14,3,20^; Huila: San Agustín^1^; Meta: Villavicencio^3,5^; Nariño: Pasto^1,12,17,3^, Yacuanquer^3^; Norte de Santander: Pamplona^5^; Quindío: Calarcá^1^, Salento^15^; Risaralda: Santuario^1^; Santander: Charalá^1^, Piedecuesta^13^; Tolima: Chaparral^3^, Murillo^1^; Valle del Cauca: Buenaventura^1^, Dagua^12^, La Cumbre^12^, Palmira^12^, Pradera^12^, Río Frío^1^, Cali^12,16,20^.

***Bombus* *(Cullumanobombus)* *rubicundus* Smith, 1854**

**Country records**: Antioquia: Belmira^13,8^, Betania^28^, Caicedo^28^, Entrerríos^13^, La Ceja^3^, Liborina^13^, San José de la Montaña^13,28^, Urrao^9^; Boyacá: Arcabuco^1,17,28^, Chiquiza^1,17,7,28^, Cómbita^1,28^, Duitama^5,28^, El Cocuy^17^, Mongua^28^, Paipa^28^, Pajarito^5^, Ráquira^1^, Samacá^1^, San Miguel de Sema^1^, Santa María^18^, Sogamoso^28^, Tunja^1^, Ventaquemada^17^, Villa de Leyva^1,7,28^; Caldas: Aranzazu^15,28^, Neira^15,28^, Pensilvania^15^, Salamina^15,28^; Cauca: Cajibío^1^, Popayán^17^, Puracé^1,11,16,^ Cundinamarca: Bogotá, D.C.^1,17,18,2,3,5,7,28,20^, Cabrera^17^, Cajicá^7^, Chipaque^1^, Choachí^1,18,5,7,28,20^, Chocontá^3,20^, Chía^7^, Cogua^1,7^, El Rosal^7^, Fusagasugá^5^, Fómeque^1,17,2,28^, Granada^1^, Guasca^1,3,7,28^, Guatavita^28^, Guayabetal^28^, Junín^28^, La Calera^1,17,2,7,28^, Lenguazaque^28^, Machetá^5^, Medina^1^, Pacho^1^,, Pasca^28^, San Cayetano^7,28^, Sesquilé^7^, Sopó^28^, Subachoque^1,3,28^, Tausa^1,7,28^, Tenjo^1^, Ubaque^1,28^, Villapinzón^1,28^, Zipaquirá^1,3,7,20^; Huila: San Agustín^1,11,28^; Meta: San Juanito^28^; Nariño: Consacá^1^, Pasto^17,3,28^; Norte de Santander: Cucutilla^4,28^, Pamplona^28^; Quindío: Calarcá^15,28^, Salento^13,15,28^; Risaralda: Pereira^9^, Santuario^1^; Santander: Cerrito^1^, Charalá^28^, Concepción^28^, Encino^28^, Piedecuesta^28^, Santa Bárbara^17,28^, Tona^28^; Tolima: Herveo^15^, Murillo^1,15^; Valle del Cauca: Cali^16^.

**Subgenus *Thoracobombus* Dalla Torre, 1880**

***Bombus* *(Thoracobombus)* *excellens* Smith, 1879**

**Country records**: Antioquia: Angostura^1^, Bello^14^, Betania^13,14^, Ciudad Bolívar^13^, Cocorná^13^, Envigado^14^, Jardín^13^, La Ceja^14^, La Estrella^14^, Medellín^14,4,28^, Retiro^28^, Rionegro^14^; Bolívar: El Carmen de Bolívar^6,28^; Boyacá: Garagoa^2^, Motavita^7^, Pajarito^1^; Caldas: Aguadas^15,28^; Casanare: Chámeza^1^; Cauca: Popayán^11,28^, Santa Rosa^11^; Cundinamarca: Gachetá^1^, Guayabetal^28^, San Antonio del Tequendama^7^, Tena^1^,Villavieja^7^ ; Huila: Acevedo^18^, Hobo^1^, San Agustín^1^; Magdalena: Aracataca^1^, Santa Marta^1^; Norte de Santander: Cucutilla^13,4^; Putumayo: Santiago^20^; Santander: Charalá^1^, Floridablanca^1^, Piedecuesta^13^; Valle del Cauca: El Cerrito^28^, Palmira^12^, Santiago de Cali^16^.

***Bombus* *(Thoracobombus)* *pauloensis* Friese, 1913**

**Country records**: Antioquia: Alejandría^13^, Amalfi^13,28^, Bello^13^, Caldas^13^, Campamento^13^, Caucasia^13,4^, El Carmen de Viboral^1,13^, Envigado^1,13^, Fredonia^13^, Granada^13^, Guarne^1,13^, Gómez Plata^13^, Jardín^13,28^, La Ceja^3^, Maceo^13^, Medellín^13,3,6,28,20^, Puerto Berrío^13^, Retiro^1,28^, Rionegro^13,3^, Sabaneta^13^, San Francisco^13^, San José de la Montaña^3^, San Roque^13^, San Vicente^13^, Santa Fé de Antioquia^28^, Santo Domingo^13^, Santuario^13^, Sonsón^13^, Venecia^13^, Yolombó^13,28^; Boyacá: Aquitania^1^, Arcabuco^1,28^, Belén^28^, Boavita^1^, Buenavista^1^, Cerinza^28^, Chiquiza^28^, Combita^28^, Cucaita^28^, Duitama^18,3,28^, El Cocuy^1^, Floresta^28^, Gachantivá^1,7,28^, Macanal^1^, Miraflores^1^, Monguí^17^, Moniquirá^5^, Morro^18^, Motavita^7^, Nuevo Colón^1^, Paipa^5,28^, Puerto Boyacá^5^, Saboyá^1^, Samacá^28^, San Miguel de Sema^1^, Santa Rosa de Viterbo^5,28^, Sotaquirá^1,18^, Tibasosa^28^, Tinjacá^1^, Toca^28^, Tunja^17,28^, Turmequé^28^, Tuta^28^, Úmbita^28^, Ventaquemada^1,5,28^, Villa de Leyva^1,28^; Caldas: Aranzazu^15^, Manizales^28^, Pácora^15^, Salamina^15^; Casanare: Chámeza^1^, Yopal^28^; Cauca: Balboa^11^, Caldono^12^, Inzá^1,5^, Popayán^3^; Siberia^¹²^;Siberia^¹²,^ Cundinamarca: Albán^28^, Anolaima^1,3,28^, Arbeláez^1,5^, Arcabuco^18^, Beltrán^7^, Bogotá, D.C.^1,2,3,5,7,28^, Bojacá^5,28^, Cabrera^1^, Cajicá^1,7,28^, Caparrapí^5^, Cáqueza^1^, Carmen de Carupa^20^, Chaguaní^5^, Chipaque^3^, Choachí^1,7^, Chocontá^3,5,7,28,18^, Chía^1,2,28^, Cogua^1,2,7^, Cota^1,3,7,28^, El Colegio^5^, El Peñon^3^, El Rosal^1,28^, Facatativá^1,18,3,28^, Funza^3,28^, Fúquene^1,2^, Fusagasugá^1,28^, Gachalá^1^, Girardot^1,5^, Granada^1,28^, Guachetá^1^, Guasca^1,18,7,28^, Guatavita^12,5,28^, Guayabal de Síquima^28^, Guayabetal^3,5^, Junín^1^, La Calera^1,18,5,7,28^, La Mesa^1^, La Vega^1^, Machetá^1^, Madrid^1^, Medina^1,28^, Mosquera^1,3,5,28^, Nariño^28^, Nemocón^1^, Nilo^1,5^, Nocaima^28^, Pacho^18,3,28^, Quetame^5^, San Antonio del Tequendama^2,7^, San Cayetano^28^, San Francisco^1,28^, Sasaima^5^, Sesquilé^1,7,28^, Silvania^1,3^, Soacha^1,7^, Sopó^1,18,2,7^, Subachoque^1,18,5,7,28^, Suesca^1,5,28^, Tabio^7,28^, Tausa^7^, Tena^1,2^, Tenjo^1,18,2,7,28^, Tibirita^1^, Tocancipá^7,28^, Ubalá^1^, Ubaté^7^, Villapinzón^1,7,28,18^, Villeta^1,3,5^, Zipaquirá^1,7,28,20^, Útica^1^; Córdoba: Montelíbano^13^; Huila: Algeciras^1^, Guadalupe^1^, La Argentina^1^, La Plata^1^, Neiva^5, 28^, Palestina^1^, Pitalito^1,11^, San Agustín^1,5^, Villavieja^7^; Magdalena: Santa Marta^18^; Meta: Chirajara^18^, Puerto López^5^, Restrepo^5^, San Martín^18,5^, Villavicencio^1,3,5^, Vistahermosa^5^; Nariño: Buesaco^28^, Chachagüí^3^, Colón^28^, Cumbal^3^, Los Andes^1^, Pasto^3^, Sapuyes^28^, Yacuanquer^3^; Norte de Santander: Cúcuta^28^; Putumayo: Mocoa^5^, Sibundoy^1^; Quindío: Calarcá^15^, Pradera^15;^ Salento^1,3^; Risaralda: Pereira^1,15,2^, Santuario^1^; Santander: Charalá^1^, Cimitarra^5^, Floridablanca^28^, Santa Bárbara^13^; Tolima: Alvarado^3^, Armero^5^, Ataco^1^, Cajamarca^3^, Chaparral^5^, El Espinal^3^, Honda^5^, Ibagué^1^, Icononzo^1,18^, Melgar^18,2,5,28^, Nilo^2^; Valle del Cauca: Buga^16^, Bugalagrande^16^, Candelaria^12^, Dagua^12,16^, Palmira^12^,, Roldanillo^12^, Santiago de Cali^16,28^, Sevilla^1^, Yotoco^12^.

***Bombus* *(Thoracobombus)* *pullatus* Franklin, 1913**

**Country records**: Antioquia: Amalfi^20^, Guarne^28^, Medellín^28,20^, Retiro^28^, Rionegro^17^, Sonsón^17^; Atlántico: Barranquilla^5^; Boyacá: Chinavita^5^, Duitama^3^, Muzo^3,28^, Otanche^5^, Pauna^5^, Puerto Boyacá^1^, San Luis de Gaceno^1^, Yacopí^1^; Caldas: La Dorada^1^, Salamina^28^, Victoria^3^, Villamaría^3^; Casanare: Sabanalarga^5^, Yopal^17^; Cesar: Pueblo Bello^28^; Chocó: Medio San Juan^3^, Riosucio^5^; Cundinamarca: Agua de Dios^7^, Apulo^1,5^, Arbeláez^1^,Bogotá, D.C.^1^ Cachipay^3,5^, Caparrapí^5^, Chaguaní^5^, El Colegio^1^, Fusagasugá^1^, Guaduas^1,3,5^, Guayabetal^5^, La Mesa^1,3,5^, La Palma^1,5^, La Vega^1^, Medina^15^, Mosquera^1,5^, Nilo^1^, Nimaima^1^, Pacho^1,3^, Paime^1^, Paratebueno^1^,Puerto Salgar^1^, Ricaurte^1^, San Francisco^1^, Silvania^1^, Soacha^1^, Tena^1^, Villeta^1,3,5^, Viotá^5^, Zipaquirá^1^, Útica^1^; Córdoba: Tierralta^1^; Huila: Baraya^1^, Colombia^1^, Garzón^3^, La Argentina^1^, Neiva^1,17,28^, Palermo^1^, Rivera^1^, Villavieja^7^; Magdalena: Aracataca^5,6^, Barrancas^1^, Ciénaga^1,6^, Santa Marta^5^; Meta: Acacías^1,5^, Cumaral^1^, El Castillo^1^, La Macarena^1,5^, Mesetas^1^, Puerto Gaitán^1^, Puerto López^1,3,5^, Puerto Rico^1^, Restrepo^1^, San Juan de Arama^1^, San Vicente del Caguán^1^, Villavicencio^1,3,5^, Vistahermosa^1^; Norte de Santander: Bochalema^1^; Risaralda: Pereira^2,5,20^; Santander: Bucaramanga^3,5^, Cimitarra^1,28^,Suaita^1^; Tolima: Ataco^1^, Cunday^1,2^, El Espinal^3^,Honda^3,5^, Ibagué^3,5^, Melgar^5^, Rovira^3^, San Sebastián de Mariquita^1^; Valle del Cauca: Cartago^5^.

***Bombus* *(Thoracobombus)* *transversalis* (Olivier, 1789)**

**Country records**: Amazonas: La Pedrera^1^, Leticia^1,2^, Puerto Nariño^1^, Puerto Santander^1^; Boyacá: Santa María^1^, Santa Rosa de Viterbo^5^; Caquetá: Florencia^1^, San Vicente del Caguán^1^, Solano^1^, Valparaíso^11^; Casanare: Chámeza^1^; Cauca: Piamonte^11^,; Cundinamarca: Medina^1^; Guainía: Inírida^11^; Guaviare: Calamar^1^, San José del Guaviare^5^; Meta: Acacías^1^, Barranca de Upía^1^, La Macarena^1,28^, Puerto López^3^, Puerto Rico^1^, Restrepo^1,3,28^, San Juan de Arama^1^, San Vicente del Caguán^1^, Villavicencio^1,3^; Putumayo: Mocoa^1^, Villagarzón^5^; Vaupés: Mitú^5,28^; Vichada: Cumaribo^5^.

**Centridini Cockerell & Cockerell, 1901**

**Genus *Centris* Fabricius, 1804**

**Subgenus *Aphemisia* Ayala, 2002**

***Centris* *(Aphemisia)* *celadonia* Vivallo, Vélez & Fernández, 2016***

**Country records**: Huila: Villavieja†^7^.

***Centris (Aphemisia)* *lilacina* Cockerell, 1919***

**Country records**: Caquetá: Solano^17^.

***Centris* *(Aphemisia) magdalenica* Vivallo, 2022*§**

**Country records**: Magdalena: Santa Marta†^(Vivallo, 2022b)^.

***Centris* *(Aphemisia)* *mocsaryi* Friese, 1899**

**Country records**: Meta: La Macarena^(Vivallo, 2022b)^.

***Centris* *(Aphemisia)* *plumipes* Smith, 1854**

**Country records**: Amazonas: Mirití-Paraná^1^, Puerto Santander^1^; Vaupés: Mitú^1^.

***Centris* *(Aphemisia) quadrimaculata* Packard, 1869**

**Country records**: Amazonas: Leticia^17^; Antioquia: Amalfi^28^, Andes^14^, Apartadó^14^, Bello^13,28^, Medellín^13,14,28^, Sabaneta^14^, San Pedro de los Milagros^14^, Yolombó^28^; Caquetá: Morelia^1^; Cundinamarca: El Colegio^1^, La Mesa^1^; Guaviare: San José del Guaviare^17^; Huila: Gigante^1^; Meta: Villavicencio^14^; Valle del Cauca: Palmira^1^

***Centris* *(Aphemisia)* *vallecaucensis* Vivallo, Vélez & Fernández, 2016**

**Country records**: Valle del Cauca: El Dovio†^1^

**Subgenus *Centris* Fabricius, 1804**

***Centris (Centris) adani* Cockerell, 1949**

***Country records***: Antioquia: Itagüí^14^, Medellín^14^, Pintada^14^, Santa Fe de Antioquia^14^; Cauca: Popayán^11^; Huila: Villavieja^7^; Meta: Villavicencio^14^; Valle del Cauca: Buga^14^.

***Centris (Centris) aenea* Lepeletier, 1841**

**Country records**: Casanare: Hato Corozal^1^

***Centris* *(Centris)* *decolorata* Lepeletier, 1841***

**Country records**: Antioquia: Medellín^14^, Santa Fe de Antioquia^14^; Caldas: Pácora^15^; Magdalena: Santa Marta^17^; San Andrés y Providencia: Providencia^1^; Sucre: San Onofre^13^.

***Centris* *(Centris)* *dichroma* Vivallo, 2024***

**Country records**: Casanare: Nunchía† ^(Vivallo, 2024)^

***Centris* *(Centris)* *flavifrons* (Fabricius, 1775)**

**Country records**: Antioquia: Amalfi^28^, Envigado^14^, Gómez Plata^14^, Santa Fé de Antioquia^14^, Yolombó^28^; Caldas: Chinchiná^3^; Cauca: Patía^¹¹^; Popayán^3^; Guainía: Inírida^1,28^; Huila: Palermo^1^, Rivera^1^, Uribe^1^, Villavieja^7,28^; Meta: La Macarena^1^, Puerto López^2^, Puerto Rico^1^, San Juan de Arama^5^, Vistahermosa^1^; Sucre: San Onofre^13^; Tolima: Carmen de Apicalá^1^, Cunday^1,28^, El Espinal^14^, Melgar^1,14^; Valle del Cauca: Buenaventura^15^, Candelaria^17^, Dagua^20^, Palmira^3^, Cali^1,19,28^; Vaupés: Mitú^1^.

***Centris* *(Centris)* *leprieurii* (Spinola, 1841) ***

**Country records**: Cundinamarca^(Vivallo, 2024) (Buysson, 1901)^; San Andrés and Providencia: San Andrés^(Vivallo, 2024)^.

**Note:** Vivallo (Vivallo, 2024) reports this species from Bogotá following the record of Buysson (Buysson, 1901) for Centris decolorata, wich only includes the information: “Bogota (1862)” is provided. However, since no collector is indicated and considering the date of collection, the specimen may actually originate from another locality within the department of Cundinamarca rather than from the city of Bogotá.

***Centris* *(Centris)* *maculifrons* Smith, 1854***

**Country records**: Cauca: Guapí^(Vivallo, 2024)^.

***Centris* *(Centris)* *nitens* Lepeletier, 1841***

**Country records**: Meta: Vistahermosa^18^; Santander: Cimitarra^1^; Vichada: Cumaribo^1^.

***Centris* *(Centris)* *niveofasciata* Friese, 1899**

**Country records**: Bolívar: El Carmen de Bolívar^17^; Cesar: Valledupar^14^; Magdalena: Santa Marta^1^; Sucre: San Onofre^13^.

***Centris* *(Centris)* *obscurior* Michener, 1954**

**Country records**: Antioquia: Yolombó^28^, Amalfi^28^

***Centris* *(Centris)* cf. *spilopoda***

**Country records**: Casanare: Villanueva^1^.

***Centris* *(Centris)* *varia* (Erichson, 1848)**

**Country records**: Amazonas: Mirití-Paraná^1^, Puerto Santander^1^; Antioquia: Alejandría^14^, Amalfi^28^, Envigado^14^, San Luís^2^, Santa Fe de Antioquia^14^, Yolombó^28^, Zaragoza^28^; Atlántico: Juan de Acosta^6,28^; Bolívar: Zambrano^17^; Casanare: Hato Corozal^1^; Cauca: Corinto^28^; Cesar: Agustín Codazzi^19^; Guainía: San Felipe^1^; Huila: Palermo^1^, Tello^1^, Villavieja^7,28^; Magdalena: Nueva Granada^28^, Santa Marta^6,28^; Meta: Cumaral^1^, Puerto López^2^, Puerto Rico^1^, Restrepo^19,28^, San Juan de Arama^1^, Villavicencio^1^; Norte de Santander: Los Patios^1^; Santander: Cimitarra^1^; Sucre: San Onofre^13^; Tolima: Venadillo^1^; Vaupés: Mitú^1^

**Subgenus *Hemisiella* Moure, 1945**

***Centris* *(Hemisiella)* *dichrootricha* (Moure, 1945)**

**Country records**: Amazonas: La Pedrera^1^, Leticia^1,17,2,28^, Mirití-Paraná^1^; Bolívar: San Juan Nepomuceno^1^; Guaviare: San José del Guaviare^17^; Meta: Cumaral^2^, Mesetas^1^, Puerto Rico^1^.

***Centris* *(Hemisiella)* *facialis* Mocsáry, 1899**

**Country records**: Cauca: Popayán^11,19,28^; Cesar: Agustín Codazzi^19^; Huila: Villavieja^7^; Magdalena: Santa Marta^19^; Valle del Cauca: Cali^28^.

***Centris* *(Hemisiella)* *geminata* Cockerell, 1914***

**Country records**: Antioquia: Bello^3^, Copacabana^14^, Envigado^13,14^, Girardota^14^, Itagüí^14^, Jericó^14^, Medellín^13,14^, Santa Fe de Antioquia^14^; Boyacá: Miraflores^1^, Soatá^1^; Caquetá: Solano^5^; Cesar: Valledupar^14,6,28^; Cundinamarca: Arbeláez^1^, Fusagasugá^1^, Paime^1^, San Antonio del Tequendama^1^; Magdalena: Aracataca^6^, El Retén^6^, Santa Marta^1,14^; Meta: Villavicencio^14^; Quindío: Quimbaya^6,28^; Santander: Cimitarra^13^; Sucre: Colosó^1^; Tolima: Armero^14^, San Sebastián de Mariquita^1^; Valle del Cauca: Palmira^3^.

***Centris* *(Hemisiella) nebulosa* Vivallo & Vélez, 2016***

**Country records**: Bolívar: Zambrano†(Vivallo & Vélez, 2016).

***Centris* *(Hemisiella)* *nigrescens* Lepeletier, 1841***

**Country records**: Amazonas: Puerto Santander^1^; Antioquia: Santa Fe de Antioquia^14^; Bolívar: San Juan Nepomuceno^1^; Casanare: Hato Corozal^1^; Cesar: Valledupar^14,6^; Magdalena: Aracataca^6^; Santander: Cimitarra^1^; Sucre: Colosó^1^, Tolú^14^.

***Centris* *(Hemisiella)* *nitida* Smith, 1874**

**Country records**: Antioquia: Bello^28^, Medellín^14,28^; Bolívar: Cartagena^19^; Cundinamarca: Anolaima^1^; Magdalena: Santa Marta^28^, Zona Bananera^6,28^; Santander: Cimitarra^1^; Valle del Cauca: Cali^28^.

***Centris* *(Hemisiella)* *tarsata* Smith, 1874**

**Country records**: Bolívar: San Juan Nepomuceno^1^; Casanare: Orocué^1^; Cesar: Valledupar^1,14,17,28^; Cundinamarca: Arbeláez^1^; Magdalena: El Retén^6^, Santa Marta^1,6^, Zona Bananera^3^; Meta: San Juan de Arama^1^; Vichada: Cumaribo^1^.

***Centris* *(Hemisiella)* *trigonoides* Lepeletier, 1841**

**Country records**: Antioquia: Caucasia^14^, Medellín^14^, Santa Fé de Antioquia^14^; Atlántico: Juan de Acosta^6^, Puerto Colombia^28^; Bolívar: Cartagena^14^, Santa Cruz de Mompox^1^, Turbaco^14^; Boyacá: Miraflores^1^; Cesar: Aguachica^14^, Becerril^28^, Tamalameque^28^, Valledupar^6^; Cundinamarca: Anolaima^1^, Apulo^1^, Arbeláez^1^, La Palma^1^, Ricaurte^1^, Sasaima^3^, Tocaima^3^; Córdoba: Montería^19^, San Antero^14^; Huila: Acevedo^1^, Tello^1^, Villavieja^7,28^; La Guajira: Maicao^3^; Magdalena: Aracataca^6^, Ciénaga^1^, Santa Marta^1,17,6,28^, Zona Bananera^3^; Santander: Socorro^1^; Sucre: Colosó^1^, Galeras^28^, Majagual^19^, Ovejas^1^, San Onofre^13^.

***Centris* *(Hemisiella)* *vittata* Lepeletier, 1841**

**Country records**: Bolívar: San Juan Nepomuceno^1^; Cesar: Agustín Codazzi^19,28^, Valledupar^14,6,28^; Huila: Rivera^1^; Magdalena: Santa Marta^17^.

**Subgenus *Heterocentris* Cockerell, 1899**

***Centris* *(Heterocentris)* *analis* (Fabricius, 1804)**

**Country records**: Amazonas: Puerto Santander^1,5^; Antioquia: Barbosa^14^, Copacabana^14^, Envigado^9^, Girardota^14^, Itagüí^9^, La Estrella^9^, Medellín^14^, Santa Fe de Antioquia^14^; Bolívar: Cartagena^14^; Caldas: Pácora^15^, Supía^14^, Victoria^1^; Caquetá: Cartagena del Chairá^1^; Casanare: Hato Corozal^1^, San Luís de Palenque^1^; Cauca: Corinto^28^; Cesar: Agustín Codazzi^19^, Valledupar^1^; Cundinamarca: Nilo^1^; Córdoba: ; Guainía: Inírida^11^; Huila: Paicol^1^, Villavieja^7^; Magdalena: Ciénaga^1,3^, Santa Marta^1,6^; Meta: Cumaral^1^, Granada^1^, Mesetas^5^, Puerto Concordia^1^, Puerto Rico^1^, Restrepo^1^, San Juan de Arama^5^, Villavicencio^1^; Quindío: Armenia^15^, La Tebaida^1^; Santander: Cimitarra^1^; Sucre: Coloso^1^, San Onofre^13^; Valle del Cauca: Buenaventura^15^, Buga^14^, Cartago^28^, El Cerrito^12^, Palmira^12,3^, Cali^28^.

***Centris* *(Heterocentris)* *bicornuta* Mocsáry, 1899**

**Country records**: Cesar: Agustín Codazzi^19^

***Centris* *(Heterocentris)* *ceratops* Vélez & Vivallo, 2012**

**Country records**: Sucre: Colosó†^1^.

***Centris* *(Heterocentris)* *difformis* Smith, 1854**

**Country records**: Amazonas: Leticia^17^; Cundinamarca: Viotá^1^; Meta: La Uribe^17^; Tolima: Honda^17,28^

***Centris* *(Heterocentris)* *labrosa* Friese, 1899***

**Country records**: Cundinamarca: Arbeláez^1^, Paime^1^; Meta: Vistahermosa^5^

***Centris* *(Heterocentris)* *terminata* Smith, 1874**

**Country records**: Amazonas: Leticia^1^, Mirití-Paraná^1^, Puerto Santander^1^; Antioquia: Bello^28^, Medellín^28^, Zaragoza^28^; Cundinamarca: Arbeláez^14^, Paime^1,14^, Pulí^1^; Meta: La Macarena^17^, Puerto Rico^1^; Nariño: Ipiales^17^; Putumayo: Puerto Leguízamo^17,28^; Quindío: Armenia^15^, Circasia^15^; Santander: Cimitarra^19^, Zapatoca^28^; Tolima: Armero^14^; Valle del Cauca: Palmira^1^; Vaupés: Mitú^1^; Vichada: Cumaribo^1^.

**Subgenus *Melanocentris* Friese, 1901**

***Centris* *(Melanocentris)* *atriventris*, Mocsáy 1896***

**Country records**: Amazonas: Leticia^1^; Guaviare: San José del Guaviare^17^; Vaupés: Mitú^1^.

***Centris*** *(****Melanocentris)* *bitaeniata* Moure, 2002***

**Country records**: Amazonas: La Pedrera^(Vivallo, 2022a)^.

***Centris* *(Melanocentris)* *caurensis* Cockerell, 1919***

**Country records**: Antioquia: Cocorná^13^.

***Centris* *(Melanocentris)* *clypeata* Lepeletier, 1841***

**Country records**: Cundinamarca: Nocaima^(Vivallo, 2022a)^.

***Centris* *(Melanocentris)* *colombicola* Vivallo, 2022* §**

**Country records**: No specific location records

**Note**: According to Vivallo (2022a), the holotype label reads: ‘MUSEUM

PARIS COLOMBIE Fr. APOLLINAIRE-MARIE 1918’ deposited at MNHP [Muséum National d’Histoire Naturelle, Paris, France].

***Centris* *(Melanocentris)* *cryphia* Vivallo, 2022***

**Country records**: No specific location records.

**Note**: According to Vivallo (2022a), the holotype label reads: ‘Columbien \ 19761 \ Centris atra ♀ det. Friese 1989’ deposited at ZMB [Museum

für Naturkunde, Berlin, Germany].

***Centris* *(Melanocentris)* *furcata* (Fabricius, 1804)***

**Country records**: Boyacá: Nocaima^(Vivallo, 2022a)^; Valle del Cauca: Buenaventura^(Vivallo, 2022a)^

***Centris* *(Melanocentris)* *germani* Vivallo, 2021***

**Country records**: Caquetá: Solano†^(Vivallo, 2021)^; Vaupés: Carurú^1, (Vivallo, 2021)^, Mitú^1, (Vivallo, 2021)^, Papunaua^1^.

***Centris* *(Melanocentris)* *insignis* Smith, 1854**

**Country records**: Antioquia: Santa Fe de Antioquia^14^; Cundinamarca: La Mesa^1^, San Juan de Río Seco^1^, Vergara^1^; Huila: Algeciras^1^; Magdalena: Ciénaga^14^.

***Centris* *(Melanocentris)* *laticincta* (Spinola, 1841)***

**Country records**: Antioquia: San Francisco^28^, Sonsón^1^; Cundinamarca: Nocaima^28^; Huila: Rivera^1^; Magdalena: Ciénaga^6^; Meta: Villavicencio^3^; Santander: Cimitarra^1^, Landázuri^1^.

***Centris* *(Melanocentris)* *mariae* Mocsáry, 1896***

**Country records**: Meta: Villavicencio^(Vivallo, 2022a)^.

***Centris* *(Melanocentris) rhodoprocta* Moure & Seabra, 1960***

**Country records**: Amazonas: La Pedrera^1^; Cauca: Corinto^28^; Guaviare: San José del Guaviare^17^; Meta: Mesetas^1^, San Juan de Arama^1^; Putumayo: Puerto Asís^14^; Valle del Cauca: Santiago de Cali^28^; Vaupés: Mitú^1^, Taraira^13^.

***Centris*** ***(Melanocentris)*** ***rubripes*** **Friese, 1899***

**Country records**: Antioquia: Maceo^13^; Boyacá: La Libertad^(Vivallo, 2022a)^.

***Centris*** ***(Melanocentris)*** ***smithiana*** **Friese, 1901**

**Country records**: Amazonas: La Pedrera^1^; Antioquia: Medellín^28^.

***Centris*** ***(Melanocentris) xylocopoides*** **Fox, 1899***

**Country records**: Caquetá: Belén de los Andaquíes^(Vivallo, 2022a)^.

**Subgenus *Odontoxys* Vivallo 2022**

***Centris*** ***(Odontoxys)*** ***ephippium*** **Smith, 1854***

**Country records**: Meta: Villavicencio^(Vivallo, 2022a)^.

***Centris*** ***(Odontoxys)*** ***marinae*** **Vivallo, 2021***

**Country records**: Cesar: Valledupar†^(Vivallo, 2022a)^.

**Note**: The holotype of *Centris marinae* was deposited in the “Museu Nacional” of Rio de Janeiro, and was destroyed during the fire of 2018

***Centris*** ***(Odontoxys)*** ***obsoleta*** **Lepeletier, 1841**

**Country records**: Cauca: Corinto^28^; Cundinamarca: Nilo^1^; Huila: Neiva^1^, Rivera^1^, Villavieja^7,28^; Meta: San Juan de Arama^1^; Tolima: El Espinal^3^.

**Subgenus *Ptilocentris* Snelling, 1984**

***Centris*** ***(Ptilocentris)*** ***deiopeia*** **Gribodo, 1892**

**Country records**: Antioquia†^(Gribodo, 1891)^; Cauca: Popayán^28^.

***Centris*** ***(Ptilocentris) festiva*** **Smith, 1854**

**Country records**: Antioquia: Bolívar^14^, Caldas^9^, Guatapé^13^, Medellín^4,1428^, San Pedro de los Milagros^28^; Santa Elena^¹⁴^; Boyacá: Buenavista^1^, Nuevo Colón^1^; Caquetá: Florencia^1^; Cauca: Caloto^12^, Patía^16^; Cundinamarca: San Antonio del Tequendama^7^, Une^14^; Huila: Colombia^1^; Magdalena: Aracataca^17,28^; Nariño: La Unión^1^, Pasto^3^, Ricaurte^17,28^; Norte de Santander: Pamplona^28^; Putumayo: Mocoa^17,28^, Sibundoy^28^; Quindío: Filandia^15^; Risaralda: Pereira^1^; Valle del Cauca: El Dovio^1^, Cali^12,20^.

***Centris*** ***(Ptilocentris)*** cf. ***semicaerulea***

**Country records**: Antioquia: Caldas^14^, Concepción^14^, Piedras Blancas¹⁴, San Carlos^14^.

**Subgenus *Ptilotopus* Klug, 1810**

***Centris*** ***(Ptilotopus)*** ***americana*** **(Klug, 1810)**

**Country records**: Caquetá: Florencia^13^; Meta: Mesetas^5^.

***Centris*** ***(Ptilotopus)*** ***denudans*** Lepeletier, 1841

**Country records**: Amazonas: Leticia^2,28^, Puerto Nariño^7^; Guainía: Inírida^11^; Guaviare: El Retorno^17^, San José del Guaviare^5^; Meta: Puerto Rico^1^.

***Centris*** ***(Ptilotopus)*** ***derasa*** Lepeletier, 1841*

**Country records**: Amazonas: Leticia^7^; Vichada: Cumaribo^5^.

***Centris* *(Ptilotopus)* *dimidiata* (Olivier, 1789)**

**Country records**: Norte de Santander: Cúcuta^1^.

***Centris* *(Ptilotopus)* *erythrosara* Moure & Seabra, 1960**

**Country records:** Antioquia: Medellín^5^, Puerto Nare^28^, San Roque^13^; Cundinamarca: Anapoima^28^, Quipile^28^, Tocaima^7^; Huila: Algeciras^1^, Baraya^1^, Colombia^1^, Gigante^1^, Neiva^1,17^, Rivera^1^, Tello^1^, Villavieja^7^; Magdalena: Santa Marta^5^; Meta: Puerto López^18^; Tolima: Chaparral^12^, El Espinal^3^, Melgar^1^.

***Centris* *(Ptilotopus)* *nobilis* Westwood, 1840***

**Country records:** Caquetá: Morelia^1^; Guainía: Inírida^11^; Meta: Vistahermosa^20^.

***Centris* *(Ptilotopus)* *superba* Ducke, 1904***

**Country records:** Guainía: Inírida^11^.

**Subgenus *Schisthemisia* Ayala, 2002**

***Centris* *(Schisthemisia)* *flavilabris*, Mocsáry 1899**

**Country records:** Amazonas: La Pedrera^1^, Leticia^1^; Antioquia: Medellín^14^; Guaviare: San José del Guaviare^2^; Vaupés: Mitú^1^.

***Centris* *(Schisthemisia)* *fulva*, Friese, 1924***

**Country records**: Amazonas: Leticia^1^, Mirití-Paraná^1^; Vaupés: Mitú^1^.

***Centris* *(Schisthemisia)* *restrepoi* Moure, 2002 §**

**Country records**: Risaralda: Mistrató†^(Moure, Urban & Melo, 2022; Vivallo, 2022b)^.

***Centris* *(Schisthemisia)*** ***scutellata* Friese, 1901***

**Country records**: Cundinamarca^(Vivallo, 2022b)^.

**Subgenus *Trachina* Klug, 1807**

***Centris* *(Trachina)* *claripennis* Friese, 1900***

**Country records**: Atlántico: Polonuevo^14^; Magdalena: Santa Marta^1,6^, Zona Bananera^6^; Quindío: Quimbaya^6^

***Centris* *(Trachina)* *dentata* Smith, 1854***

**Country records:** Antioquia: Santa Fe de Antioquia^14^; Meta: Mesetas^1^, Puerto Rico^1^.

***Centris* *(Trachina) fuscata* Lepeletier, 1841**

**Country records**: Bolívar: Cartagena^28^; Magdalena: Santa Marta^28^

***Centris* *(Trachina)* *longimana* Fabricius, 1804**

**Country records**: Amazonas: La Pedrera^1^, Leticia^1,17,5,20^, Mirití-Paraná^1^, Puerto Santander^5^; Antioquia: Mutatá^14^, San Francisco^13^; Meta: San Martín^2^, Uribe^20^, Villavicencio^1^, Vistahermosa^1,5^; Putumayo: Puerto Leguízamo^1^; Valle del Cauca: Buenaventura^20^; Vaupés: Mitú^1^.

***Centris* *(Trachina)* *pachysoma* Cockerell, 1919**

**Country records**: Amazonas: La Pedrera^1^; Vaupés: Mitú^1^.

***Centris* *(Trachina)* *similis* (Fabricius, 1804)**

**Country records**: Amazonas: Leticia^17,20^; Antioquia: Bello^28^, Copacabana^14^, Medellín^13,14,28^, Zaragoza^28^; Caquetá: Morelia^1^; Casanare: Yopal^1^; Cauca: Santander de Quilichao^14^; Cundinamarca: Chía^2^, Medina^1^; Magdalena: Zona Bananera^6^; Meta: Guamal^1^, Mesetas^5,20^, Puerto Rico^1^, Uribe^5,20^, Villavicencio^1^; Quindío: Armenia^28^, Calarcá^15^, Quimbaya^13^; Santander: Cimitarra^1^; Valle del Cauca: Buga^12,14^, Dagua^12^, El Dovio^1^, La Unión^12^, Palmira^12^, Roldanillo^12^, Santiago de Cali^28,20^.

***Centris* *(Trachina)* *vidua* Mocsáry, 1899***

**Country records**: Nariño: Tumaco^1^

**Subgenus *Xanthemisia* Moure, 1945**

***Centris* *(Xanthemisia)* *aureiventris* Vivallo, Vélez & Fernández, 2013***

**Country records**: Boyacá: Buenavista^1^; Huila: La Argentina^1^; Valle del Cauca: El Dovio†^1^.

***Centris* *(Xanthemisia)* *ferruginea* Lepeletier, 1841**

**Country records**: Amazonas: La Pedrera^1^, Mirití-Paraná^1^; Antioquia: Puerto Berrío^13^, Yolombó^28^; Caquetá: Solano^28^; Guainía: Inírida^11^; Guaviare: San José del Guaviare^17^; Meta: San Martín^2^.

***Centris* *(Xanthemisia)* *lutea* Friese, 1899**

**Country records**: Antioquia: Bello^3^; Cundinamarca: Fusagasugá^5^; Huila: Villavieja^7^; Valle del Cauca: Cali^16^.

**Genus *Epicharis* Klug, 1807**

**Subgenus *Epicharis* Klug, 1807**

***Epicharis* *(Epicharis) conica* Smith, 1874***

**Country records**: Guaviare: Solano^18^.

***Epicharis* *(Epicharis) flava* (Friese, 1901)**

**Country records**: Amazonas: La Pedrera^1^, Puerto Santander^1^; Antioquia: Amalfi^14^, Bello^3,28^, El Retiro^14^, Hatillo^14^, Medellín^3,28^, La Pintada^14^, Yolombó^14^; Casanare: Villanueva^1^; Cundinamarca: Paratebueno^1^; Guaviare: San José del Guaviare^1^; Huila: La Argentina^1^, Rivera^1^; Meta: Acacías^1^, Cumaral^1^, La Macarena^1^, Puerto Rico^1^, San Juan de Arama^1^, Villavicencio^1,3^; Tolima: Icononzo^1^; Valle del Cauca: El Dovio^1^, Tuluá^1^.

***Epicharis* *(Epicharis)* *lindigii* Friese, 1899**

**Country records**: Antioquia: Medellín^14^; Chocó: San José del Palmar^1^; Huila: Algeciras^1^; Valle del Cauca: El Dovio^1^.

***Epicharis* *(Epicharis)* *rustica* (Olivier, 1789)**

**Country records**: Antioquia: Amalfi^14,28,^, Barbosa^14^, Bello^3,28^, Caucasia^13^, Ciudad Bolívar^13^, Envigado^13^, Hatillo^14^, Maceo^13,14^, Medellín^13,14^,La Pintada^14^, Santa Fe de Antioquia^14^, Santuario^13^, Yolombó^28^; Boyacá: Buenavista^1^, Pajarito^1^; Caldas: Chinchiná^1^; Caquetá: San Vicente del Caguán^1^; Casanare: Chámeza^1^; Cesar: Pueblo Bello^1^; Cundinamarca: La Vega^1^, Sasaima^3^; Huila: Algeciras^1^, Hobo^1^, Rivera^1^; Nariño: Chachagüí^1^; Quindío: Armenia^28^, Filandia^13^; Risaralda: Pereira^1^; Santander: Cimitarra^1,13^, El Carmen de Chucurí^13^, Puerto Parra^3^, Rionegro^13^; Tolima: Rovira^13^; Valle del Cauca: El Cairo^1^, El Dovio^1^, Guacarí^1^, Cali^28,20^.

***Epicharis* *(Epicharis)* *umbraculata* (Fabricius, 1804)***

**Country records**: Casanare: Villanueva^1^; Meta: Puerto Gaitán^1^; Valle del Cauca: Cali^1^; Vichada: Cumaribo^17^

**Subgenus *Epicharitides* Moure, 1945**

***Epicharis* *(Epicharitides)* *duckei* Friese, 1901***

**Country records**: Meta: La Macarena ^(Villamizar & Vivallo, 2024)^.

**Subgenus *Epicharoides* Radoszkowski, 1884**

***Epicharis* *(Epicharoides) albofasciata* Smith, 1874**

**Country records**: Antioquia: Yolombó^28^; Guaviare: San José del Guaviare^1^; Huila: Paicol^1^, Villavieja^7^; Meta: Puerto Rico^1^.

***Epicharis* *(Epicharoides)* *maculata* Smith, 1874**

**Country records**: Antioquia: Amalfi^28^, Puerto Berrío^13^, Yolombó^28^.

**Subgenus *Hoplepicharis* Moure, 1945**

***Epicharis* *(Hoplepicharis)* *affinis* Smith, 1874***

**Country records**: Amazonas: Leticia^17^; Meta: La Macarena^20^.

***Epicharis* *(Hoplepicharis) fasciata* Lepeletier & Audinet-Serville, 1828**

**Country records**: Huila: Rivera^1^; Meta: San Juan de Arama^1^; Risaralda: Pereira^13^; Santander: Cimitarra^1^; Valle del Cauca: Guacarí^1^, Santiago de Cali^20^.

***Epicharis* *(Hoplepicharis)* *monozona* Mocsáry, 1898**

**Country records**: Antioquia: Amalfi^28^, Maceo^13^, San Jerónimo^13^, Yolombó^28^; Huila: Neiva^17^.

**Subgenus *Parepicharis* Moure, 1945**

***Epicharis* *(Parepicharis)* *metatarsalis* Friese, 1899**

**Country records**: Valle del Cauca: Calima†^(Friese, 1899a)^.

***Epicharis* *(Parepicharis)* *zonata* Smith, 1854**

**Country records**: Antioquia: San Francisco^17^; Casanare: Villanueva^1^; Guaviare: San José del Guaviare^1^; Meta: San Juan de Arama^1^.

**Euglossini Latreille, 1802**

**Genus *Aglae* Lepeletier & Serville, 1825**

***Aglae* *caerulea* Lepeletier & Serville, 1825**

**Country records**: Caquetá: San Vicente del Caguán^1^; Chocó: Bahía Solano^1,11,28^; Meta: Fuente de Oro^1,5^, Villavicencio^1,20^; Santander: Cimitarra^18^.

**Genus *Eufriesea* Cockerell, 1908**

***Eufriesea* *auripes* (Gribodo, 1882)**

**Country records**: Caquetá: Solano^28^; Meta: San Juan de Arama^1^.

***Eufriesea* *bare* González & Gaiani, 1989**

**Country records:** Cauca: Piamonte^11^; Guainía: Inírida^11^, San Felipe^11^; Putumayo: Mocoa^11^; Vaupés: Taraira^1^.

***Eufriesea* *chrysopyga* (Mocsáry, 1898)**

**Country records**: Cauca: Piamonte^16^; Chocó: Bahía Solano^1^, Nuquí^1^; Magdalena: Santa Marta^1,6,28^; Putumayo: Mocoa^1^; Valle del Cauca: Buenaventura^28^, Calima^28^, Dagua^28^.

***Eufriesea* *concava* (Friese, 1899)**

**Country records**: Antioquia: Amalfi^28^, Yolombó^28^; Atlántico: Barranquilla^1^; Bolívar: San Juán Nepomuceno^1^; Boyacá: Puerto Boyacá^1^; Cundinamarca: La Vega^1^; Magdalena: Ciénaga^28^, Santa Marta^28^; Quindío: Quimbaya^28^; Risaralda: Pereira^13^; Santander: Suaita^1^.

***Eufriesea* *distinguenda* (Gribodo, 1882)***

**Country records**: Cauca: Balboa^11^, Patía^11^; Meta: La Macarena^1^.

***Eufriesea* *dressleri* (Kimsey, 1977)**

**Country records**: Antioquia: Puerto Nare^13^; Casanare: Villanueva^1^.

***Eufriesea* *duckei* (Friese, 1923)**

**Country records**: Magdalena: Santa Marta^28^

***Eufriesea* *elegans* (Lepeletier, 1841)**

**Country records**: Antioquia: Yolombó^28^; Cundinamarca: Medina^5^; Meta: San Martín^8^.

***Eufriesea* *excellens* (Friese, 1925)**

**Country records**: Putumayo: Mocoa ^(Londoño-Carvajal, 2022)^.

***Eufriesea* *flaviventris* (Friese, 1899)**

**Country records**: Caldas: Pácora^15^.

***Eufriesea* *fragrocara* (Kimsey, 1977)**

**Country records**: Caquetá: Solano^28^

***Eufriesea* *laniventris* (Ducke, 1902)***

**Country records**: Cauca: Piamonte^11^; Guainía: Inírida^11^, La Guadalupe^11^, San Felipe^11^.

***Eufriesea* *limbata* (Mocsáry, 1897)***

**Country records**: Nariño: Barbacoas^17^.

***Eufriesea* *longipennis* (Friese, 1925)***

**Country records**: Caquetá: Florencia^5^; Huila: Neiva^1^.

***Eufriesea* *lucida* (Kimsey, 1977)**

**Country records**: Valle del Cauca: Buenaventura^(Kimsey, 1977)^.

***Eufriesea* *lucifera* Kimsey, 1977**

**Country records**: Antioquia: San Francisco^18^, Yolombó^28^; Boyacá: Otanche^28^; Chocó: Bahía Solano^28^, Lloró^28^; Cundinamarca: Nilo^7^; Nariño: Barbacoas^1^, Francisco Pizarro^1^, Tumaco^1^; Valle del Cauca: Buenaventura^1^, Dagua^28^.

***Eufriesea* *macroglossa* (Moure, 1965)**

**Country records**: Antioquia: Amalfi^28^, Valparaíso^13^, Yolombó^28^; Chocó: Acandí^13^, Bahía Solano^11^.

***Eufriesea* *magrettii* (Friese, 1899)**

**Country records**: Antioquia: Alejandría^13^, Anorí^28^; Boyacá: Otanche^28^; Caquetá: San José del Fragüa^1^; Cauca: Santa Rosa^28^; Magdalena: Ciénaga^6^, Santa Marta^1^; Meta: Villavicencio^28^; Norte de Santander: Toledo^1^; Putumayo: Mocoa^1^; Santander: Socorro^7^; Valle del Cauca: Dagua^28^; Vichada: Cumaribo^17,28^.

***Eufriesea* *mussitans* (Fabricius, 1787)**

**Country records**: Antioquia: Amalfi^28^, Yolombó^28^; Nariño: Barbacoas^1^; Putumayo: Mocoa^1^.

***Eufriesea* *nigrescens* (Friese, 1923)**

**Country records**: Boyacá: Santa María^1^; Caldas: Aguadas^15,28^; Meta: Puerto López^1^.

***Eufriesea* *ornata* (Mocsáry, 1896)**

**Country records**: Casanare: Trinidad^1^; Chocó: Litoral del Bajo San Juan^1^; Córdoba: Tierralta^1^; Meta: La Macarena^5^; Nariño: Francisco Pizarro^1^; Putumayo: Mocoa^28^; Valle del Cauca: Buenaventura^11,15^.

***Eufriesea* *pretiosa* (Friese, 1903)**

**Country records**: Caquetá: Florencia ^(Londoño-Carvajal, 2022)^; Nariño: Barbacoas ^(Londoño-Carvajal, 2022)^; Risaralda: Pueblo Rico ^(Londoño-Carvajal, 2022)^; Valle del Cauca: Dagua ^(Londoño-Carvajal, 2022)^.

***Eufriesea* *pulchra* (Smith, 1854)**

**Country records**: Amazonas: La Pedrera^1^, Leticia^1^, Puerto Ñarino^1^; Antioquia: Anorí^13^, Puerto Nare^13^, San Francisco^13,17^, San Luís^13^, Sonsón^17^; Boyacá: Otanche^28^, Puerto Boyacá^28^; Caquetá: Florencia^1^, Solano^17,28^; Cauca: Piamonte^11^, Santa Rosa^11,28^; Chocó: Bahía Solano^1,28^, Litoral del Bajo San Juan^1^, Nuquí^4^, Quibdó^15^; Cundinamarca: Nilo^7^; Córdoba: Montería^1^, Tierralta^1^; Guainía: Inírida^1,11,5^, La Guadalupe^11^, San Felipe^11^; Meta: Cubarral^1^; Nariño: Francisco Pizarro^1^, Tumaco^1^; Norte de Santander: Chitagá^1^; Putumayo: Mocoa^11,28^, Puerto Leguízamo^1^; Risaralda: Pereira^13^; Santander: Cimitarra^1^; Valle del Cauca: Buenaventura^1,28^; Vaupés: Mitú^1^.

***Eufriesea* *purpurata* (Mocsáry, 1896)**

**Country records**: Amazonas: Leticia^1,28,20^, Puerto Nariño^1^, Puerto Santander^1,5^, Tarapacá^1^; Antioquia: Yolombó^28^; Caquetá: Florencia^1^; Solano, Chocó: Bahía Solano^11^; Cundinamarca: Fusagasugá^1^; Meta: Puerto Concordia^1^; Santander: Bucaramanga^1^, Cimitarra^1^; Tolima: Ambalema^28^; Valle del Cauca: Buenaventura^1^; Vichada: Cumaribo^5^.

***Eufriesea* *rufocauda* (Kimsey, 1977)**

**Country records**: Cauca: Santa Rosa^11^; Valle del Cauca: Dagua^28^.

***Eufriesea* *surinamensis* (Linnaeus, 1758)**

**Country records**: Amazonas: La Chorrera^13^, La Pedrera^1^; Antioquia: Caucasia^13^, Frontino^1^, Sonsón^17^, Yondó^1^; Boyacá: Miraflores^1^, Otanche^1^, Puerto Boyacá^1^, Quipama^1^, Santa María^1^; Caldas: La Dorada^1^, Viterbo^28^; Casanare: Hato Corozal^1^, Villanueva^1^; Cauca: Corinto^28^ Chocó: Acandí^13^, Litoral del Bajo San Juan^1^, Lloró^28^, Quibdó^1^, San José del Palmar^1^; Cundinamarca: Agua de Dios^7^, Apulo^1^, Caparrapí^1^, Fusagasugá^1,5^, La Vega^1^, Silvania^1,15^; Huila: Acevedo^1^, Gigante^1^, Neiva^1,17^, Palermo^1^, Villavieja^7^; Magdalena: Santa Marta^1,15,28^, Zona Bananera^1^; Meta: Cumaral^1^, El Castillo^1^, Puerto López^1^, Restrepo^1^, San Juan de Arama^1^, Villavicencio^1,28^; Nariño: El Charco^1^, Tumaco^1,3^; Quindío: Armenia^15^, Buenaventura^15^, Circasia^15,28^, Córdoba^15^, Quimbaya^15,28^; Santander: Cimitarra^20^, Suaita^1^; Tolima: Cunday^1^, Honda^17,28^; Valle del Cauca: Bugalagrande^1^; Vaupés: Mitú^1^.

***Eufriesea* *theresiae* (Mocsáry, 1908)**

**Country records**: Caquetá: San Lorenzo (Londoño-Carvajal, 2022), Solano^17,(Londoño-Carvajal, 2022)^; Cauca: Santa Rosa ^(Londoño-Carvajal, 2022)^; Putumayo: Mocoa^17,(Londoño-Carvajal, 2022)^.

***Eufriesea* *venusta* (Moure, 1965)**

**Country records**: Nariño: Barbacoas^1^

***Eufriesea* *vidua* (Moure, 1976)**

**Country records**: Amazonas: Puerto Nariño^1^

***Eufriesea* *violascens* (Mocsáry, 1898)**

**Country records**: Meta: La Macarena^1^, Puerto López^1^, San Martín^1^; Vichada: Cumaribo^17^, Puerto Carreño^17^.

**Genus *Euglossa* Latreille, 1802**

**Subgenus *Alloglossura* Hinojosa-Díaz & Engel, 2012**

***Euglossa* *(Alloglossura)* *fuscifrons* Dressler, 1982**

**Country records**: Putumayo: Puerto Asís^1^

***Euglossa* *(Alloglossura) gorgonensis* Cheesman, 1929**

**Country records**: Antioquia: San Francisco^17^; Boyacá: Otanche^7^; Chocó: Bahía Solano^1^, Lloró^15,28^, Nuquí^1^, Quibdó^15^; Nariño: Barbacoas^1^; Valle del Cauca: Buenaventura^1,5^, Dagua^20^, Gorgona†^(Cheesman, 1929)^.

***Euglossa* *(Alloglossura)* *paisa* Ramírez, 2005**

**Country records**: Antioquia: Anorí†^1,17,5,28^; Quindío: Circasia^15,28^.

***Euglossa* *(Alloglossura)* *stilbonota* Dressler, 1982**

**Country records**: Amazonas: Leticia^1,28^; Caquetá: Solano^17^, Valparaíso^1^; Huila: Rivera^1^; Putumayo: Mocoa^28^, Puerto Leguízamo^1^.

***Euglossa* *(Alloglossura) trinotata* Dressler, 1982**

**Country records**: Antioquia: Urrao^20^; Huila: Rivera^1^; Nariño: Barbacoas^1^

**Subgenus *Euglossa* Latreille, 1802**

***Euglossa* *(Euglossa)* *amazonica* Dressler, 1982**

**Country records**: Amazonas: Puerto Ñarino^1^; Arauca: Arauca^28^; Caquetá: El Paujil^1^; Cundinamarca: Puerto Salgar^1^; Meta: La Macarena^20^, Puerto Rico^1^, San Juan de Arama^1,5^; Putumayo: Puerto Asís^1,20^.

***Euglossa* *(Euglossa)* *analis* Westwood, 1840**

**Country records**: Amazonas: Leticia^17^, Puerto Ñarino^1^; Caldas: Norcasia^17^; Caquetá: Florencia^1^; San Vicente Del Caguán^¹^; Cauca: Santa Rosa^28^; Magdalena: Santa Marta^6,28^; Putumayo: Mocoa^28^, Puerto Leguízamo^17^; Vichada: Cumaribo^17,28^.

***Euglossa* *(Euglossa)* *azureoviridis* Friese, 1930***

**Country records**: Boyacá: Puerto Boyacá^¹^ Chocó: Bahía Solano^28^; Santander: Landázuri^¹^.

***Euglossa* *(Euglossa)* *bidentata* Dressler, 1982**

**Country records**: Amazonas: Leticia^7^; Caquetá: Solano^17,28^; Guainía: Inírida^5^; Meta: San Martín^28^, Villavicencio^1^; Vichada: Cumaribo^17^.

***Euglossa* *(Euglossa)* *championi* Cheesman, 1929**

**Country records**: Antioquia: Amalfi^1^, San Francisco^17^, Sonsón^17^, Yolombó^28^; Boyacá: Labranzagrande^1^; Cauca: Guapi^1^; Chocó: Bahía Solano^28^, Lloró^28^, Nuquí^1^, Quibdó^15^; Cundinamarca: Nilo^7^; Nariño: Barbacoas^1^, Francisco Pizarro^1^; Valle del Cauca: Buenaventura^1,11,5,20^, Dagua^1^.

***Euglossa* *(Euglossa) chlorina* Dressler, 1982**

**Country records**: Antioquia: Anorí^28^, San Francisco^17^, Sonsón^17^; Arauca: Arauca^28^; Bolívar: Cartagena^2^; Caldas: Chinchiná^28^, Viterbo^28^; Caquetá: Valparaíso^1^; Cauca: Guapi^17^; Cesar: Valledupar^28^; Cundinamarca: Agua de Dios^7^; Meta: Villavicencio^28,20^; Santander: Socorro^7^; Valle del Cauca: Buenaventura^1^.

***Euglossa* *(Euglossa) cognata* Moure, 1970**

**Country records**: Amazonas: Leticia^17^, Puerto Ñarino^1^; Antioquia: Murindo^¹^; Caquetá: El Paujil^1^, San José del Fragüa^1^, Solano^17,28^; Casanare: Villanueva^1^; Chocó: Bahía Solano^11,28^, Murindó^1^, Nuquí^1^; Cundinamarca: Nilo^7^; Magdalena: Santa Marta^1,15,28^; Meta: Villavicencio^1^; Nariño: Tumaco^1^; Putumayo: Mocoa^1,28^; Santander: Bolivar^17^; Vichada: Cumaribo^17,28^.

***Euglossa* *(Euglossa)* *cordata* (Linnaeus, 1758)**

**Country records**: Antioquia: Medellín^28^; Bolívar: San Juan Nepomuceno^1^; Valle del Cauca: P almira^1^, Santiago de Cali^11^.

***Euglossa* *(Euglossa)* *cyanaspis* Moure, 1968***

**Country records**: Bolívar: Santa Cruz de  Mompox^1^.

***Euglossa* *(Euglossa)* *cybelia* Moure, 1968**

**Country records**: Antioquia: Amalfi^28^, Anorí^28^, San Francisco^17^, Sonsón^17^, Yolombó^28^; Boyacá: Otanche^7^, Santa María^1^; Cundinamarca: Nilo^7^; Magdalena: Santa Marta^1^; Nariño: Barbacoas^1^; Norte de Santander: Toledo^1,17,28^; Santander: Socorro^7^; Valle del Cauca: Calima^28^.

***Euglossa* *(Euglossa)* *deceptrix* Moure, 1968**

**Country records**: Antioquia: Amalfi^1,28^,Amalfí^¹^; Bello^28^, Medellín^28^, San Francisco^17^, Sonsón^17^, Yolombó^28^; Arauca: Arauca^28^; Bolívar: Mahates^1^, San Jacinto^1^, San Juan Nepomuceno^1^; Boyacá: Otanche^7,^; Chocó: Lloró^28^; Cundinamarca: Agua de Dios^7^, Apulo^1^, Arbeláez^1^, Pandi^1^, Sasaima^1,28^, Tena^1^; Huila: La Argentina^1^, Palestina^1^, Pitalito^1^; Magdalena: Santa Marta^1,15,28^; Meta: Acacías^1^, Puerto Rico^1^, Restrepo^1^, San Juan de Arama^1^, Villavicencio^1^; Quindío: Quimbaya^15^; Risaralda: Santuario^1^; Santander: Floridablanca^1^, Socorro^7^, Suaita^1^; Tolima: Chaparral^1^, Cunday^1^, Dolores^1^, San Sebastián de Mariquita^1^; Valle del Cauca: Dagua^20^, El Dovio^1^, Palmira^12^ ; Santiago de Cali^28,20^, Yotoco^15,28^; Vichada: Cumaribo^17,28^.

***Euglossa* *(Euglossa)* *despecta* Moure, 1968**

**Country records**: Antioquia: San Francisco^17^, Yolombó^28^; Boyacá: Otanche^7^, Santa María^1^; Chocó: Bahía Solano^1,28^, Lloró^28^; Huila: Neiva^1,5^; Magdalena: Santa Marta^15,28^; Meta: Acacías^1^, Cubarral^1^, La Macarena^1^, Villavicencio^28^; Norte de Santander: Cúcuta^28^; Quindío: Circasia^15^, Quimbaya^15,28^; Santander: Socorro^7^; Valle del Cauca: Buenaventura^1^.

***Euglossa* *(Euglossa)* *dilemma* Bembé & Eltz, 2011***

**Country records**: San Andrés y Providencia: San Andrés^17^

***Euglossa* *(Euglossa)* *dissimula* Dressler, 1978**

**Country records**: Antioquia: San Francisco^17^, Sonsón^17^; Chocó: Bahía Solano^28^, Juradó^20^; Córdoba: Ayapel^1^; Putumayo: Mocoa^28^.

***Euglossa* *(Euglossa)* *dressleri* Moure, 1968**

**Country records**: Antioquia: Amalfi^28^, San Francisco^17^, Yolombó^28^; Bolívar: San Juan Nepomuceno^1^; Boyacá: Otanche^7^; Caquetá: Solano^28^; Cesar: Valledupar^28^; Chocó: Acandí^1^, Bahía Solano^1^, Lloró^28^, Nuquí^1^, Quibdó^1^; Nariño: Barbacoas^1^; Valle del Cauca: Buenaventura^1,20^, Dagua^1^, Palmira^12^, Santiago de Cali^11^.

***Euglossa* *(Euglossa)* *gaianii* Dressler, 1982**

**Country records**: Antioquia: Puerto Nare^13^; Caldas: Manizales^12^; Caquetá: San José del Fragua, San Vicente del Caguán^1^; Chocó: Bahía Solano^1^; Meta: Villavicencio^28^; Nariño: Tumaco^1^.

***Euglossa* *(Euglossa)* *gibbosa* Dressler, 1982**

**Country records**: Chocó: Nuquí^1^

***Euglossa* *(Euglossa)* *hansoni* Moure, 1965**

**Country records**: Antioquia: Sonsón^17^, Yolombó^28^; Boyacá: Otanche^7^; Chocó: Bahía Solano^1,28^, Lloró^15,28^, Nuquí^1^, Quibdó^1,15^; Nariño: Barbacoas^1^; Valle del Cauca: Buenaventura^1,20,28^, Dagua^28^

***Euglossa* *(Euglossa)* *hemichlora* Cockerell, 1917**

**Country records**: Antioquia: Amalfi^28^, Anorí^9^, Yolombó^28^; Arauca: Arauca^28^; Boyacá: Miraflores^1^; Caquetá: El Paujil^1^, Solano^28^; Cesar: Valledupar^6^; Chocó: Bahía Solano^1,28^, Lloró^28^; Magdalena: Santa Marta^15,28^; Meta: Acacías^1^, Restrepo^1^, Villavicencio^1^; Nariño: Tumaco^1^; Santander: Floridablanca^1^, Socorro^7^; Valle del Cauca: Buenaventura^1^, Cali^1^; Vichada: Cumaribo^17,28^.

***Euglossa* *(Euglossa)* *heterosticta* Moure, 1968**

**Country records**: Amazonas: Leticia^17^; Antioquia: Bello^28^, San Francisco^17^, Sonsón^17^; Boyacá: Otanche^7^; Caldas: Chinchiná^28^, Viterbo^28^; Chocó: Bahía Solano^28^, Lloró^28^, Quibdó^15^; Cundinamarca: Silvania^15^; Magdalena: Santa Marta^1^; Meta: Acacías^1^, San Juan de Arama^1^, Villavicencio^1^; Nariño: Barbacoas^1^, Francisco Pizarro^1^, Tumaco^1^; Quindío: Armenia^15^; Santander: Socorro^7^; Tolima: San Sebastián de Mariquita^1^; Valle del Cauca: Buenaventura^20^, Palmira^1,3^.

***Euglossa* *(Euglossa)* *igniventris* Friese, 1925**

**Country records**: Antioquia: Anorí^28^, Sonsón^17^; Arauca: Arauca^28^; Caldas: Viterbo^28^; Caquetá: Miraflores^17^, Solano^17^; Meta: Cubarral^1^, Cumaral^1^.

***Euglossa* *(Euglossa)* *ioprosopa* Dressler, 1982**

**Country records**: Amazonas: La Pedrera^1^, Leticia^1,28^; Caquetá: El Paujil^1^, Solano^17^; Chocó: Bahía Solano^1^; Cundinamarca: Zipaquirá^1^; Meta: San Martín^28^.

***Euglossa* *(Euglossa)* *iopyrrha* Dressler, 1982***

**Country records**: Amazonas: Leticia^2^

***Euglossa* *(Euglossa)* *liopoda* Dressler, 1982**

**Country records**: Antioquia: Medellín^28^; Arauca: Arauca^28^; Boyacá: Otanche^7^; Caquetá: Miraflores^17^, Solano^17,28^; Cauca: Mondomo^12^; Cundinamarca: Agua de Dios^7^; Magdalena: Santa Marta^1^; Meta: Puerto López^2^; Santander: Socorro^7^; Vichada: Cumaribo^17,28^.

***Euglossa* *(Euglossa)* *maculilabris* Moure, 1968**

**Country records**: Antioquia: San Luís^1^; Cundinamarca: Silvania^15^; Nariño: Barbacoas^1^; Quindío: Circasia^15,28^, Quimbaya^15^; Valle del Cauca: Buenaventura^1,5^.

***Euglossa* *(Euglossa)* *magnipes* Dressler, 1982**

**Country records**: Amazonas: La Pedrera^1^; Antioquia: San Francisco^1^; Boyacá: Santa María^1^; Chocó: Bahía Solano^11^; Cundinamarca: Silvania^15^; Huila: Rivera^1^; Meta: Acacias^1^, Puerto Rico^1^, Restrepo^1^, Villavicencio^1^.

***Euglossa* *(Euglossa)* *milenae* Bembé, 2007**

**Country records**: Antioquia: Sonsón^17^; Chocó: Bahía Solano^(Bembé, 2007)^.

***Euglossa* *(Euglossa)* *mixta* Friese, 1899**

Country records: Amazonas: La Pedrera^1^, Leticia^1,17,7^, Puerto Ñarino^1^; Antioquia: Amalfi^1,28^, Murindo^¹^ ; San Francisco^1,17^, Sonsón^17^, Yolombó^28^; Arauca: Arauca^28^; Bolívar: San Jacinto^1^; Boyacá: Labranzagrande^1^, Otanche^7,28^, Puerto Boyacá^28^, San Luis de Gaceno^1^, Santa María^1^; Caldas: La Dorada^28^; Caquetá: Albania^1^, El Paujil^1^, Florencia^1^, San Vicente del Caguán^1^, Solano^17,28^; Casanare: Villanueva^1^; Cauca: Inzá^1^; Chocó: Bahía Solano^1,11^, Cundinamarca: Medina^17^, Nilo^7^, Paime^1^, Sasaima^1^, Tena^1^; Magdalena: Santa Marta^1^; Meta: Cubarral^1^, La Macarena^1^, Mesetas^1^, Puerto Rico^1^, San Martín^28^, Villavicencio^1,28^; Nariño: Barbacoas^1^, Francisco Pizarro^1^, Tumaco^1^; Putumayo: Mocoa^28^; Risaralda: Pereira^1^; Santander: Cimitarra^1^, Socorro^7^; Tolima: Dolores^1^, San Sebastián de Mariquita^1^; Valle del Cauca: Buenaventura^1^, Calima^28^, Dagua^28^; Vichada: Cumaribo^17^.

***Euglossa* *(Euglossa)* *modestior* Dressler, 1982**

**Country records**: Amazonas: La Pedrera^1^, Leticia^1^; Arauca: Arauca^28^; Boyacá: Otanche^7^; Caquetá: El Paujil^1^; Casanare: Orocué^1^, San Luís de Palenque^1^, Trinidad^1^, Villanueva^1^; Cesar: Valledupar^28^; Meta: Acacías^1,17^, Granada^1^, Guamal^1^, La Macarena^1^, Puerto Rico^1^, Restrepo^1^, San Juan de Arama^1^, Villavicencio^1,28,20^; Quindío: Quimbaya^15^; Risaralda: Santuario^1^; Valle del Cauca: Buenaventura^1^; Vaupés: Mitú^1^; Vichada: Cumaribo^17,28^.

***Euglossa* *(Euglossa)* *mourei* Dressler, 1982**

**Country records**: Amazonas: Leticia†^1,28^; Arauca: Arauca^28^; Caquetá: Valparaíso^1^; Meta: Villavicencio^28^; Nariño: Barbacoas^1^.

***Euglossa* *(Euglossa)* *nigropilosa* Moure, 1965**

**Country records**: Antioquia: Medellín^1^; Boyacá: Puerto Boyacá^1^; Caquetá: Florencia^28^, San José del Fragüa^1^; Cundinamarca: Tena^1^; Nariño: Barbacoas^1^, Los Andes^11^; Quindío: Circasia^15,28^, Quimbaya^15^; Santander: Socorro^7^, Tona^1^; Valle del Cauca: Santiago de Cali^20^,

***Euglossa* *(Euglossa)* *retroviridis* Dressler, 1982**

**Country records**: Amazonas: La Pedrera^1^ ; Leticia†^28^; Cauca: Santa Rosa^28^; Putumayo: Mocoa^28,^ Puerto Asís^20^

***Euglossa* *(Euglossa) securigera* Dressler, 1982***

**Country records**: Meta: La Macarena^20^

***Euglossa* *(Euglossa)* *townsendi* Cockerell, 1904**

**Country records**: Amazonas: Leticia^17^; Antioquia: Amalfi^28^, Medellín^28^, Yolombó^28^; Bolívar: San Juan Nepomuceno^1^; Boyacá: Puerto Boyacá^1^; Caquetá: Albania^1^, Solano^18^; Cauca: Patía^28^; Cundinamarca: Agua de Dios^7^, El Colegio^1^; Córdoba: Montería^1^; Magdalena: Santa Marta^6^; Meta: La Macarena^1^, Puerto López^1,20^, Puerto Rico^1^, San Juan de Arama^1^; Quindío: Quimbaya^15,28^; Santander: Floridablanca^1^; Valle del Cauca: Buenaventura^1^, Dagua^20^, Palmira^12^; Santiago de Cali^1,12,28^, Vichada: Cumaribo^17,28^.

***Euglossa* *(Euglossa) tridentata* Moure, 1970**

**Country records**: Antioquia: La Pintada^28^, Medellín^1^, San Francisco^17^, Sonsón^17^; Arauca: Arauca^28^; Bolívar: San Jacinto^1^, San Juán Nepomuceno^1^; Boyacá: Puerto Boyacá^1^, Santa María^1^; Caldas: La Dorada^1,28^, Viterbo^28^; Caquetá: El Paujil^1^, San Vicente del Caguán^1^; Casanare: Hato Corozal^1^, Orocué^1^, San Luís de Palenque^1^, Trinidad^1^; Chocó: Bahía Solano^1,28^, Lloró^15,28^, Nuquí^1^, Quibdó^1^; Magdalena: Santa Marta^1,15,28^; Meta: Acacías^1,17,5^, Cubarral^1^, Cumaral^1^, Guamal^1^, Mesetas^1^, Puerto López^2^, Restrepo^1^, San Juan de Arama^1^, Villavicencio^1,28^; Nariño: Tumaco^1^; Norte de Santander: Cúcuta^28^; Quindío: Circasia^15^, Filandia^28^, Quimbaya^15,28^; Risaralda: Pueblo Rico^2^, Santuario^1^; Tolima: San Sebastián de Mariquita^1^; Valle del Cauca: Armenia^¹⁵^, Buenaventura^1,15,28^, Dagua^20^, Palmira^1,3^,Quimbaya^15^ ; Santiago Cali^20^ , Yotoco^15^.

***Euglossa* *(Euglossa)* *variabilis* Friese, 1899**

**Country records**: Antioquia: Bello^28^, Envigado^28^, Itagüí^28^, Medellín^1,5,28,20^, San Francisco^17^, Sonsón^17^; Arauca: Arauca^28^; Boyacá: Otanche^7^; Caldas: Chinchiná^28^, Manizales^12^, Viterbo^28^; Cauca: Santander de Quilichao^12^; Cundinamarca: Puerto Salgar^1^; Huila: Rivera^1^; Magdalena: Santa Marta^15,6,28^; Meta: San Juan de Arama^1^, Villavicencio^1,28^; Putumayo: Mocoa^28^; Quindío: Circasia^15,28^, Quimbaya^15,28^; Risaralda: Santuario^1^; Tolima: Dolores^1^, Ibagué^5^; Valle del Cauca: Buenaventura^15^,Calima^12^, Dagua^20^, Palmira^12^ ; Santiago de Cali^28^.

***Euglossa* *(Euglossa) villosiventris* Moure, 1968**

**Country records**: Antioquia: Sonsón^17^; Boyacá: Otanche^7^; Chocó: Bahía Solano^28^, Lloró^15,28^; Cundinamarca: Nilo^7^; Nariño: Barbacoas^1^; Valle del Cauca: Buenaventura^1,15,28^.

**Subgenus *Euglossella* Moure, 1967**

***Euglossa* *(Euglossella)* *azurea* Ducke, 1902***

**Country records**: Casanare: Villanueva^28^; Meta: Puerto López^2^

***Euglossa* *(Euglossella)* *celiae* Hinojosa-Díaz & Engel, 2014***

**Country records**: Amazonas: Leticia† ^(Hinojosa-Díaz & Engel, 2014)^; Meta: Guamal^1^.

***Euglossa* *(Euglossella) cyanura* Cockerell, 1917**

**Country records**: Antioquia: Amalfi^28^, Yolombó^28^; Cundinamarca: La Vega^1^.

***Euglossa* *(Euglossella)* *decorata* Smith, 1874***

**Country records**: Amazonas: La Pedrera^1^, Puerto Santander^1^; Caquetá: Florencia^1^; Meta: Acacías^1,17^, La Macarena^20^, Restrepo^1^, San Juan de Arama^17^, San Martín^1^, Villavicencio^1^; Putumayo: Mocoa^1,28^.

***Euglossa* *(Euglossella)* *granti* Cheesman, 1929**

**Country records**: Cauca: Guapi†^(Cheesman, 1929)^.

***Euglossa* *(Euglossella) singularis* Mocsáry, 1899**

**Country records**: Amazonas: Puerto Santander^1^

***Euglossa* *(Euglossella)* *subandina* Hinojosa-Díaz & Engel, 2014***

**Country records**: Guaviare: San José del Guaviare^1^

***Euglossa* *(Euglossella)* *viridis* (Perty, 1833)**

**Country records**: Amazonas: Puerto Santander^1^; Putumayo: Puerto Leguízamo^1^; Vaupés: Yavaraté^1^.

**Subgenus *Eurhytisma* Engel, 2021**

***Euglossa* *(Eurhytisma)* *dodsoni* Moure, 1965**

**Country records**: Antioquia: San Francisco^17^; Boyacá: Otanche^7^; Chocó: Bahía Solano^28^, Lloró^28^, Medio San Juan^28^, Nuquí^1^, Quibdó^1^; Nariño: Barbacoas^1^, Tumaco^1^; Quindío: Quimbaya^15^.

**Subgenus *Glossura* Cockerell, 1917**

***Euglossa* *(Glossura)* *allosticta* Moure, 1969**

**Country records**: Antioquia: Guadalupe^28^, San Francisco^15,17^, Sonsón^17^, Yolombó^13^; Arauca: Arauca^28^; Bolívar: San Jacinto^1^, San Juan Nepomuceno^1^; Boyacá: Otanche^7^, Santa María^1^; Caldas: La Dorada^28^, Viterbo^28^; Caquetá: El Paujil^1^; Casanare: Orocué^1^, San Luís de Palenque^1^; Cundinamarca: Agua de Dios^7^, Silvania^15^; Córdoba: Ayapel^1,20^; Meta: Cumaral^1^, Guamal^1^, La Macarena^1,5^, Mesetas^1^, Puerto Rico^1^, Restrepo^1^, San Juan de Arama^1^, Villavicencio^1^; Putumayo: Puerto Asís^1,5^; Quindío: Armenia^15^, Circasia^15^, Quimbaya^15,28^; Santander: Floridablanca^1^, Socorro^7^; Tolima: Cunday^1^, Dolores^1^; Valle del Cauca: Buenaventura^1^.

***Euglossa* *(Glossura)* *asarophora* Moure & Sakagami, 1969**

**Country records**: Chocó: Bahía Solano^28^, Lloró^15,28^, Medio San Juan^28^, Nuquí^1^, Quibdó^15^; Valle del Cauca: Buenaventura^1,15,5,20^.

***Euglossa* *(Glossura)* *bazinga* Nemésio & Ferrari, 2012***

**Country records**: Amazonas: Leticia^17^.

***Euglossa* *(Glossura)* *chalybeata* Friese, 1925**

**Country records**: Bolívar: Zambrano^17^; Boyacá: Otanche^7^; Caquetá: Florencia^1^, San Vicente del Caguán^1^, Solano^17,28^, Valparaíso^1^; Chocó: Bahía Solano^1^, Quibdó^15^; Magdalena: Santa Marta^1^; Meta: Acacías^1^, Guamal^1^, La Macarena^1,5^, Puerto Rico^1^, Restrepo^1^, San Juan de Arama^1^; Nariño: Barbacoas^1^; Putumayo: Mocoa^28^; Valle del Cauca: Buenaventura^1^; Vaupés: Mitú^1^.

***Euglossa* *(Glossura)* *flammea* Moure, 1969**

**Country records**: Antioquia: Amalfi^28^, Yolombó^13^; Boyacá: Otanche^7^; Caldas: Samaná^28^; Chocó: Bahía Solano^1^, Quibdó^15^; Santander: El Carmen de Chucurí^13^; Valle del Cauca: Buenaventura^1,11,15,20^, Santiago de Cali^11^.

***Euglossa* *(Glossura)* *ignita* Smith, 1874**

**Country records**: Amazonas: La Pedrera^1^, Leticia^1,2,5,7^, Puerto Ñarino^1^ ; Puerto Santander^1^, ; Antioquia: Amalfi^28^, Murindo^¹^, San Francisco^17^, Sonsón^17^, Valparaíso^20^, Yolombó^13,28^; Boyacá: Maripí^1^, Otanche^7^, Puerto Boyacá^1,5^; Caldas: Norcasia^17,28^; Caquetá: El Paujil^1^, Florencia^1^, Miraflores^17^, San José del Fragüa^1^, San Vicente del Caguán^1^, Solano^17,28^; Casanare: Trinidad^1^, Villanueva^1^; Cauca: Guapi^1,11^; Chocó: Acandí^1^, Bahía Solano^1,11^, Medio San Juan^28^, Murindó^1^, Nuquí^1^, Quibdó^1,15,28^; Cundinamarca: Nilo^7^, Puerto Salgar^1^; Guaviare: San José del Guaviare^1^; Magdalena: Santa Marta^15,28^; Meta: Acacías^17^, Fuente de Oro^5^, La Macarena^1^, Mesetas^1^, Puerto López^1,2^, Puerto Rico^1^, Restrepo^1^, San Juan de Arama^1^, San Martín^2^, Villavicencio^1^; Nariño: Barbacoas^1^, Francisco Pizarro^1^, Tumaco^1^; Putumayo: Puerto Leguízamo^17^;  Risaralda: Pueblo Rico^2^; Valle del Cauca: Buenaventura^1,15,5,20^,Obando^15^; Vaupés: Mitú^1^, Yavaraté^1^; Vichada: Cumaribo^1,17,28^.

***Euglossa* *(Glossura)* *imperialis* Cockerell, 1922**

**Country records**: Amazonas: La Pedrera^1^, Leticia^1,17,28^, Puerto Ñarino^1^; Antioquia: Amalfi^13,28,20^, Cocorná^5^, Medellín^13,28^, San Francisco^1,17^, Sonsón^17^, Yolombó^13,28^; Boyacá: Puerto Boyacá^1^, Santa María^1^; Caldas: La Dorada^28^, Norcasia^13^; Caquetá: El Paujil^1^, San Vicente del Caguán^1^, Solano^17,28^, Valparaíso^1^; Casanare: Villanueva^1^; Cesar: Valledupar^1,6,28^; Chocó: Bahía Solano^1,11^, Nuquí^1^, Quibdó^1^; Córdoba: Ayapel^1,5^, Buenavista^1^, Montería^1^; Magdalena: El Retén^1^, Santa Marta^1,17,6,28^; Meta: Acacías^1^, Cumaral^1^, Guamal^1^, La Macarena^1,5^, Mesetas^1^, Puerto López^2^, Puerto Rico^1^, San Juan de Arama^1^, Uribe^17,28^, Villavicencio^1,3^; Nariño: Francisco Pizarro^1^, Putumayo: Mocoa^1^; Risaralda: Pueblo Rico^5^, Santuario^1^; Tolima: San Sebastián de Mariquita^1^; Valle del Cauca: Buenaventura^1,11,20^, El Cairo^1^, Palmira^12^, Yotoco^1^; Vichada: Cumaribo^17^.

***Euglossa* *(Glossura)* *inflata* Roubik, 2004***

**Country records**: Vaupés: Papunahua^1^, Yavaraté^1^.

***Euglossa* *(Glossura)* *intersecta* Latreille, 1817**

**Country records**: Amazonas: Leticia^1,17,2,5,7,28^, Puerto Santander^1^, Puerto Ñarino^1^, Tarapacá^1^; Antioquia: Valparaíso^20^; Boyacá: Puerto Boyacá^28^; Caquetá: El Paujil^1^, Solano^17,28^, Valparaíso^1^; Casanare: Villanueva^1,28^; Cauca: Piamonte^1^, Santa Rosa^28^; Meta: Acacías^17^, La Macarena^1,5^, Puerto López^2,5^, Puerto Rico^1^, Villavicencio^1^; Putumayo: Mocoa^1,28^, Puerto Asís^1,5^; Vaupés: Mitú^1^; Vichada: Cumaribo^1,17,28^.

***Euglossa* *(Glossura)* *lugubris* Roubik, 2004**

**Country records**: Amazonas: Leticia^17,2^, Puerto Santander^1^; Bolívar: El Carmen de Bolívar^17^, Zambrano^17^; Caquetá: Solano^17^; Cesar: Valledupar^17^; Meta: Puerto López^2^; Vaupés: Mitú^1^, Taraira^17^; Vichada: Cumaribo^17,2^.

***Euglossa* *(Glossura)* *natesi* Parra, Ospina-Torres & Ramírez, 2006**

**Country records**: Antioquia: Urrao^28^; Nariño: Barbacoas†^1^; Valle del Cauca: Buenaventura^1,20.^

***Euglossa* *(Glossura)* *occidentalis* Roubik, 2004**

**Country records**: Amazonas: Leticia^17,28^; Caquetá: Valparaíso^1^; Putumayo: Mocoa^1^; Vaupés: Mitú^1^, Taraira^1^.

***Euglossa* *(Glossura)* *orellana* Roubik, 2004**

**Country records**: Amazonas: Cumaribo^¹^,La Pedrera^1^, Leticia^18,28^, Puerto Ñarino^1^; Antioquia: San Francisco^17^, Sonsón^17^; Boyacá: Puerto Boyacá^1^, Santa María^1,2^; Caquetá: El Paujil^1^, Solano^17,28^, Valparaíso^1^; Meta: Acacías^1^, Cubarral^1^, Guamal^1^, Puerto Rico^1^, Restrepo^1^, San Juan de Arama^1^, Villavicencio^1^; Nariño: Barbacoas^1^; Norte de Santander: Herrán^17,28^; Putumayo: Mocoa^1^, Puerto Leguízamo^1^; Vaupés: Mitú^1^, Yavaraté^1^; Vichada: Cumaribo^1^.

***Euglossa* *(Glossura)* *piliventris* Guérin-Méneville, 1845**

**Country records**: Amazonas: Leticia^1,17^, Puerto Santander^1^; Vichada: Cumaribo^1,5^.

***Euglossa* *(Glossura)* *rufipes* Rasmussen & Skov, 2006***

**Country records:** Caquetá: San José del Fragua^17^.

***Euglossa* *(Glossura)* *rugilabris* Moure, 1967**

**Country records**: Amazonas: Leticia^7,28^; Caquetá: Solano^17^; Guainía: Inírida^5^.

***Euglossa* *(Glossura)* *viridifrons* Dressler, 1982**

**Country records**: Amazonas: Leticia^1,28^, Puerto Ñarino^1^; Caquetá: Solano^28^; Meta: La Macarena^1^; Putumayo: Puerto Leguízamo^1^.

**Subgenus *Glossurella* Dressler, 1982**

***Euglossa* *(Glossurella)* *augaspis* Dressler, 1982**

**Country records**: Amazonas: Leticia^1,17,28^; Caquetá: El Paujil^1^, Florencia^1^, Solano^17,28^; Chocó: Bahía Solano^1^; Meta: Mesetas^1^; Putumayo: Mocoa^28^; Tolima: San Sebastián de Mariquita^1^; Vichada: Cumaribo^17,28^.

***Euglossa* *(Glossurella)* *bursigera* Moure, 1970**

**Country records**: Caldas: La Dorada^28^; Chocó: Bahía Solano^1^, Lloró^28^, Nuquí^1,13^; Valle del Cauca: Buenaventura^1^.

***Euglossa* *(Glossurella)* *embera* Hinojosa-Díaz, Nemésio & Engel, 2012***

**Country records**: Antioquia: San Francisco^17^, Sonsón^17^; Chocó: Bahía Solano^1^, Nuquí^1^; Valle del Cauca: Buenaventura†^1,20^.

***Euglossa* *(Glossurella)* *laevicincta* Dressler, 1982**

**Country records**: Amazonas: Leticia^28^, Puerto Ñarino^1^; Boyacá: Santa María^1^; Chocó: Bahía Solano^1,28^; Cundinamarca: Nilo^7^; Meta: Villavicencio^1^; Tolima: San Sebastián de Mariquita^1^; Valle del Cauca: Buenaventura^1^; Vichada: Cumaribo^17^.

***Euglossa* *(Glossurella)* *prasina* Dressler, 1982**

**Country records**: Amazonas: La Pedrera^1^, Leticia^1,7,28^; Boyacá: Otanche^7^; Meta: Acacias^1^.

***Euglossa* *(Glossurella)* *turbinifex* Dressler, 1978**

**Country records**: Antioquia: Sonsón^17^; Boyacá: Puerto Boyacá^1^, Santa María^1^; Casanare: Villanueva^1^; Chocó: Nuquí^1^; Guainía: Inírida^5^; Huila: La Argentina^1^; Nariño: Barbacoas^1^; Quindío: Circasia^15^; Tolima: Dolores^1^.

**Subgenus *Parisoglossa* Engel, 2021**

***Euglossa* *(Parisoglossa) macrorhyncha* Dressler, 1982**

**Country records**: Antioquia: Amalfi^13^, Anorí^28^, Medellín^13^, Murindo^1^; Yolombó^13^; Bolívar: San Jacinto^1^; Chocó: Bahía Solano^1^; Huila: Palermo^1^; Meta: Restrepo^1^; Nariño: Tumaco^1^; Quindío: Circasia^28^; Santander: El Carmen de Chucurí^13^; Valle del Cauca: Buenaventura^1^, Cali^28^, El Cerrito^15^; Santiago de Cali^28^; Vichada: Cumaribo^17,28^.

**Subgenus *Trachyglossa* Engel, 2021**

***Euglossa* *(Trachyglossa)* *crassipunctata* Moure, 1968**

**Country records**: Antioquia: Amalfi^1,13^, Anorí^13^, Bello^28^, Itagüí^28^, Medellín^28^, Puerto Triunfo^13^, San Francisco^17^, Sonsón^17^, Yolombó^13,28^; Caldas: La Dorada^28^, Viterbo^28^; Caquetá: Solano^17,28^; Casanare: Villanueva^1^; Chocó: Bahía Solano^1,11,28^, Lloró^28^, Quibdó^15,28^; Cundinamarca: Agua de Dios^7^, Nilo^7^, Sasaima^28^, Silvania^1^; Huila: Gigante^1^, Palermo^1^, Rivera^1^; Magdalena: Santa Marta^1,15,28^; Meta: Restrepo^1^, Villavicencio^1^; Putumayo: Mocoa^28^; Quindío: Filandia^28^, Quimbaya^15,28^; Santander: Socorro^7^;, Tolima: Armero^17,28^, Cunday^1^, Dolores^1^, Floridablanca^1^ ; Honda^17,28^, San Sebastián de Mariquita^1^; Valle del Cauca: Buenaventura^15,28^, El Cerrito^12^,  Yotoco ^¹⁵,28^; Vichada: Cumaribo^17^.

***Euglossa* *(Trachyglossa)* *parvula* Dressler, 1982**

**Country records**: Amazonas: Leticia^1,28^; Quindío: Quimbaya^15^.

***Euglossa* *(Trachyglossa)* *sapphirina* Moure, 1968**

**Country records**: Amazonas: Tarapacá^17^; Huila: Pital^1^, Rivera^1^; Valle del Cauca: Buenaventura^1^.

**Genus *Eulaema* Lepeletier, 1841**

**Subgenus *Apeulaema* Moure, 1950**

***Eulaema* *(Apeulaema)* *boliviensis* Friese, 1898**

**Country records**: Antioquia: San Francisco^13^; Boyacá: Maripí^1^; Cundinamarca: Caparrapí^1^, La Vega^7^, San Antonio del Tequendama^7^, San Francisco^1^; Huila: Algeciras^1^, Hobo^1^, La Argentina^1^; Meta: Villavicencio^28^; Nariño: Barbacoas^1^, Ricaurte^17,28^; Santander: Charalá^1^, El Carmen de Chucurí^13^, Socorro^7^; Valle del Cauca: Buenaventura^1^, Cali^28^.

***Eulaema* *(Apeulaema)* *cingulata* (Fabricius, 1804)**

**Country records**: Amazonas: Leticia^1,17,28^, Puerto Ñarino^1^; Antioquia: Amalfi^28,20^, Anorí^13^, Apartadó^4,28^, Bello^1,13,3,28^, Carepa^13^, Caucasia^13^, Copacabana^28^, Envigado^13,9,28^, Frontino^1^, Itagüí^28^, La Estrella^28^, Maceo^13^, Medellín^1,13,3,4,8,9,28^, Puerto Berrío^13^, Puerto Nare^13^, San Francisco^13,17^, San Roque^13^, Sonsón^1,17^, Támesis^28^, Yolombó^13,28^, Yondo^1^; Arauca: Arauquita^5^, Tame^1,28^; Bolívar: San Jacinto^1,17^, San Juán Nepomuceno^1^; Boyacá: Buenavista^¹^, Combita^1^, Miraflores^1^, Moniquirá^1^, Otanche^7^, Paipa^1,5^, Puerto Boyacá^¹,28^, San Mateo^1^, Santa María^1^, Soata^28^; Caldas: Aranzazu^15,28^, Chinchiná^28^, La Dorada^1,28^, Manizales^28^, Pácora^15^, Victoria^13^, Viterbo^28^; Caquetá: El Paujil^1,5^, Florencia^1,28^, San Vicente del Caguán^1^, Solano^17,28^; Casanare: Chámeza^1^, San Luís de Palenque^1^, Tauramena^2^, Trinidad^1^, Villanueva^1,28^, Yopal^7^; Cauca: Patía^1,28^, Popayán^1^; Cesar: , Pueblo Bello^28^, Valledupar^1,17,6,28^; Chocó: Bahía Solano^1,16,28^, Juradó^1^, Litoral del Bajo San Juan^1^, Lloro^28^, Nuquí^1,13^, Riosucio^1,5^; Sipi^¹⁷^;  Cundinamarca: Agua de Dios^7^, Bogotá, D.C.^1,7^, Caparrapí^1^, Carmen de Carupa^2^, Chaguaní^1,5^, El Colegio^1^, Fusagasugá^1^, Guayabetal^1,5^, La Mesa^1^, La Palma^2^, La Vega^7^, Madrid^1^, Medina^1^, Nilo^7^, Nimaima^1,2^, Nocaima^2,28^, Pacho^28^, Pandi^1^, San Antonio del Tequendama^2,7^, Silvania^1,15,5^, Sopó^1^, Tena^1,5^, Tibacuy^1^, Villeta^1^, Zipacon^1^, Zipaquirá^1^; Córdoba: Montería^1^; Huila: Acevedo^1,28^, Algeciras^1^, Neiva^1^, Pital^1^, Pitalito^1^, Rivera^1^, Teruel^1^; Magdalena: Aracataca^⁶^,Ciénaga^6^, Santa Marta^1,15,17,6,28^; Meta: Acacias^1^, Cumaral^1^, Fuente de Oro^1,5^, La Macarena^1,5,28,20^, Mesetas^1^, Puerto Concordia^1^, Puerto López^1,2^, Puerto Rico^1^, Restrepo^1^, San Juan de Arama^1^, San Martín^2,8^, Villavicencio^1,2,28^, Vistahermosa^18,5,28^; Nariño: Barbacoas^1^, Francisco Pizarro^1^, San Pedro de Cartago^1^, Tumaco^1^; Putumayo: Colon^28^, Mocoa^1,28^, Orito^2^, Puerto Leguízamo^1^; Quindío: Armenia^15,28^, Buenaventura^¹⁵^,   Buenavista^1,28^, Calarcá^15^, Circasia^15^, La Tebaida^15,28^, Quimbaya^15,6,7,28^; Risaralda: Dosquebradas^28^, Pereira^13,28^, Santa Rosa de Cabal^1^, Santuario^1,28^; Santander: Betulia^13^, Bucaramanga^28^, Charalá^1^, Cimitarra^13,17,9^, Floridablanca^28^, Giron^1,5^, Piedecuesta^13,28^, Socorro^7^, Suaita^5^; Tolima: Cunday^1^, Dolores^1^, Honda^17,28^, Ibagué^1,28^, Icononzo^1^, Villahermosa^1^; Valle del Cauca: Buenaventura^1,12,16,2,5,28^, Calima^12^, Cali^1,11,12,17,28,20^, Cartago^28^, Dagua^12,28^, El Cerrito^15,28^, El Dovio^1^, Guacarí^1,12^, Jamundí^12,28^, Palmira^1,12,3,28^, Santiago de Cali^1,11,12,16,20,28^; Yotoco^12,15,28^, Yumbo^28^; Vaupés: Mitú^1^; Vichada: Cumaribo^17,8,28^.

***Eulaema* *(Apeulaema)* *mocsaryi* (Friese, 1899)**

**Country records**: Amazonas: La Pedrera^1^, Leticia^1,17,5,28^, Puerto Ñarino^1^, Tarapacá^1^; Arauca: Arauca^28^; Caquetá: Miraflores^17^, San Vicente del Caguán^1^, Solano^17,28^; Casanare: Villanueva^1^; Cundinamarca: Caparrapí^1^, Fusagasugá^1^, Gachalá^1^; Meta: La Macarena^1^, Puerto Rico^1^, San Martín^28^, Uribe^18,28^, Villavicencio^1^; Putumayo: Mocoa^1,28^, Puerto Leguízamo^18^; Valle del Cauca: Buenaventura^1^, Cali^17^; Vaupés: Mitú^1^, Yavaraté^1^; Vichada: Cumaribo^5^, Puerto Carreño^28.^

***Eulaema* *(Apeulaema)* *nigrita* Lepeletier, 1841**

**Country records**: Antioquia: Amalfi^28^, Apartadó^4,28^, Bello^1,3^, Carepa^13^, Medellín^1,3,28^, San Francisco^17^, San Jerónimo^4,28^, Sonsón^17^, Yolombó^28^; Arauca: Arauca^28^; Bolívar: San Jacinto^1,17^, San Juan Nepomuceno^1^; Boyacá: Otanche^7^, Pajarito^5^, Santa María^1^; Caldas: Viterbo^28^; Caquetá: Florencia^1^, San José del Fragua^1^; Casanare: San Luís de Palenque^1^, Trinidad^1^, Villanueva^1^; Cauca: Caldono^12^, Patía^28^, Puerto Tejada^12^; Cesar: Valledupar^28^; Chocó: Bahía Solano^1^; Cundinamarca: Agua de Dios^7^, Anapoima^1^, Apulo^1,5^, Bogotá, D.C.^3^, Caparrapí^1^, El Colegio^1^, Fusagasugá^1^, La Vega^1^, Madrid^1^, Medina^1^, Nilo^1,7^, Nocaima^28^, Pandi^1^, Paratebueno^28^, Ricaurte^1^, San Antonio del Tequendama^7^, Silvania^1,15,5^, Tena^1^, Villeta^1,5^; Córdoba: Ayapel^1,5^; Huila: Acevedo^28^, La Plata^1^, Rivera^1^, San Agustín^28^, Teruel^1^, Villavieja^7,28^; Magdalena: Santa Marta^1^, Zona Bananera^1^; Meta: Acacías^1^, Cumaral^1^, Guamal^1^, La Macarena^1^, Puerto López^1,5^, Puerto Rico^1^, Restrepo^1^, San Juan de Arama^1^, Villavicencio^1,3^; Quindío: La Tebaida^15^, Quimbaya^15,28^; Risaralda: Pereira^1^, Santuario^1^; Santander: Cimitarra^1^, Floridablanca^1^, Socorro^7^; Tolima: Carmen de Apicalá^1^, Chaparral^1,5^, Cunday^1^, Dolores^1^, El Espinal^3^, Icononzo^1^, San Luís^1^, San Sebastián de Mariquita^1^; Valle del Cauca: Buenaventura¹⁶, Bugalagrande^¹⁶^, Caicedonia^¹²^, Cartago^28^, Dagua^¹²^, El Cairo¹, El Cerrito¹⁵, El Dovio¹, Ginebra¹⁵, Guacarí¹, Jamundí28, Palmira¹,¹²,¹⁶,³,28, Santiago de Cali¹,¹¹,¹²,¹⁶,28; Vichada: Cumaribo¹⁷, Puerto Carreño^28^.

***Eulaema* *(Apeulaema)* *peruviana* (Friese, 1903)**

**Country records**: Boyacá: Miraflores^1^; Meta: Villavicencio^1,28^.

***Eulaema* *(Apeulaema)* *polychroma* (Mocsáry, 1899)**

**Country records**: Amazonas: Leticia^8^; Antioquia: Bello^1,13,3,28^, Ciudad Bolívar^28^, Copacabana^28^, Envigado^13,28^, Fredonia^13^, Guarne^13^, Itagüí^28^, La Estrella^28^, Medellín^1,13,3,8,28^, Santa Fe de Antioquia^2^, Yolombó^28^; Bolívar: San Juan Nepomuceno^1^; Boyacá: Maripí^1^, Otanche^7^, Villa de Leyva^17,28^; Caldas: Chinchiná^28^, Manizales^28^; Casanare: Chámeza^1^; Cauca: Patía^28^; Cesar: Valledupar^17,6,28^; Cundinamarca: Anapoima^28^, Apulo^1^, Arbeláez^1^, Bogotá, D.C.^1,2^, Caparrapí^1^, Caqueza^1^, Choachí^1^, Fusagasugá^1^, Paime^1^, Quetame^1,28^, San Antonio del Tequendama^7^, Silvania^1^, Tena^1^; Huila: Algeciras^1^, Baraya^1^, Colombia^1^, Gigante^1^, Neiva^1^, Palestina^28^, Rivera^1^, San Agustín^28^, Villavieja^7^; Meta: San Juan de Arama^1^, Villavicencio^1,28^; Nariño: Pasto^1,3^; Quindío: Armenia^15^, Buenavista^28^, Calarcá^1,15^, Circasia^15^, La Tebaida^28^, Quimbaya^28^; Risaralda: Pueblo Rico^2^; Santander: Barichara^28^, Bucaramanga^1,3,5^, Cimitarra^28^, Enciso^7^, Piedecuesta^28^, Puente Nacional^28^, San Gil^28^, Socorro^7^, Suaita^28^, Tona^1^; Tolima: Libano^1^, San Sebastián de Mariquita^3^, Venadillo^1^; Valle del Cauca: Buga^12^, Caicedonia^12^, Calima^12^, Cali^1,12,17,28^, Cartago^12^, Dagua^12,20^, El Dovio^1^, Florida^12^, Guacarí^1,12^, Cali^¹,¹²,¹⁶,28^, Sevilla^12^, Yumbo^28^.

***Eulaema* *(Apeulaema)* *speciosa* (Mocsáry, 1897)**

**Country records**: Amazonas: Leticia^2^; Antioquia: Alejandría^13^, Amalfi^28^, Anorí^13^, Maceo^13^, Puerto Triunfo^13^, San Francisco^17^, San Luís^13^, San Roque^13^, Yarumal^9^, Yolombó^28^; Boyacá: Otanche^1,28^, Santa María^1^; Caquetá: San Vicente del Caguán^1^; Chocó: Quibdó^1^, San José del Palmar^1^; Cundinamarca: Bogotá, D.C.^7^, Fusagasugá^1^, La Vega^1^; Magdalena: Santa Marta^1,6,28^; Meta: Acacías^1^, Cumaral^1^, La Macarena^1^, Villavicencio^1^; Nariño: Albán^1^, Barbacoas^1^, Los Andes^17^; Risaralda: Pueblo Rico^5,28^; Santander: Barichara^1^, El Carmen de Chucurí^13^, Floridablanca^1^; Tolima: Icononzo^1^; Valle del Cauca: Buenaventura^16^, Cali^1^.

**Subgenus *Eulaema* Lepeletier, 1841**

***Eulaema* *(Eulaema)* *bombiformis* (Packard, 1869)**

**Country records**: Amazonas: El Encanto^5^, La Pedrera^1^, Leticia^1,17,7^, Puerto Santander^1^, Puerto Ñarino^1^; Antioquia: San Francisco^17^, Sonsón^17^; Bolívar: San Juan Nepomuceno^1^; Boyacá: Miraflores^1^; Caquetá: San Vicente del Caguán^1^, Solano^1,17,28^; Casanare: San Luís de Palenque^1^, Villanueva^1^; Cauca: Guapi^1^; Chocó: Bahía Solano^1,16,28^, Lloró^28^, Nuquí^1^, Quibdó^28^, San José del Palmar^1^; Cundinamarca: Apulo^1^, Bogotá, D.C.^1^, La Mesa^1^; Guaviare: San José del Guaviare^17,28^; Meta: Acacías^1^, Cumaral^1^, La Macarena^1^,Puerto Concordia^1^, Puerto Rico^1^, Restrepo^1^, Villavicencio^1^; Nariño: Francisco Pizarro^1^, Tumaco^1^; Putumayo: Mocoa^17,28^, Puerto Asís^1^; Quindío: Circasia^28^; Santander: Floridablanca^28^; Tolima: Honda^28^; Valle del Cauca: Buenaventura^1,15^; Vaupés: Mitú^1^; Vichada: Cumaribo^17,28^, Puerto Carreño^28^.

***Eulaema* *(Eulaema)* cf. *bomboides***

**Country records**: Santander: Floridablanca^28^

Note: Ramírez et al. (2002) reported this species from the Amazonian and Pacific regions of Colombia. However, the studied specimens were not cited, and its presence in the country could not be confirmed, as it was not found by any of the authors in the collections examined in the present study.

***Eulaema* *(Eulaema)* *chocoana* Ospina-Torres & Sandino-Franco, 1997**

**Country records**: Chocó: Lloró^28^; Nariño: Barbacoas†^5^; Valle del Cauca: Buenaventura^1,20^.

***Eulaema* *(Eulaema)* *leucopyga* Friese, 1898**

**Country records**: Chocó: Acandí^2^, Bahía Solano^1^; Nariño: Barbacoas^1^; Santander: El Carmen de Chucurí^13^, Puerto Parra^3^; Valle del Cauca: Buenaventura^1^.

***Eulaema* *(Eulaema)* *luteola* Moure, 1967**

**Country records**: Antioquia: Medellín^13^; Magdalena: Aracataca^5^; Santander: Simacota^20^; Cauca: Miranda†

***Eulaema* *(Eulaema)* *meriana* (Olivier, 1789)**

**Country records**: Amazonas: La Pedrera^1^, Leticia^1,17,2,5^, Puerto Santander^13^, Puerto Nariño^1^, Tarapacá^1^; Antioquia: Amalfi^1,28,20^, Angostura^5^, Bello^28^, Briceno^13^, Carepa^13^, Caucasia^13^, Envigado^9^, Medellín^13,28^, Puerto Berrío^13^, Remedios^13^, San Francisco^1,17^, San Luís^13^, Sonsón^17^, Yolombó^28^; Arauca: Arauca^28^; Bolívar: El Carmen de Bolívar^17^, San Jacinto^1,17^, San Juan Nepomuceno^1,17^; Boyacá: Maripí^1^, Miraflores^1^, Otanche^7^, Puerto Boyacá^1,5^, San Luis de Gaceno^1^, Santa María^1^; Caldas: Chinchiná^28^, La Dorada^1,28^, Victoria^1^, Viterbo^28^; Caquetá: El Paujil^1^, Florencia^1,28^, San José del Fragua^1^, Solano^17,28^; Casanare: San Luís de Palenque^1^, Trinidad^1^, Villanueva^1^, Yopal^28^; Chocó: Bahía Solano^11,16,28^, Lloró^15,28^, Medio San Juan^28^, Nuquí^1^, Quibdó^28^, San José del Palmar^1^; Cundinamarca: Agua de Dios^7^, Apulo^1^, Caparrapí^1^, Fusagasugá^28^, La Mesa^1^, La Vega^1^, Nilo^7^, San Antonio del Tequendama^7^, Sasaima^1^, Silvania^15^, Tena^1^, Zipacón^1^; Córdoba: Ayapel^1,5^, Montería^1^; Guaviare: Calamar^1^, San José del Guaviare^1^; Huila: Gigante^1^, La Argentina^1^, Palestina^1^; Magdalena: Ciénaga^1,28^, Santa Marta^1,28^; Meta: Acacías^1^, Cubarral^1^, Cumaral^1^, Guamal^1^, La Macarena^1^, Puerto Rico^1^, Restrepo^1^, San Juan de Arama^1^, San Martín^1,28^, Uribe^20^, Villavicencio^1,2,28^; Nariño: Barbacoas^1^, Cuaspud^1^, Francisco Pizarro^1^, Tumaco^1^; Putumayo: Mocoa^1,28^, Puerto Leguízamo^1^; Quindío: Armenia^15,28^, Buenavista^15^, Calarcá^15,28^, Circasia^15^, La Tebaida^28^, Quimbaya^15,28^, Salento^15^; Risaralda: Mistrató^5^; Santander: Cimitarra^1^, Simacota^20^, Socorro^7^, Tona^1^; Sucre: Ovejas^17^; Tolima: Cunday^1^, Dolores^1^, Honda^17,20^, Icononzo^1^, Melgar^1^, San Sebastián de Mariquita^1^; Valle del Cauca: Buenaventura^1,15,16^, Calima^12^, Cali^1,17,28^, El Cairo^1^, El Cerrito^15^, El Dovio^1^, Guadalajara de Buga^15^, Jamundí^28^, Palmira^1,12,16^, Cali^¹,¹⁶,28^ ; Yotoco^15^; Vaupés: Taraira^17,28^; Vichada: Cumaribo^1,17^, Puerto Carreño^28^.

***Eulaema* *(Eulaema)* *parapolyzona* Oliveira, 2006**

**Country records**: Cauca: Piamonte^1^

***Eulaema* *(Eulaema)* *polyzona* (Mocsáry, 1897)**

**Country records**: Amazonas: Puerto Ñarino^1^; Cundinamarca: Tena^1^, Bogotá D.C.^1^.

***Eulaema* *(Eulaema)* cf. *seabrai***

**Country records**: Cauca: Piendamó^12^

***Eulaema* *(Eulaema)* *sororia* Dressler & Ospina-Torres, 1997**

**Country records**: Chocó: Bahía Solano^28^, Lloró^28^, Nuquí^1^, San José del Palmar^1^; Nariño: Barbacoas^1^, Tumaco^1^; Valle del Cauca: Buenaventura^1^.

**Genus *Exaerete* Hoffmannsegg, 1817**

***Exaerete* *dentata* (Linnaeus, 1758)**

**Country records**: Antioquia: Carmen de Viboral^14^; Boyacá: Otanche^1^; Casanare: Hato Corozal^5^, Yopal^11^; Meta: San Juan de Arama^1^; Tolima: Cunday^1,28^.

***Exaerete* *frontalis* (Guérin-Méneville, 1845)**

**Country records**: Amazonas: La Chorrera^13^, Leticia^17^, Puerto Ñarino^1^; Antioquia: Envigado^28^, Puerto Nare^13,28^, San Francisco^17^, Sonsón^17^; Bolívar: San Jacinto^1,17^, San Juan Nepomuceno^1^; Boyacá: Puerto Boyacá^28^, Santa María^1,2^; Caldas: Chinchiná^28^, La Dorada^1^, Manizales^28^, Viterbo^28^; Caquetá: Florencia^¹^, San Vicente Del Caguán^¹^, Solano^¹⁷,28^; Casanare: San Luís de Palenque^1^, Trinidad^1^, Villanueva^1^, Yopal^28^; Cauca: Mercaderes^12^; Chocó: Bahía Solano^1,28^, Lloró^28^; Cundinamarca: Agua de Dios^7^, Medina^1^, Nilo^7^, Viota^1,5^; Guaviare: San José del Guaviare^1^; Huila: Gigante^1^; Magdalena: Santa Marta^1,15,28^; Meta: Acacias^1^, Guamal^1^, La Macarena^1^, Restrepo^1^, San Juan de Arama^1^, San Martín^2^, Villavicencio^1,5^; Quindío: Quimbaya^15,28^, Santander: Socorro^7^; Tolima: Cunday^1^; Valle del Cauca: Buenaventura^1,28^, El Cairo^1^, El Cerrito^15^, Guacarí^12^, Palmira^1,12,3^, Obando^28^,  Restrepo^12^, Roldanillo^15^, Cali^11^; Vaupés: Mitú^1,20^, Taraira^1^; Vichada: Cumaribo^1,17,5^.

***Exaerete* *smaragdina* (Guérin-Méneville, 1845)**

**Country records**: Amazonas: Leticia^1,17^, Puerto Santander^2^, Puerto Ñarino^1^; Antioquia: Amalfi^14,28^, Andes^13^, Bello^14,28^, Carepa^13^, Caucasia^13^, Envigado^9,28^, Girardota^14^, Medellín^13,14,28^, Puerto Berrío^14^, Salgar^13^, San Francisco^17^, San Jerónimo^13^, Santa Fe de Antioquia^14^, Sonsón^17^, Támesis^13,14^, Valparaíso^5^, Yolombó^28^; Arauca: Arauca^28^; Bolívar: El Carmen de Bolívar^1,17^, San Jacinto^1,17^, San Juan Nepomuceno^1,17^; Boyacá: Macanal^1^, Puerto Boyacá^1,13,5,28^, San Luis de Gaceno^1^, Santa María^1,2^, Sogamoso^5^; Caldas: Aguadas^15,28^, Aranzazu^15,28^, Chinchiná^28^, La Dorada^1,28^, Pácora^15,28^, Viterbo^28^; Caquetá: Florencia^1^, San Vicente del Caguán^1^, Solano^17,28^, Valparaíso^1^; Casanare: Orocué^1^, San Luís de Palenque^1^, Trinidad^1^, Villanueva^1^, Yopal^1,28^; Cauca: Guapi^1^, Miranda^12^; Cesar: Valledupar^1,17,6,28^; Chocó: Acandí^1^; Cundinamarca: Agua de Dios^7^, El Colegio^1,5^, Guaduas^1^, La Mesa^1^, Nilo^1,7^, Nocaima^28^, Paratebueno^28^, Ricaurte^28^, San Antonio del Tequendama^28^, Viotá^1,5^, Zipacón^1^, Zipaquirá^1^; Córdoba: Ayapel^1,5^, Tierralta^1^; Huila: La Argentina^1^, Neiva^18^, Palermo^1^, Pitalito^6,28^, Rivera^1^; Magdalena: Santa Marta^1,15,17,6,28^; Meta: Acacías^1,17,5^, Cubarral^1^, Granada^1^, Guamal^1^, La Macarena^1,5^, Mesetas^1^, Puerto Gaitán^17,28^, Puerto López^2^, Restrepo^1,28^, San Juan de Arama^1^, San Martín^1,2^, Uribe^17,28^, Villavicencio^1,28^; Nariño: Francisco Pizarro^1^, Los Andes^14^, Tumaco^1^; Norte de Santander: Cucuta^28^; Putumayo: Mocoa^28^; Quindío: Filandia^28^, La Tebaida^15^, Quimbaya^13,15,28^; Risaralda: Pereira^12,17^, Pueblo Rico^2^; Santander: Cimitarra^1^, Piedecuesta^1,20^, Puerto Parra^14^, San Gil^1^, Socorro^7^; Tolima: Flandes^1^, Ibagué^1^, Piedras^3^; Valle del Cauca: Buenaventura^1,15,28^, Cali^28^, Cartago^14,28^, Dagua^1,12,20^, El Cairo^1^, El Cerrito^12^, El Dovio^1^, Buga^15^, Jamundí^12^, La Unión^28^, Palmira^1,12^, Cali^5,11^, Yumbo^12,28^; Vaupés: Mitú^1^; Vichada: Cumaribo^1,17,5,28^.

***Exaerete* *trochanterica* (Frise, 1900)**

**Country records**: Caquetá: Solano^28^.

**Meliponini Lepeletier, 1836**

**Genus *Aparatrigona* Moure, 1951**

***Aparatrigona* *impunctata* (Ducke, 1916)**

**Country records**: Amazonas: Leticia^1^; Caquetá: Florencia^1^; Cauca: Piamonte^11^; Guainía: La Guadalupe^11^, San Felipe^11^; Meta: Granada^1^.

***Aparatrigona* *isopterophila* (Schwarz, 1934)**

**Country records**: Antioquia: Amalfi^28^, Chigorodó^1^, Cocorná^13^, Sonsón^17^, Yolombó^1,28^; Boyacá: Muzo^19^; Caldas: Chinchiná^1^; Cundinamarca: Paime^1^; Nariño: Tumaco^1^; Sucre: Coloso^¹^.

**Genus *Celetrigona* Moure, 1950**

***Celetrigona* *longicornis* (Friese, 1903)**

**Country records**: Caquetá (Pedro & Camargo, 2009).

**Genus *Cephalotrigona* Schwarz, 1940**

***Cephalotrigona* *capitata* (Smith, 1854)**

**Country records**: Amazonas: Leticia^1,2,17^; Boyacá: San Luis de Gaceno^1^; Caquetá: Florencia^11^, San José del Fragua^5^; Cauca: Piamonte^1,11^; Cundinamarca: Medina^1,28^; Guaviare: Calamar^1^; Meta: Cumaral^1^, Restrepo^1^, Villavicencio^1^, Vistahermosa^1^; Putumayo: Puerto Asís^1^.

***Cephalotrigona* *femorata* (Smith, 1854)**

**Country records**: Amazonas: Leticia^1^; Caquetá: Florencia^1^; Cundinamarca: Medina^1^; Guaviare: San José del Guaviare^1^; Meta: Puerto Rico^1^, Restrepo^1^, Villavicencio^19^.

***Cephalotrigona* *zexmeniae* (Cockerell, 1912)**

**Country records**: Antioquia: Amalfi^14,28^, Porce^14^, Puerto Nare^13^, San Rafael^1^, San Roque^1^, Sonsón^17^, Yolombó^13,28^; Bolívar: San Jacinto^1^, San Juan Nepomuceno^1^; Caldas: Cañaveral^14^, La Dorada^1^, Támesis^1^, Victoria^13^; Cesar: Valledupar^1,17,3,6,28^; Chocó: Juradó^14^, Riosucio^17^; Cundinamarca: Guaduas^1^, La Vega^1^, Medina^1^, Paime^1^, Puerto Salgar^1^; Córdoba: Tierralta^1^; Magdalena: Santa Marta^6,28^, Zona Bananera^19^; Risaralda: Pereira^1,13^; Santander: Cimitarra^1^; Sucre: Coloso^¹^ Tolima: Armero^2^, Venadillo^1^.

**Genus *Duckeola* Moure, 1944**

***Duckeola* *ghilianii* (Spinola, 1853)**

**Country records**: Amazonas: Leticia^1^, Tarapacá^17^; Cauca: Piamonte^1,16^; Guainía: San Felipe^11^; Meta: La Macarena^1^.

***Duckeola* *pavani* (Moure, 1963)***

**Country records**: Cauca: Piamonte^11^; Guainía: La Guadalupe^11^, San Felipe^1^.

**Genus *Exochotrigona* Engel 2022**

***Exochotrigona* *crispula* (Pedro & Camargo, 2009)***

**Country records**: Antioquia: Puerto Triunfo†^(Pedro & Camargo, 2009)^, San Francisco^17^.

**Genus *Frieseomelitta* Ihering, 1912**

***Frieseomelitta* *paupera* (Provancher, 1888)**

**Country records**: Antioquia: Amalfi^13^, Fredonia^13^, San Francisco^1,17^, San Roque^1^, Santa Fé de Antioquia^1,2^, Tarso^13^, Yolombó^1,28^; Atlántico: Barranquilla^1^, Puerto Colombia^1,28^, Tubará^1^; Bolívar: Arjona^1^, Cartagena^1^, San Juan Nepomuceno^1^; Turbaco^¹^; Boyacá: Puerto Boyacá^1^; Caldas: Aguadas^15^, Anserma^1^, Filadelfia^1^, La Palma^1^, Victoria^1,3^; Cauca: Popayán†^(Friese, 1901)^.Cesar: Pueblo Bello^28^; Cundinamarca: Anapoima^1^, Apulo^1^, Beltrán^1^, El Colegio^1^, Fusagasugá^1^, Girardot^28^, Guaduas^1^, La Mesa^1^,  La Palma^¹^, NILO^5^, Puerto Salgar^1^, Tibacuy^1^; Córdoba: Montería^1^; Guainía: San Felipe^1^; Huila: Baraya^1^, Garzón^1^, Neiva^17^, Palermo^1^, Rivera^1^, Villavieja^1,7,28^; Magdalena: Ciénaga^6^, Santa Marta^1,28,20^, Zona Bananera^3,6,19^;Risaralda: Pereira^1^; Santander: Cimitarra^1,28^, Floridablanca^1^, Piedecuesta^5^, Puerto Parra^1^, Socorro^1^; Sucre: Coloso^¹^, Galeras^28^, Ricaurte^1^, Sampués^1^, San Onofre^13^, Sincelejo^1^; Tolima: Armero^3^, Chaparral^1^, El Espinal^3^, Honda^1^, Ibagué^10^, Melgar^1,2^, San Luís^10^.

***Frieseomelitta* *portoi* (Friese, 1900)***

**Country records:** Cauca: Piamonte^11^**.**

**Genus *Geotrigona* Moure, 1943**

***Geotrigona* *fulvohirta* (Friese, 1900)**

**Country records**: Amazonas: Leticia^1^; Boyacá: Miraflores^1^, Pajarito^5^, Santa María^1,2^; Caquetá: San Vicente del Caguán^1^; Meta: Uribe^1^, Villavicencio^1^.

***Geotrigona* *joearroyoi* Gonzalez & Engel, 2012***

**Country records**: Magdalena: Santa Marta^1,20^†.

***Geotrigona* *kaba* Gonzalez & Sepúlveda, 2007**

**Country records:** Antioquia: Amalfi^13^†, San Francisco^17^, Sonsón^17^, Yolombó^13^; Boyacá: Muzo^19^, Pauna^1^; Caldas: Samaná^2^; Cundinamarca: Paime^1^, Sasaima^1^; Magdalena: Santa Marta^1^; Santander: Floridablanca^28^.

***Geotrigona* *subfulva* Camargo & Moure, 1996**

**Country records**: Meta: Villavicencio^5^.

***Geotrigona* *subgrisea* (Cockerell, 1920)**

**Country records**: Antioquia: Amalfi^28^, San Francisco^17^, Sonsón^17^, Yolombó^28^; Boyacá: Berbeo^1^, Buenavista^1^, Duitama^5^, Maripí^5^, Miraflores^1^, Muzo^19^; Caldas: Chinchiná^1^; Cesar: Agustín Codazzi^19^, Becerril^19^; Cundinamarca: Bituima^1^, La Vega^1^, Medina^1^, Paratebueno^1^, Quipile^1^, Ricaurte^1^, Villeta^1^; Guainía: Inírida^1^; Huila: Acevedo^1^, Gigante^1^, Palestina^1^, Pitalito^1^, San Agustín^28^; Sucre: Ricaurte^1^; Tolima: Fresno^1^, Melgar^1^.

***Geotrigona*** ***tellurica* Camargo & Moure, 1996***

**Country records**: Amazonas: El Encanto^28^, La Chorrera^19^; Antioquia: San Roque^1^, Yolombó^13^; Caquetá: San Vicente del Caguán^1^.

**Genus *Lestrimelitta* Friese, 1903**

***Lestrimelitta* *camposi* Melo, 2023***

**Country records**: Caquetá: La Macarena^1^, San Vicente del Caguán^1^; Cauca: Piamonte^11^.

***Lestrimelitta* *diminuta* Guevara, Gonzalez & Ospina, 2020***

**Country records**: Guainía: San Felipe†^1^.

***Lestrimelitta* *galvisi* Guevara, Gonzalez & Ospina, 2020***

**Country records:** Nariño: El Charco†^1^.

***Lestrimelitta* *glabrata* Camargo & Moure, 1990***

**Country records**: Caquetá: Florencia^1^; Meta: Villavicencio^1^; Putumayo: Mocoa^1^.

***Lestrimelitta* cf. *guyanensis***

**Country records**: Magdalena: Santa Marta^1^.

***Lestrimelitta*** ***huilensis* Gonzalez & Griswold, 2012***

**Country records**: Antioquia: Santa Fé de Antioquia^2^; Huila: Neiva^17^, Palermo^1^, Rivera^1,5^†; Norte de Santander: Convención^1^; Sucre: Coloso^¹^; Tolima: Coello^2^.

***Lestrimelitta* *opita* Gonzalez & Griswold, 2012***

Country records: Antioquia: Yolombó^1^; Cesar: Valledupar^6^; Cundinamarca: Apulo^1^, Beltrán^1^, Fusagasugá^1^, La Mesa^1^, La Vega^1^, Paime^1^, Pandi^1^, Prado^1^, San Antonio del Tequendama^1^, Tena^1^, Villeta^1^, Útica^1^; Huila: Gigante^1^, Neiva^17^, Rivera^1^; Norte de Santander: Convención^16^; Sucre: Ricaurte^1^; Tolima: Armero†^(Gonzalez & Griswold, 2012)^, Ataco^1^, Ibagué^1^, Prado^1^.

***Lestrimelitta* *rufa* (Friese, 1903)***

**Country records**: Caquetá: La Macarena^1^; Cauca: Santa Rosa^11^; Cundinamarca: Medina^1^; Guainía: Inírida^11^; Meta: Villavicencio^1,20^.

***Lestrimelitta* *rufipes* (Friese, 1903)**

**Country records**: Cauca: Piamonte^1,11^; Meta: Puerto López^1^, Villavicencio^1^.

***Lestrimelitta* *spinosa* Marchi & Melo, 2006***

**Country records**: Caquetá: Florencia^11^; Casanare: Villanueva^1^; Guainía: San Felipe^11^; Meta: Cumaral^1^, Granada^1^, La Macarena^1^, Restrepo^1^, Villavicencio^1^.

**Genus *Leurotrigona* Moure, 1950**

***Leurotrigona* *muelleri* (Friese, 1900)***

**Country records**: Cauca: Piamonte^16^; Guainía: La Guadalupe^11^, San Felipe^11^.

**Genus *Melipona* Illiger, 1806**

**Subgenus *Eomelipona* Moure, 1992**

***Melipona* *(Eomelipona)* *puncticollis* Friese, 1902**

**Country records**: Amazonas: El Encanto^5^

**Subgenus *Melikerria* Moure, 1992**

***Melipona* *(Melikerria)* *ambigua* Roubik & Camargo, 2012***

**Country records**: Bolívar: Cartagena^20^†, Turbaco^1^; Córdoba: Montería^1^; Sucre: Ricaurte^1^.

***Melipona* *(Melikerria)* *compressipes* (Fabricius, 1804)**

**Country records**: Amazonas: Leticia^5^; Arauca: Arauca^1^; Boyacá: Puerto Boyacá^5^; Caquetá: Florencia^1^; Casanare: Pore^1^, Villanueva^1^; Cundinamarca: Nilo^5^, Paime^1^, Paratebueno^1^; Guainía: Inírida^1^, Puerto Colombia^1^, San Felipe^1^; Meta: Puerto Gaitán^1^, Puerto López^1^, Vistahermosa^1^; Norte de Santander: Convención^1^, Los Patios^1^; Santander: Encino^17^; Vaupés: Papunaua^1^, Yavaraté^1^; Vichada: Cumaribo^17,5,8^, Puerto Carreño^17^.

***Melipona* *(Melikerria)* *grandis* Guérin-Méneville, 1845**

**Country records**: Amazonas: La Chorrera^19^, La Pedrera^1^, Leticia^1,17^, Puerto Santander^1^; Caquetá: El Paujil^1^, Florencia^1,11^, San Vicente del Caguán^1^; Casanare: Villanueva^28^; Cauca: Piamonte^11^; Cundinamarca: Medina^1,5^, Paratebueno^1^; Guaviare: Calamar^1^, San José del  Guaviare^1^; Meta: Acacías^1^, Cumaral^1^, La Macarena^17,20^, Puerto López^1^, Puerto Rico^1^, Restrepo^1^, San Juan de Arama^1,17^, Villavicencio^1^, Vistahermosa^1,5^; Putumayo: Mocoa^1^, Puerto Asís^5^; Santander: Lebrija^1^; Vaupés: Mitú^1^, Papunaua^1^.

***Melipona (Melikerria) interrupta* Latreille, 1811**

**Country records**: Amazonas: Tarapacá^1^

***Melipona* *(Melikerria)* *salti* Schwarz, 1932**

**Country records**: Antioquia: San Carlos^1^, San Francisco^1^, San Luís^2^, San Roque^1^, Sonsón^1^; Boyacá: Muzo^19,28^, Puerto Boyacá^1^; Caldas: Marulanda^1^, Victoria^1^; Cesar: Agustín Codazzi^19^, Becerril^28^, La Paz^28^; Cundinamarca: Arbeláez^1^, Paime^1^, Pandi^1^, Puerto Salgar^1^, Útica^1^; Magdalena: Santa Marta^1,17^, Zona Bananera^19,28^†; Santander: Cimitarra^1,17^, Floridablanca^1^, Lebrija^1^, Piedecuesta^1^; Sucre: Sampués^1^; Tolima: Ambalema^1^, Cunday^1^, Honda^17^.

**Subgenus *Melipona* Illiger, 1806**

***Melipona* *(Melipona) favosa* (Fabricius, 1798)**

**Country records**: Antioquia: Yondo^1^; Arauca: Arauca^1^, Arauquita^1,5^; Atlántico: Barranquilla^1^, Galapa^28^, Puerto Colombia^1^, Tubará^1^; Bolívar: Cartagena^1,5,20^, Córdoba^17^, Magangué^1^, Mompós^13^, San Juan Nepomuceno^1^, Zambrano^1,17^; Caldas: La Palma^1^; Casanare: Maní^1,5,28^, Orocué^1^, Paz de Ariporo^1^, Sabanalarga^5^, San Luís de Palenque^1^, Trinidad^1^, Villanueva^1^, Yopal^1,28^; Cesar: Becerril^28^, Pueblo Bello^28^, Valledupar^1,17,28^, Cundinamarca: Apulo^1^, Arbeláez^1^, El Colegio^1^, Guaduas^1^, La Mesa^1^, Medina^1,5^, Melgar^1^, Nilo^1,5^, Paratebueno^1^, Ricaurte^1^, San Antonio del Tequendama^1^, Silvania^1^, Tocaima^3^, Útica^1^; Córdoba: Buenavista^28^, Montería^1,28^, San Antero^1^; Guainía: Puerto Carreño^1^; Guaviare: San José del Guaviare^1^; Huila: Neiva^1,17^, Rivera^1^, Villavieja^7,28^; La Guajira: Manaure^28^, Riohacha^1,6^, Uribia^18,28^; Magdalena: Aracataca^6^, Ciénaga^6,28^, El Guamo^28^, El Retén^6,28^,Santa Marta^1,17,6,28^, Tenerife^19^,Zona Bananera^3^; Meta: Puerto Gaitán^1,11^, Puerto López^1,2^, Villavicencio^1^; Putumayo: Puerto Guzmán^1^; Santander: Aratoca^1^, Barrancabermeja^13^, Floridablanca^1^, Sabana de Torres^5^; Sucre: Corozal^28^, Galeras^28^, Los Palmitos^1^, Ricaurte^1^, Sampués^1^, San Benito Abad^28^, San Marcos^28^, San Onofre^1,13^, Sincelejo^1^; Tolima: Ambalema^1^, Ataco^1^; Vichada: Puerto Carreño^1^.

**Subgenus *Meliponiella* Melo, 2021**

***Melipona* *(Meliponiella)* *bradleyi* Schwarz, 1932**

**Country records**: Amazonas: Puerto Santander^1^; Guainía: Inírida^2^, San Felipe^1^.

***Melipona* *(Meliponiella)* *illustris* Schwarz, 1932***

**Country records**: Amazonas: Leticia^17^; Caquetá: Florencia^1^, Miraflores^17^; Guainía: Inírida^11^, La Guadalupe^11^, San Felipe^1,11^; Vaupés: Papunaua^1^, Yavaraté^1^.

**Subgenus *Michmelia* Moure, 1975**

***Melipona* *(Michmelia) captiosa* Moure, 1962**

**Country records**: Amazonas: Tarapacá^1^; Caquetá: Miraflores^17^, San Vicente del Caguán^1^, Solano^1^; Cauca: Piamonte^1^; Guaviare: Calamar^1^; Vaupés: Taraira^17^.

***Melipona* *(Michmelia)* *costaricensis* Cockerell, 1919***

**Country records**: Antioquia: Amalfi^13^, Anorí^13^, El Carmen de Viboral^1^, Jericó^1^, Maceo^1,13^, Puerto Berrío^13^, Puerto Nare^1,13^, San Francisco^1,13,17^, San Luís^2^, San Rafael^1^, San Roque^1,13^, Sonsón^1,17^, Yolombó^13^; Boyacá: Miraflores^1^, Otanche^1^, Pauna^1^; Casanare: La Salina^1^, Puerto Boyacá^1^, Sacama^1^; Chocó: Alto Baudó^1^, Bahía Solano^1,17^, Nuquí^13^, Quibdó^1^; Cundinamarca: Arbeláez^1^, El Peñon^1^, Paime^1^, Vianí^1^, Zipacon^1^; Risaralda: Pereira^1^; Santander: Bucaramanga^1^, Cimitarra^1^, El Carmen de Chucurí^13^, Floridablanca^1^, Landázuri^1^, Lebrija^1^, Piedecuesta^1^, Socorro^1^; Sucre: Ricaurte^1^; Valle del Cauca: Buenaventura^1,15,17^.

***Melipona* *(Michmelia)* *cramptoni* Cockerell, 1920***

**Country records**: Meta: Vista Hermosa^5^.

***Melipona* *(Michmelia)* *crinita* Moure & Kerr, 1950**

**Country records**: Amazonas: Leticia^1^, Tarapacá^19^; Boyacá: Muzo^19^; Cauca: Piamonte^11^; Guainía: San Felipe^11^; Vaupés: Mitú^1^, Papunaua^1^, Yavaraté^1^.

***Melipona* *(Michmelia)* *eburnea* Friese, 1900**

**Country records**: Amazonas: La Pedrera^1^, Leticia^28^, Puerto Santander^1^; Bolívar: Cartagena^28^; Boyacá: Muzo^19^; Caquetá: Florencia^11^; Cauca: Piamonte^11^, Cundinamarca: Medina^1,28^, Paratebueno^1^, Guainía: Inírida^1^, La Guadalupe^11^; Guaviare: Miraflores^28^; Meta: Acacías^1^, La Macarena^20^, San Juan de Arama^28^, Villavicencio^1^; Putumayo: Mocoa^1^; Vaupés: Taraira^17^.

***Melipona* *(Michmelia)* *fulva* Lepeletier, 1836**

**Country records**: Amazonas: La Pedrera^1^, Puerto Santander^1^;

***Melipona* *(Michmelia)* *fuscopilosa* Moure & Kerr, 1950***

**Country records**: Amazonas: Leticia^17,28^; Caquetá: Miraflores^17^; Meta: La Macarena^28^.

***Melipona* *(Michmelia)* *illota* Cockerell, 1919***

**Country records:** Amazonas: El Encanto^28^.

***Melipona* *(Michmelia)* *indecisa* Cockerell, 1919***

**Country records**: Antioquia: Chigorodó^1^, Frontino^1^; Córdoba: Tierralta^1^; Nariño: Barbacoas^1^; Norte de Santander: Toledo^17^; Valle del Cauca: Buenaventura^15,17^.

***Melipona* *(Michmelia) nebulosa* Camargo, 1988**

**Country records**: Amazonas: La Pedrera^1^, Leticia^1^, Puerto Santander^1^, Tarapacá^1^; Cauca: Piamonte^1^; Guaviare: Calamar^1^, Miraflores^1^; Putumayo: Mocoa^1^; Vaupés: Mitú^1^.

***Melipona* *(Michmelia)* *nigrescens* Friese, 1900**

**Country records**: Antioquia: Betania^1^, Concordia^1^, Jardín^13^; Boyacá: Chiquiza^1^, Muzo^19^, Zetaquira^3^; Caldas: El Carmen^1^, Neira^28^, Salamina^15,28^, Villamaría^1^; Caquetá: San Vicente del Caguán^1^; Cauca: Él Tambo^28^, Inzá^1^, La Vega^28^, Popayán^19^; Cundinamarca: San Antonio del Tequendama^1^, Tena^1^; Huila: Hobo^1^, Isnos^1^; Nariño: Barbacoas^1,11,17^, Cumbitara^1^, Ricaurte^1,17^; Norte de Santander: Cucutilla^13^; Quindío: Armenia^15^, Calarcá^15,28^; Risaralda: Mistrató^1^, Pereira^1^, Santuario^5^; Tolima: Ibagué^1^; Valle del Cauca: Buenaventura^19^, El Águila^28^, El Dovio^1^, Cali^11,28,20^ ; Trujillo^28^.

***Melipona* *(Michmelia)* *panamica* Cockerell, 1919**

**Country records**: Valle del Cauca: Buenaventura^1^.

***Melipona* *(Michmelia)* *paraensis* Ducke, 1916***

**Country records**: Guainía: Inírida^16^, La Guadalupe^16^, Puerto Colombia^1^, San Felipe^1^; Meta: Vistahermosa^5^; Vaupés: Mitú^1^, Papunaua^1^.

***Melipona* *(Michmelia)* *rufescens* Friese, 1900**

**Country records**: Amazonas: Leticia^1^, Puerto Santander^1^; Boyacá: Miraflores^1^; Cundinamarca: Silvania^1^; Meta: La Macarena^1^, San Juan de Arama^1^.

***Melipona* *(Michmelia)* *seminigra* Friese, 1903**

Country records: Meta: Villavicencio^1^, Vistahermosa^5^; Vichada: Cumaribo^5^.

**Subgenus *Mouremelia* Engel, 2021**

***Melipona* *(Mouremelia)* *fallax* Camargo & Pedro, 2008***

**Country records**: Antioquia: Amalfi^(Camargo & Pedro, 2008)^, Sonsón^17^.

***Melipona* *(Mouremelia) fuliginosa* Lepeletier, 1836***

**Country records**: Cundinamarca: Medina^28^; Meta: La Macarena^20^.

***Melipona* *(Mouremelia)* *titania* Gribodo, 1893***

**Country records**: Amazonas: Leticia^1^; Antioquia: El Carmen de Viboral^1^; Bolívar: Cartagena^1^; Caquetá: Cartagena del Chairá^1^; Cauca: Caloto^1^, Piamonte^1,11^; Chocó: San José del Palmar^1^; Cundinamarca: Medina^1^, Paime^1^; Meta: Acacías^1^, Villavicencio^1,17^; Putumayo: Mocoa^1,9^; Santander: Bucaramanga^1^, Lebrija^1^; Tolima: Ibagué^10^; Vaupés: Mitú^1^, Papunaua^1^.

**Genus *Nannotrigona* Cockerell, 1922**

***Nannotrigona* *camargoi* Rasmussen & Gonzalez, 2017***

**Country records**: Antioquia: Frontino^1^, Ituango^14^; Chocó: Condoto^1^, Litoral del Bajo San Juan^1^, Lloró^1^, Nuquí^1^, Quibdó^1^, San José del Palmar^1^, Tadó^1^; Nariño: El Charco^1^, Tumaco^1^; Risaralda: Mistrató^1^; Valle del Cauca: Buenaventura^1^.

***Nannotrigona* *gaboi* Jaramillo, Ospina & Gonzalez, 2019***

**Country récords**: Antioquia: Amagá^13^, Amalfi^13^, Andes^13^, Barbosa^14^, Cisneros^14^, Concordia^1^, Damasco^14^, Don Matías^1^, Fredonia^13^, Hispania^13^, Maceo^13^, Medellín^1,13,9^, San Francisco^18^, San Jerónimo^14^, San Roque^1^, Santa Fe de Antioquia^14^, Sonsón^17^, Tarazá^14^, Yalí^3^, Yolombó^1^; Boyacá: Boavita^1^, Buenavista^1^, Moniquirá^1^, Otanche^1^, San Mateo^1^; Caldas: Chinchiná^1^, Pensilvania^1^, Victoria^1^; Cauca: Popayán^1^; Cesar: Agustín Codazzi^19^, Pueblo Bello^1^; Cundinamarca: Anolaima^1^, Apulo^1^, Arbeláez^1^, Beltrán^1^, Cachipay^1,3^, Caparrapí^1^, El Peñón^1^, Fusagasugá^1^, Gachalá^1^, Girardot^1^, Guaduas^1^, La Mesa^1^, La Palma^1,14^, La Vega^1^, Pacho^1^, Paime^1,14^, Pandi^1^, Paratebueno^1^, Quipile^1^, Ricaurte^1^, San Antonio del Tequendama^1^, Sasaima^1^, Silvania^1^, Tena^1^, Ubalá^1^, Vergara^1^, Villeta^1^, Útica^1^; Córdoba: Tierralta^1^; Huila: Altamira^1^, Baraya^1^, Campoalegre^1^, Colombia^1^, Garzón^1^, Gigante^1^, Guadalupe^1^, La Argentina^1^, Neiva^1,18^, Palestina^1^, Pital^1^, Rivera^1^, Tello^1^, Teruel^1^, Villavieja^7^; La Guajira: Dibulla^6^; Magdalena: Ciénaga^1^, Córdoba^1^, Pivijay^1^, Santa Marta^1,6^; Quindío: Armenia^1^, Calarcá^1^, Circasia^1^, Granada^1^, Montenegro^1^, Quimbaya^1^; Risaralda: Pereira^1^; Santander: Bucaramanga^1^, Charalá^1^, Enciso^7^, Floridablanca^1^, Lebrija^1^, Málaga^1^, Piedecuesta^1^, Socorro^1,7^; Sucre: Coloso†^¹^ ; Tolima: Anzoátegui^1^, Ataco^1^, Chaparral^1^, Coello^2^, Dolores^1^, Ibagué^1,3^, Icononzo^1^, Líbano^1^, Prado^1^; Valle del Cauca: Palmira^1^.

***Nannotrigona* *melanocera* (Schwarz, 1938)**

**Country records**: Amazonas: La Pedrera^1^, Leticia^1,2^, Puerto Santander^1^, Puerto Ñarino^7^; Puerto Santander^¹^; Boyacá: Santa María^2^;Caquetá: Florencia^1,16^, San Vicente del Caguán^1^, Valparaíso^11^; Casanare: Chámeza^1^, La Salina^1^, Pore^1^, Villanueva^1^; Cauca: Piamonte^1,11^; Cundinamarca: Gachalá^1^, Guayabetal^1^, Medina^1^, Paratebueno^1^; Guaviare: Calamar^1^, San José del Guaviare^1,2^; Meta: Acacías^1^, Cumaral^1^, Fuente de Oro^3^, Granada^1^, Guamal^1^, Puerto Gaitán^1^, Puerto Lleras^1^, Puerto López^1,2,3^, Puerto Rico^1^, Restrepo^1^, San Martín^2,3^, Villavicencio^1^; Putumayo: Santa Rosa^1^; Vaupés: Taraira^17^; Vichada: Cumaribo^1^.

***Nannotrigona* *mellaria* (Smith, 1862)**

**Country records**: Antioquia: Amalfi^28^, Angelopolis^28^, Medellín^1^, San Roque^1^, Vijes^28^, Yolombó^28^; Cauca: Corinto^19^, Puracé^19^; Cesar: Valledupar^28^; Cundinamarca: Anapoima^1^, Anolaima^1^, Fusagasugá^1^, La Mesa^1^, Paime^1^, San Antonio del Tequendama^1^, Sasaima^1^, Tena^1^, Villeta^28^; Huila: Timana^28^; Quindío: San Sebastián de Mariquita^¹⁵^; Tolima: Ataco^1^, Honda^17,28^; Valle del Cauca: Cali^19,28^, Restrepo^19^.

***Nannotrigona* *occidentalis* Jaramillo, Ospina & Gonzalez, 2019***

**Country records**: Antioquia: Amalfi^14^, San Francisco^17^; Caldas: Riosucio^1^; Chocó: Litoral del Bajo San Juan†^1^.

***Nannotrigona* *pilosa* Jaramillo, Ospina & Gonzalez, 2019***

**Country records**: Antioquia: Amagá^13^, Andes^13^, Bello^14^, Envigado^14^, Fredonia^13^, Hispania^13^, Medellín^13,14^, Tarso^13^, Yolombó^1^; Caldas: Chinchiná^1^; Cauca: Santander de Quilichao^1^; Putumayo: Mocoa^1^; Quindío: Armenia^1^, La Tebaida^1^, Montenegro^1^, Quimbaya^6^; Risaralda: Mistrató^1^, Pereira^1^, Santa Rosa de Cabal^1^; Valle del Cauca: Buenaventura^1^, Caicedonia^1^, Ginebra^1^, Palmira^1,12^, Cali^1^†; Tuluá^14^.

***Nannotrigona* *schultzei* (Friese, 1901)**

**Country records**: Amazonas: Leticia^1,2,28^, Puerto Santander^1^; Caquetá: Solano^¹^; Cauca: Piamonte^¹¹^; Guainía: La Guadalupe^¹¹^, San Felipe^¹¹^; Guaviare: San José del Guaviare^¹^.

***Nannotrigona* *tristella* Cockerell, 1922***

**Country records**: Antioquia: Amalfi^13,14^, Betania^1^, Betulia^1^, Caucasia^13^, Ciudad Bolívar^13,14^, Fredonia^13^, Hispania^13^, Itagüí^14^, Ituango^14^, Jericó^1^, Maceo^13^, Puerto Triunfo^14^, San Francisco^17^, San Jerónimo^14^, San Roque^1^, Santa Fe de Antioquia^14,2^, Sonsón^17^, Tarazá^14^, Tarso^13^, Yolombó^1^; Bolívar: San Jacinto^1^, San Juan Nepomuceno^1^; Boyacá: Muzo^19^, Puerto Boyacá^1^; Caldas: Aguadas^1^, Chinchiná^1,28^, La Palma^1^, Pensilvania^1^, Victoria^1^; Cauca: Corinto^19^,Popayán^19^, Tierradentro^14^; Cesar: Becerrill^19^; Ariguaní^19^, Becerril^19^, Valledupar^14^; Cundinamarca: Agua de Dios^7^, Anapoima^1^, Anolaima^1^, Apulo^1^, El Colegio^1^, Fusagasugá^1^, Gachalá^1^, La Mesa^1^, La Vega^1,7^, Nimaima^1^, Pandi^1^, Puerto Salgar^1^, Quipile^1^, San Antonio del Tequendama^1^, Sasaima^1^, Tena^1^, Tibacuy^1^, Tocaima^1,7^, Villeta^1,19^, Zipacon^1^, Útica^1^; Córdoba: Tierralta^1^; Huila: Colombia^1^, Neiva^17^, Oporapa^1^, Palermo^1^, Pitalito^1^; Magdalena: Ciénaga^1^, Córdoba^1^, Santa Marta^1,6^, Zona Bananera^19^; Quindío: Buenavista^28^, Quimbaya^6,7^; Risaralda: Pereira^1^; Santander: Cimitarra^1,19^, Landázuri^1^; Sucre: Ricaurte^1^; Tolima: Armero^2^, Ataco^1^, Carmen de Apicalá^1^,Chaparral^1^, Cunday^1,2^, Dolores^1^, Honda^1^, Melgar^1^; Valle del Cauca: Andalucía^1^, Buga^1^, El Cerrito^1^, Palmira^1,12^, Cali^1^Tuluá^14^, Uribe^19^.

**Genus *Nanoplebeia* Engel, 2021**

***Nanoplebeia* *asthenes* Engel, 2021***

**Country records**: Amazonas: Leticia^17^; Caquetá: Solano^17^; Meta: La Macarena^17^.

***Nanoplebeia* *franki* (Friese, 1900)**

**Country records**: Antioquia: Amalfi^28^, Hispania^13^, San Francisco^17^, Yolombó^28^; Caldas: Aguadas^15,28^, Riosucio^1^, Victoria^1^; Risaralda: Pereira^1^; Tolima: Armero^3^; Valle del Cauca: El Cerrito^15^.

***Nanoplebeia*** ***margaritae*** **(Moure, 1962)***

**Country records**: Caquetá: Solano^17^.

**Genus *Nogueirapis* Moure, 1953**

***Nogueirapis* *butteli* (Friese, 1900)**

**Country records**: Amazonas: El Encanto^5^, La Pedrera^1^, Leticia^1,17,2^, Mirití-Paraná^1^, Puerto Santander^1,5^; Caquetá: Florencia^1^, Miraflores^17^, Puerto Santander^1^; Solano^¹^; Guainía: Inírida^17^; San Felipe^¹¹^; Guaviare: San José del Guaviare^¹⁷^; Vaupés: Taraira^¹⁷^.

***Nogueirapis* *mirandula* (Cockerell, 1917)**

**Country records**: Cauca: Guapi^1,17^; Chocó: Litoral del Bajo San Juan^1^, Nuquí^1^, San José del Palmar^1^; Valle del Cauca: Buenaventura^17^.

**Genus *Oxytrigona* Cockerell, 1917**

***Oxytrigona* *chocoana* Gonzalez & Roubik, 2008***

**Country records**: Chocó: Nuquí^(González & Roubik, 2008)^.

***Oxytrigona* *daemoniaca* Camargo, 1984**

**Country records**: Antioquia: Amalfi^13,14,28^, Chigorodó^3^, Jardín^1,14^, San Jerónimo^14^, San Pedro de Urabá^14^, Santa Fe de Antioquia^14^, Sonsón^17^, Sopetrán^14,17^, Tarazá^14^, Tarso^13^, Yolombó^1,13,28^; Boyacá: Otanche^1^; Caldas: Aguadas^15^, La Dorada^1^, Victoria^1^; Cesar: Valledupar^14,3^; Cundinamarca: Anapoima^1^, Apulo^1^, Caparrapí^1^, El Colegio^1^, Fusagasugá^1^, Guayabal de Síquima^1^, La Mesa^1^, Pandi^1,14^, Puerto Salgar^1^, Quipile^1^, Sasaima^1^, Tena^1^, Tocaima^1^, Topaipí^1^,Vergara^1,14^, Villeta^1,28^, Zipacon^1^; Huila: Baraya^1^, Campoalegre^1^, Palermo^1^, Pital^1^, Rivera^1^; Magdalena: Ciénaga^6^, Santa Marta^17,6,28^; Norte de Santander: Tibú^1^; Santander: Cimitarra^1^, Landázuri^1^, Piedecuesta^1^, Rionegro^1^; Sucre: Ricaurte^1^; Tolima: Ataco^1^, Cunday^1,2^, Dolores^1^, Ibagué^1^, Lérida^1^, Melgar^1^, Rovira^3^, San Sebastián de Mariquita^1^.

***Oxytrigona* *flaveola* (Friese, 1900)**

**Country records**: Amazonas: Leticia^1^; Boyacá: Sotaquirá^1^; Caquetá: San Vicente del Caguán^1^, Solano^17^; Cundinamarca: Guayabetal^1^, Medina^1^; Guaviare: Calamar^1^; Meta: Acacías^1^, La Macarena^1^, Restrepo^1^, Villavicencio^1,14,3^; Vichada: Cumaribo^8^.

***Oxytrigona* *mulfordi* (Schwarz, 1948)**

**Country records**: Cundinamarca: Medina^1^; Guaviare: San José del Guaviare^1^; Meta: Fuente de Oro^1^, Puerto Lleras^1^, Restrepo^1^, Vistahermosa^20^.

***Oxytrigona* *obscura* (Friese, 1900)***

**Country records**: Caquetá: San José del Fragua; Cauca: Piamonte^11^; Cundinamarca: Medina^28^; Putumayo: Mocoa^1^.

***Oxytrigona* *pallida* Lepeco, 2024***

**Country records**: Antioquia: Amalfi^14^, Currulao^14^, Jardín^14^, Puerto Berrío^14^, Puerto Nare^13^, San Francisco^17^, Sonsón^17^, Tarazá^14^, Yolombó^1,28^; Bolívar: San Jacinto^1^; Boyacá: Pajarito^1^, Puerto Boyacá^1^; Caldas: La Dorada^1^, La Palma^1^, Samaná^2^, Támesis^1^, Victoria^1^; Cundinamarca: Anapoima^1^, Apulo^1^, Beltrán^1^, Fusagasugá^28^, Guaduas^1^, Nilo^1^, Puerto Salgar^1^; Córdoba: Montería^1^, San Antero^1^; Huila: Neiva^1^, Palermo^1^, Villavieja^7^; Nariño: Tumaco^14^; Norte de Santander: Él Carmen^1^; Santander: Cimitarra^1^, Rionegro^1^; Sucre: Coloso^¹^; Tolima: Chaparral^³^, Coello^²^, Cunday^¹,²^, El Espinal^³^, Guamo^28^, Honda^¹^, Ibagué^¹^, Melgar^¹^.

***Oxytrigona* *sauronica* Lepeco, 2024 §***

**Country records**: Casanare: Tamará†^(Lepeco, 2024)^.

**Genus *Parapartamona* Schwarz, 1939**

***Parapartamona* *brevipilosa* (Schwarz, 1948)**

**Country records**: Antioquia: Alejandría^13^, Amalfi^14^, Belmira^13^, Copacabana^8^, El Carmen de Viboral^14^, Envigado^1,14^, Frontino^1^, Giraldo^8^, Jardín^13^, La Estrella^14^, Medellín^1,13,14,8^, Rionegro^14^, Yarumal^13^; Boyacá: Arcabuco^1,17^, Chiquiza^1^, Paipa^1^, Ventaquemada^17^, Villa de Leyva^1,17,18,7^; Caldas: Manizales^13^,Pácora^15^, Salamina^1,15^; Caquetá: Florencia^1^, San Vicente del Caguán^1^; Cauca: Cajibío^1^, Puracé^5^, Santa Rosa^11^; Cesar: Agustín Codazzi^19^; Chocó: San José del Palmar^1^; Cundinamarca: Funza^1^, Gachetá^1^, Medina^1^, San Antonio del Tequendama^1,2^, Venecia^2^, Villeta^1^; Huila: Algeciras^1^, Hobo^1^, La Plata^1,11^, San Agustín^1^; Quindío: Calarcá^15^, Filandia^2^, Salento^15^; Risaralda: Mistrató^1^, Pereira^1^, Santuario^1^; Tolima: Ibagué^1^.

***Parapartamona*** ***zonata* (Smith, 1854)**

**Country records**: Antioquia: Frontino^1^; Boyacá: Aquitania^1^, Arcabuco^1^, Boyacá^1^, Buenavista^1^, Duitama^1^, Macanal^1^, Mongua^18^, Pajarito^1^, Pauna^1^, Saboya^1^, Villa de Leyva^3^; Caldas: Pácora^28^, Pensilvania^28^, Salamina^28^; Caquetá: San Vicente del Caguán^1^; Casanare: Maní^1^; Cauca: Cajibío^1^; Chocó: San José del Palmar^1^; Cundinamarca: Albán^1^, Anolaima^1^, Bogotá, D.C.^28^, Bojacá^1^, Cáqueza^1^, Choachí^7^, El Peñón^1^, Funza^1^, Fómeque^1^, Gachetá^1^, Guasca^1^, Guayabetal^1^, Junín^1^, La Vega^7^, Madrid^1^, San Antonio del Tequendama^1,2,7^, San Francisco^1^, Silvania^1,15^, Soacha^7^, Tena^1^, Tenjo^1^ ; Villavicencio^¹^ ;  Villeta^1^, Viotá^1^, Zipaquirá^1^; Huila: La Plata^1^; Meta: Puerto López^1^, Villavicencio^5,1^; Nariño: Barbacoas^1,5^, La Tola^1^, Pasto^1^, Ricaurte^17^; Quindío: Calarcá^15^, Filandia^2^; Risaralda: El Santuario^5^, Pereira^20^, Santuario^1^; Santander: Charalá^5^, Enciso^7^, Socorro^7^; Tolima: San Sebastián de Mariquita^2^; Valle del Cauca: Calima^20^, Dagua^20^, El Cairo^1^; Cali^12,19,28,20^.

**Genus *Paratrigona* Schwarz, 1938**

***Paratrigona* *anduzei* (Schwarz, 1943)**

**Country records**: Boyacá: Macanal^1^, Santa María^2^; Casanare: La Salina^1^; Cundinamarca: Gachalá^1^, Gachetá^1^, Junín^1^, Tibiritá^1^, Ubalá^1^.

***Paratrigona* *eutaeniata* Camargo & Moure, 1994**

Country records: Antioquia: Bello^3^, Betania^1^, La Ceja^3^, Rionegro^1^; Boyacá: Buenavista^1^, Chiquinquirá^5^, Duitama^5^, Maripí^1^; Cauca: Él Tambo^19,28^; Cundinamarca: Anolaima^1,3^, Apulo^1^, Arbeláez^1,5^, Cachipay^1^, Chipaque^28^, Fusagasugá^1^, Gachalá^1,28^, Gama^1^, La Mesa^1^, La Palma^1^, La Vega^1^, Pacho^1^, Quipile^1^, San Antonio del Tequendama^1^†, San Juan de Río Seco^1^, Silvania^1,5^, Tena^1^, Villeta^1^; Santander: Bucaramanga^1^, Charalá^1,5^, Curití^1^, Floridablanca^1^, Landázuri^5^, Oiba^1^, Santa Bárbara^1^, Socorro^1^, Tona^1^; Tolima: Dolores^1^, Icononzo^1,5^.

***Paratrigona* *lineatifrons* (Schwarz, 1938)***

**Country records**: Guainía: San Felipe^11^

***Paratrigona*** ***lophocoryphe* Moure, 1963**

**Country records**: Antioquia: Frontino^1^, San Roque^1^; Caldas: Chinchiná^1^; Chocó: San José del Palmar^1^; Valle del Cauca: Buenaventura^28,20^.

***Paratrigona* *nuda* (Schwarz, 1943)**

**Country records**: Caquetá: San Vicente del Caguán^1^; Putumayo: Mocoa^1^.

***Paratrigona*** ***opaca* (Cockerell, 1917)**

**Country records**: Antioquia: San Francisco^17^, Sonsón^17^; Bolívar: San Juan Nepomuceno^1^; Boyacá: Arcabuco^28^, Chiquinquirá^28^, Muzo^19^, Pauna^1,5^; Cesar: Pueblo Bello^28^; Cundinamarca: La Vega^1^, Paime^1^, Villeta^1^; Magdalena: Ciénaga^1^, Fundación^19^, Santa Marta^1,6,28^; Nariño: Tumaco^13^; Quindío: Quimbaya^6^; Santander: Floridablanca^1,28^, Landázuri^1^, Lebrija^1^; Tolima: Fresno^1^.

***Paratrigona*** ***pacifica* (Schwarz, 1943)**

**Country records**: Amazonas: Leticia^1^; Boyacá: Miraflores^1^, Santa María^1^; Casanare: Chámeza^1^; Cundinamarca: Medina^1^; Meta: Cumaral^1^, Villavicencio^1^.

***Paratrigona*** ***prosopiformis* (Gribodo, 1893)**

**Country records**: Amazonas: La Pedrera^1^, Leticia^20^, Tarapacá^1^; Boyacá: San Luis de Gaceno^1^, Santa María^1^; Caquetá: El Paujil^1^, Florencia^1^; Cundinamarca: Medina^1^; Guaviare: Calamar^1^; Meta: Acacías^1,5^, Cumaral^1^, Restrepo^1^, Villavicencio^1,5^.

***Paratrigona*** ***rinconi*** **Camargo & Moure, 1994**

**Country records**: Antioquia: Amagá^13^, Andes^13^, Bello^3^, Ciudad Bolívar^13^, Envigado^28^, Fredonia^13,28^, Guarne^28^, Itagüí^13,28^, Jericó^13^, La Ceja^3^, Medellín^1,13,9,28^, Pueblorrico^28^†, San Andrés de Cuerquía^2^, Santa Bárbara^19^, Yarumal^2^; Caldas: Aguadas^1^, Aranzazu^15^, Chinchiná^1^, Pensilvania^1^, Riosucio^1^, Supía^1^; Cauca: Balboa^11^, La Sierra^16^, Popayán^11^, Santa Rosa^11^, Timbío^11^; Huila: Acevedo^1,20^, San Agustín^20^; Nariño: La Florida^5^; Quindío: Armenia^15^, Circasia^15^, Montenegro^15^, Quimbaya^6^; Risaralda: Pereira^5^, Pueblo Rico^13,2^; Santander: Santa Barbara^19^; Valle del Cauca: Él Dovio^¹^, Pradera^¹⁵^, Santiago de Cali^1,3,28,20^.

***Paratrigona* *scapisetosa* Gonzalez & Griswold, 2011***

**Country records**: Caquetá: Solano^(Gonzalez & Griswold, 2011)^; Vaupés: Mitú^1^.

***Paratrigona* *uwa* Gonzalez & Vélez, 2007**

**Country records**: Antioquia: San Carlos^1^, San Roque^1^; Cundinamarca: La Vega^1^, Tena^1^, Tibacuy^1^, Villeta^1^; Santander: Charalá†^1^, Curití^5^.

**Genus *Paratrigonoides* Camargo & Roubik, 2005**

***Paratrigonoides* *mayri* Camargo & Roubik, 2005**

**Country records**: Antioquia: Maceo^13^, San Jerónimo†^14^, Santa Fe de Antioquia^14^; Bolívar: Cartagena^14^, San Jacinto^17^, San Juan Nepomuceno^1,5,19^_._

**Genus *Partamona* Schwarz, 1939**

***Partamona*** ***aequatoriana* Camargo, 1980**

Country records: Chocó: Bahía Solano^28^, Litoral del Bajo San Juan^1^; Magdalena: Santa Marta^1^; Risaralda: Mistrató^20^; Valle del Cauca: Buenaventura^28,20^.

***Partamona* *ailyae* Camargo, 1980***

Country records: Guainía: San Felipe^11^.

***Partamona*** ***auripennis* Pedro & Camargo, 2003**

Country records: Meta: Puerto López^(Pedro & Camargo, 2003)^.

***Partamona*** ***epiphytophila* Pedro & Camargo, 2003**

**Country records**: Caquetá: Florencia^11^; Guainía: San Felipe^16^; Meta: Restrepo^28^, Villavicencio^1,19^.

***Partamona*** ***musarum* (Cockerell, 1917)**

**Country records**: Atlántico: Puerto Colombia^28^; Boyacá: Muzo^28^; Cesar: Pueblo Bello^28^, Valledupar^6^; Cundinamarca: San Cayetano^28^; Valle del Cauca: Cali^20^.

***Partamona*** ***peckolti*** **(Friese, 1901)**

**Country records**: Antioquia: Amalfi^20^, El Bagre^28^, Fredonia^28^, Guarne^28^, Rionegro^20^, San Francisco^17^, San Roque^1^, Sonsón^17^, Yarumal^28^, Zaragoza^28^; Boyacá: Arcabuco^1^, Muzo^19^; Caldas: Aguadas^15,28^, Aranzazu^28^, Chinchiná^28^, Pácora^15^, Salamina^15,28^; Casanare: Maní^28^, Yopal^1^; Cauca: Bolívar^20^, El Tambo^28^, Popayán^19,28^, Puracé^28^; Cesar: Agustín Codazzi^19^, Becerril^19^; Curumani^19^ ; Chocó: Istmina^19^, Litoral del Bajo San Juan^1,19^, Pangala^19^; Cundinamarca: Arbeláez^28^, La Mesa^1^, La Palma^1^, Medina^28^, Pacho^1^, Paime^1^, Sasaima^3^, Villeta^28^; Huila: Acevedo^1^, Baraya^1^, Campoalegre^1^, Garzón^1^, Gigante^1^, Guadalupe^1^, Neiva^1^, San Agustín^15,20^, Tello^1^; Magdalena: Santa Marta^19^; Quindío: Armenia^15^, Calarcá^15^, Ibagué^¹⁵^,   Quimbaya^6,28^; Salento^¹⁵^;  Risaralda: Pereira^20^; Santander: Enciso^7^, Floridablanca^28^, Puerto Parra^1^; Tolima: Ibagué^15^,Libano^28^, Melgar^28^; Valle del Cauca: , Andalucía^15^, Buenaventura^19,20^, Calima^20^, Dagua^20^, Jamundí^20^, Pradera^15^ ; Cali^19,28,20^.

***Partamona* *testacea* (Klug, 1807)**

**Country records**: Cauca: Piamonte^11^, Santa Rosa^11^; Cundinamarca: Girardot^28^; Meta: La Macarena^20^, Restrepo^28^, Villavicencio^1,19^.

***Partamona* *vicina* Camargo, 1980**

**Country records**: Caquetá: Florencia^11^; Cauca: Piamonte^11^; Guainía: Inírida^11^, San Felipe^11^; Meta: San Juan de Arama^20^.

**Genus *Plebeia* Schwarz, 1938**

***Plebeia*** ***jatiformis* (Cockerell, 1912)***

**Country records**: Antioquia: San Francisco^17^.

***Plebeia* *mutisi* Engel, 2022 §***

**Country records**: Cundinamarca: La Mesa†^20^.

**Genus *Ptilotrigona* Moure, 1951**

***Ptilotrigona* *lurida* (Smith, 1854)**

**Country records**: Amazonas: Leticia^1, 2, 9,17,18^; Boyacá: San Luis de Gaceno^1^, Santa María^1,2^; Caquetá: El Doncello^11^, Florencia^1,17^; Cundinamarca: Medina^1^, Ubalá^1^; Guainía: Inírida^11^, San Felipe^1,11^; Guaviare: El Retorno^17^, San José del Guaviare^1^; Meta: Acacías^1,18^, La Macarena^1^, Puerto Lleras^1^, Puerto López^1,28^, Puerto Rico^1^, Restrepo^1,19,28^, San Martín^1,2^, Villavicencio^1^; Putumayo: Puerto Leguízamo^17^; Vaupés: Mitú^1^; Vichada: Cumaribo^1,17^, San José del Guaviare^1^.

***Ptilotrigona*** ***occidentalis* (Schulz, 1904)**

Country records: Antioquia: Frontino^1^, Medina^1^, San Luís^2^; Chocó: Bahía Solano^17^, Litoral del Bajo San Juan^1,28^, Lloró^14^, Novita^28^, Nuquí^1^, Quibdó^1,28^, Yuta^14^; Nariño: Tumaco^28^; Valle del Cauca: Buenaventura^1,15^.

***Ptilotrigona*** ***pereneae* (Schwarz, 1943)**

Country records: Amazonas: Leticia^1^; Caquetá: El Doncello^11,28^, Milan^28^; Cauca: Piamonte^1,11^; Cundinamarca: Medina^28^; Guaviare: Calamar^1^, San José del Guaviare^1^; Meta: La Macarena^1^; Putumayo: Mocoa^1^.

**Genus *Scaptotrigona* Moure, 1942**

***Scaptotrigona* *baldwini* Engel, 2022***

**Country records**: Caquetá: Florencia^16^; Guainía: La Guadalupe^16^, San Felipe^16^.

***Scaptotrigona* cf. *bipunctata***

**Country records**: Caquetá: San José del Fragua; Cauca: Piamonte^11^.

***Scaptotrigona*** ***caduceus* Engel, 2022***

**Country records**: Caquetá: San José del Fragüa^⁵^; Meta: San Martín†^19,20^.

***Scaptotrigona* *ederi* Engel, 2022***

**Country records**: Antioquia: Andes^13^, Arboletes^14^, Barbosa^14^, Chigorodó^1^, Jardín^14^, Medellín^13,14,9,20^, Sabaneta^14^, San Francisco^1^, San Jerónimo^14^, Santa Fe de Antioquia^14^, Tarso^13^; Caldas: Aguadas^15^; Cauca: Miranda^12^,Popayán^11^ ; Suárez^11^,  Magdalena: Santa Marta^19,20^; Quindío: Armenia^15^, Calarcá^15^, Circasia^15^, Filandia^13^ La Tebaida^15^; Risaralda: Belén de Umbria^15^,Mistrató^1^; Valle del Cauca: Buenaventura^20^, Buga^14,15^, Calima^20^, Dagua†^20^, El Cairo^14^, El Cerrito^15^, Guacarí^1^, Guadalajara de Buga^15^, Jamundí^20^, La Victoria^15^, Palmira^12,12^, San Pedro^15^, Cali^¹²,¹⁵,20^, Tuluá^14^.

***Scaptotrigona* *gonzalezi* Engel, 2022***

**Country records**: Antioquia: Amalfi^14^, Andes^13^, Barbosa^14^, Caucasia^14^, Cocorná^14^, El Retiro^14^, Fredonia^13^, Girardota^14^, Ituango^14^, Jardín^14^, Medellín^14^, San Luís^¹⁴^, Santa Fé De Antioquia^¹⁴^; Bolívar: San Cristóbal^14^, Caldas: Aguadas^15^, Pensilvania^14^; Cauca: Bolívar†^20^, La Sierra^11^, Patía^20^; Chocó: Litoral del Bajo San Juan^1^; Quindío: Armenia^15^, La Tebaida^15^, Quimbaya^15^; Valle del Cauca: Él Cerrito^15^, Palmira^12^.

***Scaptotrigona* cf. *limae* (Brèthes, 1920)**

**Country records**: Caquetá: Solano^1^; Cundinamarca: Pandi^1^, Ricaurte^1^; Meta: Acacías^1^, Villavicencio^1^.

***Scaptotrigona* *macarenensis* Engel, 2022***

**Country records**: Meta: La Macarena†^20^, Mesetas^20^.

***Scaptotrigona* *magdalenae* Engel, 2022***

**Country records**: Antioquia: Amalfi^13^, Andes^13^, Barbosa^14^, Ciudad Bolívar^13^, Copacabana^14^, Girardota^14^, Ituango^14^, Medellín^14^, Puerto Berrío^13^, Sabaneta^14^, Santa Fé de Antioquia^14^, Sonsón^1,17^, Tarso^13^, Yolombó^1,13^; Caldas: Aguadas^15^, Támesis^1^; Chocó: Quibdó^1^; Cundinamarca: Anapoima^1^, Anolaima^1^, Apulo^1^, Arbeláez^1^, Beltrán^1^, Cachipay^1^, El Colegio^1^, Fusagasugá^1^, Gachalá^1^, Guaduas^1^, La Mesa^1^, La Palma^1^, La Vega^1^, Nilo^1^, Nimaima^1^, Paime^1^, Pandi†^1^, Ricaurte^1^, Sasaima^1^, Silvania^1^, Tocaima^3^, Vergara^1^, Villeta^1^; Huila: Acevedo^1^, Baraya^1^, Campoalegre^1^, Colombia^1^, Gigante^1^, Guadalupe^1^, Neiva^1^, Oporapa^1^, Tello^1^; Magdalena: Santa Marta^1^; Santander: Bucaramanga^3^, Piedecuesta^1^, Sabana de Torres^5^; Tolima: Ambalema^1^, Armero^3^, Ataco^1^, Guamo^20^, Ibagué^1^, Icononzo^20^, San Sebastián de Mariquita^1^.

***Scaptotrigona* *ochrotricha* (Buysson, 1892)***

**Country records**: Meta: Villavicencio^1^.

***Scaptotrigona*** ***pectoralis* (Dalla Torre, 1896)**

**Country records**: Antioquia: Amalfi^28^, Yolombó^1,28^.

***Scaptotrigona* *rosellae* Engel, 2022 §***

**Country records**: Valle del Cauca: Buenaventura†^20^.

***Scaptotrigona*** ***subobscuripennis* (Schwarz, 1951)***

**Country records**: Antioquia: Ituango^14^, Medellín^14^, Bolívar: San Cristóbal^14^; Córdoba: Montería^14^, Tres Palmas^14^.

***Scaptotrigona*** ***tatacoensis* Engel, 2022 §***

**Country records**: Huila: Villavieja†^20^.

***Scaptotrigona* *totobi* Engel, 2022***

**Country records**: Amazonas: Leticia^1^; Boyacá: San Luis de Gaceno^1^; Caquetá: Solano^1^; Cundinamarca: Medina^1^; Guaviare: Calamar^1^; Meta: Cumaral^1^, Granada^1^, La Macarena^1^, Puerto López^1^, San Juan de Arama†^20^, Villavicencio^1^, Vistahermosa^1^.

***Scaptotrigona* *tricolorata* Camargo, 1988**

**Country records**: Amazonas: Leticia^1^; Boyacá: San Luis de Gaceno^1^; Caquetá: El Paujil^1^, Florencia^1^, San Vicente del Caguán^1^; Cauca: Piamonte^1,11^; Meta: La Macarena^1,20^, Villavicencio^1^.

***Scaptotrigona* *tubiba* (Smith, 1863)**

**Country records**: Meta: San Martín^5^.

**Genus *Scaura* Schwarz, 1938**

***Scaura* *amazonica* Nogueira, Oliveira & Oliveira, 2019***

**Country records**: Amazonas: La Pedrera^1^, Puerto Ñarino^1^; Boyacá: Miraflores^1^; Caquetá: Florencia^1^, San Vicente del Caguán^1^; Casanare: Chámeza^1^; Cauca: Piamonte^11^; Cundinamarca: Guayabetal^1^, Medina^1^, Paratebueno^1^; Guainía: La Guadalupe^11^, San Felipe^11^; Guaviare: Calamar^1^, San José del Guaviare^1^; Meta: Granada^1^, Villahermosa^17^, Villavicencio^1^.

***Scaura*** ***argyrea* (Cockerell, 1912)***

**Country records**: Chocó: Litoral del Bajo San Juan^1^, Nuquí^1^; Valle del Cauca: Buenaventura^16^.

***Scaura*** ***longula* (Lepeletier, 1836)***

**Country records**: Amazonas: Puerto Santander^5^; Caquetá: Florencia^1^; Casanare: Sabanalarga^5^; Cauca: Piamonte^16^; Cundinamarca: Guayabetal^1^, Medina^1^, Paratebueno^1^, Zipaquirá^1^; Guainía: Inírida^1^, La Guadalupe^16^, San Felipe^16^; Guaviare: Calamar^1^; Meta: Acacías^1^, Puerto Rico^1^, San Juan de Arama^1^, Villahermosa^18^, Villavicencio^1^; Putumayo: Mocoa^1^; Vaupés: Mitú^1^, Papunaua^1^.

***Scaura* *tenuis* (Ducke, 1916)***

**Country records**: Amazonas: La Pedrera^1^, Leticia^1^, Puerto Santander^¹^; Casanare: Sabanalarga^5^; Cundinamarca: Medina^28^; Guainía: Inírida^1^, La Guadalupe^16^, San Felipe^16^; Guaviare: Calamar^1^, San José del Guaviare^1^; Meta: La Macarena^5^, Restrepo^1^; Putumayo: Mocoa^1^; Risaralda: Mistrató^5^; Valle del Cauca: Yotoco^1^.

**Genus *Schwarzula* Moure, 1946**

***Schwarzula* *coccidophila* Camargo & Pedro, 2002***

**Country records**: Amazonas: La Pedrera^1^, Leticia^1^, Puerto Santander^1^; Cauca: Piamonte^16^; Guainía: San Felipe^16^; Guaviare: San José del Guaviare^17^.

***Schwarzula* *timida* (Silvestri, 1902)***

Country records: Amazonas: La Pedrera^1^; Caquetá: San Vicente del Caguán^1^.

**Genus *Tetragona* Lepeletier & Serville, 1828**

***Tetragona* *atahualpa* Nogueira, Rasmussen & Oliveira, 2020*§**

**Country records**: Boyacá: Buenavista† ^(Nogueira, Rasmussen & Oliveira, 2021)^

***Tetragona* *clavipes* (Fabricius, 1804)**

**Country records**: Amazonas: Tarapacá^18^; Boyacá: Santa María^2^; Caquetá: Florencia^16^; Casanare: Tauramena^2^; Cauca: Piamonte^16^, Santa Rosa^16^; Cesar: Valledupar^3^; Cundinamarca: Guayabetal^5^; Guainía: San Felipe^16^; Meta: Acacías^16,18,5^, Puerto López^2,5^, Restrepo^5,28^, San Juan de Arama^18^, San Martín^2,18^, Villavicencio^1,5,28^, Vistahermosa^5,17^; Putumayo: Puerto Asís^5^, Puerto Leguízamo^18^; Vaupés: Mitú^28^; Vichada: Cumaribo^18^.

***Tetragona* *dorsalis* (Smith, 1854)**

**Country records**: Cundinamarca: Gachalá^1^.

***Tetragona* *goettei* (Friese, 1900)**

**Country records**: Caquetá: Florencia^16^; Cauca: Piamonte^16^, Santa Rosa^16^; Vaupés: Mitú^28^.

***Tetragona*** ***handlirschii* (Friese, 1900)***

**Country records**: Amazonas: Puerto Santader^28^; Cauca: Piamonte^16^, Santa Rosa^16^.

***Tetragona*** ***perangulata*** (Cockerell, 1917)

**Country records**: Antioquia: Amalfi^13^, Caicedo^28^, Maceo^13^, San Francisco^1,18^, Santa Fé de Antioquia^2^, Sonsón^18^, Tarso^13^, Yolombó^13^; Atlántico: Juan de Acosta^6^; Caldas: Aguadas^15^, Belalcázar^28^, Chinchiná^28^, Pácora^15^, Samaná^2^; Cesar: Pueblo Bello^28^, Valledupar^28^; Chocó: Litoral del San Juan^1^; Córdoba: Tierralta^1^; Huila: Baraya^1^, Colombia^1^, Guadalupe^1^, Tello^1^; Magdalena: Santa Marta^18,6^, Zona Bananera^6^; Sucre: San Onofre^13^; Tolima: Armero^18^, Coello^2^, Honda^18^.

***Tetragona* *quadrangula* (Lepeletier, 1836)***

**Country records:** Vichada: Cumaribo^17^.

***Tetragona* *truncata* Moure, 1971***

**Country records**: Caquetá: Belén de los Andaquies^5^, Florencia^16^.

***Tetragona* *ziegleri* (Friese, 1900)***

**Country records:** Antioquia: Amalfi^13^, Cocorná^13^, San Francisco^18^, San Roque^1^, Sonsón^18^, Yolombó^13^; Bolívar: San juan Nepomuceno^17^; Chocó: Litoral del San Juan^1^; Cundinamarca: Villeta^28^; Huila: Neiva^18^; Magdalena: Aracataca^6^, Santa Marta^18,6^; Santander: Puerto Parra^1^; Tolima: El Espinal^3^, Honda^18^, Melgar^3^.

**Genus *Tetragonisca* Moure, 1946**

***Tetragonisca* *angustula* (Latreille, 1811)**

**Country records:** Amazonas: Leticia^1,2^, Mirití-Paraná^1^, Puerto Santander^1^ Solano^1^; Antioquia: Amagá^13,28^, Amalfi^28,20^, Bello^28^, Betania^1^, Caucasia^13,4,28^, Ciudad Bolívar^13,28^, Cocorná^28^, Concordia^1^, El Carmen de Viboral^1^, Envigado^28^, Fredonia^13^, Guadalupe^28^, Hispania^13^, Itagüí^28^, Jericó^1,13,28^, La Estrella^28^, La Unión^1^, Medellín^1,13,9,28^, Salgar^1^, San Francisco^1,18^, San Roque^1^, Santa Fé de Antioquia^2^, Santo Domingo^1^, Sonsón^18^, Tarso^28^, Valparaíso^28^, Venecia^28^, Yolombó^1,13,28^; Atlántico: Galapa^28^, Tubara^1^; Bolívar: San Juan Nepomuceno^1^, Turbaco^28^; Boyacá: Berbeo^1^, Buenavista^1^, Maripí^5^, Miraflores^1,28^, Pajarito^28^, Pauna^5^, Puerto Boyacá^1^, San Luis de Gaceno^1^, Santa María^1,2,28^; Caldas: Aguadas^15^, Aranzazu^28^, Belalcazar^28^, Chinchiná^1,28^, La Dorada^1,2^, La Palma^1^, Manizales^28^, Pácora^15^, Palestina^28^, Samaná^2,5^, Victoria^28^; Caquetá: Florencia^1,16^, Morelia^28^; Casanare: Chámeza^1^; Cauca: Balboa^16^, Bolívar^16^, El Tambo^28^, Guapi^18^, La Sierra^16^, Patía^16^, Piamonte^16^, Piendamó^16^, Popayán^1,16^, Totoro^16^; Cesar: La Paz^28^, Pueblo Bello^28^, Valledupar^28^; Chocó: Acandí^13^, Litoral del Bajo San Juan^1^, Quibdó^1^; Cundinamarca: Agua de Dios^7^, Alvarado^1^, Anapoima^1^, Anolaima^1^, Apulo^1^, Arbeláez^1^, El Colegio^1^, Fusagasugá^1^, Guaduas^1^, Guayabetal^1^, La Mesa^1,28^, La Vega^28^, Medina^1,28^, Nilo^1^, Nocaima^28^, Paime^1^, Pandi^28^, Puerto Salgar^1^, Quipile^1^, San Antonio del Tequendama^1,28^, Silvania^1^, Tena^1^, Tocaima^1^, Ubalá^1^, Villeta^1,28^, Zipacon^1^, Zipaquirá^1,5^, Útica^28^; Córdoba: Buenavista^28^, Montería^1^, San Antero^1^, Tierralta^1^; Guainía: La Guadalupe^16^, San Felipe^16^; Guaviare: San José del Guaviare^18^; Huila: Acevedo^1^, Baraya^1^, Campoalegre^1^, Colombia^1^, Garzón^1^, Gigante^1^, Guadalupe^1^, La Argentina^1^, Neiva^1,18,28^, Palermo^1^, Palestina^1^, Pitalito^1^, Rivera^1^, San Agustín^28^, Tarqui^28^, Tello^1^, Timana^1^, Villavieja^7^; La Guajira: Dibulla^28^, Manaure^3^; Magdalena: Aracataca^6^, Barrancas^1^, Ciénaga^1^, Córdoba^1^, Fundación^28^, Santa Marta^1,6,28^; Meta: Acacías^1^, Cumaral^1,28^, Guamal^1^, Puerto López^28^, Restrepo^1^, San Martín^2^, Villavicencio^1,28^, Vistahermosa^5^; Nariño: Tumaco^1^; Norte de Santander: Cucuta^1,28^, El Tarra^1^; Putumayo: Mocoa^1^; Quindío: Armenia^1,15,28^, Buenavista^15^, Calarcá^15,28^, Circasia^15,28^, Filandia^2^, Génova^18^, La Tebaida^1^, Montenegro^15^, Quimbaya^15,6,7,28^; Risaralda: Dosquebradas^28^, Pereira^1,28^, Pueblo Rico^13^, Santa Rosa de Cabal^1^; Santander: Betulia^28^, Bucaramanga^28^, Charalá^1^, Cimitarra^1,28^, Curití^1^, El Carmen de Chucurí^28^, Floridablanca^1,28^, Girón^1^, Lebrija^1^, Oiba^1^, Piedecuesta^5^, San Vicente de Chucurí^28^, Santa Bárbara^1^, Simacota^28^, Socorro^1,7^, Veéez^1^, Zapatoca^28^; Sucre: Ricaurte^1^, Sincelejo^28^; Tolima: Armero^18^,Cajamarca^15^,  Chaparral^3^, Coello^2^, Dolores^1^, Fresno^1^, Honda^1^, Ibagué^1,10^, Icononzo^1^, Lérida^1^, Líbano^1^, Melgar^1,28^, Prado^1^, Purificación^1^, San Sebastián de Mariquita^1,28^; Valle del Cauca: Alcalá^15^, Buenaventura^1^, Buga^15^, Bugalagrande^15^, Caicedonia^15^, Cali^1,12,3,28,20^, Candelaria^15^,El Dovio^1^, Florida^15^, La Victoria^15^, Obando^15^, Palmira^12,3,28^, Pradera^15^, San Pedro^15^, Ulloa^15^, Yotoco^1^, Zarzal^15^.

***Tetragonisca* *buchwaldi* (Friese, 1925)***

**Country records**: Chocó: Litoral del San Juan^1^.

**Genus *Trigona* Jurine, 1807**

***Trigona* *albipennis* Almeida, 1995**

**Country records**: Amazonas: La Pedrera^1^; Boyacá: Santa María^2^; Meta: La Macarena^1,5^, Puerto Rico^1^, San Martín^2^.

***Trigona* *amalthea* (Olivier, 1789)**

**Country records**: Amazonas: La Pedrera^1^, Leticia^1,28^, Puerto Santander^5^, Tarapacá^1^; Boyacá: Puerto Boyacá^1^, San Luis de Gaceno^1^; Caldas: Aguadas^15,28^, Aranzazu^15,28^, Chinchiná^3^, La Dorada^1^, Manizales^11^, Pácora^15,28^, Pensilvania^28^, Victoria^13^; Caquetá: El Doncello^16,3^, El Paujil^1^, Florencia^1,16,3^, Morelia^1^; Casanare: Villanueva^1^; Cauca: Piamonte^16^; Cundinamarca: Anolaima^1,3^, Apulo^1^, Arbeláez^1^, Fusagasugá^1,3^, Medina^1^, Nilo^1^, Pandi^1^, Paratebueno^1^, Puerto López^1^, Puerto Salgar^1^, Sasaima^1,3^; Guainía: La Guadalupe^16^; Meta: Acacías^1^, Granada^1^, Guamal^1^, La Macarena^20^, Puerto Lleras^1^, Puerto López^1^, Puerto Rico^1^, Restrepo^1^, San Juan de Arama^1^,  Villavicencio^1,3^, Vistahermosa^1^; Putumayo: Mocoa^1^.

***Trigona* *amazonensis* (Ducke, 1916)**

**Country records**: Amazonas: La Pedrera^1^, Leticia^1,2,28,20^, Puerto Santander^1^, Puerto Ñarino^1^; Caquetá: Cartagena del Chairá^1^, El Paujil^1^, Florencia^1^, La Montañita^1^, Morelia^1^; Cundinamarca: Medina^1^, Paratebueno^1,5^; Meta: Cumaral^1^, La Macarena^1,5^, Puerto Concordia^1^, Quetame^1^, Restrepo^1,5^, San Juan de Arama^20^, San Martín^2^, Villavicencio^1,5,19,28^.

***Trigona* *branneri* Cockerell, 1912**

**Country records**: Amazonas: La Pedrera^1^; Cundinamarca: Guayabetal^1^, Medina^1^, Paratebueno^1^; Guainía: Inírida^1^; Meta: Acacías^1^, Granada^1^, Puerto Gaitán^11^, Puerto López^1^, Restrepo^1^, San Martín^2^, Villavicencio^1^; Vaupés: Mitú^1^; Vichada: Cumaribo^1^.

***Trigona* *chanchamayoensis* Schwarz, 1948**

**Country records**: Amazonas: Puerto Santander^1^, Tarapacá^1^; Boyacá: Santa María^1,2^; Caquetá: Cartagena del Chaira^1^, El Paujil^1^; Cauca: Piamonte^16^; Cundinamarca: Guayabetal^1^, Medina^1^; Guaviare: San José del Guaviare^2,28^; Meta: Acacías^5^, Cumaral^1^, Granada^1^, La Macarena^1^, Puerto Rico^1^, Quetame^1^, Villavicencio^1,5^.

***Trigona*** ***cilipes* (Fabricius, 1804)**

**Country records**: Amazonas: La Pedrera^1^, Leticia^1^; Antioquia: San Francisco^18^; Cauca: Piamonte^16^; Guainía: Inírida^1,16^, San Felipe^16^; Meta: Acacias^1^, Puerto López^1^, San Martín^2^; Vichada: Cumaribo^1^.

***Trigona* *corvina* Cockerell, 1913**

**Country records**: Antioquia: Amalfi^13,28^, Andes^13^, Don Matías^1^, Gómez Plata^13^, Jardín^1^, Medellín^28^, Pueblo Rico^13^, Yolombó^13,28^; Atlántico: Juan de Acosta^28^; Bolívar: San Juán Nepomuceno^1^; Boyacá: Puerto Boyacá^1^; Chocó: Acandí^1,2^, Litoral del Bajo San Juan^1^, Unguia^1^; Cundinamarca: Pandi^1^, Sasaima^1^; Magdalena: Santa Marta^6,28^, Zona Bananera^28^; Meta: La Macarena^20^, Quetame^1^, Restrepo^1^, Villavicencio^1^; Norte de Santander: Bochalema^1^, Cucuta^1^; Risaralda: Mistrato^20^, Pueblo Rico^2^; Sucre: Sincelejo^28^.

***Trigona* *crassipes* (Fabricius, 1793)**

**Country records**: Amazonas: La Chorrera^19^, Leticia^1,2^, Puerto Santander^1^; Boyacá: Miraflores^1^; Caquetá: Florencia^1^; Cundinamarca: Paime^1^; Guainía: Inírida^1^; Meta: Acacías^1^, Cumaral^1^, Quetame^1^.

***Trigona dallatorreana* Friese, 1900**

**Country records**: Amazonas: La Chorrera^5^, La Pedrera^1^, Leticia^18,2,5,28^, Puerto Santander^1^; Caquetá: El Paujil^1^, Florencia^1^, Solano^1,28^; Cauca: Piamonte^16^; Guainía: Inírida^1^, La Guadalupe^16^, San Felipe^16^; Meta: La Macarena^1,20^, San Juan de Arama^28^; Vaupés: Mitú^28^, Yavaraté^1^.

***Trigona dimidiata* Smith, 1854**

**Country records**: Meta: Acacías^1^, Villavicencio^1^.

***Trigona ferricauda* Cockerell, 1917**

**Country records:** Antioquia: Anorí^13^, La Estrella^1^, Yarumal^13^; Chocó: Litoral del Bajo San Juan^1^, Nuquí^1^, Quibdó^1^; Cundinamarca: Anolaima^1^, Guayabetal^5^; Huila: Garzón^5^; Risaralda: Pueblo Rico^2^.

***Trigona fulviventris* Guérin-Méneville, 1845**

Country records: Antioquia: Alejandría^13^, Amagá^13^, Amalfi^1,13,28,20^, Anorí^28^, Caicedo^28^, Caramanta^28^, Caucasia^13,4,28^, Ciudad Bolívar^28^, Cocorná^13,28^, Ebejico^28^, Frontino^1^, Giraldo^8^, Jardín^13,28^, Jericó^28^, Maceo^13,9^, Medellín^1^, Necoclí^28^, Puerto Berrío^13^, Puerto Nare^13^, San Francisco^18^, San Pedro de los Milagros^5^ San Roque^1^, Santa Fé de Antioquia^2^, Santa Rosa de Osos^28^, Sonsón^18^, Yolombó^13,28^, Yondo^28^; Arauca: Tame^1^; Atlántico: Puerto Colombia^28^; Bolívar: Cartagena^1,28^, Córdoba^18^, El Carmen de Bolívar^3^, San Jacinto^18^, San Juán Nepomuceno^1^, Turbaco^28^, Zambrano^1^; Boyacá: Buenavista^1^, El Cocuy^5^, Gachantiva^28^, Miraflores^1^, Moniquirá^5^, Pajarito^1^, Puerto Boyacá^1,5^, San Mateo^1^, Zipacon^5^; Caldas: Aguadas^15,28^, Aranzazu^15,28^, Chinchiná^3^, La Dorada^1^, Manizales^28^, Pácora^15,28^, Riosucio^1^; Cauca: El Tambo^5^, Guapi^1^, La Vega^5^; Cesar: Agustín Codazzi^19,18^, Bosconia^28^, Pueblo Bello^28^, Valledupar^1,18,28^; Chocó: Acandí^1,13,2^, Riosucio^5^; Cundinamarca: Agua de Dios^7^, Anapoima^1^, Anolaima^1,3,28^, Apulo^1^, Arbeláez^1^, Cabrera^1^, Cachipay^28^, Caparrapí^1^, Chaguaní^5^, El Colegio^1^, Fusagasugá^1,3,28^, Granada^28^, Guaduas^5,1,28^, Guayabal de Síquima^28^, La Mesa^1,5,28^, La Palma^1^, La Vega^1,2,7,28^, Mosquera^1^, Nilo^1,5^, Nocaima^28^, Pacho^1,5^, Paime^1^, Pandi^1,28^, Paratebueno^1^, Puerto Salgar^1^, Quipile^1^, Ricaurte^1,13^, Sasaima^5^, San Antonio del Tequendama^1,7,28^, San Francisco^28^, San Juan de Rio Seco^1^, Sasaima^1,28^, Silvania^1,15,28^, Soacha^1^, Supata^28^, Tena^5^, Tena^1^, Tocaima^5^, Venecia^2^, Villeta^1,5,28^, Viota^1,3^, Zipaquirá^1^, Útica^1^; Córdoba: Montería^1^, San Carlos^28^, Tierralta^1^; Huila: Acevedo^1,18^, Baraya^1^, Campoalegre^1^, Colombia^1^, Garzón^1^, Gigante^1^, Guadalupe^1^, La Argentina^1^, Neiva^1,28^, Palestina^1^, Pital^28^, Pitalito^1^, Rivera^1,28^, San Agustín^15,28^, Tello^1^, Villavieja^7,28^; La Guajira: Hatonuevo^6^, San Juan del Cesar^28^; Magdalena: Aracataca^6^, Ciénaga^1^, Fundacion^28^, Guamal^5^, Pivijay^1^, Santa Marta^1,18,5,6,28^; Nariño: El Charco^1^, Tumaco^1^; Norte de Santander: Gramalote^1^, Pamplonita^8^, Toledo^18^; Putumayo: Puerto Guzman^1^; Quindío: Armenia^1,15,28^, Calarcá^15^, Circasia^15,28^, Filandia^28^, La Tebaida^15^; Risaralda: Pereira^28^; Santander: Arcabuco^28^, Barichara^28^, Barrancabermeja^28^, Bucaramanga^28^, Charalá^1,5^, Cimitarra^1,2,28^, El Carmen de Chucurí^13^, Floridablanca^1,28^, Paime^1^, Puerto Parra^1^, San Gil^28^, Simacota^20^, Socorro^1,7^, Suaita^28^, Zapatoca^28^; Sucre: Caimito^28^, Corozal^28^, Galeras^28^, Ricaurte^1^, San Luis de Sincé^28^, San Onofre^13,28^, San Pedro^28^, Sincelejo^28^; Tolima: Alvarado^10^, Armero^18,2,5^, Ataco^1^, Chaparral^1^, Coello^2^, Cunday^1^, Dolores^1^, El Espinal^3^, Honda^18,28^, Ibagué^10,5,28^, Icononzo^1^, Medina^1^, Melgar^1,2,5,28^, Pacho^1^, Prado^1^, Rovira^3^, San Luís^10^, San Sebastián de Mariquita^1,18^; Valle del Cauca: Buenaventura^1,15,20^, Dagua^20^, Palmira^12^, Pradera^15^, Río Frío^28^, Roldanillo^28^, San Pedro^28^, Tuluá^11,28^, Zarzal^28^.

***Trigona fuscipennis* Friese, 1900**

**Country records**: Antioquia: Amalfi^13,28^, Andes^13^, Barbosa^1^, Caucasia^13^, Cocorná^13^, Frontino^1^, Medellín^13^, Puerto Berrío^13^, San Francisco^1,18^, Santa Fé de Antioquia^2^, Sonsón^18^, Valparaíso^13^, Venecia^13^, Yolombó^13,28^; Bolívar: Cartagena^2^, Turbaco^20^; Boyacá: Puerto Boyacá^1,5^, San Luis de Gaceno^1^; Caldas: Chinchiná^28^, Norcasia^1^; Chocó: Acandí^1,2^, Bahía Solano^5^, Bajo baudó^5^, Litoral del Bajo San Juan^1^, Riosucio^5^; Cundinamarca: Apulo^1^, Arbeláez^1^, Fusagasugá^5^, Gachala^1^, La Mesa^1^, La Palma^1^, Medina^1^, Mosquera^5^, Nimaima^1^, Paime^1^, Pandi^1^, Puerto Salgar^1^, Quetame^1^, Sasaima^1^, Suesca^5^, Tocaima^5^, Villeta^5,28^, Zipaquirá^5^, Útica^5^; Córdoba: Tierralta^13^; Huila: Baraya^1^, Colombia^1^, Neiva^1^; La Guajira: Maicao^1^, Riohacha^1^; Magdalena: Aracataca^6^, Ciénaga^1,6^, El Banco^6^, El Retén^6^, San Sebastián de Buenavista^6^, Santa Marta^1,5,6^, Zona Bananera^6^; Nariño: Tumaco^13^; Santander: Betulia^28^, Landázuri^1^, Rionegro^13^, Simacota^20^, Zapatoca^28^; Sucre: Ricaurte^1^, San Onofre^13^; Tolima: Coello^2^, Dolores^1^, Icononzo^1^; Valle del Cauca: Buenaventura^20^.

***Trigona guianae* Cockerell, 1910**

**Country records**: Amazonas: Leticia^1,2,28^, Puerto Santander^1^; Boyacá: Santa María^2^; Casanare: Chámeza^1^, Tauramena^2^, Villanueva^1^; Cauca: Guapi^1^, Piamonte^16^, Santa Rosa^16^; Guainía: La Guadalupe^16^; Meta: Acacias^1^, La Macarena^20^, Puerto López^2^, San Martín^2^, Villavicencio^1,2^; Putumayo: Orito¹.

***Trigona hypogea* Silvestri, 1902**

**Country records**: Amazonas: Leticia^1,2^, Puerto Santander^1^; Boyacá: San Luis de Gaceno^1^; Cundinamarca: Medina^1^; Meta: Quetame^1^, Villavicencio^1^.

***Trigona muzoensis* Schwarz, 1948**

**Country records**: Antioquia: Amalfi^13,28^, Cocorná^13^, Guadalupe^28^, Gómez Plata^13^, Maceo^13^, Puerto Berrío^13^, Puerto Nare^13^, San Francisco^18^, Sonsón^18^, Yolombó^13,28^; Bolívar: El Carmen de Bolívar^18^, San Jacinto^18^; Boyacá: Muzo†^19,28^; Chocó: Acandí^13,28^, Litoral del Bajo San Juan^1^; Córdoba: Tierralta^13^; Santander: Él Carmen de Chucurí^13^, Puerto Parra^1^, Rionegro^13^.

***Trigona nigerrima* Cresson, 1878**

**Country records**: Antioquia: Amalfi^13,28^, Rionegro^20^, San Francisco^18^, San Luís^2^, Sonsón^18^, Yolombó^1,13,28^; Boyacá: Puerto Boyacá^1^, Santa Ana^2^; Cauca: Él Tambo^28^; Cesar: Pueblo Bello^28^; Chocó: Litoral del Bajo San Juan^1,28^; Cundinamarca: Caparrapí^1^, Mosquera^1^; Magdalena: Santa Marta^1,28^; Quindío: Calarcá^15^, Circasia^15^, Filandia^15^, Quimbaya^15^; Risaralda: Pueblo Rico^5^; Santander: Floridablanca^28^; Sucre: Ricaurte^1^; Tolima: Armero^2^, Dolores^1^, San Luís^1^; Valle del Cauca: Andalucía^15^, Buenaventura^11,2,20^, Dagua^20^.

***Trigona pallens* (Fabricius, 1798)**

**Country records**: Amazonas: Leticia^1,28^, Puerto Santander^1^; Caquetá: Solano^1,5^; Cundinamarca: Guayabetal^1^; Guainía: La Guadalupe^16^; Meta: Cumaral^1^, Villavicencio^5^.

***Trigona permodica* Almeida, 1995***

**Country records**: Amazonas: El Encanto^5^, Puerto Santander^5^; Caquetá: San Vicente del Caguán^1^.

***Trigona recursa* Smith, 1863**

**Country records**: Amazonas: La Pedrera^1^, Leticia^1^, Puerto Santander^1^; Antioquia: Amalfi^28^, Frontino^1^, Urrao^1^, Yolombó^28^; Boyacá: Santa María^2^; Casanare: Tauramena^2^; Meta: La Macarena^5^, Puerto Rico^1^, San Martín^2^, Villavicencio^1,2^.

***Trigona silveirai* Oliveira, Engel, Rasmussen, Feitosa-Ribeiro & Urban, 2024***

**Country records**: Antioquia: Amalfi^13^, Maceo^13^, Yolombó^13^; Guainía: San Felipe^16^; Valle del Cauca: Buenaventura†^20^.

***Trigona silvestriana* (Vachal, 1908)**

**Country records**: Antioquia: Frontino^1^, Jardín^28^; Boyacá: Santa María^2^; Caldas: La Dorada^1^; Chocó: Acandí^1,2^, Litoral del Bajo San Juan^1,28^, Lloró^1^, Nuquí^1^, Quibdó^28^; Cundinamarca: Anolaima^1^, Apulo^1^, Pacho^1^, Paratebueno^1^, Puerto Salgar^1^, Tena^1^, Villeta^1^, Viotá^1^; Magdalena: Santa Marta^28^; Nariño: Barbacoas^1^; Risaralda: Pueblo Rico^2^; Santander: Landázuri^1^; Tolima: Armero^2^; Valle del Cauca: Buenaventura^1,11,2^, Calima^1^, Dagua^11^, Cali^11^.

***Trigona truculenta* Almeida, 1985**

**Country records**: Amazonas: El Encanto^5^, La Chorrera^5^, La Pedrera^1^, Leticia^1,18,2^, Puerto Santander^1^; Caquetá: Albania^1^, El Paujil^1^, Florencia^1^, Solano^18^, Valparaíso^1^; Casanare: Sabanalarga^5^; Cauca: Miranda^11^, Patía^1^; Meta: Cumaral^1^, La Macarena^1,5,20^, Mesetas^20^, Puerto López^1^, Puerto Rico^1^, Restrepo^5^, San Martín^2^, Villavicencio^1,5^; Vaupés: Mitú^5^; Vichada: Cumaribo^1,5,18^.

***Trigona williana* Friese, 1900**

**Country records**: Amazonas: Leticia^18,28^, Puerto Santander^1,5^, Tarapacá^1^; Caquetá: Florencia^16,28^, Morelia^1^, Solano^5^; Casanare: Villanueva^28^; Cauca: Piamonte^16^; Cundinamarca: Medina^1,28^; Guainía: Inírida^1,16^, La Guadalupe^16^, San Felipe^16^; Guaviare: El Retorno^18^, San José del Guaviare^5,28^; Meta: La Macarena^1,18,5,20^, Puerto López^1^, San Juan de Arama^18,5,28,20^, Villavicencio^1,2,28^, Vistahermosa^1,18,5^; Vichada: Cumaribo^18^.

**Genus *Trigonisca* Moure, 1950**

***Trigonisca buyssoni* (Friese, 1902)**

**Country records**: Antioquia: Amalfi^28^, San Francisco^18^, Sonsón^18^, Yolombó^28^; Cauca^(Moure, Urban & Melo, 2022)^

***Trigonisca ceophloei* (Schwarz, 1938)***

**Country records**: Guainía: La Guadalupe^16^, San Felipe^16^.

***Trigonisca graeffei* (Friese, 1900)**

**Country records**: No specific location records

***Trigonisca mepecheu* Engel & Gonzelez, 2019***

**Country records**: La Guajira: Maicao^28^, Manaure†^1,5,28^, Uribia^1^.

***Trigonisca schulthessi* (Friese, 1900)**

**Country records**: Antioquia: Amalfi^28^, Yolombó^28^.

**Eucerinae Latreille, 1802**

**Ancyloscelidini Roig-Alsina & Michener, 1993**

**Genus *Ancyloscelis* Latreille, 1829**

***Ancyloscelis apiformis* (Fabricius, 1793)**

**Country records**: Antioquia: Amalfi^28^; Bolívar: Cartagena^28^; Cundinamarca: Agua de Dios^7^; Huila: Villavieja^7^; Magdalena: San Sebastián de Buenavista^28^, Santa Marta^28^; Santander: Enciso^7^.

***Ancyloscelis hertigi* (Michener, 1954)***

**Country records**: Huila: Villavieja^7^

**Emphorini Robertson, 1904**

**Genus *Diadasia* Patton, 1879**

**Subgenus *Dasiapis* Cockerell, 1903**

***Diadasia* *(Dasiapis)* *tropicalis* (Cockerell, 1918)**

Country records: Bolívar: Zambrano^20^; Huila: Villavieja^7^; La Guajira: Maicao^20^, Manaure^20^; Magdalena: Aracataca^6^, El Retén^6^, Fundación^6^, Santa Marta^6,28^; Tolima: Coello^2^, Cunday^2^.

**Genus *Melitoma* Lepeletier & Serville, 1828**

***Melitoma segmentaria*** (Fabricius, 1804) *

Country records: Atlántico: Barranquilla^3^; Caldas: Chinchiná^3^; Cesar: Aguachica^28^; Cundinamarca: Agua de Dios^7^; Huila: Villavieja^7,20^; La Guajira: Maicao^3^; Magdalena: Aracataca^6^, El Retén^6^, San Sebastián de Buenavista^6^, Santa Marta^6^; Santander: Bucaramanga^3^, Socorro^7^; Tolima: Cunday^20^.

**Genus *Melitomella* Roig-Alsina, 1999**

***Melitomella schwarzi* (Michener, 1954)**

**Country records**: Bolívar: El Carmen de Bolívar^3^, Zambrano^1^; Huila: Villavieja^7,28^; Magdalena: Aracataca^6^, El Retén^6^, Santa Marta^1,6,28^; Santander: Enciso^7^.

**Genus *Ptilothrix* Smith, 1853**

**Country records**: Magdalena: Aracataca^6^, Santa Marta^6^.

**Eucerini Latreille, 1802**

**Genus *Florilegus* Robertson, 1900**

**Subgenus *Florilegus* Robertson, 1900**

***Florilegus* *(Florilegus)* *condignus* (Cresson, 1878)**

**Country records**: Magdalena: Pueblo Bello^28^.

***Florilegus* *(Florilegus)* *flavohirtus* Urban, 1970**

**Country records**: Bolívar: Cartagena†^Urban, 1970^; La Guajira: Manaure^28^

**Subgenus *Floriraptor* Moure & Michener, 1955**

***Florilegus* *(Floriraptor)* *melectoides* (Smith, 1879)**

**Country records**: Amazonas: La Pedrera^1^; Bolívar: San Juan Nepomuceno^1^, Zambrano^20^; Cundinamarca: Puerto Salgar^3^; Meta: La Macarena^1^.

**Genus *Gaesischia* Michener, LaBerge & Moure, 1955**

**Country records**: Amazonas: Leticia^20^.

**Genus *Lophothygater* Moure & Michener, 1955**

***Lophothygater* cf*. decorata***

**Country records**: Antioquia: Amalfí ^(Gonzalez & Ospina, 2006)^

**Note**: It was not possible to corroborate this record, despite an exhaustive search, the specimens reported by Gonzalez & Ospina (2006) were not located in the MEFLG collection.

**Genus *Melissodes* Latreille, 1829**

**Subgenusi *Ecplectica* Holmberg, 1884**

***Melissodes* (*Ecplectica*) cf. *sexcinctus***

**Country records**: Cundinamarca: La Vega^7^; Huila: Villavieja^7^.

**Subgenus *Melissodes* Latreille, 1829**

***Melissodes* *(Melissodes)* *rufodentatus* Smith, 1854***

**Country records**: Cauca: Corinto^28.^; César: Pueblo Bello^28.^; La Guajira: Manaure^20.^; Valle del Cauca: La Cumbre^28^.

**Genus *Melissoptila* Holmberg, 1884**

**Country records**: Valle del Cauca: Cali^20^, Dagua^20.^

**Genus *Platysvastra* Moure, 1967**

***Platysvastra macraspis* Moure, 1967***

**Country records**: Amazonas: Leticia^2^.

**Genus *Thygater* Holmberg, 1884**

**Subgenus *Nectarodiaeta* Holmberg, 1903**

***Thygater*** ***(Nectarodiaeta)* *melanotricha* Urban, 1967**

**Country records**: Magdalena: Cienaga^(Urban, 1967)^.

***Thygater*** ***(Nectarodiaeta) nigropilosa* Urban, 1967 §**

**Country records**: Valle del Cauca: Cali†^19^.

**Subgenus *Thygater* Holmberg, 1884**

***Thygater* *(Thygater) aethiops* (Smith, 1854)**

**Country records**: Antioquia: Envigado^28^, La Unión^6,28^, Medellín^6,28^, Sonsón^28^; Boyacá: Aquitania^1^, Nuevo Colón^1^, Sotaquira^1^, Ventaquemada^1,18^, Villa de Leyva^1^; Casanare: Chámeza^1^, Maní^1^; Cauca: Inzá^1^, Popayán^28^, Puracé^28^; Cundinamarca: Anolaima^1^, Barbosa^3^, Bogotá, D.C.^1,2,7,28^, Bojacá^1^, Cajicá^7^, Choachí^1,7^, Chía^28^, Cogua^7^, Cota^1,28^, El Rosal^1^, Facatativá^1,28^, Funza^28^, Guasca^7,28^, La Calera^1^, Madrid^1^, Mosquera^1,3,5,28^, Murillo^1^, Nilo^5^, San Antonio del Tequendama^7^, Sopó^7^, Subachoque^7^, Tabio^28^, Tenjo^7,28^, Tocancipá^7^, Zipaquirá^1,28,20^; Huila: Algeciras^1^, Hobo^1^, Neiva^28^; Magdalena: Ciénaga^6^, Santa Marta^6^, Quindío: Armenia^28^, Calarcá^15^; Santander: Él Peñón^9^; Valle del Cauca: Él Cerrito^11^.

***Thygater* *(Thygater) analis* (Lepeletier, 1841)**

**Country records**: Antioquia: Envigado^28^, Itagüí^28^, Medellín^28^; Casanare: Chámeza^1^; Cauca: Popayán^28^; Huila: La Argentina^1^; Magdalena: Santa Marta^1^; Valle del Cauca: Él Dovio^1^, Cali^11^, Tuluá^11^.

***Thygater* *(Thygater) cockerelli* (Crawford, 1906)**
**Country records**: Antioquia: Frontino^1^; Boyacá: Puerto Boyacá^1.^

***Thygater* *(Thygater) colombiana* Urban, 1967**

**Country records**: Cauca: Popayán†^19,(Urban, 1967)^; Quindío: Armenia^15^, Calarcá^15^; Valle del Cauca: Andalucíaa^15^, Cali^1^.

***Thygater* *(Thygater)*** ***crawfordi* Urban, 1967**

**Country records**: Antioquia: Amalfi^28^, Yolombó^28^; Cauca: Popayán^28^; Valle del Cauca: Cali^28^.

**Genus *Xenoglossa* Smith, 1854**

**Subgenus *Peponapis* Robertson, 1902**

***Xenoglossa* *(Peponapis) citrullina* (Cockerell, 1912)**

**Country records**: Cundinamarca: Girardot^7^; Cauca: Patía^28^; Huila: Suaza^28,20^.

**Exomalopsini Vachal, 1909**

**Genus *Exomalopsis* Spinola, 1853**

**Subgenus *Exomalopsis* Spinola, 1853**

***Exomalopsis* *(Exomalopsis) aburraensis* Velez-Ruiz & Smith-Pardo, 2013***

**Country records**: Antioquia: Medellín†^28, 20^.; Caldas: Chinchiná^28^; Cauca: El Tambo^28^.; César: Pueblo Bello^28^.; Quindío: Buenavista^28^.

***Exomalopsis* *(Exomalopsis) analis* Spinola, 1853***

**Country records**: Magdalena: Santa Marta^28^.; Meta: Puerto Gaitán^1.^

***Exomalopsis* *(Exomalopsis) digressa* Timberlake, 1980**

**Country records**: Meta: Villavicencio^(Timberlake, 1980)^.

***Exomalopsis* *(Exomalopsis) similis* Cresson, 1865**

**Country records**: Valle del Cauca: Palmira^28.^

***Exomalopsis* *(Exomalopsis) subtilis* Timberlake, 1980***

**Country records**: Cundinamarca: Quetame^1^.

**Subgenus *Phanomalopsis* Michener & Moure, 1957**

***Exomalopsis* *(Phanomalopsis) griswoldi* Silveira & Almeida, 2009***

**Country records**: Cundinamarca: Agua de Dios^7^; Huila: Villavieja^7,28^; Magdalena: Santa Marta†^20,^ (Silveira & Almeida, 2009); Santander: Socorro^7^.

***Exomalopsis* *(Phanomalopsis) snowi* Cockerell, 1906**

**Country records**: Quindío: Buenavista^28.^

***Exomalopsis* *(Phanomalopsis)* cf. *trifasciata***

**Country records**: Huila: Villavieja^28.^

**Tapinotaspidini Roig-Alsina & Michener, 1993**

**Genus *Lophopedia* Michener & Moure, 1957**

***Lophopedia* *apicalis* (Cresson, 1878)***

**Country records**: Cauca: Guapi†^(Cheesman, 1929)^.

***Lophopedia* *flava* (Smith, 1879)***

**Country records**: Amazonas: Leticia; Boyacá: Muzo^(Aguiar, 2009)^; Chocó: Bahía Solano^(Aguiar, 2009)^.

***Lophopedia* *haeckeli* (Friese, 1910)**

**Country records**: Cauca: Popayán^(Aguiar, 2009)^.

***Lophopedia*** ***klugi* (Friese, 1899)***

**Country records**: Cesar: Agustín Codazzi^(Aguiar, 2009)^; Cauca: Popayán^(Aguiar, 2009)^.

***Lophopedia*** ***pygmaea* (Schrottky, 1902)***

**Country records**: Antioquia: Puerto Triunfo^(Aguiar, 2009)^; Cauca: Popayán^(Aguiar, 2009)^

**Genus *Monoeca* Lepeletier & Serville, 1828**

**Country records**: Meta: Puerto Rico^1^; Sucre: Ricaurte^1^.

**Genus *Nasutopedia* Aguiar & Melo, 2011**

***Nasutopedia* *borealis* Aguiar, 2018***

**Country records**: Cundinamarca: Tena^1^; Magdalena: Santa Marta†^(Aguiar, 2009)^ ;Risaralda: Pereira^1^; Tolima: Ibagué^1^.

***Nasutopedia* *micheneri* Aguiar & Melo, 2011***

**Country records**: Cundinamarca: San Antonio del Tequendama^7^.

***Nasutopedia* *nigripes* (Friese, 1899)***

**Country records**: Risaralda: Pereira^13^.

***Nasutopedia*** ***puncticutis* Aguiar, 2018***

**Country records**: Risaralda: Pereira^1^.

**Genus *Paratetrapedia* Moure, 1941**

***Paratetrapedia* *calcarata* (Cresson, 1878)**

**Country records**: Antioquia: Amalfi^28^, Santa Fe de Antioquia^13,4,28^, Sonsón^18^, Yolombó^28^; Cesar: Valledupar^28^; Cundinamarca: Anapoima^1^; Huila: Neiva^1^, Tello^1^; Magdalena: Aracataca^6^, Santa Marta^6,28^; Valle del Cauca: Dagua^20^, Palmira^1^.

***Paratetrapedia*** ***chocoensis* Aguiar & Melo, 2011***

**Country records:** Magdalena: Santa Marta^6^; Valle del Cauca: Buenaventura^20^.

***Paratetrapedia*** ***connexa* (Vachal, 1909)***

**Country records**: Santander: Puerto Parra^28^.

***Paratetrapedia* *lineata* (Spinola, 1853)**

**Country records**: Risaralda San Juan de Arama^20^.

***Paratetrapedia*** ***lugubris* (Cresson, 1878)**

**Country records**: Amazonas: Leticia^1^; Magdalena: Santa Marta^1^; Meta: Restrepo^1^.

**Genus *Tapinotaspoides* Moure, 1944**

***Tapinotaspoides* *septentrionalis* Melo & Aguiar, 2008***

**Country records**: Bolívar: Córdoba^18^, Zambrano^1^.; Magdalena: El Retén^6^.

**Nomadinae Latreille, 1802**

**Epeolini Linsley & Michener, 1939**

**Genus** ***Epeolus* Latreille, 1802**

***Epeolus*** ***boliviensis* Friese, 1908***

**Country records**: Bolívar: San Juan Nepomuceno^1^.

***Epeolus claripennis* Friese, 1908***

**Country records**: Quindío: Filandia^2^.

***Epeolus luteipennis* Friese, 1916.***

**Country records:** Huila: Villa Vieja^7^

**Genus *Odyneropsis* Schrottky, 1902**

**Subgenus *Parammobates* Friese, 1906**

***Odyneropsis* *(Parammobates)* *batesi* Cockerell, 1916***

**Country records**: Boyacá: Santa María^2^.

***Odyneropsis* *(Parammobates)* *columbiana* Schrottky, 1920**

**Country records**: Cundinamarca: Guayabetal†^(Schrottky, 1920; Moure, Urban & Melo, 2022)^; Valle del Cauca: Buenaventura^18^.

**Genus *Thalestria* Smith, 1854**

***Thalestria*** ***spinosa* (Fabricius, 1804)**

**Country records**: Meta: San Juan de Arama^28^, Villavicencio^3,28^

**Genus *Triepeolus* Robertson, 1901**

***Triepeolus* *flavipennis* (Friese, 1917)**

**Country records**: Cauca: Popayán†^(Onuferko et al., 2024)^; Cundinamarca: Guayabetal^(Onuferko et al., 2024)^; Valle del Cauca: Dagua^(Onuferko et al., 2024)^.

***Triepeolus* *nemoralis* (Holmberg, 1886)***

**Country records:** Antioquia: San Vicente^14^; Bolívar: Zambrano^(Onuferko et al., 2024)^; Cesar: Valledupar^(Onuferko et al., 2024)^;  Huila: Acevedo^1^; Meta: Puerto López^(Onuferko et al., 2024)^; Valle del Cauca: Cali^(Onuferko et al., 2024)^, Dagua^(Onuferko et al., 2024)^.

***Triepeolus* *osiriformis* (Schrottky, 1910)***

Country records: Cauca: Tierradentro^14^; Valle del Cauca: ^(Onuferko et al., 2024)^, Candelaria^(Onuferko et al., 2024)^.

***Triepeolus*** ***rufotegularis* (Ashmead, 1900)**

**Country records**: Cesar: Valledupar^(Onuferko et al., 2024)^; Magdalena: Santa Marta^(Onuferko et al., 2024)^.

***Triepeolus*** ***tuberculifer*** **Onuferko, Rightmyer & Roig-Alsina 2024**

**Country records**: Bolívar: Zambrano†^(Onuferko et al., 2024)^; Casanare: Orocué^(Onuferko et al., 2024)^; Huila: Villavieja^7^; Magdalena: El Retén^6^; Meta: Puerto Gaitán^(Onuferko et al., 2024)^.

**Ericrocidini Cockerell & Atkins, 1902**

**Genus *Acanthopus* Klug, 1807**

***Acanthopus* *palmatus* (Olivier, 1789)**

**Country records**: Bolívar: Córdoba^18^, Zambrano^18^; Casanare: Yopal^1^; Cundinamarca: Nocaima^28^, Tena^28^; Huila: Neiva^18^; Magdalena: Santa Marta^1^; Sucre: San Onofre^13^.

**Genus *Ctenioschelus* Romand, 1840**

***Ctenioschelus*** ***goryi* (Romand, 1840)**

**Country records**: Antioquia: Jardín^28^; Santander: Los Santos^28^.

**Genus *Mesocheira* Lepeletier & Serville, 1825**

***Mesocheira*** ***bicolor*** (Fabricius, 1804)

**Country records**: Amazonas: Puerto Santander^1^; Antioquia: Envigado^14^, Medellín^14^, Yolombó^28^; Bolívar: Calamar^14^, Córdoba^18^, Zambrano^1,18^; Cesar: Valledupar^28^; Cundinamarca: La Mesa^1^; Huila: Villavieja^7^; Magdalena: El Retén^6,28^, Santa Marta^1,18^; Meta: Puerto López^20^; Santander: Cimitarra^28^; Valle del Cauca: Cali^17^, Dagua^20^, Palmira^3,20^.

**Genus *Mesoplia* Lepeletier, 1841**

**Subgenus *Eumelissa* Snelling & Brooks, 1985**

***Mesoplia* *(Eumelissa)* *friesei* (Ducke, 1902)***

**Country records:** Huila: Villavieja^7^.

***Mesoplia* *(Eumelissa)* *guedesii* (Ducke, 1902)**

**Country records**: Amazonas: Leticia^28^.

***Mesoplia (Eumelissa)* *regalis* (Smith, 1854)***

**Country records**: Meta: Vista Hermosa^18^.

**Subgenus *Mesoplia* Lepeletier, 1841**

***Mesoplia* *(Mesoplia)* *rufipes* (Perty, 1833)***

**Country records**: Amazonas: Leticia^18^; Antioquia: Yolombó^28^; Huila: Villavieja^7^; Magdalena: Santa Marta^18^.

***Mesoplia* *(Mesoplia)* *sapphirina* Melo & Rocha-Filho, 2011***

**Country records**: Huila: Villavieja^7^.

**Isepeolini Rozen, Eickwort & Eickwort, 1978**

**Genus *Isepeolus* Cockerell, 1907**

***Isepeolus* *viperinus* (Holmberg, 1886)**

**Country records**: Antioquia: Yolombó^28^; Valle del Cauca: Cali^28^.

**Nomadini Latreille, 1802**

**Genus *Nomada* Scopoli, 1770**

***Nomada* *advena* Smith, 1860 §**

**Country records**: Valle del Cauca: Cali^(Odanaka et al., 2022).^

***Nomada*** ***medellinensis*** Velez-Ruiz & Smith-Pardo, 2013 §*

**Country records**: Antioquia: Medellín†^19, 28^.

**Osirini Handlirsch, 1925**

**Genus *Osiris* Smith, 1854**

***Osiris*** ***analis*** **Friese, 1930***

**Country records**: Amazonas: La Chorrera^(Shanks, 1986)^; Caquetá: Florencia^(Shanks, 1986)^; Putumayo: Mocoa^(Shanks, 1986)^.

***Osiris* *atriventris* Friese, 1930**

**Country records**: Amazonas: La Chorrera^(Shanks, 1986)^; Putumayo: Mocoa^(Shanks, 1986).^

***Osiris* *barrocoloradensis* Schwarz, 1934**

**Country records**: Antioquia: Yolombó^28^; Putumayo: Mocoa^(Shanks, 1986)^.

***Osiris* *boliviensis* Friese, 1930**

**Country records**: Meta: La Macarena^(Shanks, 1986)^.

***Osiris* *fasciatus* (Radoszkowski, 1884)**

**Country records**: Cesar: Bosconia^28^.

***Osiris*** ***marginatus* Cresson, 1878**

**Country records**: Meta: La Macarena^(Shanks, 1986)^.

***Osiris* *mourei* Michener, 1954**

**Country records**: Antioquia: Yolombó^28^; Nariño: Pueblo Rico^(Shanks, 1986)^; Putumayo: Mocoa^(Shanks, 1986)^.

***Osiris*** ***panamensis* Cockerell, 1919**

**Country records**: Antioquia: Amalfi^28^, Yolombó^28^.

***Osiris*** ***stenolobus* Shanks, 1986**

**Country records**: Magdalena: Santa Marta^(Shanks, 1986)^.

***Osiris*** ***tarsatus* Smith, 1879***

**Country records**: Putumayo: Mocoa^(Shanks, 1986)^.

**Genus *Protosiris* Roig-Alsina, 1989**

***Protosiris*** ***obtusus* (Michener, 1954)**

**Country records**: Antioquia: Yolombó^28^

**Protepeolini Linsley & Michener, 1939**

**Genus *Leiopodus* Smith, 1854**

***Leiopodus* *lacertinus* Smith, 1854**

**Country records:** Casanare: Trinidad^1^; Huila: Villavieja^7^; Vichada: Cumaribo^18^.

***Leiopodus*** ***nigripes*** **Friese, 1908**

**Country records:** La Guajira: Uribia^19,28^

**Rhathymini Lepeletier, 1841**

**Rhathymus Lepeletier & Serville, 1828**

***Rhathymus*** ***bicolor*** **Lepeletier & Audinet-Serville, 1828**

Country records: Antioquia: Ciudad Bolívar^13^; Santander: Rionegro^13^.

***Rhathymus*** ***scoliaeformis*** **Schrottky, 1920 §***

Country records: Cundinamarca: Guayabetal† ^(Schrottky, 1920; Moure, Urban & Melo, 2022)^.

**Xylocopinae Latreille, 1802**

**Ceratinini Latreille, 1802**

**Genus *Ceratina* Latreille, 1802**

**Subgenus *Calloceratina* Cockerell, 1924**

***Ceratina* *(Calloceratina)* cf. *belizensis***

**Country records**: Antioquia: Pueblorrico^(Mahlmann, 2023)^.

***Ceratina* *(Calloceratina)* *chloris* (Fabricius, 1804)**

**Country records**: Antioquia: Amalfi^28^, Yolombó^28^; Santander: Girón^28^; Valle del Cauca: Dagua^20^.

***Ceratina* *(Calloceratina)* *eximia* Smith, 1862**

**Country records**: Antioquia: Yolombó^28.^

***Ceratina* *(Calloceratina)* *mourei* Mahlmann & Oliveira, 2023 §***

**Country records**: Amazonas: Tarapacá† ^(Mahlmann, Oliveira & Oliveira, 2023).^

***Ceratina* *(Calloceratina)* *silveirai* Mahlmann & Oliveira, 2023 §***

**Country records**: Putumayo: Villagarzón†^(Mahlmann, Oliveira & Oliveira, 2023)^.

***Ceratina* *(Calloceratina)* *triangulifera* Cockerell, 1914***

**Country records**: Huila: Villavieja^7^; Valle del Cauca: Palmira^3^.

**Subgenus *Ceratinula* Moure, 1941**

***Ceratina* *(Ceratinula)* *tricolor* Michener, 1954**

**Country records**: Cesar: Becerril^28^, Valledupar^28^; Huila: Villavieja^7^; Magdalena: Santa Marta^28^; Sucre: Toluviejo^28^.

***Ceratina* *(Ceratinula)* *trimaculata* Friese, 1917***

**Country records**: Antioquia: Yolombó^28^.

***Ceratina* *(Ceratinula)* *virescens* Friese, 1910***

**Country records**: Valle del Cauca: Cali^28,20^, Dagua^20.^

***Ceratina* *(Ceratinula)* *zeteki* Cockerell, 1934**

**Country records**: Antioquia: Yolombó^28^, Huila: Villavieja^7^

**Subgenus *Crewella* Cockerell, 1903**

***Ceratina* *(Crewella)* *dalyana* Roig-Alsina, 2022 §***

**Country records**: Atlántico: Barranquilla†^(Roig-Alsina, 2022)^.

***Ceratina* *(Crewella)* *lehmanni* Friese, 1910 §**

**Country records**: Cauca: Popayán†^(Friese, 1910)^

***Ceratina* *(Crewella)* *maculifrons* Smith, 1854***

**Country records**: Meta: Restrepo^(Roig-Alsina, 2022)^.

***Ceratina* *(Crewella)* *placida* Smith, 1862**

**Country records**: Antioquia: Amalfi^28^, Yolombó^28^; Huila: Villavieja^7^.

**Subgenus *Rhysoceratina* Michener, 2000**

***Ceratina* *(Rhysoceratina)* *macondiana* Flórez-Gómez & Griswold, 2020**

Country records: Atlántico: Barranquilla†^(Flórez-Gómez & Griswold, 2020)^; Bolívar: San Juan Nepomuceno^1^, Zambrano^1^.; Magdalena: Aracataca^6^, El Retén^6^, Santa Marta^6^.

***Ceratina* *(Rhysoceratina)* *nitidifrons* Roig-Alsina, 2016 §**

**Country records**: Meta: Villavicencio†^(Roig Alsina, 2016)^.

**Subgenus *Zadontomerus* Ashmead, 1899**

***Ceratina* *(Zadontomerus)* *nautlana* Cockerell, 1897***

**Country records**: Antioquia: Porce^(Flórez-Gómez et al., 2022)^; Atlántico: Puerto Colombia^1^; Caldas: Pensilvania^28^; Cesar: Valledupar^(Flórez-Gómez et al., 2022)^; Cundinamarca: La Mesa^1^, La Vega^1^; Huila: Acevedo^1^, Neiva^1^, Villavieja^7^; Magdalena: , Santa Marta^6, (Flórez-Gómez et al., 2022)^.

**Tetrapediini Michener & Moure, 1957**

**Genus *Tetrapedia* Klug, 1810**

***Tetrapedia* *clypeata* Friese, 1899**

**Country records**: Cundinamarca: Bogotá, D.C^(Friese, 1899b)^.

Note: According to Friese (1899b) and Moure, Urban & Melo (2022), one of the syntypes bears the following label information: “von Bogota (Lindig).” It is likely that this specimen originates from a locality other than the city of Bogotá, within the department of Cundinamarca, as also proposed by Melo & Liz (2025) for the holotype of *Habralictus xanthogastris* (Vachal, 1911).

**Xylocopini Latreille, 1802**

**Genus *Xylocopa* Latreille, 1802**

**Subgenus *Neoxylocopa* Michener, 1954**

***Xylocopa* *(Neoxylocopa)* *aeneipennis* (de Geer, 1773)**

Country records: Amazonas: Leticia^12^; Antioquia: Maceo^13^, San Francisco^18^, Titiribí^13^; Casanare: Villanueva^1^; Cauca: Miranda^12^, Santander de Quilichao^12^; Chocó: Acandí^2^; Meta: Puerto Rico^1^, San Juan de Arama^1^; Nariño: Santa Bárbara^11^; Quindío: Armenia^15^, Calarcá^15^, Pijao^15^, Quimbaya^15^; Santander: Piedecuesta^13^; Tolima: Honda^18,28^, San Sebastián de Mariquita^2^; Valle del Cauca: Buenaventura^11,12^, Cali^11,12^, Candelaria^12^, El Cerrito^12^, Florida^12^, Ginebra^12^, Jamundí^11^, Palmira^12^, Yotoco^11^.

***Xylocopa* *(Neoxylocopa)* cf. *amazonica***

**Country records**: Amazonas: Leticia^1^; Meta: San Juan de Arama^1^.

***Xylocopa* *(Neoxylocopa)* *aurulenta* (Fabricius, 1804)**

**Country records:** Amazonas: Leticia^1^; Caquetá: Florencia^1^; Meta: Granada^1^, La Macarena^1,18^, Puerto Gaitán^1^, Vistahermosa^18^; Vichada: Cumaribo^1,18^.

***Xylocopa* *(Neoxylocopa)* cf. *brasilianorum***

**Country records:** Meta: San Juan de Arama^1^; Valle del Cauca: Dagua^1^.

***Xylocopa* *(Neoxylocopa)* cf. *carbonaria***

**Country records:** Boyacá: San Luis de Gaceno^1^; Cundinamarca: Medina^1^; Guaviare: El Retorno^18^; Meta: Puerto Gaitán^1^, La Macarena^1^, Puerto López^1^, Puerto Rico^1^, San Juan de Arama^1,18^, San Vicente del Caguán^1^, Villavicencio^1^; Valle del Cauca: Yotoco^1^.

***Xylocopa* *(Neoxylocopa)* *fimbriata* Fabricius, 1804**

**Country records**: Antioquia: Andes^13^, Caceres^13^, Caucasia^13^, Ciudad Bolívar^28^, Copacabana^28^, Granada^13^, Medellín^13,28^, San Juan de Urabá^28^, Santa Fé de Antioquia^5^, Sopetrán^28^, Yolombó^13^; Atlántico: Barranquilla^5^, Juan de Acosta^28^; Bolívar: Arjona^5^, Cartagena^1,5,28^, Córdoba^18^, El Carmen de Bolívar^18,28^, Hatillo de Loba^18^, Santa Ana^1^, Turbaco^28^, Zambrano^1,18^; Boyacá: Moniquirá^5^, Tipacoque^1^; Caldas: Chinchiná^28^, Manizales^28^; Casanare: Aguazul^7^, Orocué^5^, Paz de Ariporo^18^, Trinidad^1^, Villanueva^1,28^, Yopal^28^; Cesar: Chimichagua^5^, Valledupar^5,6,28^; Cundinamarca: El Colegio^28^, Nilo^5^, Viotá^5^, Zipaquirá^5^; Córdoba: Lorica^28^, Montería^1^, Tierralta^13^; Huila: Gigante^5^, Palermo^1^; La Guajira: Uribia^19,28^; Magdalena: Aracataca^6^, Ariguaní^18,6^, Chivolo^6^, Ciénaga^6,28^, El Piñón^28^, El Retén^6^, Plato^18^, Santa Marta^1,18,6,28^, Zona Bananera^6,28^; Meta: Acacías^5^, Cubarral^5^, Cumaral^1^, El Castillo^1^, Granada^1^, Puerto Gaitán^1^, Puerto López^1,2,5,20^, Restrepo^1^, San Vicente del Caguán^1^, Villavicencio^1,3,5^; Norte de Santander: Bochalema^1^, Villa del Rosario^1^; Santander: Bucaramanga^28^; Sucre: Galeras^28^, San Onofre^1,13,9^, Sincelejo^1,28^; Tolima: San Sebastián de Mariquita^5^; Valle del Cauca: Ginebra^28^, Palmira^28^, Cali^11^.

***Xylocopa* *(Neoxylocopa)* *frontalis* (Olivier, 1789)**

**Country records:** Amazonas: Leticia^1^, Mirití-Paraná^1^, Puerto Santander^5^; Antioquia: Bello^28^, Caucasia^13^, Copacabana^5^, Envigado^9,28^, Gómez Plata^13^, Maceo^13^, Medellín^1,13,9,28^, Puerto Nare^13^, San Luís^2^, Sopetrán^28^, Támesis^13^; Arauca: Arauca^28^, Tame^28^; Atlántico: Barranquilla^5^; Bolívar: Cartagena^2,28^, San Juán Nepomuceno^1^; Boyacá: Miraflores^1,5^, Moniquirá^5^, Puerto Boyacá^1^, Tipacoque^1^; Caldas: Chinchiná^28^, La Dorada^28^, Topaipi^1^, Viterbo^1^; Caquetá: Florencia^5,28^, Miraflores^18^, San Vicente del Caguán^5^; Casanare: Tauramena^13^, Trinidad^1,2^, Villanueva^1,28^, Yopal^13^; Cauca: Mercaderes^12^, Popayán^12^, Santander de Quilichao^1,11,17^; Cesar: Valledupar^28^; Chocó: Acandí^13^, Lloró^5^, Nuquí^13^, Riosucio^5^; Cundinamarca: Apulo^1,5^, El Colegio^1^, Funza^1,3^, Fusagasugá^1,5^, Guaduas^1,3^, La Mesa^1^, Nilo^1,7^, Nimaima^1^, Pacho^1^, Pandi^1^, Puerto Salgar^1^, Sasaima^1,28^, Silvania^5^, Tocaima^1,5^, Villeta^1,5^, Zipaquirá^1,5^; Guaviare: San José del Guaviare^18^; Huila: Algeciras^1^, Colombia^1^, Garzón^5^, Gigante^1,5^, La Argentina^1^, La Plata^11^, Neiva^1,18^, Rivera^1^, San Agustín^5^, Teruel^1^, Villavieja^7^; La Guajira: Dibulla^28^; Magdalena: Aracataca^6^, Ciénaga^28^, El Retén^6^, Santa Marta^1,15,18,5,6,28^, Zapayan^1^, Zona Bananera^6,28^; Meta: Barranca de Upía^1^, Cumaral^2,5^, Puerto López^1,2,5,20^, Puerto Rico^1^, San Juan de Arama^1,5^, San Vicente del Caguán^1^, Villavicencio^1,3,5^, Vistahermosa^18^; Nariño: Los Andes^11,17^; Norte de Santander: Bucarasica^13^; Quindío: Armenia^12,15^, Buenavista^28^, Calarcá^15^, Filandia^15^, La Tebaida^15^, Quimbaya^15,7^, Salento^15^; Risaralda: Pereira^28^; Santander: Barichara^2^, Charalá^1^, Cimitarra^1,2^, El Playon^28^, Landázuri^1^; Sucre: San Onofre^13^, Toluviejo^28^; Tolima: Alvarado^1^, Armero^17,2,3,5^, Chaparral^5^, Cunday^1,2^, El Espinal^3^, Guamo^5^, Honda^18,3,28^, Ibagué^10,17,5,28^, Icononzo^1^, Melgar^2,5,28^, Nilo^2^, San Sebastián de Mariquita^1,2,5^; Valle del Cauca: Andalucía^12^, Buenaventura^11,17^, Buga^2^, Bugalagrande^5^, Cali^1,11,12,17,5,28^, Candelaria^12^, Cartago^12^, Dagua^1,11,17,28,20^, El Cerrito^11,12^, El Dovio^1^, El Queremal^17^, Jamundí^11^, La Cristalina^17^, Palmira^11,12,17,3,28,20^, Pradera^12^, Roldanillo^11,17^, , Trujillo^17^, Tuluá^11,17^, Yotoco^12,17^, Yumbo^28^, Zarzal^11^; Vaupés: Mitú^17^; Vichada: Cumaribo^18^.

***Xylocopa* *(Neoxylocopa)* *lachnea* Moure, 1951**

**Country records**: Amazonas: La Pedrera^1^; Antioquia: Guarne^28^, Itagüí^28^, Medellín^28^, Retiro^28^, Rionegro^3^, Yolombó^28^; Boyacá: Chitaraque^1^, San Miguel de Sema^1^, Tibaná^1^, Villa de Leyva^28^; Caldas: Pácora^15^, Riosucio^28^; Caquetá: Florencia^28^; Cauca: Caldono^12^, Inzá^1^, Silvia^12^; Cundinamarca: Anolaima^1^, Arbeláez^1^, Cabrera^1^, Fusagasugá^1^, San Antonio del Tequendama^7^, Silvania^1^, Suesca^1^; Magdalena: Ciénaga^1^; Meta: Carimagua^17^, Puerto Gaitán^11^; Nariño: Santa Bárbara^11^; Norte de Santander: Pamplonita^28^; Quindío: Armenia^15^, Calarcá^15^, Filandia^15^, La Tebaida^15^, Montenegro^15^, Quimbaya^15^, Salento^15^; Risaralda: Pereira^15^, Santa Rosa de Cabal^15^, Santuario^15^; Tolima: Ibagué^1^; Valle del Cauca: Cali^11,17,28,20^, Dagua^11,17^, El Cerrito^1,15^, Guacarí^11,17^, La Cumbre^11,17^, Palmira^1,11,12,3^, Cali^11^, Trujillo^11^, Tuluá^11^, Yotoco^12^.

***Xylocopa* *(Neoxylocopa)* *mastrucata* Pérez, 1901**

**Country records**: Antioquia: Beláo^13^, Jardín^13^, Medellín^13^; Boyacá: Buenavista^1^, Duitama^1^, Maripí^1^, Sotaquira^1^, Tunja^18^, Villa de Leyva^1,18^; Caquetá: San José del Fragua^1^; Cauca: Inzá^1^, Páez^1^, Popayán^1^; Cundinamarca: Anolaima^1^, Arbeláez^1^, Cabrera^1^, Fosca^1^, Nimaima^1^, Pacho^1^, Paime^1^, Quetame^1^, Quipile^1^, San Antonio del Tequendama^1,2,7^, San Juan de Río Seco^1^, Sasaima^1^, Subachoque^2^, Suesca^1^, Supatá^2^, Tena^1^, Villeta^1^; Huila: Acevedo^18^, Algeciras^1^, Hobo^1^, La Argentina^1^, Miraflores^18^, San Agustín^1^; Risaralda: Pereira^1,2^; Santander: Encino^1^, Floridablanca^1^; Tolima: Ataco^1^; Valle del Cauca: Él Dovio^1^.

***Xylocopa* *(Neoxylocopa)* *mordax* Smith, 1874**

**Country records**: Cesar: Chimichagua^28^; Magdalena: Santa Marta^1^; Tolima: El Espinal^3^.

***Xylocopa (Neoxylocopa)* *nasica* Pérez, 1901**

**Country records**: Cundinamarca: Medina^1^; Meta: Puerto Rico^1^.

***Xylocopa* *(Neoxylocopa)* *orthogonaspis* Moure, 2003**

**Country records**: Bolívar: El Carmen de Bolívar^18^.

***Xylocopa* *(Neoxylocopa)* *similis* Smith, 1874**

**Country records**: Amazonas: La Pedrera^1^, Leticia^1,12,7,28^, Mirití-Paraná^1^; Caquetá: Florencia^13^, Mapiripán^18^; Guaviare: San José del Guaviare^1^; Putumayo: Puerto Asís^1^, Puerto Guzmán^1^, Puerto Leguízamo^18^; Valle del Cauca: Palmira^12^.

***Xylocopa* *(Neoxylocopa)* *transitoria* Pérez, 1901**

**Country records**: Amazonas: El Encanto^1^; Antioquia: Amalfi^28^, Barbosa^13^, Bello^13^, Caucasia^13^, Girardota^13^, Guarne^13^, Itagüí^28^, Marinilla^13^, Medellín^1,13,3,28^, Puerto Berrío^13^, Yolombó^28^, Zaragoza^28^; Bolívar: Cartagena^1,13,18,2,19,28^, Córdoba^18^, El Carmen de Bolívar^18^, San Martín de Loba^1^, , Zambrano^1,18^; Boyacá: Maripí^1^, Pauna^1^, Puerto Boyacá^1^; Caquetá: Morelia^1^; Casanare: Paz de Ariporo^18^, San Luís de Palenque^1^, Trinidad^1^, Villanueva^1^, Yopal^1^; Cesar: Valledupar^3,28^; Cundinamarca: Arbeláez^1^, Guaduas^1^, La Vega^1^, Nilo^1^, Paime^1^, Puerto Salgar^1^, Silvania^1^, Villeta^1^; Huila: Acevedo^1^, Agrado^1^, Colombia^1^, La Argentina^1^, Neiva^1^, Villavieja^1,7^; La Guajira: Manaure^1^, Riohacha^1^; Magdalena: Ciénaga^6^, El Retén^6^, San Zenón^18^, Santa Marta^1,18,6,28^, Zona Bananera^3^; Meta: Puerto Gaitán^11^, Puerto López^1,2^, Villavicencio^1,3^; Norte de Santander: Cúcuta^1^, Los Patios^1^, San Cayetano^1^; Quindío: Quimbaya^13^; Santander: San José de Miranda^7^, Villavieja^7^; Sucre: San Onofre^13^; Tolima: Ataco^1^, El Espinal^3^, Natagaima^7^,Honda^3^, San Sebastián de Mariquita^1,2^; Valle del Cauca: Cali^11^.

**Subgenus *Schonnherria* Lepeletier, 1841**

***Xylocopa* *(Schonnherria)* *auriventris* Villamizar, Fernández & Vivallo, 2020 §***

**Country records**: Norte de Santander: Ábrego†^(Villamizar, Fernández & Vivallo, 2020)^.

***Xylocopa* *(Schonnherria)* *dimidiata* Latreille, 1809**

**Country records:** Amazonas: La Pedrera^(Villamizar, Fernández & Vivallo, 2020)^.

***Xylocopa* *(Schonnherria)* *ecuadorica* Cockerell, 1909***

**Country records**: Amazonas: La Pedrera ^(Villamizar, Fernández & Vivallo, 2020)^; Antioquia: Envigado ^9,(Villamizar, Fernández & Vivallo, 2020)^, Itagüí ^13,(Villamizar, Fernández & Vivallo, 2020)^, Medellín ^(Villamizar, Fernández & Vivallo, 2020)^, Sabaneta^(Villamizar, Fernández & Vivallo, 2020)^; Bolívar: Zambrano ^(Villamizar, Fernández & Vivallo, 2020)^; Caldas: Viterbo^1^; Casanare: Orocué ^(Villamizar, Fernández & Vivallo, 2020)^; Cundinamarca: Chaguaní^12^; Valle del Cauca: Dagua^12^, El Cerrito^12^, La Unión^12^, Palmira^12^.

***Xylocopa* *(Schonnherria)* *lateralis* Say, 1837**

**Country records**: Amazonas: La Pedrera^(Villamizar, Fernández & Vivallo, 2020)^; Antioquia: Itagüí^13^, Medellín^(Villamizar, Fernández & Vivallo, 2020)^, Turbo^(Villamizar, Fernández & Vivallo, 2020)^; Caldas: Pensilvania^28^;Nariño: Tumaco^(Villamizar, Fernández & Vivallo, 2020)^ Norte de Santander: Pamplonita^28^; Valle del Cauca: Buenaventura^18^, Cali^12^, Palmira^1^,

***Xylocopa* *(Schonnherria)* *lucida* Smith, 1874**

**Country records**: Guainía: Inírida^18,2^; Huila: La Argentina^1^; Meta: San Juan de Arama^1^; Valle del Cauca: Él Dovio^1^.

***Xylocopa* *(Schonnherria)* *macrops* Lepeletier, 1841**

**Country records**: Valle del Cauca: Él Dovio^1.^

***Xylocopa* *(Schonnherria)* *maidli* Maa, 1940 §**

Country records: No specific location records ^(Maa, 1940)^.

***Xylocopa* *(Schonnherria)* *metallica* Smith, 1874**

**Country records**: Meta: San Juan de Arama^1^.

***Xylocopa* *(Schonnherria)* *muscaria* (Fabricius, 1775)**

**Country records**: Antioquia: Amalfi^28^, Copacabana^8^, Medellín^(Villamizar, Fernández & Vivallo, 2020)^, Necoclí^(Villamizar, Fernández & Vivallo, 2020)^, Yolombó^28^, Zaragoza^(Villamizar, Fernández & Vivallo, 2020)^; Bolívar: Cartagena^19^; Casanare: Villanueva^1^; Caquetá: San Vicente del Caguán^1^,Cundinamarca: Medina^1^, Paime^1^,Topaipí^1^; Guainía: Inírida^1^;Guaviare: El Retorno^(Villamizar, Fernández & Vivallo, 2020)^, Huila: Villavieja^7,(Villamizar, Fernández & Vivallo, 2020)^; Magdalena: El Retén^6^, Santa Marta^1^; Meta: La Macarena^1^, Puerto Rico^1^, Restrepo^1^, Vistahermosa^18^; San Andrés y Providencia: Providencia^1,13,17^, San Andrés^1,17^; Sucre: San Onofre^13^; Tolima: Armero^2^, Nilo^2^; Valle del Cauca: El Dovio ^(Villamizar, Fernández & Vivallo, 2020)^ Vichada: Cumaribo^18^.

***Xylocopa* *(Schonnherria)* *ornata* Smith, 1874**

**Country records**: Amazonas: Puerto Santander^1^; Antioquia: Zaragoza ^(Villamizar, Fernández & Vivallo, 2020)^; Caquetá: Solano ^(Villamizar, Fernández & Vivallo, 2020)^; Putumayo: Puerto López ^(Villamizar, Fernández & Vivallo, 2020)^; Tolima: Armero ^(Villamizar, Fernández & Vivallo, 2020)^; Santander: Cimitarra^1^.

***Xylocopa* *(Schonnherria)* *romeroi* Villamizar, Fernandez & Vivallo, 2020 §***

**Country records**: Valle del Cauca: Calima†^1,(Villamizar, Fernández & Vivallo, 2020).^

***Xylocopa* *(Schonnherria) simillima* Smith, 1854**

**Country records:** Amazonas: Puerto Santander^1^; Meta: San Juan de Arama^1^, San Vicente del Caguán^1^; Valle del Cauca: Buenaventura^17^.

***Xylocopa* *(Schonnherria)* *viridis* Smith, 1854**

**Country records**: Antioquia: Ituango^28^, Maceo^13^, Mutatá ^(Villamizar, Fernández & Vivallo, 2020)^, Yolombó ^(Villamizar, Fernández & Vivallo, 2020)^, Zaragoza ^(Villamizar, Fernández & Vivallo, 2020)^; Boyacá: Puerto Boyacá^1^; Caquetá: Solano^18^; Casanare: Villanueva^1^; Cundinamarca: La Mesa^1^, Puerto Salgar^1^; Guaviare: Calamar ^(Villamizar, Fernández & Vivallo, 2020)^, San José del Guaviare^18, (Villamizar, Fernández & Vivallo, 2020)^; Huila: Palestina^1^; Meta: Cumaral^1^, Puerto López^2^; Risaralda: Pueblo Rico^20^; Tolima: Armero ^(Villamizar, Fernández & Vivallo, 2020)^, Valle del Cauca: Dagua^17^,Vijes^28^, Vichada: Cumaribo ^(Villamizar, Fernández & Vivallo, 2020)^.

**Subgenus *Stenoxylocopa* Hurd & Moure, 1960**

***Xylocopa* *(Stenoxylocopa)* *lehmanni* Friese, 1903 §**

**Country records**: Cauca: Popayán† ^(Friese, 1903)^.

**Subgenus *Xylocopina* Hurd & Moure, 1963**

***Xylocopa* *(Xylocopina) asedothae* Zama & Silveira, 2024 §***

**Country records:** Caquetá: Curillo†^(Zama & Silveira, 2024)^.

**Colletidae Lepeletier, 1841**

**Colletinae Lepeletier, 1841**

**Genus *Colletes* Latreille, 1802**

***Colletes costaricensis* Friese, 1916**

**Country records:** Cauca: Popayán^(Friese, 1916)^

***Colletes* *nitidicollis* Friese, 1900 ***

**Country records**: Cundinamarca: Bogotá D.C^7^, Cota^1^, Tenjo^7^.

***Colletes* *petropolitanus* Dalla Torre, 1896**

**Country records**: Antioquia: Amalfi^28^, Yolombó^28^; Cauca: Morales^1^; Cesar: Agustín Codazzi^19^; Cundinamarca: Apulo^1^, Tibacuy^1^; Magdalena: Santa Marta^1^; Santander: Zapatoca^28^; Valle del Cauca: El Dovio^1^, Palmira^12^.

***Colletes*** ***rugicollis* Friese, 1900**

**Country records**: Antioquia: Amalfi^14^, Andes^14^, Cocorná^13^, Itagüí^14^, Maceo^13^, Medellín^14^; Boyacá: Santa María^1^; Cundinamarca: Anolaima^1^; Meta: Villavicencio^1^; Santander: El Carmen de Chucurí^13^.

***Colletes*** ***rutilans* Vachal, 1909***

**Country records**: Antioquia: Acandí^14^, Amalfi^14^, Rionegro^14^, Sabaneta^13^, Yolombó^14^; Cauca: Puracé^28^; Cesar: Agustín Codazzi^19^; Cundinamarca: Anolaima^1^, Pandi^1^, Quetame^1^; Valle del Cauca: Cali^17,28^, El Cairo^1^, El Dovio^1^, Palmira^1^.

***Colletes*** ***spilopterus* Cockerell, 1917***

**Country records**: Cundinamarca: Anolaima^1^; Huila: Colombia^1^; Nariño: San Sebastián^1^; Valle del Cauca: Él Dovio^1^, Palmira^12^.

***Colletes*** ***ursinus* Smith, 1853 §**

**Country records**: No specific location records ^(Smith, 1853)^.

**Diphaglossinae Vachal, 1909**

**Caupolicanini Michener, 1944**

**Genus *Ptiloglossa* Smith, 1853**

***Ptiloglossa buchwaldi Friese, 1908***

**Country records:** Cauca: Popayán^28^

***Ptiloglossa*** ***costaricana* Moure, 1945***

**Country records**: Antioquia: El Retiro^14^.

***Ptiloglossa*** ***cyaniventris*** **Friese, 1925**

**Country records**: Cauca: Popayán† ^(Friese, 1925a)^.

***Ptiloglossa*** ***eximia* (Smith, 1861)***

**Country records**: Antioquia: Amagá^14^, Guarne^14^, Medellín^14^, Porce^14^, San Luís^14^; Caldas: Chinchiná^14^; La Guajira: Maicao^24^; Meta: Puerto Gaitán^24^; Valle del Cauca: Buenaventura^24^, Calima^24^.

***Ptiloglossa* *fassli* Friese, 1925 §**

**Country records**: Cundinamarca: San Antonio del Tequendama† ^(Friese, 1925a)^.

***Ptiloglossa* cf. *lanosa* Moure, 1945**

**Country records:** Magdalena: Ciénaga ^(Velez-Ruiz, 2015)^**.**

**Notes**: This record relies exclusively on the report included in the unpublished doctoral thesis of Vélez-Ruiz (2015). However, the species was not found by any of the authors in the collections examined. Since its known distribution is restricted to the state of Minas Gerais, Brazil (Moure, Urban & Melo, 2022), its occurrence in the region should be corroborated by the examination of properly identified specimens.

***Ptiloglossa*** ***lucernarum* Cockerell, 1923***

**Country records**: Antioquia: Caldas^14^, Envigado^14^, Itagüí^14^, Medellín^14^, Porce^14^; Caldas: Chinchiná^14^; Cauca: Popayán^28^; Cundinamarca: Junín^1^, Pandi^1^, San Antonio del Tequendama^7,20^; Valle del Cauca: Dagua^20^.

***Ptiloglossa*** ***mayarum* Cockerell, 1912***

**Country records**: Antioquia: Medellín^14^, Porce^14^, San Francisco^14^

***Ptiloglossa*** ***mexicana* (Cresson, 1878)**

**Country records**: Magdalena: Santa Marta^22^.

***Ptiloglossa*** ***pretiosa* (Friese, 1898)***

**Country records:** Caldas: Chinchiná^14^.

***Ptiloglossa*** ***steinheili*** **Friese, 1899 §**

**Country records**: No specific location records.

**Note:** According to Friese (Friese, 1899c), the holotype label reads: “von Guayabal, Columbia (Steinheil).” However, since Guayabal does not correspond to a specific locality in Colombia, as several villages and some municipalities bear this name, it is impossible to determine the exact site where the specimen was collected.

***Ptiloglossa*** ***tenuimarginata* (Smith, 1879)***

**Country records**: Antioquia: Amagá^14^, Medellín^14^.

***Ptiloglossa*** ***trichrootricha* Moure, 1989 §**

**Country records**: Cauca: Miranda† ^(Moure, 1987)^.

***Ptiloglossa*** ***xanthorhina* Moure, 1945***

**Country records:** Casanare: Villanueva^1^.

**Genus *Zikanapis* Moure, 1945**

***Zikanapis*** ***modesta* Moure, 1964 §**

**Country records**: Magdalena: Santa Marta†^20^.

**Diphaglossini Vachal, 1909**

**Genus *Cadegualina* Michener, 1986**

***Cadegualina* *andina* (Friese, 1925)**

**Country records:** Antioquia: Liborina^13^; Boyacá: Villa de Leyva^1^.; Cundinamarca: Une^14^; Magdalena: Aracataca^1^.

***Cadegualina* *sericata* (Friese, 1925)**

**Country records**: Boyacá: Villa de Leyva (Gonzalez & Michener, 2004a); Cundinamarca: Fómeque^18^, Guachetá^1^, La Calera^1^.

**Dissoglottini Moure, 1945**

**Genus *Mydrosoma* Smith, 187**

***Mydrosoma* cf. *opalinum***

Country records: Meta: San Martín^2^.

**Hylaeinae Viereck, 1916**

**Genus *Hylaeus* Fabricius, 1793**

**Country records**: Amazonas: Leticia^18^; Antioquia: Bello^3^, Guarne^20^, San Francisco^18^, Sonsón^18^, Yalí^3^; Bolívar: San Jacinto^18^; Boyacá: Ventaquemada^18^; Caldas: Aranzazu^15^, Pácora^15^; Cundinamarca: Agua de Dios^7^; La Guajira: Maicao^20^; Magdalena: Santa Marta^18,6^; Meta: La Uribe^18^; Quindío: Calarcá^15^, Quimbaya^6^; Santander: Socorro^7^; Tolima: Honda^18^, Rovira^15^; Valle del Cauca: Calima^20^, Dagua^20^, Cali^20^, Palmira^15^.

**Neopasiphaeinae Cockerell, 1930**

**Eulonchopriini Moure, 1945**

**Genus *Perditomorpha* Ashmead, 1899**

***Perditomorpha*** ***rosellae* (Gonzalez, 2011) ***

**Country records**: Bolívar: San Jacinto^18^, San Juán Nepomuceno^1^; La Guajira: Maicao^28^; Magdalena: Aracataca^6^, Santa Marta†^1,5,28^.

**Tribe Lonchopriini Moure, 1945**

**Genus *Biglossidia* Moure, 1948**

***Biglossidia*** ***comforti*** **(Gonzalez & Engel, 2014) ***

**Country records:** Antioquia: Alejandría^13^, Envigado†^14,28^, La Unión^14^, Medellín^20^, Retiro^28^, Rionegro^28^; Caldas: Pensilvania^28^; Cauca: Él Tambo^24^; Cundinamarca: San Antonio del Tequendama^7^.

***Biglossidia*** ***danunciae*** **(Gonzalez & Engel, 2015) §***

**Country records**: Norte de Santander: Pamplona† ^(Gonzalez & Engel, 2015)^.

**Genus *Lonchorhyncha* Michener, 1989**

***Lonchorhyncha* *ecuadoria* (Friese, 1925) ***

**Country records**: Cauca: El Tambo^20^; Valle del Cauca: Tuluá^24^.

**Xeromelissinae Cockerell, 1926**

**Genus *Chilicola* Spinola, 1851**

**Subgenus *Anoediscelis* Toro & Moldenke, 1979**

***Chilicola* *(Anoediscelis)* *ashmeadi* (Crawford, 1906)**

**Country records**: Valle del Cauca: Cali ^28^

***Chilicola* *(Anoediscelis)* *colombiana* Michener, 2002**

**Country records:** Antioquia: Guarne ^28^, Rionegro ^28^; Boyacá: Nuevo Colón^1^; Cundinamarca: Bogotá, D.C. ^1^; Valle del Cauca: Cali† ^20^

***Chilicola* *(Anoediscelis)* *mistica* Michener, 2002***

**Country records**: Cundinamarca: Bogotá, D.C.^7^.

***Chilicola*** ***(Anoediscelis)*** ***paramo* Gonzalez & Michener, 2004§**

**Country records**: Boyacá: Arcabuco†^28^.

***Chilicola* *(Anoediscelis)* *paramoides* Gonzalez, 2009***

**Country records**: Boyacá: Arcabuco†^5^, Chiquiza^5^, Nuevo Colón^1^, Ventaquemada^28^; Cundinamarca: Fomeque(Gonzalez & Michener, 2004b).

***Chilicola* *(Anoediscelis)* *wygodzinskyi* Michener, 2002§**

**Country records:** Valle del Cauca: Cali†^20^

**Subgenus *Hylaeosoma* Ashmead, 1898**

***Chilicola* *(Hylaeosoma)* *aequatoriensis* Benoist, 1942**

**Country records:** Antioquia: Rionegro^14,28^; Boyacá: Nuevo Colón^1^, Tunja^1^; Cundinamarca: Bogotá, D.C.^7^, Junín^1^, Soacha^7^, Zipaquirá^1^; Valle del Cauca: Cali^20^.

***Chilicola* *(Hylaeosoma)* *belli* Michener, 2002**

**Country records**: Antioquia: Rionegro^14^; Valle del Cauca: Cali† ^20^

***Chilicola (Hylaeosoma)* *bochica* Gonzalez, 2009***

**Country records**: Boyacá: Arcabuco^5^, Ventaquemada†^28^

***Chilicola* *(Hylaeosoma)* *canei* Michener, 2002**

**Country records**: Antioquia: Belmira^13^, Medellín ^28^, Sonsón† ^28^

***Chilicola* *(Hylaeosoma)* *muruimuinane* Smith-Pardo & Gonzalez, 2007 §**

**Country records**: Putumayo: Mocoa†^20^

***Chilicola* (*Hylaeosoma)* *smithpardoi* Michener, 2002 §**

**Country records**: Antioquia: Amalfi† ^20^

***Chilicola* *(Hylaeosoma)* *stenocephala* Brooks & Michener, 1999 §**

**Country records:** Amazonas: La Chorrera† ^28^

***Chilicola* *(Hylaeosoma)* *umbonata* Michener, 2002 §**

**Country records**: Valle del Cauca: Cali† ^20^

**Subgenus *Oroediscelis* Michener, 2002**

***Chilicola* *(Oroediscelis)* *brooksi* Michener, 2002**

**Country records**: Antioquia: Rionegro^14^; Cundinamarca: Bogotá, D.C.^7^, San Antonio del Tequendama^7^

***Chilicola* *(Oroediscelis)* *brzoskai* Michener, 2002**

**Country records**: Valle del Cauca: Cali†^20^

***Chilicola* *(Oroediscelis)* *deborahae* Gonzalez, 2009 §***

**Country records**: Boyacá: Arcabuco ^(Gonzalez & Giraldo, 2009)^; Cundinamarca: Fómeque† ^(Gonzalez & Giraldo, 2009)^.

***Chilicola* *(Oroediscelis)* *gibbosa* Michener, 2002**

**Country records**: Caldas: Pensilvania† ^28^

***Chilicola* *(Oroediscelis)* *transversaria* Michener, 2002**

**Country records**: Norte de Santander: Abrego ^(Michener, 2002)^.

**Halictidae Thomson, 1869**

**Halictinae Thomson, 1869**

**Augochlorini Beebe, 1925**

**Genus *Ariphanarthra* Moure, 1951**

***Ariphanarthra* *palpalis* Moure, 1951**

**Country records**: Caquetá: Curillo ^(Engel, 1996)^.

**Genus *Augochlora* Smith, 1853**

***Augochlora*** ***laneifrons* (Vachal, 1911) §\**

**Country records**: Cundinamarca† ^(Vachal, 1911)^.

**Note**: The type locality indicated on the syntype labels, according to Vachal (1911) and Moure, Urban & Melo (2022), is “Colombia, Cundinamarca, Bogotá.” However, it is likely that the locality corresponds to another place within the department of Cundinamarca.

**Subgenus *Augochlora* Smith, 1853**

***Augochlora* *(Augochlora) alexanderi* Engel, 2003 §**

**Country records**: Caquetá: El Paujil†^1,20^.

***Augochlora* *(Augochlora)* *bogotensis* (Vachal, 1911) §**

**Country records**: Cundinamarca† ^(Vachal, 1911)^.

**Note**: The type locality indicated on the syntype labels, according to Vachal (1911) and Moure, Urban & Melo (2022), is “Colombia, Cundinamarca, Bogotá.” However, it is likely that the locality corresponds to another place within the department of Cundinamarca.

***Augochlora (Augochlora) dryas* Gonçalves & Lepeco 2026**

**Country records**: Meta: Puerto Gaitan (Gonçalves & Lepeco, 2026)^.^

***Augochlora* *(Augochlora)* *engys* (Vachal, 1911) §**

**Country records**: Quindío: Salento† ^(Vachal, 1911; Moure, 1999)^.

***Augochlora (Augochlora) essequibensis* Cockerell, 1923.**

**Country records:** Antioquia: Santa Fé de Antioquia ^(Gonçalves & Lepeco, 2026)^.

***Augochlora* *(Augochlora)* *nigrocyanea* Cockerell, 1897**

**Country records**: Antioquia: Amalfi^28^, Yolombó^28^.

***Augochlora* *(Augochlora)* *quiriguensis* Cockerell 1913**

**Country records**: Meta: Puerto Colombia^1^.

***Augochlora* *(Augochlora)* *repandirostris* (Vachal, 1911)**

**Country records**: Antioquia: Amalfi^28^, Yolombó^28^.

***Augochlora* *(Augochlora)* *rohdei* (Vachal, 1911)**

**Country records**: Boyacá: Muzo† ^(Vachal, 1911)^.

***Augochlora* *(Augochlora)* *sidaefoliae* Cockerell, 1913**

**Country records**: Antioquia: Amalfi^28^, Yolombó^28^; Cundinamarca: Arbeláez^1^.

**Subgenus *Oxystoglossella* Eickwort, 1969**

***Augochlora* *(Oxystoglossella)* *antonita* Michener, 1954***

**Country records**: Valle del Cauca: Cali^(Lepeco & Gonçalves, 2023)^.

**Note**: This species was reported for the department of Meta by Lepeco & Gonçalves (2023) by based on a specimen with the following label information: “Carimagua – Meta \ Colombia \ V-1978 \ Cesar García.” However, Carimagua does not correspond to a municipality or locality in the department of Meta, and the dash “–” may refer to a collection range. Moreover, considering that the known distribution of the species is restricted to the western Andes (pers. comm. Anderson Lepeco), we consider that this species does not occur in the Orinoco Llanos region of the department of Meta.

***Augochlora* *(Oxystoglossella)* *brevipilosa* Lepeco & Gonçalves, 2023 §***

**Country records**: Valle del Cauca: Cali† ^(Lepeco & Gonçalves, 2023).^

***Augochlora* *(Oxystoglossella)* *clarki* Michener, 1954***

**Country records**: Antioquia: La Pintada^14^, Medellín^14^, Santa Fé de Antioquia^14^, Tarazá^14^; Bolívar: Cartagena^14^; Córdoba: Cerete^14^, Montería^14^.

***Augochlora* *(Oxystoglossella)* *cordiaefloris* Cockerell, 1907**

**Country records**: Antioquia: Amalfi^28^, Yolombó^28^.

***Augochlora (Oxystoglossella) morrae* Strand, 1910**

**Country records:** No specific location recorded.

**Note**: Based on the syntype labels, the type locality of this species is indicated as: “Bolivia; Peru, Lima, Callanga; Brazil, São Paulo; Paraguay; Colombia; Mexico, Veracruz, Orizaba; Costa Rica.” (Moure & Hurd, 1987; Moure, Urban & Melo, 2022)

***Augochlora* *(Oxystoglossella)* *nominata* Michener, 1954**

**Country records**: Antioquia: Alejandría^14^, Amalfi^28^, Bello^28^, Itagüí^14,28^, Medellín^14,28^, Rionegro^14^, Yolombó^28^

**Genus *Augochlorella* Sandhouse, 1937**

***Augochlorella* *comis* (Vachal, 1911)**

**Country records**: Antioquia: Amalfi^14,28^, Envigado^28^, Itagüí^28^, Medellín^28^, Yolombó^28^.

***Augochlorella* *neglectula* (Cockerell, 1897)**

**Country records**: Bolívar: Cartagena^1928^.

***Augochlorella* *tredecim* (Vachal, 1911)**

**Country records**: Cesar: Pueblo Bello^28^; Magdalena: Fundación^28^.

**Genus *Augochloropsis* Cockerell, 1897**

**Subgenus *Augochloropsis* Cockerell, 1897**

***Augochloropsis* *(Augochloropsis)* *anquisita* (Cockerell, 1913)**

**Country records**: Antioquia: La Estrella^23^; Valle del Cauca: Buenaventura^5,19,27^.

***Augochloropsis* (*Augochloropsis*) *auriventris* (Friese, 1921)**

**Country records**: Antioquia: Amalfi^14,28^, Yolombó^28^; Caldas: Aguadas^15^; Cundinamarca: Agua de Dios^7^; Quindío: Quimbaya^15^.

***Augochloropsis* *(Augochloropsis)* *callichroa* (Cockerell, 1900)**

**Country records**: Antioquia: Alejandría^13^, Amalfi^13,28^, Caldas^13^, Caucasia^13^, Itagüí^13^, Maceo^13^, Yolombó^13,28^; Meta: San Juan de Arama^20^; Santander: Cimitarra^13^; Sucre: San Onofre^13^.

***Augochloropsis* *(Augochloropsis)* *calypso* (Smith, 1879)***

**Country records**: Amazonas: Leticia^18^; Casanare: Villanueva^1^; Cundinamarca: Medina^1^, Nilo^5,7^, Tocaima^5^; Huila: Neiva^1^, Villavieja^7^; Meta: Cumaral^23^, Puerto Gaitán, Puerto López^1^, Puerto Rico^1^; Vichada: Cumaribo^18^.

***Augochloropsis* *(Augochloropsis)* *charapina* (Cockerell, 1913)***

**Country records**: Quindío: Filandia^13^.

***Augochloropsis* *(Augochloropsis)* *cyclis* (Vachal, 1903)***

**Country records**: Putumayo: Puerto Asís^1^

***Augochloropsis* *(Augochloropsis)* *huebneri* (Alfken, 1930)***

**Country records**: Amazonas: Leticia^27^.

***Augochloropsis* *(Augochloropsis)* *ignita* (Smith, 1861)***

**Country records**: Antioquia: Itagüí^28^.

***Augochloropsis* *(Augochloropsis)* *notophos* (Vachal, 1903)**

**Country records**: Meta: La Uribe^20.^

***Augochloropsis* *(Augochloropsis)* *semilaeta* (Cockerell, 1923)***

**Country records**: Amazonas: Cumaribo^5^; Vichada: Cumaribo^5^.

***Augochloropsis* *(Augochloropsis)* *toralis* (Vachal, 1904)**

**Country records**: Guaviare: El Retorno^18^, San José del Guaviare^18^; Meta: Vistahermosa^18^.

**Subgenus *Glyptochlora* Moure, 1958**

***Augochloropsis* *(Glyptochlora)* *ticuna* Celis & Melo, 2022 §***

**Country records**:  Amazonas: Leticia† ^(Celis & Melo, 2022)^.

**Subgenus *Paraugochloropsis* Schrottky, 1906**

***Augochloropsis* *(Paraugochloropsis)* *aurifluens* (Vachal, 1903)**

**Country records**: No specific location recorded.

**Note**: Based on the syntype labels, the type locality of this species is indicated as: “Venezuela; Colombia; Brazil, São Paulo” (Moure & Hurd, 1987; Moure, Urban & Melo, 2022)

***Augochloropsis* (*Paraugochloropsis)* *cupreola* (Cockerell, 1900)**

**Country records**: Valle del Cauca: Dagua ^(Vachal, 1903)^.

***Augochloropsis* *(Paraugochloropsis)* *fuscocyanea* (Michener, 1954)***

**Country records**: Antioquia: Amalfi^13,14^, Maceo^13^, Porce^14^; Caldas: Norcasia^14^, Victoria^14^.

***Augochloropsis* *(Paraugochloropsis)* *hebescens* (Smith, 1879)***

**Country records**: Cundinamarca: Agua de Dios^7^; Santander: Socorro^7^.

***Augochloropsis* *(Paraugochloropsis)* *metallica* Fabricius, 1793**

**Country records**: Antioquia: Guarne^14^, Medellín^14^, Turbo^14^; Meta: Villavicencio^1^.

***Augochloropsis* *(Paraugochloropsis)* *pallitarsis* Friese, 1916**

**Country records**: Magdalena: Santa Marta^1^.

***Augochloropsis* *(Paraugochloropsis)* *vesta* (Smith, 1853)**

**Country records**: Antioquia: Alejandría^13,14^, Amalfi^14,28^, Apartadó^4,28^, Caldas^14^, Itagüí^13^, Jericó^13^, Maceo^13^, Medellín^14^, Tarazá^14^, Titiribí^14^, Yolombó^28^; Cauca: Inzá^14^; Risaralda: Pereira^13^.

**Genus *Caenaugochlora* Michener, 1954**

**Subgenus *Caenaugochlora* Michener, 1954**

***Caenaugochlora* *(Caenaugochlora)* *gemmella* (Cockerell, 1912)**

**Country records**: Antioquia: Amalfi^28^, Yolombó^28^; Cundinamarca: Arbeláez^1^.

***Caenaugochlora* (*Caenaugochlora*) *gonzalezi* Engel 2014***

**Country records**: Magdalena: Santa Marta† ^(Engel, 2014)^; Quindío: Calarcá^15^.

***Caenaugochlora* (*Caenaugochlora*) *jeffreyi* Engel, 1997 §**

**Country records**: Valle del Cauca: Buenaventura† ^(Engel, 1997a)^.

**Subgenus *Ctenaugochlora* Eickwort, 1969**

***Caenaugochlora* (*Ctenaugochlora*) *perpectinata* (Michener, 1954)**

**Country records**: Antioquia: Yolombó^28.^; Cundinamarca: Arbeláez^1^.

**Genus *Chlerogas* Vachal, 1904**

***Chlerogas colombiensis* Brooks & Engel, 1999 §**

**Country records**: Antioquia: Retiro†^28^.

***Chlerogas* *cyaneus* Brooks & Engel, 1999**

**Country records**: Cundinamarca: Cabrera†^20^, Choachí^5^.

***Chlerogas* *nephos* Brooks & Engel, 1999**

**Country records**: Antioquia: Caldas^14,20^; Medellín†^20^.

***Chlerogas* *tatamaensis* Engel & Gonzalez, 2009§***

**Country records**: Risaralda: Pueblo Rico†^5^.

**Genus *Chlerogella* Michener, 1954**

***Chlerogella* *anchicaya* Engel, Gonzalez & Hinojosa-Díaz, 2014 §***

**Country records**: Valle del Cauca: Buenaventura† ^(Engel, González & Hinojosa-Díaz, 2014)^.

***Chlerogella*** ***cyranoi* Engel, 2010 §***

**Country records**: Antioquia: Amalf† ^(Engel, 2010)^.

***Chlerogella*** ***elongaticeps* Michener, 1954***

**Country records**: Antioquia: Amalfi ^28^.

***Chlerogella* *hypermeces* Engel, 2010***

**Country records**: Putumayo: Villagarzón† ^(Engel, 2010)^.

***Chlerogella*** ***materdonnae* Engel, 2010***

**Country records**: Santander: Oiba^1^, Suaita^1^.

***Chlerogella* *picketti* Engel, 2010 §***

**Country records**: Nariño: Barbacoas† ^(Engel, 2010)^.

***Chlerogella*** ***terpsichore*** Engel, 2010 §*

**Country records**: Nariño: Barbacoas†^20^.

***Chlerogella* *tychoi* Engel, 2010 §***

**Country records**: Valle del Cauca: Buenaventura†^20^.

***Chlerogelloides* Engel**, Brooks & Yanega, 1997

***Chlerogelloides*** ***femoralis*** **Engel, Brooks & Yanega, 1997**

**Country records**: Putumayo: Mocoa ^(Engel et al., 1997)^, Villagarzón^(Engel et al., 1997)^; Vichada: Cumaribo^18^

**Genus *Corynurella* Eickwort, 1969**

***Corynurella* *singularis* Gonçalves, 2010***

**Country records**: Amazonas: Puerto Santander ^(Gonçalves, 2010)^.

**Genus *Ischnomelissa* Engel, 1997**

***Ischnomelissa* *cyanea* Brooks & Engel, 1998**

**Country records**: Nariño: Ricaurte†^5^.

***Ischnomelissa* *zonata* Engel, 1997**

**Country records**: Antioquia: Anorí^13^, Envigado^14^; Huila: Acevedo^(Engel, 1997b)^; Risaralda: Pereira^13^; Tolima: Rovira^15^; Valle del Cauca: Cali†^20^.

**Genus *Megalopta* Smith, 1853**

**Subgenus *Megalopta* Smith, 1853**

***Megalopta* *(Megalopta)* *amoena* (Spinola, 1853)**

**Country records**: Antioquia: Amalfi^28^, Caucasia^13^, Porce^14^; Santander: Cimitarra^13^; Sucre: San Onofre^13^.

***Megalopta* *(Megalopta)* *genalis* Meade-Waldo, 1916**

**Country records:** Antioquia: Girardota^13^, Porce^14^, Puerto Berrío^13^, San Luís^13^, Yolombó^28^; Casanare: Aguazul^7^, San Luís de Palenque^7^; Chocó: Litoral del San Juan^1^, Nuquí^14^; Magdalena: Santa Marta^14^; Santander: Cimitarra^13^.

***Megalopta* *(Megalopta)* *mura* Santos & Melo, 2014***

**Country records**: Valle del Cauca: Buenaventura^14^.

***Megalopta* *(Megalopta)* *nitidicollis* Friese, 1926**

**Country records**: No specific location records.

**Note:** The type locality is Bolivia, Cochabamba, Tarata, according to the lectotype designation by Santos & Melo (Santos & Melo, 2015). In addition to the lectotype and the paralectotype from this locality, there are three other paralectotypes deposited in the National Museum in Szczecin, bearing the following label information: “3♀ von Columbie im Mus. Stettin, Pehlke leg” (Friese, 1926)

***Megalopta* *(Megalopta)* *sodalis* (Vachal, 1904)***

**Country records**: Antioquia: Puerto Berrío^13^.

**Genus *Megaloptidia* Cockerell, 1900**

*M****egaloptidia* *nocturna* (Friese, 1926)**

**Country records**: Amazonas: Leticia^18^; Caquetá: Belén de los Andaquíes^17^; Cauca: Guapi^18^; Guaviare: San José del Guaviare^18^; Meta: La Macarena^1^, San Martín^2^; Valle del Cauca: Buenaventura^20^; Vichada: Cumaribo^18^.

**Genus *Megaloptilla* Moure & Hurd, 1987**

***Megaloptilla*** ***callopis* (Vachal, 1911)***

**Country records:** Putumayo: Mocoa ^(Engel & Brooks, 1999)^, Villagarzón ^(Engel & Brooks, 1999)^

**Genus *Neocorynura* Schrottky, 1910**

***Neocorynura* cf. *banarae***

**Country records**: Valle del Cauca: Cali^20^

**Note**: This specimen was identified by Smith-Pardo as *Neocorynura banarae* (Ducke, 1906). However, this species has only been reported from the state of Pará, Brazil (Gonçalves, 2019; Moure, Urban & Melo, 2022).

***Neocorynura* *caucana* Smith-Pardo & Gonzalez, 2009 §***

**Country records**: Valle del Cauca: Cali†^20^.

***Neocorynura* *colombiana* Eickwort, 1979**

**Country records**: Magdalena: Santa Marta†^20^; Valle del Cauca: El Cerrito^20^.

***Neocorynura*** ***cosmetor* (Vachal, 1911)**

**Country records**: Antioquia: La Pintada^4,28^; Boyacá: Tunja^28^; Cundinamarca: Bogotá D.C† ^(Vachal, 1911)^; Quindío: Calarcá^7^; Tolima: Rovira^15^.

***Neocorynura*** ***iguaquensis* Smith-Pardo & Gonzalez, 2006**

**Country records**: Boyacá: Arcabuco†^18^, Chiquiza^1,5^, Ventaquemada^18,28^, Villa de Leyva^1,7,28^; Cundinamarca: Bogotá, D.C.^7^, Choachí^1^, Granada^1^, Junín^18^, La Calera^1,7^, San Antonio del Tequendama^7,20^, San Francisco^1^, Subachoque^7^, Villavieja^7^.

***Neocorynura*** ***joannisi* (Vachal, 1904)**

**Country records**: Nariño: San Lorenzo ^(Engel, 1996)^.

***Neocorynura*** ***muiscae* Smith-Pardo & Gonzalez, 2006**

**Country records**: Boyacá: Arcabuco^18^, Chíquiza^5^, San Miguel de Sema^19^, Ventaquemada^18,28^, Villa de Leyva†^1,28^; Cundinamarca: Guasca^7^, Sopó^7^, Subachoque^7^, Tena^19^, Tocancipá^7^.

***Neocorynura*** ***pubescens* (Friese, 1917)**

**Country records**: Antioquia: Alejandría^14^, Carmen de Viboral^14^, Medellín^14^, Rionegro^14^.

***Neocorynura* *seeleyi* (Engel & Klein, 1997)**

**Country records**: Boyacá: Arcabuco^14,18^, Chíquiza^1,18,28^, Nuevo Colón^14^, Saboya^18^, Ventaquemada^18,28^; Cundinamarca: Bogotá, D.C.^1^, Guasca^1^, La Calera^1^, Mosquera^1,5,28^; Santander: Santa Bárbara^18^

**Genus *Pereirapis* Moure, 1943**

***Pereirapis*** ***semiaurata* (Spinola, 1853)**

**Country records**: Amazonas: Leticia^2^; Antioquia: Amalfi^28,20^, Bello^3^, Yolombó^28^; Magdalena: Santa Marta^18,6^; Meta: San Juan de Arama^18^; Quindío: Filandia^2^, Génova^18^, Quimbaya^18,6^; Risaralda: Mistrató^20^, Pueblo Rico^20^; Tolima: Coelloo^2^, Nilo^2^; Valle del Cauca: Buenaventura^20^, Dagua^20^, Palmira^3^, Cali^28,20^; Vichada: Cumaribo^18^.

**Genus *Pseudaugochlora* Michener, 1954**

***Pseudaugochlora* *graminea* (Fabricius, 1804)**

**Country records**: Antioquia: Bello^28^, Envigado^28^, Itagüí^28^, Medellín^28^; Caldas: Riosucio^28^; Cauca^1^; Cesar: Valledupar^28^; Cundinamarca: ^1^, Fusagasugá^1^, Medina^1^, Quetame^1^; Huila: Rivera^1^, Villavieja^7^; Magdalena: Santa Marta^6,28^; Meta: La Macarena^20^; Santander: Landázuri^1^; Tolima: Ibagué^28^ Rovira^15^; Valle del Cauca: Cali^28^.

**Genus *Rhinocorynura* Schrottky, 1909**

***Rhinocorynura* *briseis* (Smith, 1879)**

**Country records**: Amazonas: Puerto Nariño^20^.

**Genus *Stilbochlora* Engel, Brooks & Yanega, 1997**

***Stilbochlora*** ***eickworti* (Engel, Brooks & Yanega, 1997)**

**Country records**: Amazonas: La Pedrera^1^, Leticia^1^, Puerto Santander^1^; Boyacá: Santa María^2^; Caquetá: Solano^1^; Casanare: Tauramena^2^, Villanueva^1^; Guaviare: San José del Guaviare^18^; Meta: Puerto López^2^, San Martín^1^; Vaupés: Taraira^18^.

**Genus *Temnosoma* Smith, 1853**

**Country records**: Caquetá: Solano^18^; Magdalena: Santa Marta^6,18^; Meta: San Juan de Arama^18^; Tolima: Cunday^2^.

**Genus *Xenochlora* Engel, Brooks & Yanega, 1997**

***Xenochlora*** ***nigrofemorata* (Smith, 1879)***

**Country records**: Amazonas: Puerto Santander^1^.

**Caenohalictini Michener, 1954**

**Genus *Agapostemon* Guérin-Méneville, 1844**

**Subgenus *Notagapostemon* Janjic & Packer, 2003**

***Agapostemon* *(Notagapostemon)* *ascius* Roberts, 1972**

**Country records**: Caldas: Pensilvania^28^; Tolima: Cajamarca† ^(Roberts & Roberts, 1972)^.

***Agapostemon* *(Notagapostemon****)* ***intermedius* Roberts, 1972**

**Country records**: Córdoba: Tierralta^1^; Valle del Cauca: Vijes^28^.

***Agapostemon* *(Notagapostemon)* *leunculus* Vachal, 1903**

**Country records**: Antioquia: Alejandría^14^, El Carmen de Viboral^14^, El Retiro^14^, Guarne^13^, La Ceja^14^, Medellín^14^, Rionegro^14^, San Vicente^14^; Cauca: Popayán ^(Roberts & Roberts, 1972)^; Nariño: Buesaco^21^, La Unión^21^, Leiva^21^; Tolima: Mariquita^14^ Valle del Cauca: Jamundí^12^.

***Agapostemon* *(Notagapostemon)* *nasutus* Smith, 1853**

**Country records**: Antioquia: Bello^3^, Medellín^14,3,28^, Puerto Triunfo^14^, San Jerónimo^14^, Santa Fe de Antioquia^14^, Sopetrán^14^, Tarazá^14^; Atlántico: Barranquilla^1^, Puerto Colombia^1^; Bolívar: Cartagena^14,28^; Boyacá: Puerto Boyacá^1^; Cesar: Valledupar^1^; Huila: Gigante^1^, Tello^1^; La Guajira: Uribia^19,28^; Magdalena: Santa Marta^6,28^, Zona Bananera^6^; Nariño: Tumaco^14,3^; Quindío: La Tebaida^28^; Sucre: San Onofre^13^; Tolima: Ambalema^1^, Armero^14^, El Espinal^3^; Valle del Cauca: Buga^12^, Calima^12^ , Candelaria^12^, Dagua^17,20^, El Cerrito^1,12^,Jamundí^12,17^, Palmira^12,3,28^, Pradera^15^, Cali^12,17,28^, Tuluá^12^, Yumbo^17^.

***Agapostemon* *(Notagapostemon)* *semimelleus* Cockerell, 1900**

**Country records**: Antioquia: Amalfi^28^, Ituango^28^, Yolombó^28^; Boyacá: Muzo^28^; Cauca: Corinto^28^, Meta: Restrepo^28^; Risaralda: Mistrató^20^; Valle del Cauca: Dagua^20^, Jamundí^20^, La Cumbre^12^, Cali^28^.

**Genus *Agapostemonoides* Roberts & Brooks, 1987**

***Agapostemonoides* *hurdi* Roberts & Brooks, 1987**

**Country records**: Antioquia: Yolombó^28^.

**Genus *Caenohalictus* Cameron, 1903**

***Caenohalictus*** ***alexandrei* Celis, Cure & Aguilar-Benavides, 2014***

Country records: Cundinamarca: Bogotá, D.C.^1,5,7^, Cajicá^5,7^, San Antonio del Tequendama^7^, Tabio^5^, Zipaquirá†^5,7^.

***Caenohalictus*** ***colombianus* (Coelho & Engel, 2018) §***

**Country records**: Valle del Cauca: Él Cerrito†^(Coelho & Engel, 2018)^.

***Caenohalictus*** ***columbus*** **(Vachal, 1903) §**

**Country records**: Cundinamarca: Bogotá, D.C.† ^(Vachal, 1903)^.

***Caenohalictus*** ***eberhardorum*** Michener, 1979

**Country records**: Antioquia: Guarne^28^; Valle del Cauca: Dagua^20^, Cali†^20^.

***Caenohalictus* *lindigi* (Vachal, 1911)**

**Country records**: Cundinamarca: Bogotá, D.C.† ^(Vachal, 1911).^

***Caenohalictus* *modestus* (Smith, 1853)**

**Country records:** No specific location records^(Smith, 1853)^.

***Caenohalictus* *moritzi* Alfeken, 1932**

**Country records:** No specific location records^(Alfken, 1932)^.

***Caenohalictus* *sabanaensis*, Celis, Cure & Aguilar-Benavides, 2014***

**Country records**: Boyacá: Villa de Leyva^1^; Cundinamarca: Bogotá, D.C.†^1,5,7^, Guasca^7^, Tabio^7^.

**Genus *Dinagapostemon* Moure & Hurd, 1982**

***Dinagapostemon*** ***gigas*** (Friese, 1911)

**Country records:** Antioquia: Caldas^13,14^, Rionegro^14,28^; Caldas: Samaná^1^; Cauca: Popayán†^(Friese, 1911)^; Tolima: Rovira^15^; Santander: Él Carmen de Chucurí^13^.

***Dinagapostemon* cf. *orestes* Roberts & Brooks, 1987**

**Country records:** Cundinamarca: Choachí^7^.

**Genus *Habralictus* Moure, 1941**

***Habralictus*** ***bimaculatus* Michener, 1979**

**Country records:** Antioquia: Amalfi^14^, Rionegro^3^; Valle del Cauca: Buenaventura^17^, Dagua^20^, Cali†^28,20^.

***Habralictus* *xanthinus* (Cockerell, 1918)**

**Country records:** Antioquia: Amalfi^14^, Yolombó^28^.

***Habralictus*** ***xanthogastris* (Vachal, 1911) §**

**Country records:** Cundinamarca: Bogotá, D.C† ^(Vachal, 1911)^.

**Note:** According to Melo & Liz (2025), the specimens were collected by Alexander Lindig. Although the label reads “1♀ Bogotá au Koen. Mus. de Berlin,” Melo & Liz (2025) indicate that the locality likely corresponds to a place other than the city of Bogotá within the department of Cundinamarca, and that it was not possible to trace the exact collecting sites of Lindig.

**Genus *Rhinetula* Friese, 1922**

***Rhinetula*** ***denticrus* Friese, 1922**

**Country records:** Antioquia: Amalfi^14,28^, Medellín^14^, San Luís^14^.

**Halictini Thomson, 1869**

**Genus *Halictus* Latreille, 1804**

**Subgenus *Odontalictus* Robertson 1918**

***Halictus* (*Odontalictus*) *ligatus* Say, 1837**

**Country records**: Antioquia: Amalfi^14,28^, Caldas^14^, Cocorná^14^, Itagüí^14^, Medellín^14^, Santa Fe de Antioquia^14,7^, Yolombó^28^; Caldas: Chinchiná^28^, Riosucio^1^; Cundinamarca: La Vega^7^; Huila: Villavieja^7^; La Guajira: Dibulla; Magdalena: Aracataca^6^, El Retén^6^, Santa Marta^1,18,6,28^, Zona Bananera^6,28^; Risaralda: Pereira^28^; Santander: Barichara^28^, Enciso^7^, Socorro^7^; Tolima: Armero^3^, Coello^2^; Valle del Cauca: Dagua^20^, Cali^20^.

**Subgenus *Seladonia* Robertson 1918**

***Halictus* *(Seladonia)* *hesperus* Smith, 1862**

**Country records**: Cesar: Valledupar^28^; Magdalena: Santa Marta^6,28^; Huila: Villavieja^7^.

**Genus *Lasioglossum* Curtis, 1833**

**Subgenus *Dialictus* Robertson, 1902**

***Lasioglossum* *(Dialictus)* *breedi* Michener, 1979 §**

**Country records**: Valle del Cauca: Cali†^28,20^.

***Lasioglossum* *(Dialictus)* *santafense* Gonzalez & Rasmussen, 2011**

**Country records**: Cundinamarca: Bogotá, D.C.†^1,5,7^, Cajicá^7^, Mosquera^5^, Sopó^7^, Tabio^7^, Zipaquirá^7^.

**Note:** This species was originally described as *Lasioglossum* (*Dialictus*) *urbanus* González, 2006, and was later given the replacement name *Lasioglossum* *santafensis* Gonzalez & Rasmussen, 2010. However, according to some authors (Moure, Urban & Melo, 2022) who treat *Dialictus* as genus, this name replacement is unnecessary and the species is therefore regarded as *Dialictus* *urbanus* (González, 2006).

***Lasioglossum* *(Dialictus)* *seabrai* (Moure, 1956)**

**Country records**: Valle del Cauca: Dagua^20^.

**Sphecodini Schenck, 1869**

**Genus *Austrosphecodes* Michener, 1978**

***Austrosphecodes*** ***bogotensis* (Meyer, 1922) §**

**Country records:** Cundinamarca: Bogotá, D.C.†^(Meyer, 1922)^.

**Genus *Microsphecodes* Eickwort & Stage, 1972**

**Subgenus *Microsphecodes* Eickwort & Stage, 1972**

***Microsphecodes* *(Microsphecodes) kathleenae* (Eickwort, 1972)**

**Country records**: Valle del Cauca: Buenaventura^20^.

***Microsphecodes* *(Microsphecodes)* *trichommus* Michener, 1979 §**

**Country records**: Valle del Cauca: Cali†^20^, Dagua^28^.

***Microsphecodes* *(Microsphecodes)* *truncaticaudus* Michener, 1979**

**Country records**: Valle del Cauca: Cali†^20^, Dagua^20,28^, Palmira^1^.

**Megachilidae Latreille, 1802**

**Megachilinae Latreille, 1802**

**Anthidiini Ashmead, 1899**

**Genus *Anthidiellum* Cockerell, 1904**

**Subgenus *Loyalanthidium* Urban, 2001**

***Anthidiellum* *(Loyalanthidium)* *apicale* (Cresson, 1878)***

**Country records**: Huila: Villavieja^7^; Valle del Cauca: Dagua^24^.

**Genus *Anthidium* Fabricius, 1804**

***Anthidium* *sanguinicaudum* Schwarz, 1933**

**Country records:** Antioquia: Envigado^1^; Atlántico: Barranquilla^1^; Bolívar: Cartagena^1^, San Juan Nepomuceno^1^, Zambrano^18^; Casanare: Chámeza^1^, Sabanalarga^5^; Chocó: Acandí^13^; Cundinamarca: Apulo^1^; Huila: Paicol^1^, Rivera^1^, Villavieja^7^; Magdalena: Santa Marta^1^; Sucre: San Onofre^13^; Tolima: Coello^2^; Valle del Cauca: Cartago^28^, Dagua^20^, El Dovio^1^, Palmira^12^.

**Genus *Anthodioctes* Holmberg, 1903**

***Anthodioctes* *analuizae* (Urban, 1998)**

**Country records**: Antioquia: Yolombó^28^; Boyacá: Muzo† ^(Urban, 1998)^.

***Anthodioctes* *salti* (Schwarz, 1933) §**

**Country records**: Magdalena: Santa Marta†^(Schwarz, 1933)^.

**Genus *Austrostelis* Michener & Griswold, 1994**

***Austrostelis*** ***flava* (Friese, 1925)§**

**Country records:** Cauca: Popayan† ^(Friese, 1925b)^.

**Genus *Duckeanthidium* Moure & Hurd, 1960**

***Duckeanthidium*** ***silvicola* Guevara & Gonzalez, 2025 §***

**Country records:** Caquetá: Solano†^1^.

**Genus *Hoplostelis* Dominique, 1898**

***Hoplostelis* *bilineolata* (Spinola, 1841)**

**Country records**: Bolívar: San Juan Nepomuceno^1^; Boyacá: Buenavista^1^; Cesar: Curumaní^19^; Cundinamarca: Arbeláez^1^, La Mesa^1^; Meta: San Juan de Arama^1^; Santander: Cimitarra^1^, Floridablanca^1^; Valle del Cauca: El Cerrito^12^, Palmira^3^, Cali^28^.

**Genus *Hypanthidioides* Moure, 1947**

**Subgenus *Anthidulum* Michener, 1948**

***Hypanthidioides* *(Anthidulum)* *christinae* (Urban, 1993) §**

**Country records**: Boyacá: Muzo†^28^.

***Hypanthidioides* *(Anthidulum)* *currani* (Schwarz, 1933)**

**Country records**: Valle del Cauca: Dagua^20^.

**Subgenus *Dicranthidium* Moure & Urban, 1975**

***Hypanthidioides* (*Dicranthidium*) *colombiae* (Schwarz, 1933)**

**Country records**: Boyacá: Muzo^28^; Cesar: Pueblo Bello^28^; Cundinamarca: Agua de Dios^7^; Huila: Villavieja^7^; Magdalena: Santa Marta†^28^; Meta: Restrepo^28^; Valle del Cauca: Dagua^28^.

**Genus *Hypanthidium* Cockerell, 1904**

***Hypanthidium*** ***magdalenae* Urban, 1998**

**Country records:** Bolívar: Cartagena^19^; Cesar: Valledupar^28^; Cundinamarca: Agua de Dios^7^; Magdalena†^(Urban, 1997)^.

***Hypanthidium* cf. *mexicanum* (Cresson, 1878)**

**Country records**: Antioquia: Amalfi^28^, Yolombó^28^.

**Genus *Saranthidium* Moure & Hurd, 1960**

***Saranthidium*** ***flavopictum* (Smith, 1854)**

**Country records**: Boyacá: Muzo^28^; Magdalena: Santa Marta^28^.

***Saranthidium* *panamense* (Cockerell, 1913)***

**Country records**: Antioquia: Sonsón^18^.

**Genus *Stelis* Panzer, 1806**

**Subgenus *Dolichostelis* Parker & Bohart, 1979**

***Stelis* (*Dolichostelis*) *costaricensis* Frisese, 1921***

**Country records**: Antioquia: Andes^13^; Cundinamarca: Pacho^1^; Valle del Cauca: Dagua^20^.

***Stelis* (*Dolichostelis*) *vallenata* Gonzalez, Ascher & Engel, 2012***

**Country records**: Cesar: Pueblo Bello^28^, Valledupar†^19^; La Guajira: Maicao^20^.

**Megachilini Latreille, 1802**

**Genus *Coelioxys* Latreille, 1809**

**Subgenus *Acrocoelioxys***

***Coelioxys* *(Acrocoelioxys)* *azteca* Cresson, 1878***

**Country records**: Antioquia: Puerto Berrío^25^.

**Subgenus *Austrocleptria* Rocha-Filho, 2016**

***Coelioxys* *(Austrocleptria)* *nasidens* Friese, 1922***

**Country records**: Casanare: Villanueva^1^.

**Subgenus *Cyrtocoelioxys* Mitchell, 1973**

***Coelioxys* *(Cyrtocoelioxys)* *columbicus* Friese, 1922 §**

**Country records**: Cauca: Popayan† ^(Friese, 1922)^.

***Coelioxys* *(Cyrtocoelioxys)* *costaricensis* Cockerell, 1914**

**Country records**: Boyacá: Muzo^28^.

***Coelioxys* *(Cyrtocoelioxys) gonaspis* Cockerell, 1924**

**Country records:** Cesar: Valledupar^28^.

**Subgenus *Glyptocoelioxys* Mitchell, 1973**

***Coelioxys* *(Glyptocoelioxys) cayennensis* Spinola, 1841***

**Country records:** Huila: Villavieja^7^.

***Coelioxys* *(Glyptocoelioxys)* *mexicanus* Cresson, 1878***

**Country records**: Antioquia: Yolombó^28^.

**Subgenus *Neocoelioxys* Mitchell, 1973**

***Coelioxys* *(Neocoelioxys)* *abdominalis* Guérin-Méneville, 1845**

**Country records**: Atlántico: Puerto Colombia^28^; Cesar: Valledupar^28^; Huila: Villavieja^7^; La Guajira: Maicao^28^, Uribia^28^; Magdalena: Santa Marta^28^.

***Coelioxys* *(Neocoelioxys)* *laevigatus* Smith, 1854***

**Country records**: Meta: Restrepo^28^.

***Coelioxys* *(Neocoelioxys)* *otomita* Cresson, 1878**

**Country records**: Boyacá: Muzo^28^; Meta: Restrepo^28^.

***Coelioxys* *(Neocoelioxys)* *simillimus* Smith, 1854**

**Country records**: Antioquia: Yolombó^28^.

***Coelioxys* *(Neocoelioxys)* *tolteca* Cresson, 1878**

**Country records**: Boyacá: Muzo^28^.

**Subgenus *Platycoelioxys* Mitchell, 1973**

***Coelioxys* *(Platycoelioxys)* *alatiformis* Friese, 1922***

**Country records**: Arauca: Tame ^(Filho & Packer, 2015)^; Magdalena: Santa Marta ^(Filho & Packer, 2015)^; Meta: La Macarena ^(Filho & Packer, 2015)^; Quindío: La Tebaida^15^; Valle del Cauca: Dagua^20^.

**Subgenus *Rhinocoelioxys* Mitchell, 1973**

***Coelioxys* *(Rhinocoelioxys)* *agilis* Smith, 1879***

**Country records**: Amazonas: La Chorrera^(Filho & Packer, 2015)^, Leticia^(Filho & Packer, 2015)^, Tarapacá ^(Filho & Packer, 2015)^; Nariño: Barbacoas^(Filho & Packer, 2015)^; Putumayo: Mocoa^(Filho & Packer, 2015)^.

***Coelioxys* *(Rhinocoelioxys)* *barbatus* Schwarz & Michener, 1954***

**Country records**: Magdalena: Santa Marta^(Filho & Packer, 2015)^; Valle del Cauca: Dagua^20, (Filho & Packer, 2015)^.

***Coelioxys* *(Rhinocoelioxys)* *zapoteca* Cresson, 1878***

**Country records**: Caquetá: Curillo ^(Filho & Packer, 2015)^; Guaviare: Alamar^(Filho & Packer, 2015)^; Magdalena: Santa Marta ^(Filho & Packer, 2015)^.

**Genus *Megachile* Latreille, 1802**

**Subgenus *Acentron* Mitchell, 1943**

***Megachile* *(Acentron)* *candida* Smith, 1879**

**Country records**: Magdalena: Ciénaga^(Mitchell, 1930)^; Valle del Cauca: Sevilla^(Mitchell, 1930)^.

**Subgenus *Austromegachile* Mitchell, 1943**

***Megachile* *(Austromegachile)* *antiqua* Mitchell, 1930***

**Country records**: Boyacá: Muzo^28^; Cauca: Corinto^28^.

***Megachile* *(Austromegachile)* *habilis* Mitchell, 1930**

**Country records**: Magdalena: Santa Marta ^(Mitchell, 1930)^.

***Megachile* *(Austromegachile)* cf. *susurrans***

**Country records**: Antioquia: Yolombó^28^.

**Subgenus *Chelostomoides* Robertson, 1901**

***Megachile* *(Chelostomoides) cartagenensis* Mitchell, 1930**

**Country records**: Bolívar: Cartagena†^(Mitchell, 1930)^; La Guajira: Uribia^19,28^; Magdalena: Santa Marta^28^.

***Megachile*** ***(Chelostomoides)* *haematoxylonae* Mitchell, 1930 §**

**Country records**: Magdalena: Santa Marta†^(Mitchell, 1930)^.

***Megachile* *(Chelostomoides)* *otomita* Cresson, 1878**

Country records: Antioquia: Amalfi^14,20^, Medellín^14^, Rionegro^14^; Boyacá: Muzo^28^, Villa de Leyva^1^; Cauca: Popayán^(Mitchell, 1930)^; Cundinamarca: Anolaima^1^; Córdoba: Coveñas^14^; Magdalena: Santa Marta ^(Guevara, Ospina-Torres & Gonzalez, 2022)^ ; Valle del Cauca: Buga^12^, Dagua^12,20^, El Cerrito^12^, Palmira^12^, Cali^1,12,28^.

**Subgenus *Chrysosarus* Mitchell, 1943**

***Megachile* *(Chrysosarus)* *contempta* Mitchell, 1930**

**Country records**: Magdalena: Ciénaga ^(Mitchell, 1930)^.

**Subgenus *Cressoniella* Mitchell, 1934**

***Megachile* *(Cressoniella)* *amparo* Gonzalez, 2006**

**Country records:** Boyacá: Arcabuco^5^, Chíquiza†^1,5^, Sogamoso^1^, Ventaquemada^18^, Villa de Leyva^1,28^; Cundinamarca: Bogotá, D.C^1,3,7,28^, Choachí^1^, Chía^28^, Cota^1,28^, Fúquene^28^, Guasca^7^, La Calera^1^, Mosquera^5^, Sopó^7^, Tenjo^28^.

***Megachile* *(Cressoniella)* *arcus* Mitchell, 1930 §**

**Country records**: Magdalena: Santa Marta†^(Mitchell, 1930)^.

***Megachile* *(Cressoniella)* *fruticosa* Mitchell, 1930 §**

**Country records**: Magdalena†.

Note: In Mitchell (1930) work describing this species, the author provided the following information: “Colombia (?), (G. F. Baker), [U.S.N.M., no. 43093].” However, the labels of the holotype deposited in the 27 read: “Colom \ Collection C.F. Baker \ Type No. 43093 U.S.N.M. \ MEGACHILE fruticosa Mitchell Type \ 27 ENT 00536576.” This suggests that there is a typographical error in Mitchell (1930), as the collector was Charles Fuller Baker. The specimen most likely originated from Magdalena, Colombia, where C. F. Baker participated in H. H. Smith’s expedition between 1898 and 1891 (Essig, 1927).

**Subgenus *Eutricharaea* Thomson, 1872**

***Megachile* *(Eutricharaea)*** ***concinna* Smith, 1879 ◊***

**Country records**: Bolívar: Cartagena^1^.

**Subgenus *Leptorachina* Mitchell, 1980**

***Megachile* *(Leptorachina)* *lorenziensis* Mitchell, 1930**

**Country records**: Antioquia: Amalfi^20^; Cesar: Agustín Codazzi^19^; Magdalena: Santa Marta†^(Mitchell, 1930)^; Valle del Cauca: Cali^20^, El Dovio^1^.

**Subgenus *Leptorachis* Mitchell, 1934**

***Megachile* *(Leptorachis)* *colombiana* Mitchell, 1930**

**Country records**: Cauca: Popayán^28^; Magdalena: Ciénaga†^(Mitchell, 1930)^, Santa Marta^(Mitchell, 1930)^.

***Megachile* *(Leptorachis)* *indigoferae* Mitchell, 1930 §**

**Country records**: Magdalena: Ciénaga†^(Mitchell, 1930)^.

***Megachile* *(Leptorachis)* *paulistana* Schrottky, 1902**

**Country records**: Magdalena^(Mitchell, 1930)^

**Subgenus *Melanosarus* Mitchell, 1934**

***Megachile* *(Melanosarus)* *nigripennis* Spinola, 1841**

**Country records**: Amazonas: Leticia^(Raw, 2007)^.

**Subgenus *Moureapis* Raw, 2002**

***Megachile* *(Moureapis)* *trepida* Mitchell, 1930§**

**Country records**: Magdalena: Ciénaga†^(Mitchell, 1930)^.

**Subgenus *Neochelynia* Schrottky, 1920**

***Megachile* *(Neochelynia)* *clara* Mitchell, 1930**

**Country records**: Antioquia: Amalfi^28^, Yolombó^28^.

***Megachile* *(Neochelynia)* *praecipua* Mitchell, 1930 §**

**Country records**: Magdalena: Ciénaga†^(Mitchell, 1930)^.

***Megachile* *(Neochelynia) stomatura* Cockerell, 1917**

**Country records**: Atlántico: Puerto Colombia^28^; Bolívar: Cartagena^19, (Mitchell, 1930)^; La Guajira: Uribia^19,28^; Magdalena: Santa Marta^28^.

**Subgenus *Pseudocentron* Mitchell, 1934**

***Megachile* *(Pseudocentron)* *banksi* Mitchell, 1930**

**Country records**: Magdalena: Ciénaga^(Mitchell, 1930)^.

***Megachile* *(Pseudocentron)* *curvipes* Smith, 1853**

**Country records**: Magdalena: Ciénaga^(Mitchell, 1930)^.

***Megachile* *(Pseudocentron)* *furcata* Vachal, 1909**

**Country records:** Cesar: Valledupar^28^; Huila: Villavieja^7^; Magdalena: Ciénaga^(Mitchell, 1930)^, Santa Marta^(Mitchell, 1930)^, Zona Bananera^(Mitchell, 1930)^.

***Megachile* *(Pseudocentron)* cf. *luctifera***

**Country records**: Cesar: Agustín Codazzi^3^.

***Megachile* *(Pseudocentron) rubricata* Smith, 1853***

**Country records**: Meta: Restrepo^25^.

**Subgenus *Pseudomegachile* Mitchell, 1934**

***Megachile* *(Pseudomegachile)* *lanata* (Fabricius, 1775)*◊**

**Country records**: Antioquia: Medellín^13^; Cesar: Valledupar^1^; La Guajira: Riohacha^1^; Magdalena: Ciénaga Grande^5,28^; Meta: Puerto López^1^.

**Subgenus *Ptilosaroides* Mitchell, 1980**

***Megachile* *(Ptilosaroides)* *pilosa* Smith, 1879***

**Country records**: Huila: Villavieja^7^.

**Subgenus *Ptilosarus* Mitchell, 1943**

***Megachile* *(Ptilosarus)* *microsoma* Cockerell, 1912***

**Country records**: Meta: Restrepo^28^.

**Subgenus *Rhyssomegachile* Mitchell, 1980**

***Megachile* *(Rhyssomegachile)* *simillima* Smith, 1853***

**Country records**: Amazonas: Leticia^20^; Vaupés: Mitú ^(Guevara, Ospina-Torres & Gonzalez, 2022)^.

**Subgenus *Sayapis* Titus, 1906**

***Megachile* *(Sayapis)* *zaptlana* Cresson, 1878**

**Country records**: Antioquia: Yolombó^28^; Atlántico: Puerto Colombia^28^; La Guajira: Uribia^28^; Magdalena: Ciénaga^(Mitchell, 1930)^, Santa Marta^28^.

**Subgenus *Tylomegachile* Moure, 1953**

***Megachile* *(Tylomegachile)* *hamata* Mitchell, 1930**

**Country records**: Magdalena: Zona Bananera†^(Mitchell, 1930)^; Santander: Enciso^7^.

***Megachile* *(Tylomegachile)* *orba* Schrottky, 1913**

**Country records**: Magdalena: Zona Bananera^(Mitchell, 1930)^.

**Subgenus *Zonomegachile* Mitchell, 1980**

***Megachile* *(Zonomegachile)* *kalina* Gonzalez, Griswold & Engel, 2018***

**Country records**: Vichada: Cumaribo ^(Guevara, Ospina-Torres & Gonzalez, 2022)^.

***Megachile* *(Zonomegachile)* *moderata* Smith, 1879***

**Country records**: Amazonas: Leticia^20^, Mirití-Paraná^(Guevara, Ospina-Torres & Gonzalez, 2022)^; Meta: La Macarena^(Guevara, Ospina-Torres & Gonzalez, 2022)^, Puerto Rico^(Guevara, Ospina-Torres & Gonzalez, 2022)^; Vaupés: Mitú^(Guevara, Ospina-Torres & Gonzalez, 2022)^.

***Megachile* *(Zonomegachile)* *paisa* Gonzalez, Griswold & Engel, 2018*†**

**Country records**: Antioquia: Amalfi† ^(Gonzalez, Griswold & Engel, 2018)^.

**Osmiini Newman, 1834**

**Genus *Heriades* Spinola, 1808**

**Subgenus *Neotrypetes* Robertson, 1918**

***Heriades* *(Neotrypetes)* *currani* Michener, 1943***

**Country records:** Cesar: Valledupar^1^; Magdalena: Santa Marta^1,18,28^.

**Genus *Osmia* Panzer, 1806**

**Subgenus *Diceratosmia***

***Osmia* *(Diceratosmia)* *azteca* Cresson, 1878 ***

**Country records**: Antioquia: Mutatá^15^.

**References**

Aguiar AJC. 2009. Taxonomic revision of the bee genus *Lophopedia* Michener and Moure (Hymenoptera, Apidae, Tapinotaspidini). *Zootaxa* 2193. DOI: 10.11646/zootaxa.2193.1.1.

Alfken JD. 1932. Die chilenischen Arten der Gattung *Caenohalictus* Cam. *Archiv für Naturgeschichte* 1:654–659.

Bembé B. 2007. Revision der *Euglossa* *cordata*-gruppe und untersuchungen zur funktionsmorphologie und faunistik der Euglossini (Hymenoptera, Apidae). *Entomofauna* 14:1–146.

Buysson R. 1901. Catalogue des hyménoptères melliférides des collections du muséum. *Bulletin du Muséum d’histoire naturelle* 7:207–214.

Camargo JMF, Pedro SRM. 2008. Revisão das espécies de *Melipona* do grupo *fuliginosa* (Hymenoptera, Apoidea, Apidae, Meliponini). *Revista Brasileira de Entomologia* 52:411–427. DOI: 10.1590/S0085-56262008000300014.

Celis CJ, Melo GAR. 2022. Taxonomic revision of *Augochloropsis* (*Glyptochlora*) Moure, a rare bee subgenus from the Amazon Basin (Hymenoptera: Halictinae). *Zootaxa* 5182:21–40. DOI: 10.11646/zootaxa.5182.1.2.

Cheesman LE. 1929. Hymenoptera collected on the “St. George” expedition in Central America and the W. Indies. *Transactions of the Royal Entomological Society of London* 77:141–154. DOI: 10.1111/j.1365-2311.1929.tb00683.x.

Coelho BWT, Engel MS. 2018. A new group of species within the bee genus *Ruizantheda*, with a revised key to the males of the genus (Hymenoptera: Halictidae: Caenohalictini). *Zootaxa* 4415. DOI: 10.11646/zootaxa.4415.3.6.

Engel MS. 1996. Taxonomic and Geographic Notes on Some Halictine Bee Species (Hymenoptera: Halictidae). *Journal of the New York Entomological Society* 104:106–110.

Engel MS. 1997a. Two new species of the neotropical bee genus *Caenaugochlora* (s. str.) Michener (Insecta: Hymenoptera: Halictidae: Augochlorini). *Reichenbachia* 32:91–95.

Engel MS. 1997b. *Ischnomelissa*, a New Genus of Augochlorine Bees (Halictidae) from Colombia. *Studies on Neotropical Fauna and Environment* 32:41–46. DOI: 10.1076/snfe.32.1.41.13461.

Engel M. 2010. Revision of the Bee Genus *Chlerogella* (Hymenoptera, Halictidae), Part II: South American Species and Generic Diagnosis. *ZooKeys* 47:1–100. DOI: 10.3897/zookeys.47.416.

Engel MS. 2014. Three new species of the genus *Caenaugochlora* from Central America and Colombia (Hymenoptera: Halictidae). *Journal of Melittology*:1–15.

Engel MS, Brooks RW. 1999. The augochlorine bee genus *Megaloptilla* (Hymenoptera: Halictidae). In: *Entomological contributions in memory of Byron A. Alexander, Special Publication*. 24. Lawrence, Kansas: University of Kansas Natural History Museum, 9–15.

Engel MS, Engel MS, Brooks RW, Yanega D. 1997. *New genera and subgenera of Augochlorine bees (Hymenoptera: Halictidae)*. Lawrence, Kan: Natural History Museum, The University of Kansas. DOI: 10.5962/bhl.title.4042.

Engel MS, González VH, Hinojosa-Díaz IA. 2014. Revision of the bee genus *Chlerogella* (Hymenoptera: Halictidae), Part IV: A new species from southwestern Colombia. *Journal of Melittology*:1–9.

Engel MS, Guevara DA, Ospina-Torres R, Gonzalez VH. 2021. The bee genus *Psaenythia* in northern South America (Hymenoptera: Andrenidae). *Entomologist’s Monthly Magazine* 157:153–167. DOI: 10.31184/M00138908.1573.4082.

Essig E. 1927. Charles Fuller Baker. *Journal of Economic Entomology* 20:748–754.

Filho LCDR, Packer L. 2015. Revision of the Neotropical subgenera *Coelioxys* (*Platycoelioxys*) Mitchell and *C*. (*Rhinocoelioxys*) Mitchell (Hymenoptera; Megachilidae) with the description of one new species. *Zootaxa* 3941. DOI: 10.11646/zootaxa.3941.2.1.

Flórez-Gómez N, Ayala R, Hinojosa-Díaz I, Morrone JJ. 2022. The neotropical species of small carpenter bees of the subgenus *Ceratina* (*Zadontomerus*) Ashmead, 1899 (Hymenoptera: Apidae). *Zootaxa* 5214:189–223. DOI: 10.11646/zootaxa.5214.2.3.

Flórez-Gómez NA, Griswold T. 2020. A new bee species of the subgenus *Ceratina* (*Rhysoceratina*) (Hymenoptera: Apidae) from northernmost South America. *Revista Mexicana de Biodiversidad* 91:e913062. DOI: 10.22201/ib.20078706e.2020.91.3062.

Friese H. 1899a. Neue Arten der Bienengattungen *Epicharis* Klug und *Centris* Fabr. *Természetrajzi Füzetek kiadja a Magyar nemzeti Muzeum* 23:39–48.

Friese H. 1899b. Monographie der Bienengattungen *Exomalopsis*, *Ptilothrix*, *Melitoma* und *Tetrapedia*. *Annalen des K.K. Naturhistorischen Hofmuseums* 14:247–304.

Friese H. 1899c. Monographie der Bienengattungen *Megacilissa*, *Caupolicana* und *Oxaea*. *Annalen des K.K. Naturhistorischen Hofmuseums* 14:239–246.

Friese H. 1903. Neue Arten der Bienengattung *Xylocopa* Latr. aus der neotropischen und orientalischen Region. (Hym.). *Zeitschrift für systematische hymenopterologie und dipterologie* 3:202–208.

Friese H. 1910. Neue Bienen aus Süd-Amerika. (Hym.). *Deutsche Entomologische Zeitschrift* 1910:693–711.

Friese H. 1911. Neue Bienen aus Süd-Amerika. (Hym.). *Deutsche Entomologische Zeitschrift* 1911:453–456. DOI: 10.1002/mmnd.48019110414.

Friese Heinrich. 1916. Zur Bienenfauna von Costa Rica. (Hym.). *Stettiner Entomologische Zeitung* 77:287–348.

Friese H. 1922. Über die Kegelbienen (*Coelioxys*) Brasiliens. *Zoologische Jahrbücher. Abteilung für Systematik, Geographie und Biologie der Tiere* 44:421–486.

Friese H. 1925a. Neue neotropische Bienenarten, zugleich II. Nachtrag zur Bienenfauna von Costa Rica. (Hym.). *Stettiner Entomologische Zeitung* 86:1–41.

Friese H. 1925b. Neue Formen von Schmarotzerbienen, besonders aus dem paläarktischen Gebiet. *Konowia* 4:27–42.

Friese H. 1926. Die Nachtbienen-Gattung *Megalopta* Sm. *Stettiner Entomologische Zeitung* 87:11–135.

Gonçalves RB. 2010. Phylogeny and revision of the Neotropical bee genus *Rhectomia* s.l. Moure (Hymenoptera, Apidae, Augochlorini). *Systematic Entomology* 35:90–117. DOI: 10.1111/j.1365-3113.2009.00491.x.

Gonçalves RB. 2019. New species of *Neocorynura* Schrottky from Brazil (Hymenoptera: Apoidea). *Zootaxa* 4542:1–132. DOI: 10.11646/zootaxa.4542.1.1.

Gonçalves RB, Lepeco A. 2026. Taxonomic Revision of the Subgenus *Augochlora* (*Augochlora*) Smith (Apoidea, Halictinae) in Brazil. *Zootaxa* 5803:1–127. DOI: 10.11646/zootaxa.5803.1.1.

Gonzalez VH, Engel MS. 2015. A new species of *Lonchopria* Vachal from Norte de Santander, Colombia (Hymenoptera: Colletidae). In: Aguiar AJC, Gonçalves RB, Ramos KS eds. *Ensaios sobre as Abelhas da região Neotropical: Homenagem aos 80 anos de Danuncia Urban*. Curitiba, Paraná: Universidade Federal do Paraná, 235–244.

Gonzalez VH, Giraldo C. 2009. New Andean Bee Species of *Chilicola* Spinola (Hymenoptera: Colletidae, Xeromelissinae) With Notes on Their Biology. *Caldasia* 31:145–154.

Gonzalez VH, Griswold TL. 2011. Two new species of *Paratrigona* and the male of *Paratrigona ornaticeps* (Hymenoptera, Apidae). *ZooKeys* 120:9–25. DOI: 10.3897/zookeys.120.1732.

Gonzalez VH, Griswold terry. 2012. New species and previously unknown males of Neotropical cleptobiotic stingless bees (Hymenoptera, Apidae, *Lestrimelitta*). *Caldasia* 34:227–245.

Gonzalez VH, Griswold T, Engel MS. 2018. South American Leaf-Cutter Bees (Genus *Megachile*) of the Subgenera *Rhyssomegachile* and *Zonomegachile*, with Two New Subgenera (Hymenoptera: Megachilidae). *Bulletin of the American Museum of Natural History* 2018:1. DOI: 10.1206/00030090-425.1.1.

Gonzalez VH, Michener CD. 2004a. Application of Specific Names and Association of Sexes in *Cadegualina* Michener (Hymenoptera: Colletidae: Diphaglossini). *Proc. Entomol. Soc. Wash* 106:851–857.

Gonzalez VH, Michener CD. 2004b. A New *Chilicola* Spinola from Colombian Paramo (Hymenoptera: Colletidae: Xeromelissinae). 131:24–30.

Gonzalez V, Ospina M. 2006. Nuevos registros genéricos de abejas (Hymenoptera: Apoidea) para Colombia. *Acta Biológica Colombiana* 11:89–90.

González VH, Roubik DW. 2008. Especies nuevas y filogenia de las abejas de fuego, *Oxytrigona* (Hymenoptera: Apidae, Meliponini). *Acta Zoológica Mexicana* 24. DOI: 10.21829/azm.2008.241615.

Gribodo G. 1891. Contribuzioni imenotterologiche. Sopra alcune specie nuove o poco conosciute di imenotteri antofili (generi *Ctenoplectra*, *Xylocopa*, *Centris*, *Psithyrus*, *Trigona*, e *Bombus*). Nota III. *Bollettino della Società entomologica italiana* 23:102–119.

Guevara DA, Ospina-Torres R, Gonzalez VH. 2022. New records of megachilid bees (Hymenoptera: Megachilidae) for Colombia. *Revista Peruana de Biología* 29:e22935. DOI: 10.15381/rpb.v29i4.22935.

Hinojosa-Díaz IA, Engel MS. 2014. Revision of the orchid bee subgenus *Euglossella* (Hymenoptera: Apidae), part II: The *viridis* and *mandibularis* species groups. *Journal of Melittology*:1–108. DOI: 10.17161/jom.v0i36.4777.

Kimsey LS. 1977. New species of bees in the genera *Euplusia* and *Eufriesia*. (Hymenoptera: Apidae, Euglossini). *The Pan-Pacific entomologist* 53:8–18.

Lepeco A. 2024. Contribution to the taxonomy of *Oxytrigona* Cockerell, 1917 (Apidae, Meliponini). *Zootaxa* 5506:369–395. DOI: 10.11646/zootaxa.5506.3.4.

Lepeco A, Gonçalves RB. 2023. A review of *Augochlora* (*Oxystoglossella*) bees from South America: unexpected Amazonian diversity and assessment of vulnerable species. *Journal of Natural History* 57:608–664. DOI: 10.1080/00222933.2023.2195564.

Londoño-Carvajal CA. 2022. Abejas del género *Eufriesea* Cockerell, 1908 (Apidae: Euglossini): Actualización sobre presencia y distribución en Colombia. Medellín: Universidad Nacional de Colombia.

Maa T. 1940. On the nomenclature of certain *Xylocopa* species (Hymen.). *Notes Entomologie Chinoise, Shanghai* 7:131–138.

Mahlmann T. 2023. Sistemática de *Ceratina* (*Calloceratina*) Cockerell, 1924 (Hymenoptera: Apidae: Xylocopinae) e sua relação com os demais subgêneros de *Ceratina* no Novo Mundo. Manaus, Amazonas, Brasil: Instituto Nacional de Pesquisas da Amazônia.

Mahlmann T, Oliveira FF de, Oliveira ML de. 2023. Taxonomy of South American species of *Ceratina* (*Calloceratina*) Cockerell, 1924 with comments on new species-groups proposed for this subgenus (Hymenoptera: Apidae: Xylocopinae). *EntomoBrasilis* 16:e1062–e1062. DOI: 10.12741/ebrasilis.v16.e1062.

Melo GAR, Liz JAD. 2025. The type material of the Neotropical bee genus *Habralictus* Moure (Apidae, Halictinae) in the Museum für Naturkunde, Berlin, Germany. *Papéis Avulsos de Zoologia* 65:e202565005. DOI: 10.11606/1807-0205/2025.65.005.

Meyer R. 1922. Nachtrag zur Bienengattung *Sphecodes* Latr. *Archiv für Naturgeschichte. Abteilung A* 88:165–174.

Michener CD. 2002. The bee genus *Chilicola* in the Tropical Andes, with observations on nesting biology and a Phylogenetic analisys of the subgenera (Hymenoptera: colletida, Xeromelissinae). *The University of Kansas Science Bulletin* 26:1–47.

Mitchell TB. 1930. A contribution to the knowledge of neotropical Megachile with descriptions of new species (Hymenoptera: Megachilidae). *Transactions of the American Entomological Society (1890-)* 56:155–306.

Moure JS. 1987. Contribuição para o conhecimento do gênero *Ptiloglossa* (Hymenoptera, Colletidae). *Acta Biológica Paranaense* 16. DOI: 10.5380/abpr.v16i0.813.

Moure JS. 1999. Tipos de Halictidae de Vachal no Naturkunde Museum, Berlin (Hymenoptera, Apoidea). *Revista Brasileira de Zoologia* 16:73–89. DOI: 10.1590/S0101-81751999000500003.

Moure JS, Hurd PD. 1987. *An Annotated Catalog of the Halictid Bees of the Western Hemisphere (Hymenoptera: Halictidae)*. Washington: Smithsonian Institution Press.

Moure JS, Urban D, Melo GA. 2022.Catalogue of Bees (Hymenoptera, Apoidea) in the Neotropical Region. *Available at* *http://moure.cria.org.br/catalogue* (accessed March 20, 2024).

Nogueira DS, Rasmussen C, Oliveira ML. 2021. A New Species of *Tetragona* Lepeletier & Serville, 1828 from the “*truncata* group” and New Distribution Records of T. truncata Moure, 1971 (Hymenoptera: Apidae). *Neotropical Entomology* 50:68–77. DOI: 10.1007/s13744-020-00822-6.

Odanaka KA, Branstetter MG, Tobin KB, Rehan SM. 2022. Phylogenomics and historical biogeography of the cleptoparasitic bee genus *Nomada* (Hymenoptera: Apidae) using ultraconserved elements. *Molecular Phylogenetics and Evolution* 170:107453. DOI: 10.1016/j.ympev.2022.107453.

Onuferko TM, Rightmyer MG, Melo GAR, Roig-Alsina A. 2024. A revision of the South American species of the cleptoparasitic bee genus *Triepeolus* Robertson, 1901 (Hymenoptera: Apidae). *European Journal of Taxonomy* 931:1–50. DOI: 10.5852/ejt.2024.931.2505.

Pedro SRM, Camargo JMF. 2003. Meliponini neotropicais: o gênero *Partamona* Schwarz, 1939 (Hymenoptera, Apidae). *Revista Brasileira de Entomologia*.

Pedro SRM, Camargo JMF. 2009. Neotropical Meliponini: the genus *Leurotrigona* Moure two new species (Hymenoptera: Apidae, Apinae). *Zootaxa* 1983:23–44. DOI: 10.11646/zootaxa.1983.1.2.

Raw A. 2007. An annotated catalogue of the leafcutter and mason bees (genus Megachile) of the Neotropics. *Zootaxa* 1601. DOI: 10.11646/zootaxa.1601.1.1.

Roberts RB, Roberts RB. 1972. Revision of the bee genus *Agapostemon* (Hymenoptera: Halictidae). *The University of Kansas science bulletin* 49:437--590.

Roig Alsina A. 2016. A revision of the bee genus *Ceratina*, subgenus *Rhysoceratina* (Hymenoptera, Apidae, Xylocopinae). *Revista del Museo Argentino de Ciencias Naturales* 18:125–146. DOI: 10.22179/REVMACN.18.473.

Roig-Alsina A. 2022. Systematic studies of the subgenus *Crewella* of *Ceratina* (Hymenoptera, Apidae, Xylocopinae), with a revision of the species occurring in Argentina. *Revista del Museo Argentino de Ciencias Naturales*.

Santos LM, Melo GAR. 2015. Updating the taxonomy of the bee genus *Megalopta* (Hymenoptera: Apidae, Augochlorini) including revision of the Brazilian species. *Journal of Natural History* 49:575–674. DOI: 10.1080/00222933.2014.946106.

Schrottky C. 1920. Himenopteros nuevos o poco conocidos sudamericanos. *Revista do Museu Paulista* 12:177–227.

Schwarz HF. 1933. Some Neotropical Anthidiinae: *Anthidium*, *Heteranthidium*, and *Dianthidium*. *American Museum novitates* 624:1–27.

Shanks SS. 1986. A revision of the neotropical bee genus *Osiris* (Hymenoptera: Anthophoridae). *The Wasmann Journal of Biology* 44:1–56.

Silveira FA, Almeida EAB. 2009. Revision of the species of the subgenera of *Exomalopsis* Spinola, 1853 ocurring in South America. II – *Phanomalopsis* Michener & Moure, 1957 (Hymenoptera, Apidae). *Lundiana: International Journal of Biodiversity* 9:111–153. DOI: 10.35699/2675-5327.2008.23816.

Smith F. 1853. *Catalogue of Hymenopterous Insects in the Collection of the British Museum. Part I. Andrenidae and Apidae*. London: London, Printed by order of the Trustees.

Timberlake PH. 1980. Review of North American *Exomalopsis*. *University of California publications in entomology* 86:1–158.

Urban D. 1967. As espécies do gênero *Thygater* Holmberg, 1884:(Hymenoptera, Apoidea). *Zoologia* 2:177–309.

Urban D. 1997. Notas taxonôrnicas e espécies novas de Hypanthidium Cockerell (Hymenoptera, Megachilidae). *Acta Biológica Paranaense* 26:95–103. DOI: 10.5380/abpr.v26i0.692.

Urban D. 1998. Notas taxonômicas e espécies novas de *Nananthidium* Moure e descrição do macho de *Bothranthidium* Moure (Hymenoptera, Megachilidae). *Revista Brasileira de Zoologia* 15:621–632. DOI: 10.1590/S0101-81751998000300008.

Vachal J. 1903. Étude sur les Halictus d’Amerique (Hym.). *Miscellanea entomologica* 11:121–136.

Vachal J. 1911. Étude sur les Halictus d’Amérique (Hym.). *Miscellanea entomologica* 19:9–24.

Velez-Ruiz RI. 2015. A Revision of the Bee Genus *Ptiloglossa* Smith (Hymenoptera: Colletidae: Diphaglossinae). South Dakota State University.

Villamizar G, Fernández F, Vivallo F. 2020. Synopsis of the carpenter bee subgenus *Xylocopa* (*Schonnherria*) Lepeletier, 1841 (Hymenoptera: Apidae) in Colombia, with designation of lectotypes and the description of two new species. *Zootaxa* 4789. DOI: 10.11646/zootaxa.4789.2.1.

Villamizar G, Vivallo F. 2024. Taxonomic revision of the oil-collecting bee subgenus *Epicharis* (*Epicharitides*) Moure, 1945 (Hymenoptera: Apidae), with the description of two new species. *European Journal of Taxonomy* 928. DOI: 10.5852/ejt.2024.928.2477.

Vivallo F. 2021. Two new species of oil-collecting bees of *Centris* (*Melanocentris*) from Colombia (Hymenoptera: Apidae). *Iheringia - Serie Zoologia* 111:1–5. DOI: 10.1590/1678-4766e2021019.

Vivallo F. 2022a. *The oil-collecting bees of* Centris *(*Melanocentris*) (Hymenoptera:Apidae)*. Rio de Janeiro: Author’s edition.

Vivallo F. 2022b. *The oil-collecting bees of Centris (Aphemisia)/ (Schisthemisia) (Hymenoptera: Apidae)*. Rio de Janeiro: Author’s edition.

Vivallo F. 2024. *De Apibus phantasticis generis Centris descripti a Johan Fabricius in 1804 (stricto sensu), cum descriptione specierum novarum, ubi occurrunt, flores quae visitant et quomodo eas cognoscimus*. Rio de Janeiro: Author’s edition.

Vivallo F, Vélez D. 2016. A synopsis of the subgenus *Centris* (*Hemisiella*) moure, 1945 (Hymenoptera: Apidae: Centridini) in Colombia, with description of a new species. *Zootaxa* 4162:107–133. DOI: 10.11646/zootaxa.4162.1.5.

Zama PC, Silveira FA. 2024. Descriptions of a new species and of three unknown males of *Xylocopa* Latreille, 1802 from the Neotropical region (Hymenoptera: Apidae: Xylocopini). *Zootaxa* 5404:54–72. DOI: 10.11646/zootaxa.5404.1.5.
